# Subcortical volumes across the lifespan: Data from 18,605 healthy individuals aged 3–90 years

**DOI:** 10.1002/hbm.25320

**Published:** 2021-02-11

**Authors:** Danai Dima, Amirhossein Modabbernia, Efstathios Papachristou, Gaelle E. Doucet, Ingrid Agartz, Moji Aghajani, Theophilus N. Akudjedu, Anton Albajes‐Eizagirre, Dag Alnæs, Kathryn I. Alpert, Micael Andersson, Nancy C. Andreasen, Ole A. Andreassen, Philip Asherson, Tobias Banaschewski, Nuria Bargallo, Sarah Baumeister, Ramona Baur‐Streubel, Alessandro Bertolino, Aurora Bonvino, Dorret I. Boomsma, Stefan Borgwardt, Josiane Bourque, Daniel Brandeis, Alan Breier, Henry Brodaty, Rachel M. Brouwer, Jan K. Buitelaar, Geraldo F. Busatto, Randy L. Buckner, Vincent Calhoun, Erick J. Canales‐Rodríguez, Dara M. Cannon, Xavier Caseras, Francisco X. Castellanos, Simon Cervenka, Tiffany M. Chaim‐Avancini, Christopher R. K. Ching, Victoria Chubar, Vincent P. Clark, Patricia Conrod, Annette Conzelmann, Benedicto Crespo‐Facorro, Fabrice Crivello, Eveline A. Crone, Udo Dannlowski, Anders M. Dale, Christopher Davey, Eco J. C. de Geus, Lieuwe de Haan, Greig I. de Zubicaray, Anouk den Braber, Erin W. Dickie, Annabella Di Giorgio, Nhat Trung Doan, Erlend S. Dørum, Stefan Ehrlich, Susanne Erk, Thomas Espeseth, Helena Fatouros‐Bergman, Simon E. Fisher, Jean‐Paul Fouche, Barbara Franke, Thomas Frodl, Paola Fuentes‐Claramonte, David C. Glahn, Ian H. Gotlib, Hans‐Jörgen Grabe, Oliver Grimm, Nynke A. Groenewold, Dominik Grotegerd, Oliver Gruber, Patricia Gruner, Rachel E. Gur, Ruben C. Gur, Tim Hahn, Ben J. Harrison, Catharine A. Hartman, Sean N. Hatton, Andreas Heinz, Dirk J. Heslenfeld, Derrek P. Hibar, Ian B. Hickie, Beng‐Choon Ho, Pieter J. Hoekstra, Sarah Hohmann, Avram J. Holmes, Martine Hoogman, Norbert Hosten, Fleur M. Howells, Hilleke E. Hulshoff Pol, Chaim Huyser, Neda Jahanshad, Anthony James, Terry L. Jernigan, Jiyang Jiang, Erik G. Jönsson, John A. Joska, Rene Kahn, Andrew Kalnin, Ryota Kanai, Marieke Klein, Tatyana P. Klyushnik, Laura Koenders, Sanne Koops, Bernd Krämer, Jonna Kuntsi, Jim Lagopoulos, Luisa Lázaro, Irina Lebedeva, Won Hee Lee, Klaus‐Peter Lesch, Christine Lochner, Marise W. J. Machielsen, Sophie Maingault, Nicholas G. Martin, Ignacio Martínez‐Zalacaín, David Mataix‐Cols, Bernard Mazoyer, Colm McDonald, Brenna C. McDonald, Andrew M. McIntosh, Katie L. McMahon, Genevieve McPhilemy, Susanne Meinert, José M. Menchón, Sarah E. Medland, Andreas Meyer‐Lindenberg, Jilly Naaijen, Pablo Najt, Tomohiro Nakao, Jan E. Nordvik, Lars Nyberg, Jaap Oosterlaan, Víctor Ortiz‐García de la Foz, Yannis Paloyelis, Paul Pauli, Giulio Pergola, Edith Pomarol‐Clotet, Maria J. Portella, Steven G. Potkin, Joaquim Radua, Andreas Reif, Daniel A. Rinker, Joshua L. Roffman, Pedro G. P. Rosa, Matthew D. Sacchet, Perminder S. Sachdev, Raymond Salvador, Pascual Sánchez‐Juan, Salvador Sarró, Theodore D. Satterthwaite, Andrew J. Saykin, Mauricio H. Serpa, Lianne Schmaal, Knut Schnell, Gunter Schumann, Kang Sim, Jordan W. Smoller, Iris Sommer, Carles Soriano‐Mas, Dan J. Stein, Lachlan T. Strike, Suzanne C. Swagerman, Christian K. Tamnes, Henk S. Temmingh, Sophia I. Thomopoulos, Alexander S. Tomyshev, Diana Tordesillas‐Gutiérrez, Julian N. Trollor, Jessica A. Turner, Anne Uhlmann, Odile A. van den Heuvel, Dennis van den Meer, Nic J. A. van der Wee, Neeltje E. M. van Haren, Dennis van't Ent, Theo G. M. van Erp, Ilya M. Veer, Dick J. Veltman, Aristotle Voineskos, Henry Völzke, Henrik Walter, Esther Walton, Lei Wang, Yang Wang, Thomas H. Wassink, Bernd Weber, Wei Wen, John D. West, Lars T. Westlye, Heather Whalley, Lara M. Wierenga, Steven C. R. Williams, Katharina Wittfeld, Daniel H. Wolf, Amanda Worker, Margaret J. Wright, Kun Yang, Yulyia Yoncheva, Marcus V. Zanetti, Georg C. Ziegler, Paul M. Thompson, Sophia Frangou, Karolinska Schizophrenia Project (KaSP)

**Affiliations:** ^1^ Department of Psychology, School of Arts and Social Sciences City University of London London UK; ^2^ Department of Neuroimaging, Institute of Psychiatry, Psychology and Neuroscience King's College London London UK; ^3^ Department of Psychiatry Icahn School of Medicine at Mount Sinai New York New York USA; ^4^ Psychology and Human Development, Institute of Education University College London London UK; ^5^ Boys Town National Research Hospital Omaha Nebraska USA; ^6^ Norwegian Centre for Mental Disorders Research (NORMENT), Institute of Clinical Medicine University of Oslo Oslo Norway; ^7^ Department of Psychiatric Research Diakonhjemmet Hospital Oslo Norway; ^8^ Centre for Psychiatric Research, Department of Clinical Neuroscience Karolinska Institutet Stockholm Sweden; ^9^ Department of Psychiatry, Amsterdam University Medical Centre Location VUmc Amsterdam Netherlands; ^10^ Institute of Education & Child Studies Section Forensic Family & Youth Care, Leiden University Netherlands; ^11^ Institute of Medical Imaging and Visualisation, Department of Medical Science and Public Health, Faculty of Health and Social Sciences Bournemouth University Poole UK; ^12^ Clinical Neuroimaging Laboratory, Centre for Neuroimaging and Cognitive Genomics and NCBES Galway Neuroscience Centre National University of Ireland Dublin Ireland; ^13^ FIDMAG Germanes Hospitalàries Madrid Spain; ^14^ Mental Health Research Networking Center (CIBERSAM) Madrid Spain; ^15^ Division of Mental Health and Addiction, Institute of Clinical Medicine University of Oslo Oslo Norway; ^16^ Radiologics, Inc Chicago Illinois USA; ^17^ Department of Integrative Medical Biology Umeå University Umeå Sweden; ^18^ Department of Psychiatry, Carver College of Medicine The University of Iowa Iowa City Iowa USA; ^19^ Social, Genetic and Developmental Psychiatry Centre, Institute of Psychiatry, Psychology and Neuroscience King's College London London UK; ^20^ Department of Child and Adolescent Psychiatry and Psychotherapy, Central Institute of Mental Health Heidelberg University Mannheim Germany; ^21^ Imaging Diagnostic Centre, Hospital Clinic Barcelona University Clinic Barcelona Spain; ^22^ August Pi i Sunyer Biomedical Research Institut (IDIBAPS) Barcelona Spain; ^23^ Department of Psychology, Biological Psychology, Clinical Psychology and Psychotherapy University of Würzburg Wurzburg Germany; ^24^ Department of Basic Medical Science, Neuroscience and Sense Organs University of Bari Aldo Moro Bari Italy; ^25^ Department of Biological Psychology Vrije Universiteit Amsterdam Netherlands; ^26^ Department of Psychiatry & Psychotherapy University of Lübeck Lubeck Germany; ^27^ Department of Psychiatry University of Pennsylvania Philadelphia Pennsylvania USA; ^28^ Department of Radiology and Imaging Sciences Indiana University School of Medicine Indianapolis Indiana USA; ^29^ Centre for Healthy Brain Ageing, School of Psychiatry University of New South Wales Sydney Australia; ^30^ Rudolf Magnus Institute of Neuroscience University Medical Center Utrecht Utrecht Netherlands; ^31^ Donders Center of Medical Neurosciences Radboud University Nijmegen Netherlands; ^32^ Donders Centre for Cognitive Neuroimaging Radboud University Nijmegen Netherlands; ^33^ Donders Institute for Brain, Cognition and Behaviour Radboud University Nijmegen Netherlands; ^34^ Laboratory of Psychiatric Neuroimaging, Departamento e Instituto de Psiquiatria, Hospital das Clinicas HCFMUSP, Faculdade de Medicina Universidade de São Paulo São Paulo Brazil; ^35^ Department of Psychology, Center for Brain Science Harvard University Cambridge Massachusetts USA; ^36^ Department of Psychiatry Massachusetts General Hospital Boston Massachusetts USA; ^37^ Tri‐Institutional Center for Translational Research in Neuroimaging and Data Science (TReNDS), Georgia State University, Georgia Institute of Technology, Emory University, USA Neurology, Radiology, Psychiatry and Biomedical Engineering Emory University Atlanta Georgia USA; ^38^ MRC Centre for Neuropsychiatric Genetics and Genomics Cardiff University Cardiff UK; ^39^ Department of Child and Adolescent Psychiatry New York University New York New York USA; ^40^ Stockholm Health Care Services Stockholm Region Stockholm Sweden; ^41^ Imaging Genetics Center, Mark and Mary Stevens Neuroimaging and Informatics Institute, Keck School of Medicine University of Southern California Los Angeles California USA; ^42^ Department of Neuroscience KU Leuven, Mind‐Body Research Group Leuven Belgium; ^43^ Department of Psychology University of New Mexico Albuquerque New Mexico USA; ^44^ Mind Research Network Albuquerque New Mexico USA; ^45^ Department of Psychiatry Université de Montréal Montreal Canada; ^46^ Department of Child and Adolescent Psychiatry, Psychosomatics and Psychotherapy University of Tübingen Tubingen Germany; ^47^ HU Virgen del Rocio, IBiS, University of Sevilla Sevilla Spain; ^48^ Groupe d'Imagerie Neurofonctionnelle, Institut des Maladies Neurodégénératives, UMR5293 Université de Bordeaux Talence France; ^49^ Erasmus School of Social and Behavioural Sciences Erasmus University Rotterdam Rotterdam Netherlands; ^50^ Faculteit der Sociale Wetenschappen, Instituut Psychologie Universiteit Leiden Leiden Netherlands; ^51^ Department of Psychiatry and Psychotherapy University of Münster Munster Germany; ^52^ Center for Multimodal Imaging and Genetics, Department of Neuroscience and Department of Radiology University of California‐San Diego La Jolla California USA; ^53^ Department of Psychiatry University of Melbourne Melbourne Australia; ^54^ Academisch Medisch Centrum Universiteit van Amsterdam Amsterdam Netherlands; ^55^ Faculty of Health, Institute of Health and Biomedical Innovation Queensland University of Technology Brisbane Australia; ^56^ Kimel Family Translational Imaging Genetics Laboratory Campbell Family Mental Health Research Institute, CAMH Toronto Canada; ^57^ Department of Psychiatry University of Toronto Toronto Canada; ^58^ Biological Psychiatry Lab, Fondazione IRCCS Casa Sollievo della Sofferenza San Giovanni Rotondo (FG) Italy; ^59^ Department of Psychology University of Oslo Oslo Norway; ^60^ Sunnaas Rehabilitation Hospital HT Nesodden Norway; ^61^ Division of Psychological and Social Medicine and Developmental Neurosciences Technische Universität Dresden Dresden Germany; ^62^ Faculty of Medicine Universitätsklinikum Carl Gustav Carus an der TU Dresden Dresden Germany; ^63^ Division of Mind and Brain Research, Department of Psychiatry and Psychotherapy Charité‐Universitätsmedizin Berlin Berlin Germany; ^64^ Bjørknes College Oslo Norway; ^65^ Language and Genetics Department Max Planck Institute for Psycholinguistics Nijmegen Netherlands; ^66^ Department of Psychiatry and Mental Health University of Cape Town Rondebosch South Africa; ^67^ Department of Human Genetics Radboud University Medical Center Nijmegen Netherlands; ^68^ Department of Psychiatry Radboud University Medical Center Nijmegen Netherlands; ^69^ Department of Psychiatry and Psychotherapy Otto von Guericke University Magdeburg Magdeburg Germany; ^70^ Department of Psychiatry, Tommy Fuss Center for Neuropsychiatric Disease Research Boston Children's Hospital Harvard Medical School Boston Massachusetts USA; ^71^ Department of Psychology Stanford University Stanford California USA; ^72^ Department of Psychiatry and Psychotherapy University Medicine Greifswald, University of Greifswald Greifswald Germany; ^73^ German Center for Neurodegenerative Diseases (DZNE) Site Rostock/Greifswald Greifswald Germany; ^74^ Department for Psychiatry, Psychosomatics and Psychotherapy, Universitätsklinikum Frankfurt Goethe Universitat Frankfurt Germany; ^75^ Neuroscience Institute University of Cape Town Rondebosch South Africa; ^76^ Section for Experimental Psychopathology and Neuroimaging, Department of General Psychiatry Heidelberg University Heidelberg Germany; ^77^ Department of Psychiatry Yale University New Haven Connecticut USA; ^78^ Learning Based Recovery Center VA Connecticut Health System New Haven Connecticut USA; ^79^ Lifespan Brain Institute, Perelman School of Medicine University of Pennsylvania Philadelphia Pennsylvania USA; ^80^ Children's Hospital of Philadelphia University of Pennsylvania Philadelphia Pennsylvania USA; ^81^ Melbourne Neuropsychiatry Center University of Melbourne Melbourne Australia; ^82^ Interdisciplinary Center Psychopathology and Emotion regulation University Medical Center Groningen, University of Groningen Groningen Netherlands; ^83^ Brain and Mind Centre University of Sydney Sydney Australia; ^84^ Departments of Experimental and Clinical Psychology Vrije Universiteit Amsterdam Amsterdam Netherlands; ^85^ Personalized Healthcare Genentech, Inc South San Francisco California USA; ^86^ Department of Psychiatry University Medical Center Groningen, University of Groningen Groningen Netherlands; ^87^ Department of Psychology Yale University New Haven Connecticut USA; ^88^ Norbert Institute of Diagnostic Radiology and Neuroradiology University Medicine Greifswald, University of Greifswald Greifswald Germany; ^89^ Bascule, Academic Centre for Children and Adolescent Psychiatry Duivendrecht Netherlands; ^90^ Department of Psychiatry Oxford University Oxford UK; ^91^ Center for Human Development, Departments of Cognitive Science, Psychiatry, and Radiology University of California San Diego California USA; ^92^ Department of Radiology Ohio State University College of Medicine Columbus Ohio USA; ^93^ Department of Neuroinformatics Araya, Inc Tokyo Japan; ^94^ Department of Psychiatry University of California San Diego La Jolla California USA; ^95^ Mental Health Research Center Russian Academy of Medical Sciences Moskva Russia; ^96^ Sunshine Coast Mind and Neuroscience, Thompson Institute University of the Sunshine Coast Sunshine Coast Australia; ^97^ Department of Child and Adolescent Psychiatry and Psychology Hospital Clinic, University of Barcelona Barcelona Spain; ^98^ Department of Psychiatry, Psychosomatics and Psychotherapy Julius‐Maximilians Universität Würzburg Wurzburg Germany; ^99^ SA MRC Unit on Risk and Resilience in Mental Disorders, Department of Psychiatry Stellenbosch University Stellenbosch South Africa; ^100^ Queensland Institute of Medical Research Berghofer Medical Research Institute Brisbane Australia; ^101^ Department of Psychiatry Bellvitge University Hospital‐IDIBELL, University of Barcelona Barcelona Spain; ^102^ Division of Psychiatry University of Edinburgh Edinburgh UK; ^103^ School of Clinical Sciences, Institute of Health and Biomedical Innovation Queensland University of Technology Brisbane Australia; ^104^ Department of Psychiatry and Psychotherapy, Central Institute of Mental Health Heidelberg University Heidelberg Germany; ^105^ Department of Clinical Medicine Kyushu University Kyushu Japan; ^106^ CatoSenteret Rehabilitation Hospital Son Norway; ^107^ Department of Radiation Sciences, Umeå Center for Functional Brain Imaging Umeå University Umeå Sweden; ^108^ Department of Clinical Neuropsychology Amsterdam University Medical Centre, Vrije Universiteit Amsterdam Amsterdam Netherlands; ^109^ Department of Psychiatry, University Hospital “Marques de Valdecilla” Instituto de Investigación Valdecilla (IDIVAL) Santander Spain; ^110^ Instituto de Salud Carlos III Madrid Spain; ^111^ Centre of Mental Health University of Würzburg Wurzburg Germany; ^112^ Department of Psychiatry, Hospital de la Santa Creu i Sant Pau, Institut d'Investigació Biomèdica Sant Pau Universitat Autònoma de Barcelona Barcelona Spain; ^113^ Department of Psychiatry University of California at Irvine Irvine California USA; ^114^ Department of Psychosis Studies, Institute of Psychiatry Psychology & Neuroscience, King's College London London UK; ^115^ Center for Depression, Anxiety, and Stress Research McLean Hospital, Harvard Medical School Boston Massachusetts USA; ^116^ Centro de Investigacion Biomedica en Red en Enfermedades Neurodegenerativas (CIBERNED) Madrid Spain; ^117^ Orygen, The National Centre of Excellence in Youth Mental Health Parkville Australia; ^118^ Centre for Youth Mental Health The University of Melbourne Melbourne Australia; ^119^ Department of Psychiatry and Psychotherapy University Medical Center Göttingen Göttingen Germany; ^120^ Centre for Population Neuroscience and Precision Medicine, Institute of Psychiatry Psychology & Neuroscience, King's College London London UK; ^121^ Institute of Mental Health Singapore Singapore; ^122^ Center for Genomic Medicine Massachusetts General Hospital Boston Massachusetts USA; ^123^ Department of Biomedical Sciences of Cells and Systems, Rijksuniversiteit Groningen University Medical Center Groningen Göttingen Netherlands; ^124^ Queensland Brain Institute University of Queensland Brisbane Australia; ^125^ PROMENTA Research Center, Department of Psychology University of Oslo Oslo Norway; ^126^ Neuroimaging Unit, Technological Facilities Valdecilla Biomedical Research Institute IDIVAL Santander Spain; ^127^ College of Arts and Sciences Georgia State University Atlanta Georgia USA; ^128^ School of Mental Health and Neuroscience, Faculty of Health, Medicine and Life Sciences Maastricht University Maastricht Netherlands; ^129^ Department of Psychiatry Leiden University Medical Center Leiden Netherlands; ^130^ Leiden Institute for Brain and Cognition Leiden Netherlands; ^131^ Department of Child and Adolescent Psychiatry/Psychology Erasmus University Medical Center, Sophia Children's Hospital Rotterdam The Netherlands; ^132^ Center for the Neurobiology of Learning and Memory University of California Irvine Irvine California USA; ^133^ Institute of Community Medicine University Medicine, Greifswald, University of Greifswald Greifswald Germany; ^134^ German Centre for Cardiovascular Research (DZHK), partner site Greifswald Greifswald Germany; ^135^ German Center for Diabetes Research (DZD), partner site Greifswald Greifswald Germany; ^136^ Department of Psychology University of Bath Bath UK; ^137^ Department of Psychiatry and Behavioral Sciences, Feinberg School of Medicine Northwestern University Chicago Illinois USA; ^138^ Department of Radiology Medical College of Wisconsin Milwaukee Wisconsin USA; ^139^ Institute for Experimental Epileptology and Cognition Research University of Bonn Bonn Germany; ^140^ Developmental and Educational Psychology Unit Institute of Psychology, Leiden University Leiden Netherlands; ^141^ National High Magnetic Field Laboratory Florida State University Tallahassee Florida USA; ^142^ Department of Child and Adolescent Psychiatry Child Study Center, NYU Langone Health New York New York USA; ^143^ Instituto de Ensino e Pesquisa, Hospital Sírio‐Libanês São Paulo Brazil; ^144^ Division of Molecular Psychiatry, Center of Mental Health University of Würzburg Wurzburg Germany; ^145^ Department of Psychiatry, Djavad Mowafaghian Centre for Brain Health University of British Columbia Vancouver Canada

**Keywords:** brain morphometry, ENIGMA, longitudinal trajectories, multisite

## Abstract

Age has a major effect on brain volume. However, the normative studies available are constrained by small sample sizes, restricted age coverage and significant methodological variability. These limitations introduce inconsistencies and may obscure or distort the lifespan trajectories of brain morphometry. In response, we capitalized on the resources of the Enhancing Neuroimaging Genetics through Meta‐Analysis (ENIGMA) Consortium to examine age‐related trajectories inferred from cross‐sectional measures of the ventricles, the basal ganglia (caudate, putamen, pallidum, and nucleus accumbens), the thalamus, hippocampus and amygdala using magnetic resonance imaging data obtained from 18,605 individuals aged 3–90 years. All subcortical structure volumes were at their maximum value early in life. The volume of the basal ganglia showed a monotonic negative association with age thereafter; there was no significant association between age and the volumes of the thalamus, amygdala and the hippocampus (with some degree of decline in thalamus) until the sixth decade of life after which they also showed a steep negative association with age. The lateral ventricles showed continuous enlargement throughout the lifespan. Age was positively associated with inter‐individual variability in the hippocampus and amygdala and the lateral ventricles. These results were robust to potential confounders and could be used to examine the functional significance of deviations from typical age‐related morphometric patterns.

## Graphical abstract

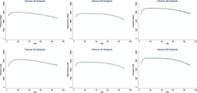

## 1 INTRODUCTION

Over the last 20 years, studies using structural magnetic resonance imaging (MRI) have confirmed that brain morphometric measures change with age. In general, whole brain, global and regional gray matter volumes increase during development and decrease with aging (Brain Development Cooperative Group, 2012; Driscoll et al., 2009; Fotenos, Snyder, Girton, Morris, & Buckner, 2005; Good et al., 2001; Pfefferbaum et al., 2013; Pomponio et al., 2019; Raz et al., 2005; Raznahan et al., 2014; Resnick, Pham, Kraut, Zonderman, & Davatzikos, 2003; Walhovd et al., 2011). However, most published studies are constrained by small sample sizes, restricted age coverage and methodological variability. These limitations introduce inconsistencies and may obscure or distort the lifespan trajectories of brain structures. To address these limitations, we formed the Lifespan Working group of the Enhancing Neuroimaging Genetics through Meta‐Analysis (ENIGMA) Consortium (Thompson et al., 2014, 2017) to perform large‐scale analyses of brain morphometric data extracted from MRI images using standardized protocols and unified quality control procedures, harmonized and validated across all participating sites.

Here we focus on ventricular, striatal (caudate, putamen, nucleus accumbens), pallidal, thalamic, hippocampal and amygdala volumes. Subcortical structures are crucial for normal cognitive and emotional adaptation (Grossberg, 2009). The striatum and pallidum (together referred to as basal ganglia) are best known for their role in action selection and movement coordination (Calabresi, Picconi, Tozzi, Ghiglieri, & Di Filippo, 2014) but they are also involved in other aspects of cognition particularly memory, inhibitory control, reward and salience processing (Chudasama & Robbins, 2006; Richard, Castro, Difeliceantonio, Robinson, & Berridge, 2013; Scimeca & Badre, 2012; Tremblay, Worbe, Thobois, Sgambato‐Faure, & Féger, 2015). The role of the hippocampus has been most clearly defined in connection to declarative memory (Eichenbaum, 2004; Shohamy & Turk‐Browne, 2013) while the amygdala has been historically linked to affect processing (Kober et al., 2008). The thalamus is centrally located in the brain and acts as a key hub for the integration of motor and sensory information with higher‐order functions (Sherman, 2005; Zhang, Snyder, Shimony, Fox, & Raichle, 2010). The role of subcortical structures extends beyond normal cognition because changes in the volume of these regions have been reliably identified in developmental (Ecker, Bookheimer, & Murphy, 2015; Krain & Castellanos, 2006), psychiatric (Hibar et al., 2016; Kempton et al., 2011; Schmaal et al., 2016; van Erp et al., 2016) and degenerative disorders (Risacher et al., 2009).

Using data from 18,605 individuals aged 3–90 years from the ENIGMA Lifespan working group we delineated the association between age and subcortical volumes from early to late life in order to (a) identify periods of volume change or stability, (b) provide normative, age‐adjusted centile curves of subcortical volumes and (c) quantify inter‐individual variability in subcortical volumes which is considered a major source of inter‐study differences (Dickie et al., 2013; Raz, Ghisletta, Rodrigue, Kennedy, & Lindenberger, 2010).

## 2 MATERIALS AND METHODS

### 2.1 Study samples

**FIGURE 1:**
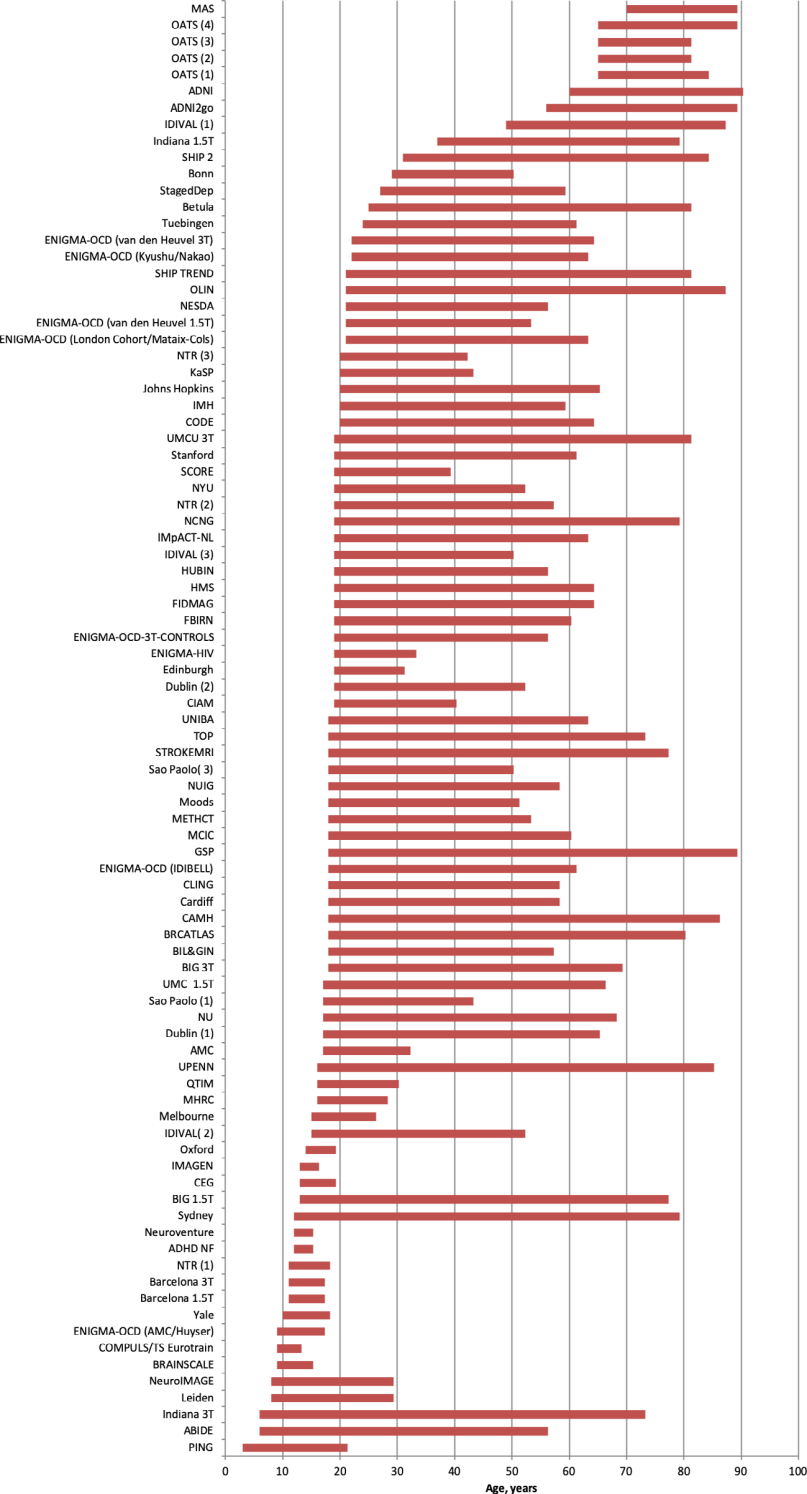
ENIGMA lifespan samples. Details of each sample are provided Table 1 and in the supplemental material. Abbreviations are provided in Table 1

The study data derive from 88 samples comprising 18,605 healthy participants, aged 3–90 years, with near equal representation of men and women (48% and 52%) (Table 1, Figure 1). At the time of scanning, participating individuals were screened to exclude the presence of mental disorders, cognitive impairment or significant medical morbidity. Details of the screening process and eligibility criteria for each research group are shown in Table S1).

**TABLE 1:** Characteristics of the included samples

Sample	Age, mean, years	Age, *SD*, years	Age range		Sample size *N*	Number of males	Number of females
ABIDE	17	7.8	6	56	534	439	95
ADHD NF	13	1	12	15	13	7	6
ADNI	76	5.1	60	90	150	70	80
ADNI2GO	73	6.1	56	89	133	55	78
AMC	23	3.4	17	32	92	60	32
Barcelona 1.5T	15	1.8	11	17	30	14	16
Barcelona 3T	15	2.1	11	17	44	24	20
Betula	61	12.9	25	81	234	104	130
BIG 1.5T	28	13.3	13	77	1,288	628	660
BIG 3T	24	7.9	18	69	1,276	540	736
BIL&GIN	27	7.8	18	57	444	217	227
Bonn	39	6.5	29	50	174	174	0
BRAINSCALE	10	1.4	9	15	270	125	145
BRCATLAS	38	15.8	18	80	153	77	76
CAMH	41	17.6	18	86	128	65	63
Cardiff	25	7.4	18	58	316	87	229
CEG	16	1.7	13	19	32	32	0
CIAM	27	5	19	40	30	16	14
CLING	25	5.3	18	58	320	131	189
CODE	40	13.3	20	64	74	31	43
COMPULS/TS Eurotrain	11	1	9	13	53	36	17
Dublin (1)	37	13	17	65	52	23	29
Dublin (2)	30	8.3	19	52	92	51	41
Edinburgh	24	2.9	19	31	55	35	20
ENIGMA‐HIV	25	4.4	19	33	31	16	15
ENIGMA‐OCD (AMC/Huyser)	14	2.6	9	17	23	9	14
ENIGMA‐OCD (IDIBELL)	33	10.1	18	61	65	29	36
ENIGMA‐OCD (Kyushu/Nakao)	39	12.5	22	63	40	15	25
ENIGMA‐OCD (London Cohort/Mataix‐Cols)	37	11.2	21	63	32	11	21
ENIGMA‐OCD (van den Heuvel 1.5T)	31	7.6	21	53	48	18	30
ENIGMA‐OCD (van den Heuvel 3T)	39	11.2	22	64	35	16	19
ENIGMA‐OCD‐3T‐CONTROLS	31	10.6	19	56	27	10	17
FBIRN	37	11.2	19	60	173	123	50
FIDMAG	38	10.2	19	64	122	53	69
GSP	26	14.9	18	89	1962	860	1,102
HMS	40	12.2	19	64	55	21	34
HUBIN	42	8.9	19	56	99	66	33
IDIVAL (1)	65	10.2	49	87	31	10	21
IDIVAL (3)	30	7.7	19	50	114	69	45
IDIVAL(2)	28	7.6	15	52	79	49	30
IMAGEN	14	0.4	13	16	1744	864	880
IMH	32	10	20	59	79	50	29
IMpACT‐NL	37	12	19	63	134	52	82
Indiana 1.5T	60	11	37	79	41	7	34
Indiana 3T	27	18.8	6	73	197	95	102
Johns Hopkins	44	12.5	20	65	87	41	46
KaSP	27	5.7	20	43	32	15	17
Leiden	17	4.8	8	29	565	274	291
MAS	78	4.5	70	89	361	137	224
MCIC	33	12	18	60	93	63	30
Melbourne	20	3	15	26	102	54	48
METHCT	27	7.3	18	53	62	48	14
MHRC	22	2.9	16	28	52	52	0
Moods	33	9.8	18	51	310	146	164
NCNG	50	16.7	19	79	311	92	219
NESDA	40	9.8	21	56	65	22	43
NeuroIMAGE	17	3.7	8	29	376	172	204
Neuroventure	14	0.6	12	15	137	62	75
NTR (1)	15	1.4	11	18	34	11	23
NTR (2)	34	10.3	19	57	105	39	66
NTR (3)	30	5.9	20	42	29	11	18
NU	41	18.8	17	68	15	1	14
NUIG	37	11.5	18	58	89	50	39
NYU	31	8.7	19	52	51	31	20
OATS (1)	71	5.3	65	84	94	27	67
OATS (2)	68	4.4	65	81	33	13	20
OATS (3)	69	4.3	65	81	128	44	84
OATS (4)	70	4.6	65	89	95	23	72
OLIN	36	12.8	21	87	594	236	358
Oxford	16	1.4	14	19	38	18	20
PING	12	4.9	3	21	518	271	247
QTIM	23	3.4	16	30	342	112	230
Sao Paolo 1	27	5.8	17	43	69	45	24
Sao Paolo 3	30	8.1	18	50	83	44	39
SCORE	25	4.3	19	39	44	17	27
SHIP 2	55	12.3	31	84	368	206	162
SHIP TREND	50	13.9	21	81	788	439	349
StagedDep	47	8	27	59	84	20	64
Stanford	37	10.7	19	61	54	20	34
STROKEMRI	42	21.3	18	77	47	17	30
Sydney	37	21.1	12	79	147	58	89
TOP	35	9.8	18	73	296	155	141
Tuebingen	40	12.1	24	61	53	24	29
UMC Utrecht 1.5T	32	12.1	17	66	289	171	118
UMCU 3T	45	15.2	19	81	109	52	57
UNIBA	27	8.7	18	63	130	66	64
UPENN	36	13.6	16	85	185	85	100
Yale	14	2.2	10	18	23	12	11
Total	31	18.4	3	90	18,605	8,980	9,625

Abbreviations: ABIDE = Autism Brain Imaging Data Exchange; ADNI = Alzheimer's Disease Neuroimaging Initiative; ADNI2GO = ADNI‐GO and ADNI‐2; ADHD‐NF = Attention Deficit Hyperactivity Disorder‐Neurofeedback Study; AMC = Amsterdam Medisch Centrum; Basel = University of Basel; Barcelona = University of Barcelona; Betula = Swedish longitudinal study on aging, memory, and dementia; BIG = Brain Imaging Genetics; BIL&GIN = a multimodal multidimensional database for investigating hemispheric specialization; Bonn = University of Bonn; BrainSCALE = Brain Structure and Cognition: an Adolescence Longitudinal twin study; CAMH = Centre for Addiction and Mental Health; Cardiff = Cardiff University; CEG = Cognitive‐experimental and Genetic study of ADHD and Control Sibling Pairs; CIAM = Cortical Inhibition and Attentional Modulation study; CLiNG = Clinical Neuroscience Göttingen; CODE = formerly Cognitive Behavioral Analysis System of Psychotherapy (CBASP) study; Dublin = Trinity College Dublin; Edinburgh = The University of Edinburgh; ENIGMA‐HIV = Enhancing NeuroImaging Genetics through Meta‐Analysis‐Human Immunodeficiency Virus Working Group; ENIGMA‐OCD = Enhancing NeuroImaging Genetics through Meta‐Analysis‐ Obsessive Compulsive Disorder Working Group; FBIRN = Function Biomedical Informatics Research Network; FIDMAG = Fundación para la Investigación y Docencia Maria Angustias Giménez; GSP = Brain Genomics Superstruct Project; HMS = Homburg Multidiagnosis Study; HUBIN = Human Brain Informatics; IDIVAL = Valdecilla Biomedical Research Institute; IMAGEN = the IMAGEN Consortium; IMH=Institute of Mental Health, Singapore; IMpACT = The International Multicentre persistent ADHD Genetics Collaboration; Indiana = Indiana University School of Medicine; Johns Hopkins = Johns Hopkins University; KaSP = The Karolinska Schizophrenia Project; Leiden = Leiden University; MAS = Memory and Ageing Study; MCIC = MIND Clinical Imaging Consortium formed by the Mental Illness and Neuroscience Discovery (MIND) Institute now the Mind Research Network; Melbourne = University of Melbourne; Meth‐CT = study of methamphetamine users, University of Cape Town; MHRC = Mental Health Research Center; Muenster = Muenster University; N = number; NESDA = The Netherlands Study of Depression and Anxiety; NeuroIMAGE = Dutch part of the International Multicenter ADHD Genetics (IMAGE) study; Neuroventure: the imaging part of the Co‐Venture Trial funded by the Canadian Institutes of Health Research (CIHR); NCNG = Norwegian Cognitive NeuroGenetics sample; NTR = Netherlands Twin Register; NU = Northwestern University; NUIG = National University of Ireland Galway; NYU = New York University; OATS = Older Australian Twins Study; Olin = Olin Neuropsychiatric Research Center; Oxford = Oxford University; QTIM = Queensland Twin Imaging; Sao Paulo = University of Sao Paulo; SCORE = University of Basel Study; SHIP‐2 and SHIP TREND = Study of Health in Pomerania; Staged‐Dep = Stages of Depression Study; Stanford = Stanford University; StrokeMRI = Stroke Magnetic Resonance Imaging; Sydney = University of Sydney; TOP = Tematisk Område Psykoser (Thematically Organized Psychosis Research); TS‐EUROTRAIN = European‐Wide Investigation and Training Network on the Etiology and Pathophysiology of Gilles de la Tourette Syndrome; Tuebingen = University of Tuebingen; UMCU = Universitair Medisch Centrum Utrecht; UNIBA = University of Bari Aldo Moro; UPENN = University of Pennsylvania; Yale = Yale University.

### 2.2 Neuroimaging

Detailed information on scanner vendor, magnet strength and acquisition parameters for each sample are presented in Table S1. For each sample, the intracranial volume (ICV) and the volume of the basal ganglia (caudate, putamen, pallidum, nucleus accumbens), thalamus, hippocampus, amygdala and lateral ventricles were extracted using FreeSurfer (http://surfer.nmr.mgh.harvard.edu) from high‐resolution T_1_‐weighted MRI brain scans (Fischl, 2012; Fischl et al., 2002). Prior to data pooling, images were visually inspected at each site to exclude participants whose scans were improperly segmented. After merging the samples, only individuals with complete data were included outliers were identified and excluded using Mahalanobis distances. All analyses described below were repeated for ICV‐unadjusted volumetric measures which yielded identical results and are only presented as a separate supplement.

Approximately 20% of the samples had a multi‐scanner design. During data harmonization the scanner was modeled as a site. In each site, the intracranial volume (Figure S1) was used to adjust the subcortical volumes via a formula based on the analysis of the covariance approach: “adjusted volume = raw volume – *b* × (ICV – mean ICV)”, where *b* is the slope of regression of a region of interest volume on ICV (Raz et al., 2005). The values of the subcortical volumes were then harmonized between sites using the ComBat method in R (Fortin et al., 2017, 2018; Radua et al., 2020). Originally developed to adjust for batch effect in genetic studies, ComBat uses an empirical Bayes to adjust for inter‐site variability in the data, while preserving variability related to the variables of interest.

### 2.3 Fractional polynomial regression analyses

The effect of age on each ICV‐ and site‐adjusted subcortical volume was modeled using high order fractional polynomial regression (Royston & Altman, 1994; Sauerbrei, Meier‐Hirmer, Benner, & Royston, 2006) in each hemisphere. Because the effect of site (scanner and Freesurfer version) was adjusted using ComBat, we only included sex as a covariate in the regression models. Fractional polynomial regression is currently considered the most advantageous modeling strategy for continuous variables (Moore, Hanley, Turgeon, & Lavoie, 2011) as it allows testing for a wider range of trajectory shapes than conventional lower‐order polynomials (e.g., linear or quadratic) and for multiple turning points (Royston & Altman, 1994; Royston, Ambler, & Sauerbrei, 1999). For each subcortical structure, the best model was obtained by comparing competing models of up to three power combinations. The powers used to identify the best fitting model were −2, −1, −0.5, 0.5, 1, 2, 3 and the natural logarithm (ln) function. The optimal model describing the association between age and each of the volumes was selected as the lowest degree model based on the partial *F*‐test (if linear) or the likelihood‐ratio test. To avoid over‐fitting at ages with more data points, we used the stricter .01 level of significance as the cut‐off for each respective likelihood‐ratio tests, rather than adding powers, until the .05 level was reached. For ease of interpretation we centered the volume of each structure so that the intercept of a fractional polynomial was represented as the effect at zero for sex. Fractional polynomial regression models were fitted using Stata/IC software v.13.1 (Stata Corp., College Station, TX). Standard errors were also adjusted for the effect of site in the FP regression.

We conducted two supplemental analyses: (a) we specified additional FP models separately for males and females and, (b) we calculated Pearson's correlation coefficient between subcortical volumes and age in the early (6–29 years), middle (30–59 years), and late‐life (60–90 years) age‐group. The results of these analyses have been included in the supplemental material.

### 2.4 Inter‐individual variability

Inter‐individual variability was assessed using two complimentary approaches. First, for each subcortical structure we compared the early (6–29 years), middle (30–59 years) and late‐life (60–90 years) age‐groups in terms of their mean inter‐individual variability; these groups were defined following conventional notions regarding periods of development, midlife and aging. The variance of each structure in each age‐group was calculated as

$$\ln\left(\frac{\sum \sqrt{e_{i}^{2}}}{n_{t}}\right)$$

where *e* represents the residual variance of each individual (*i*) around the nonlinear best fitting regression line, and *n* the number of observations in each age‐group (*t*). The residuals (*e*_*i*_) were normally distributed suggesting good fit of the model without having over‐ or under‐fitted the data. Upon calculating the square root of the squared residuals we used the natural logarithm to account for the positive skewness of the new distribution. Then the mean inter‐individual variability between early (6–29 years), middle (30–59 years) and late‐life (60–90 years) age‐groups was compared using between‐groups omnibus tests for the residual variance around the identified best‐fitting nonlinear fractional polynomial model of each structure. We conducted 16 tests (one for each structure) and accordingly the critical alpha value was set at 0.003 following Bonferroni correction for multiple comparisons.

The second approach entailed the quantification of the mean individual variability of each subcortical structure through a meta‐analysis of the *SD* of the adjusted volumes according to the method proposed by Senior, Gosby, Lu, Simpson, and Raubenheimer (2016).

### 2.5 Centile curves

Reference curves for each structure by sex and hemisphere were produced from ICV‐ and site‐adjusted volumes as normalized growth centiles using the parametric Lambda (*λ*), Mu (*μ*), Sigma (*σ*) (LMS) method (Cole & Green, 1992) implemented using the Generalized Additive Models for Location, Scale and Shape (GAMLSS) in R (http://cran.r-project.org/web/packages/gamlss/index.html) (Rigby & Stasinopoulos, 2005; Stasinopoulos & Rigby, 2007). LMS allows for the estimation of the distribution at each covariate value after a suitable transformation and is summarized using three smoothing parameters, the Box‐Cox power *λ*, the mean *μ* and the coefficient of variation *σ*. GAMLSS uses an iterative maximum (penalized) likelihood estimation method to estimate *λ*, *μ* and *σ* as well as distribution dependent smoothing parameters and provides optimal values for effective degrees of freedom (edf) for every parameter (Indrayan, 2014). This procedure minimizes the Generalized Akaike Information Criterion (GAIC) goodness of fit index; smaller GAIC values indicate better fit of the model to the data. GAMLSS is a flexible way to derive normalized centile curves as it allows each curve to have its own number of edf while overcoming biased estimates resulting from skewed data

## 3 RESULTS

### 3.1 Fractional polynomial regression analyses

**FIGURE 2:**
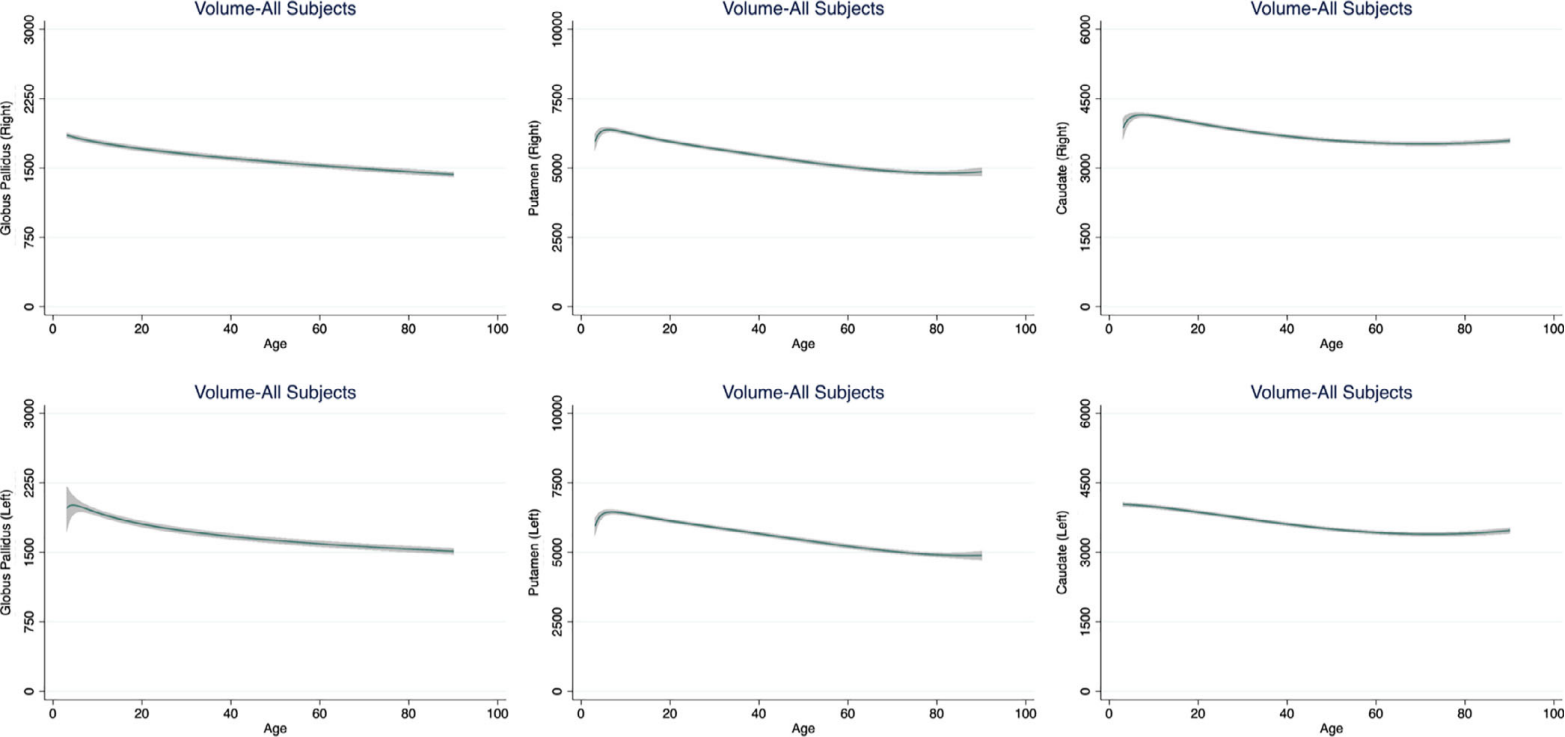
Fractional polynomial plots for the volume of the basal ganglia. Fractional Polynomial plots of adjusted volumes (mm^3^) against age (years) with a fitted regression line (solid line) and 95% confidence intervals (shaded area)

**FIGURE 3:**
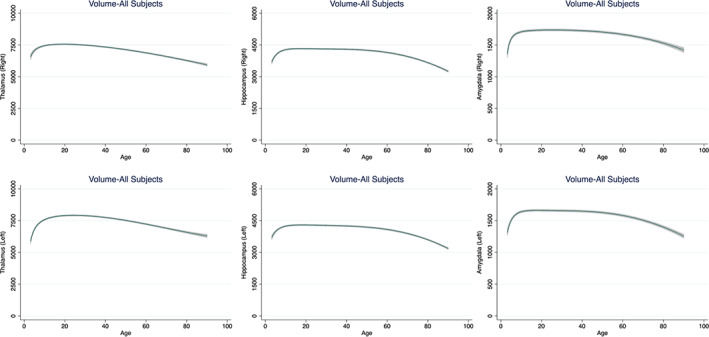
Fractional polynomial plots for the volume of the thalamus, hippocampus and amygdala. Fractional polynomial plots of adjusted volumes (mm^3^) against age (years) with a fitted regression line (solid line) and 95% confidence intervals (shaded area)

The volume of the caudate, putamen, globus pallidus and nucleus accumbens peaked early during the first decade of life and showed a linear decline immediately thereafter (Figure 2, Figures S2–S4). The association between age and the volumes of the thalamus, hippocampus and amygdala formed a flattened, inverted U‐curve (Figure 3, Figures S5 and S6). Specifically, the volumes of these structures were largest during the first 2–3 decades of life, remained largely stable until the sixth decade and declined gradually thereafter (Table S2). The volume of the lateral ventricles increased steadily with age bilaterally (Figure S7). The smallest proportion of variance explained by age and its FP derivatives was noted in the right amygdala (7%) and the largest in the lateral ventricles bilaterally (38%) (Table S2).

Striatal volumes correlated negatively with age throughout the lifespan with the largest coefficients observed in the middle‐life age‐group (*r* = −0.39 to −0.20) and the lowest (|*r*| < 0.05) in the late‐life age‐group, particularly in the caudate. The volumes of the thalamus, the hippocampus and the amygdala showed small positive correlations with age (*r* ≈ 0.16) in the early‐life age‐group. In the middle‐life age‐group, the correlation between age and subcortical volumes became negative (*r* = −0.30 to −0.27) for the thalamus but remained largely unchanged for the amygdala and the hippocampus. In the late‐life age‐group, the largest negative correlation coefficients between age and volume were observed for the hippocampus bilaterally (*r* = −0.44 to −0.39). The correlation between age and lateral ventricular volumes bilaterally increased throughout the lifespan from *r* = 0.19 to 0.20 in early‐life age‐group to *r* = 0.40 to 0.45 in the late‐life age‐group (Table S3). No effect of sex was noted for any pattern of correlation between subcortical volumes and age in any age‐group.

*Inter‐individual variability*: For each structure, the mean inter‐individual variability in volume in each age‐group is shown in Table S5. Inter‐individual variance was significantly higher for the hippocampus, thalamus amygdala and lateral ventricles bilaterally in the late‐life age‐group compared to both the early‐ and middle‐life group. These findings were recapitulated when data were analyzed using a meta‐analytic approach (Figure S8).

**FIGURE 4:**
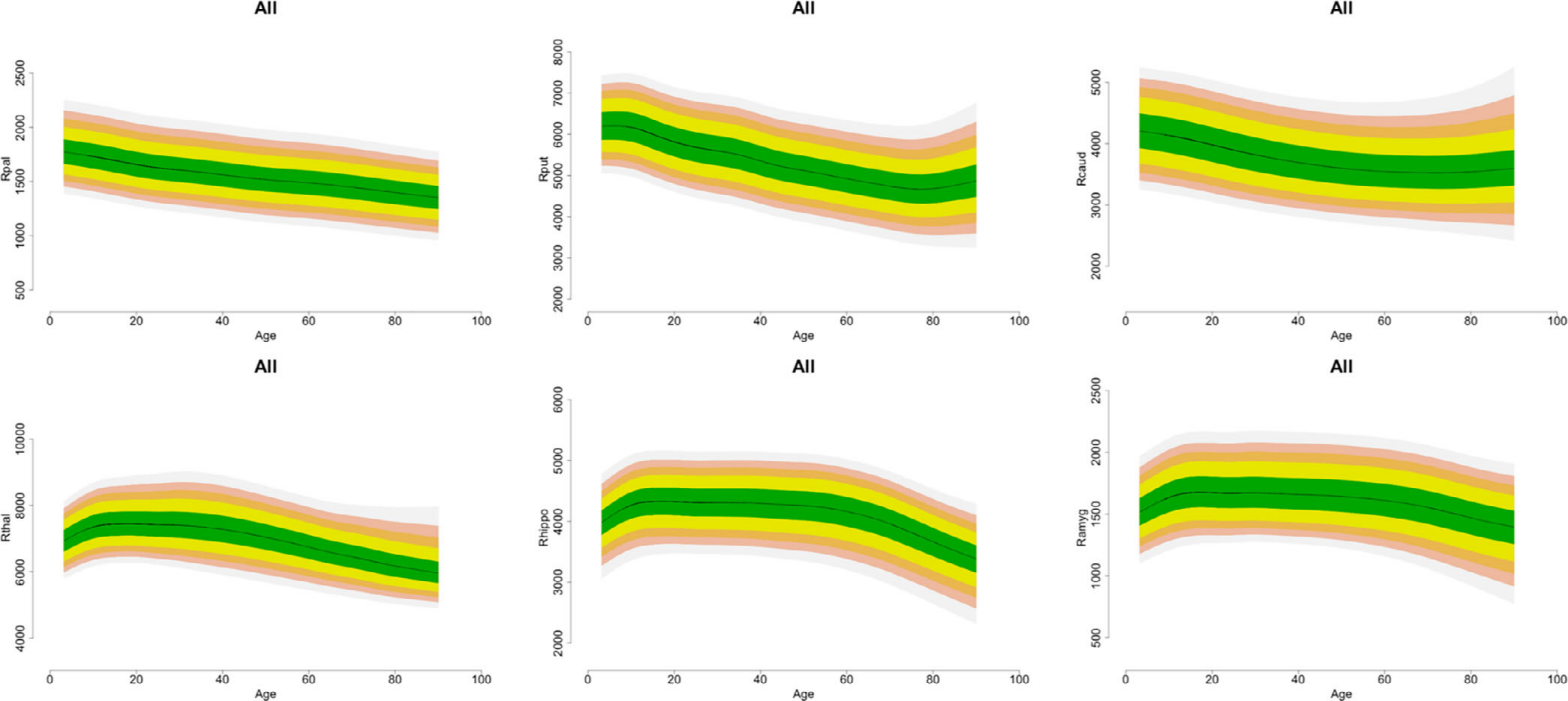
Centile values for subcortical volumes; Additional details in Tables S6‐S9

*Normative Centile Curves*: Centile normative values for each subcortical structure stratified by sex and hemisphere are shown in Figure 4 and Tables S6–S8.

## 4 DISCUSSION

We analyzed subcortical volumes from 18,605 healthy individuals from multiple cross‐sectional cohorts to infer age‐related trajectories between the ages of 3 and 90 years. Our lifespan perspective and our large sample size complement and enrich previous age‐related findings in subcortical volumes.

We found three distinct patterns of association between age and subcortical volumes. The volume of the lateral ventricles increased monotonically with age. Striatal and pallidal volumes peaked in childhood and declined thereafter. The volumes of the thalamus, hippocampuus and amygdala peaked later and showed a prolonged period of stability lasting until the sixth decade of life, before they also started to decline. These findings are in line with those of Pomponio et al. (2019), who also used harmonized multi‐site MRI data from 10,323 individuals aged 3–96 years, and those reported by Douaud et al. (2014) who analyzed volumetric data from 484 healthy participants aged 8 to 85 years. Notably, both studies reported similarity in the age‐related changes of the thalamus, hippocampus and the amygdala. Our results also underscore the significantly steeper negative association between subcortical volumes and age from the sixth decade of life onwards. This effect seemed relatively more pronounced for the hippocampus, compared to the other subcortical regions, as observed in other studies (Jernigan et al., 2001; Pomponio et al., 2019; Raz et al., 2010).

The trajectories of subcortical volumes are shaped by genetic and nongenetic exposures, biological or otherwise (Eyler et al., 2011; Somel et al., 2010; Wardlaw et al., 2011). Our findings of higher inter‐individual variability with age in the volumes of the thalamus, hippocampus and amygdala suggest that these structures may be more susceptible to person‐specific exposures, or late‐acting genes, particularly from the sixth decade onwards.

The unique strengths of this study are the availability of age‐overlapping cross‐sectional data from healthy individuals, lifespan coverage and the use of standardized protocols for volumetric data extraction across all samples. Study participants in each site were screened to ensure mental and physical wellbeing at the time of scanning using procedures considered as standard in designating study participants as healthy controls. Although health is not a permanent attribute, it is extremely unlikely given the size of the sample that the results could have been systematically biased by incipient disease

A similar longitudinal design would be near infeasible in terms of recruitment and retention both of participants and investigators. Although multisite studies have to account for differences in scanner type and acquisition, lengthy longitudinal designs encounter similar issues due to inevitable changes in scanner type and strength and acquisition parameters over time. In this study, the use of age‐overlapping samples from multiple different countries has the theoretical advantage of diminishing systematic biases reflecting cohort and period effects (Glenn, 2003; Keyes, Utz, Robinson, & Li, 2010) that are likely to operate in single site studies.

In medicine, biological measures from each individual are typically categorized as normal or otherwise in reference to a population derived normative range. This approach is yet to be applied to neuroimaging data, despite the widespread use of structural MRI for clinical purposes and the obvious benefit of a reference range from the early identification of deviance (Dickie et al., 2013; Pomponio et al., 2019). Alzheimer's disease provides an informative example as the degree of baseline reduction in medial temporal regions, and particularly the hippocampus, is one of the most significant predictors of conversion from mild cognitive impairment to Alzheimer's disease (Risacher et al., 2009). The data presented here demonstrate the power of international collaborations within ENIGMA for analyzing large‐scale datasets that could eventually lead to normative range for brain volumes for well‐defined reference populations. The centile curves presented here are a first‐step in developing normative reference values for neuroimaging phenotypes and further work is required in establishing measurement error and functional significance (see Supplement). These curves are not meant to be used clinically or to provide valid percentile measures for a single individual.

In conclusion, we used existing cross‐sectional data to infer age‐related trajectories of regional subcortical volumes. The size and age‐coverage of the analysis sample has the potential to disambiguate uncertainties regarding developmental and aging changes in subcortical volumes while the normative centile values could be further developed and evaluated.

## CONFLICT OF INTEREST

H.‐J. G.: Travel grants and speaker honoraria from Fresenius Medical Care, Neuraxpharm, Servier and Janssen Cilag; research funding from Fresenius Medical Care. O. A. A.: Consultant to HealthLytix, speaker honorarium from Lundbeck. A. M. D.: Founder and member of the Scientific Advisory Board CorTechs Labs, Inc where he holds equity; member of the Scientific Advisory of Human Longevity Inc; research grants with General Electric Healthcare.

## Supporting information


**Appendix S1:** Supplementary Information

## Data Availability

The ENIGMA Lifespan Working Group welcomes expression of interest from researchers in the field who wish to use the ENIGMA samples. Data sharing is possible subsequent to consent for the principal investigators of the contributing datasets. Requests should be directed to the corresponding authors.
