# Supplementary material for: Subcortical volumes across the lifespan: Data from 18,605 healthy individuals aged 3–90 years
Source: Hum Brain Mapp. 2021 Feb 11;43(1):452–69. doi: 10.1002/hbm.25320 (PMC8675429; doi:10.1002/hbm.25320)
Supplement: Supplementary file 1 — Appendix S1: Supplementary Information [file HBM-43-452-s001.zip › HBM_25320_ENIGMA_subcortical_Supplement R2.docx]

**Online Supplement**

**Supplementary Figures**

**Figure S1.** Intracranial volume (ICV) by sex and age (mean, standard error)

**Figure S2.** Age-related trajectories in globus pallidus, putamen, and caudate in males

**Figure S3.** Age-related trajectories in globus pallidus, putamen, and caudate in females

**Figure S4.** Age-related trajectories in nucleus accumbens

**Figure S5.** Age-related trajectories in thalamus, hippocampus, and amygdala in males

**Figure S6.** Age-related trajectories in thalamus, hippocampus, and amygdala in females

**Figure S7.** Age-related trajectories in lateral ventricles

**Figure S8.** Meta-analysis of the Pooled Standard Deviation of the Volume of each Subcortical Structure Stratified by Sex

**Supplementary Tables**

**Table S1.** Screening Process and Eligibility Criteria, Scanner, Image Acquisition Parameters and Image Segmentation Software

**Table S2.** Age at Maximum Fitted Value for Subcortical Volumes

**Table S3.** Variance Explained by Age in Fractional Polynomial Model

**Table S4:** Pearson's Correlation Coefficient between Age and Subcortical Volumes

**Table S5.** Inter-individual Variations in Subcortical Volumes

**Table S6.** Centile Values for Subcortical Volumes- All participants

**Table S7.** Centile Values for Subcortical Volumes in males

**Table S8.** Centile Values for Subcortical Volumes in females

**Table S9**. Preliminary analyses of measurement error in the whole sample centile curves

**Figure S1. Intracranial volume (ICV) by Sex and Age (mean, standard error)**

**
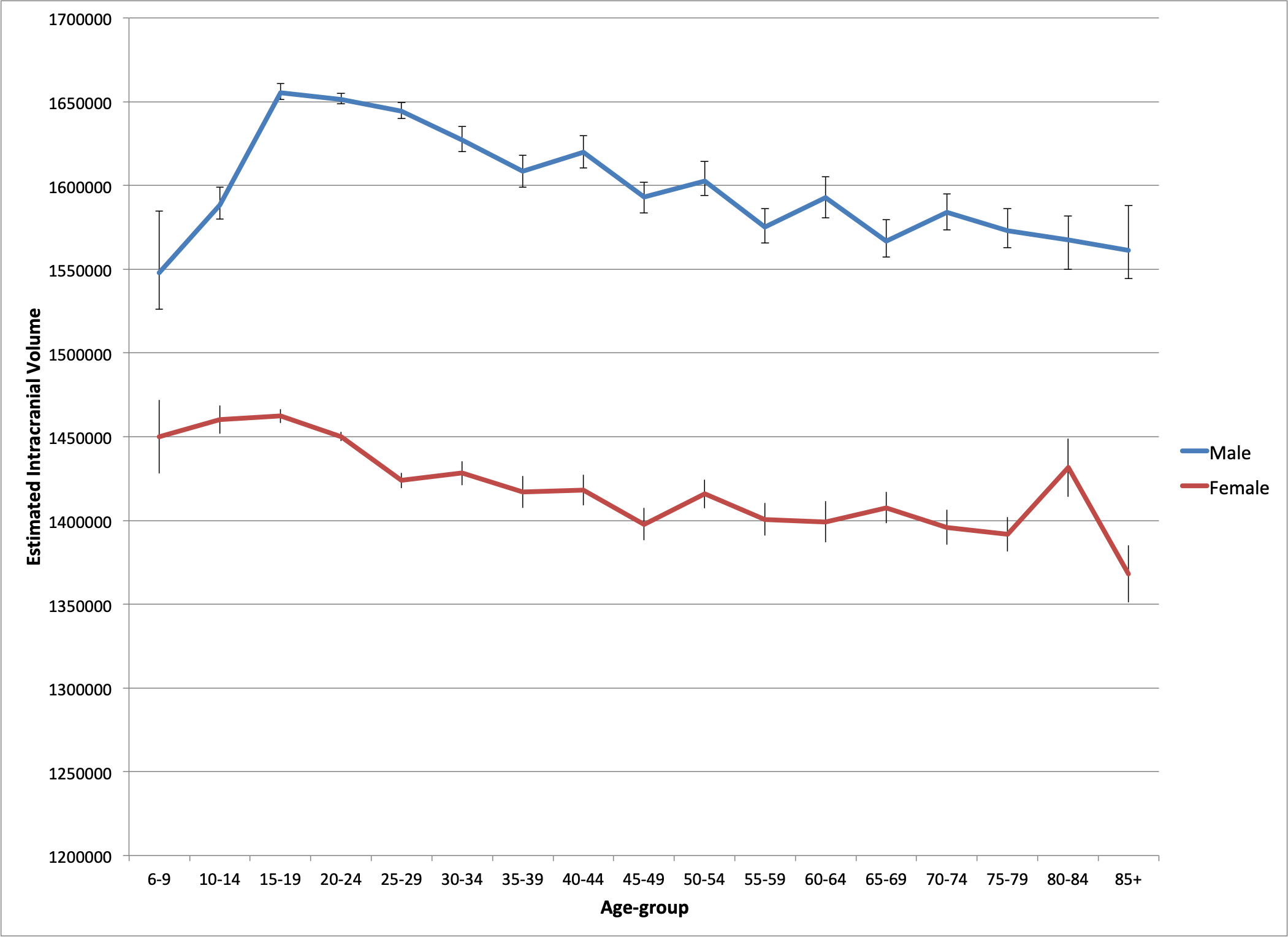
**

**Figure S2. Age-related trajectories in Globus Pallidus, Putamen and Caudate in Males**


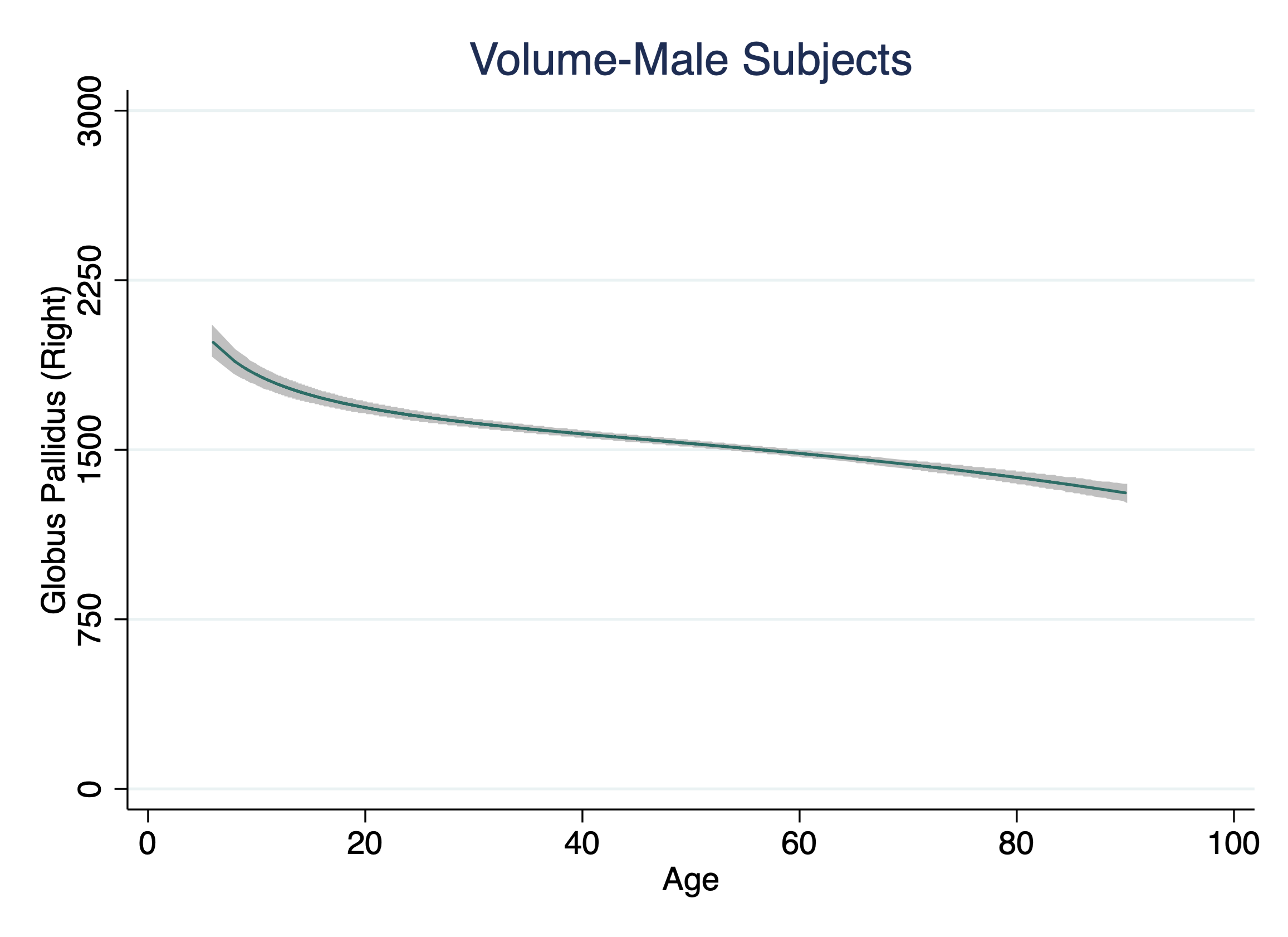

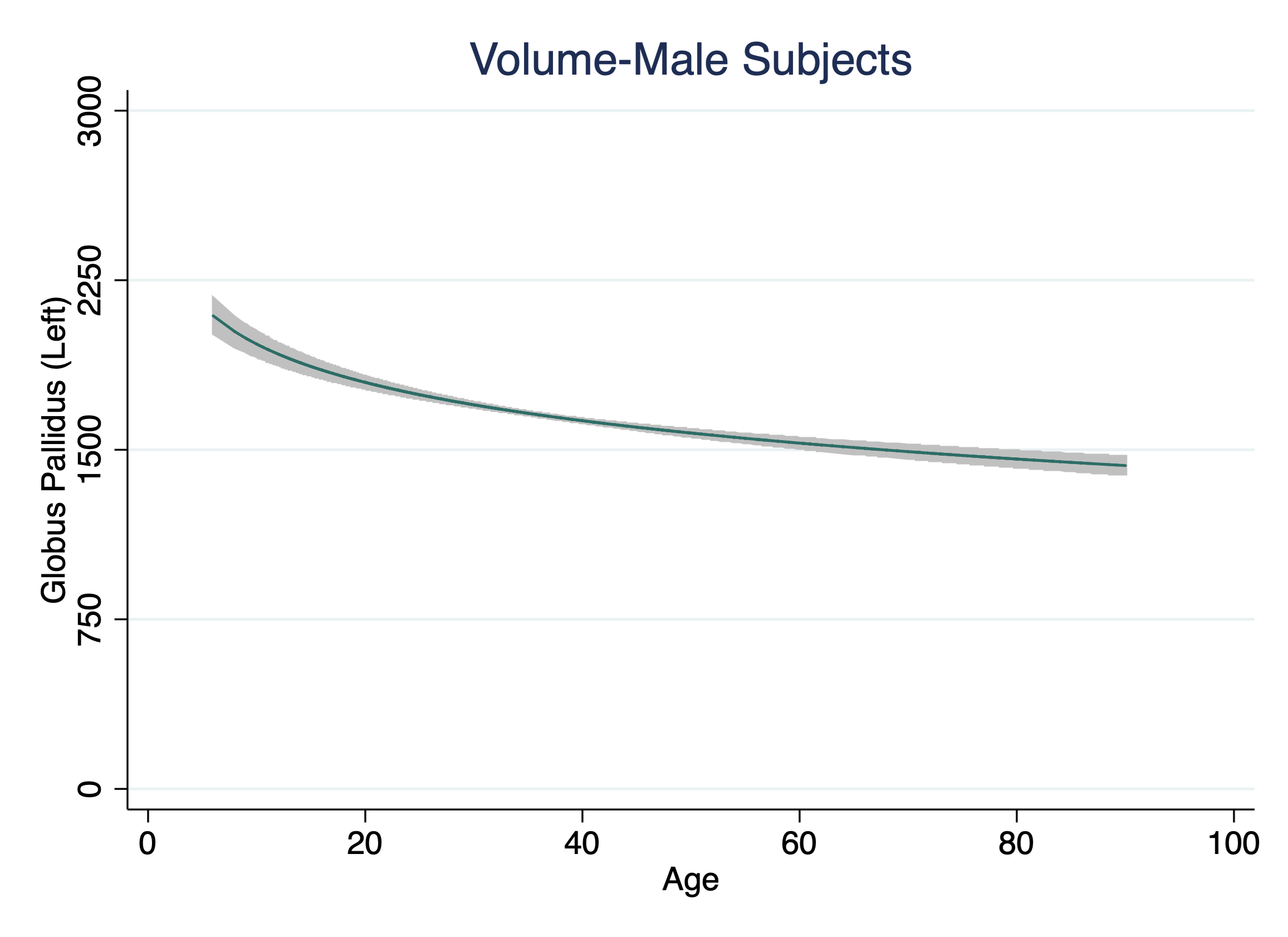

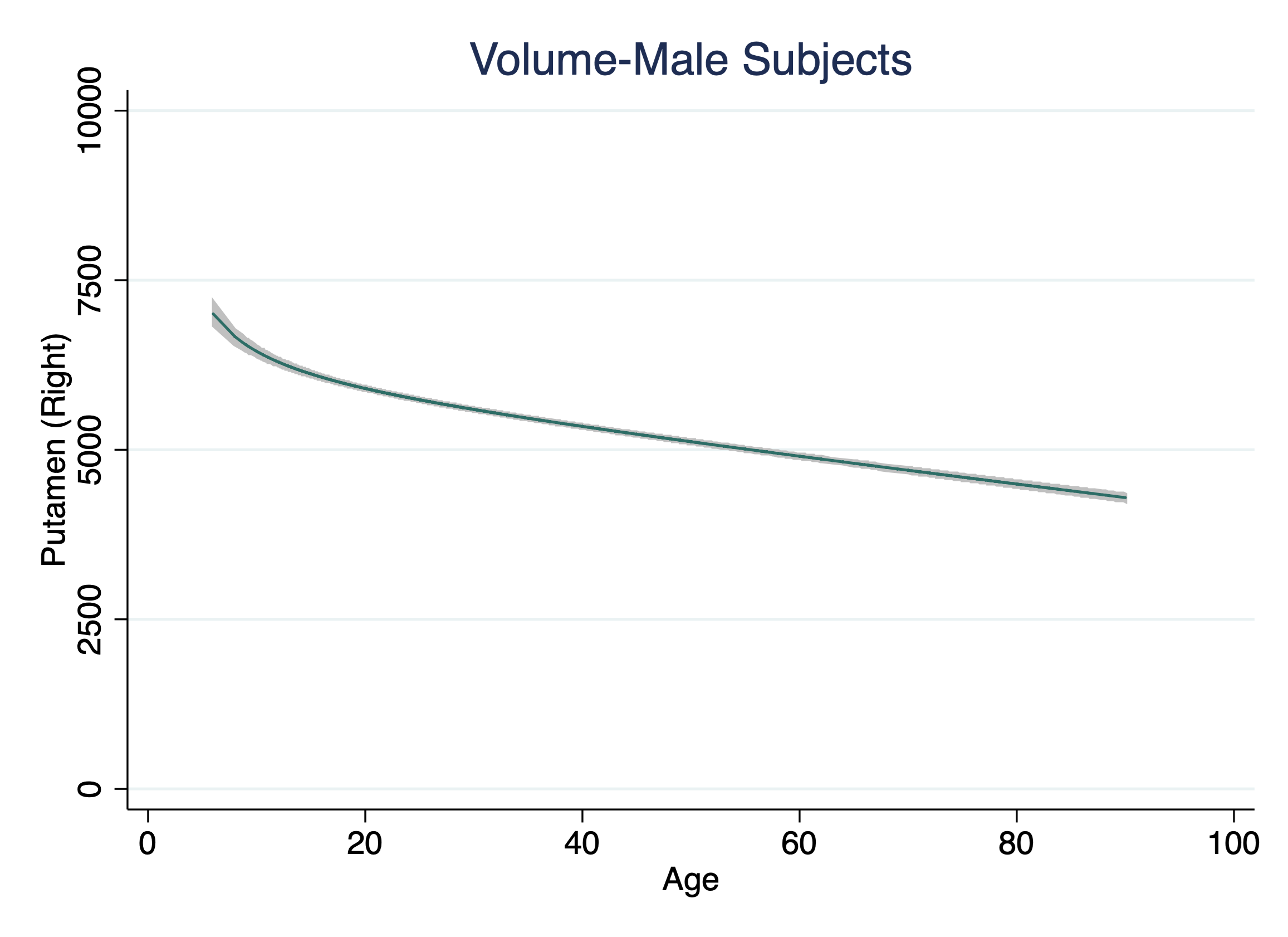

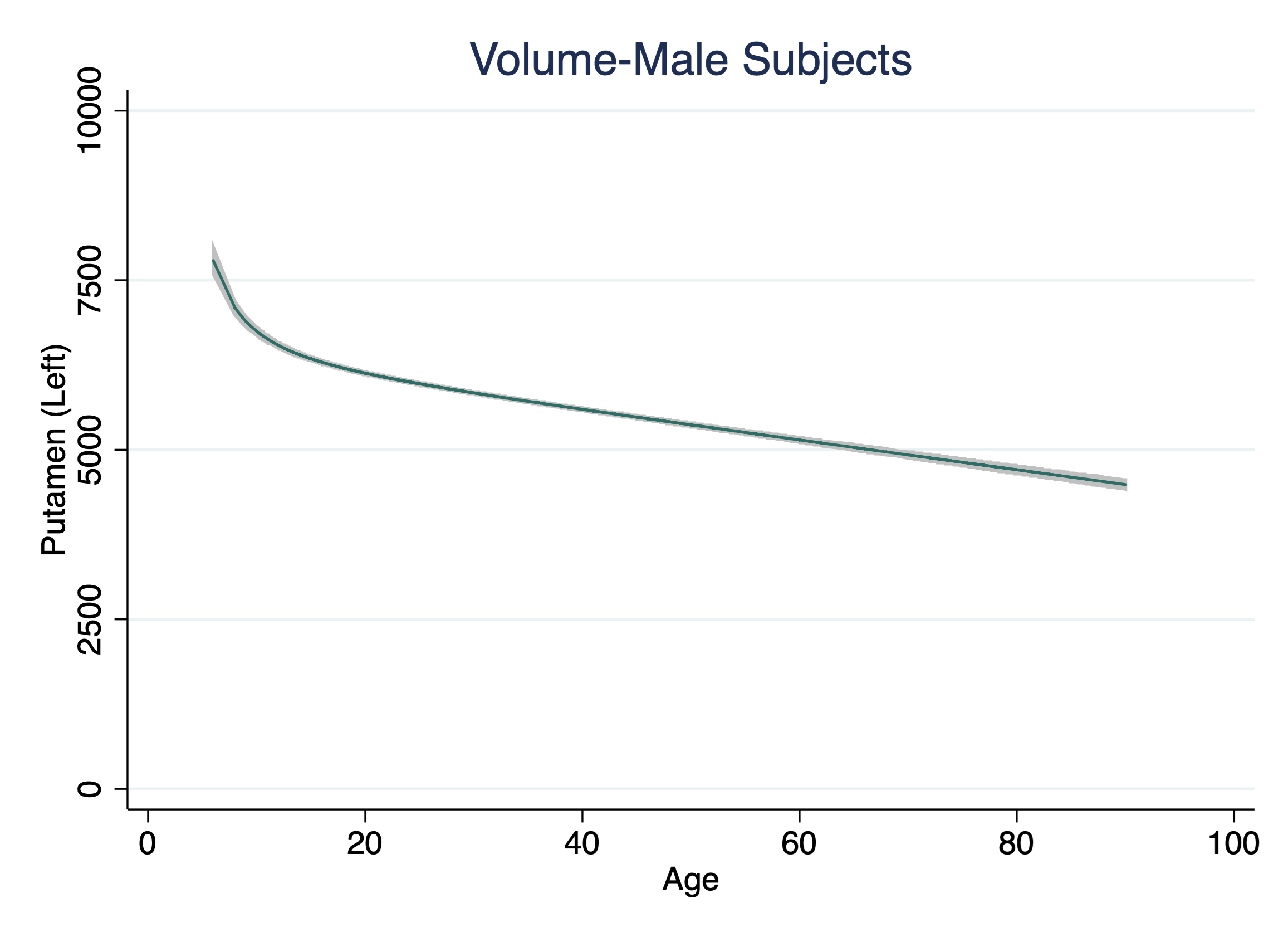

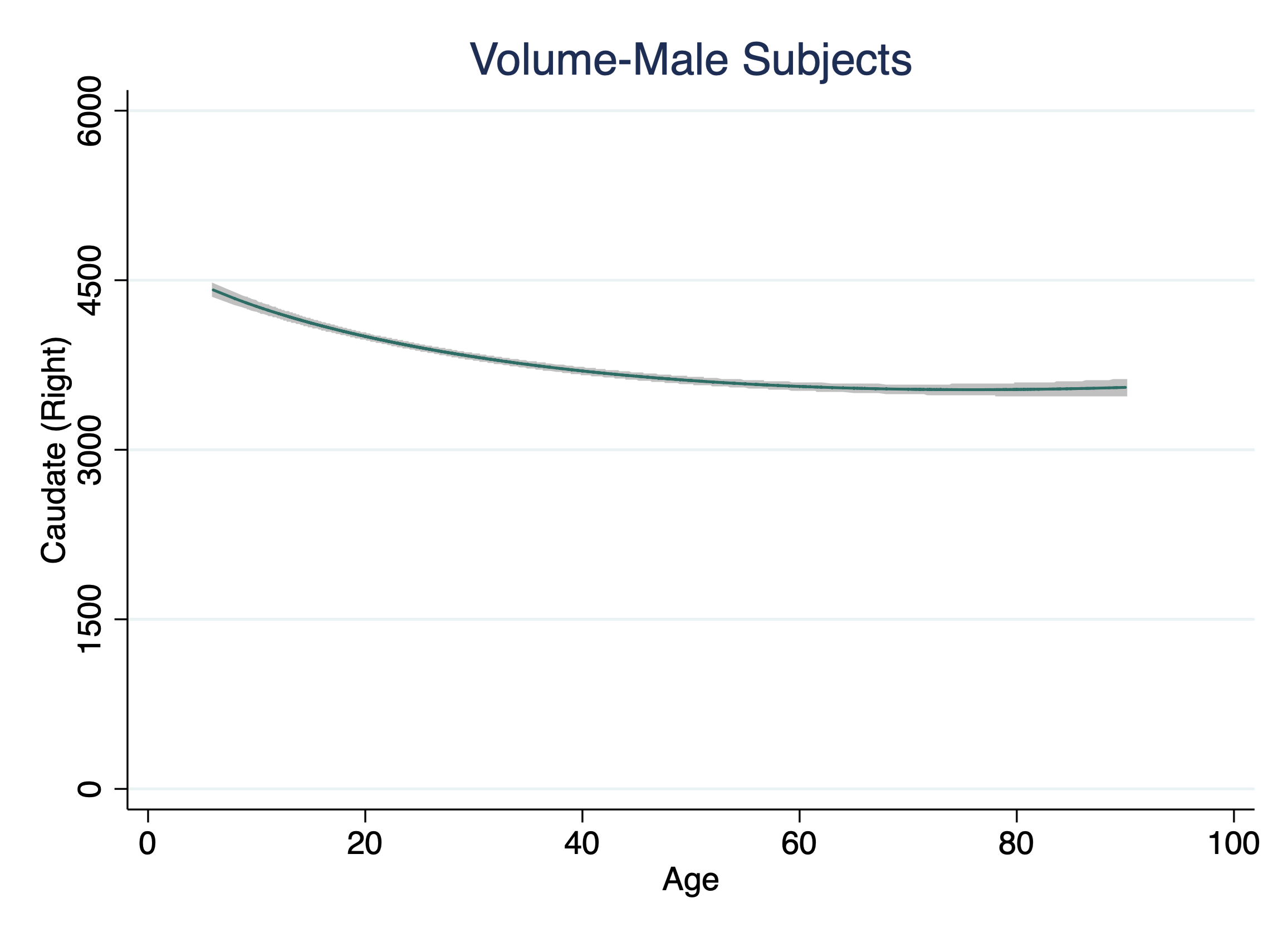

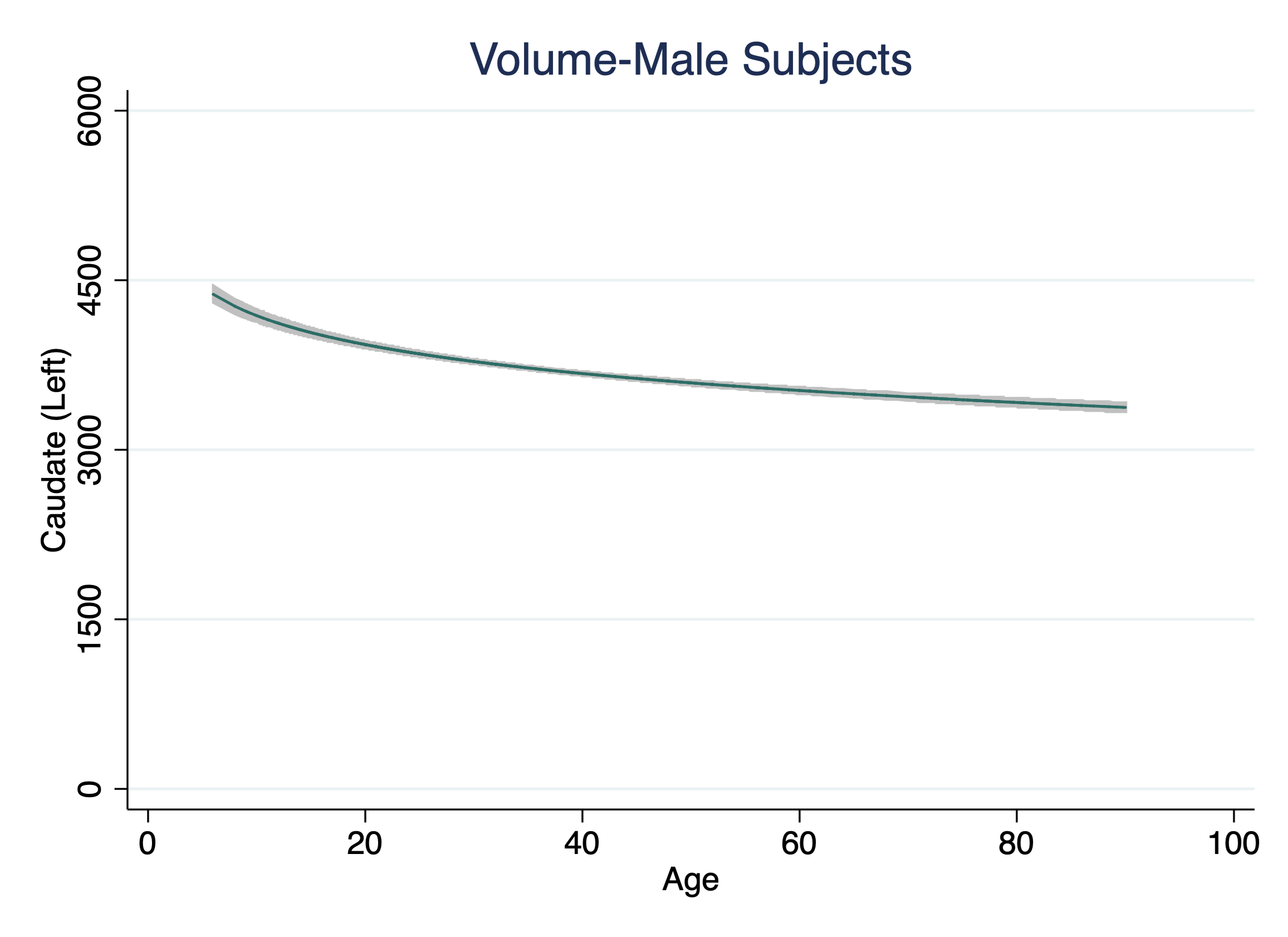


**Figure S3. Age-related trajectories in Globus Pallidus, Putamen and Caudate in Females**


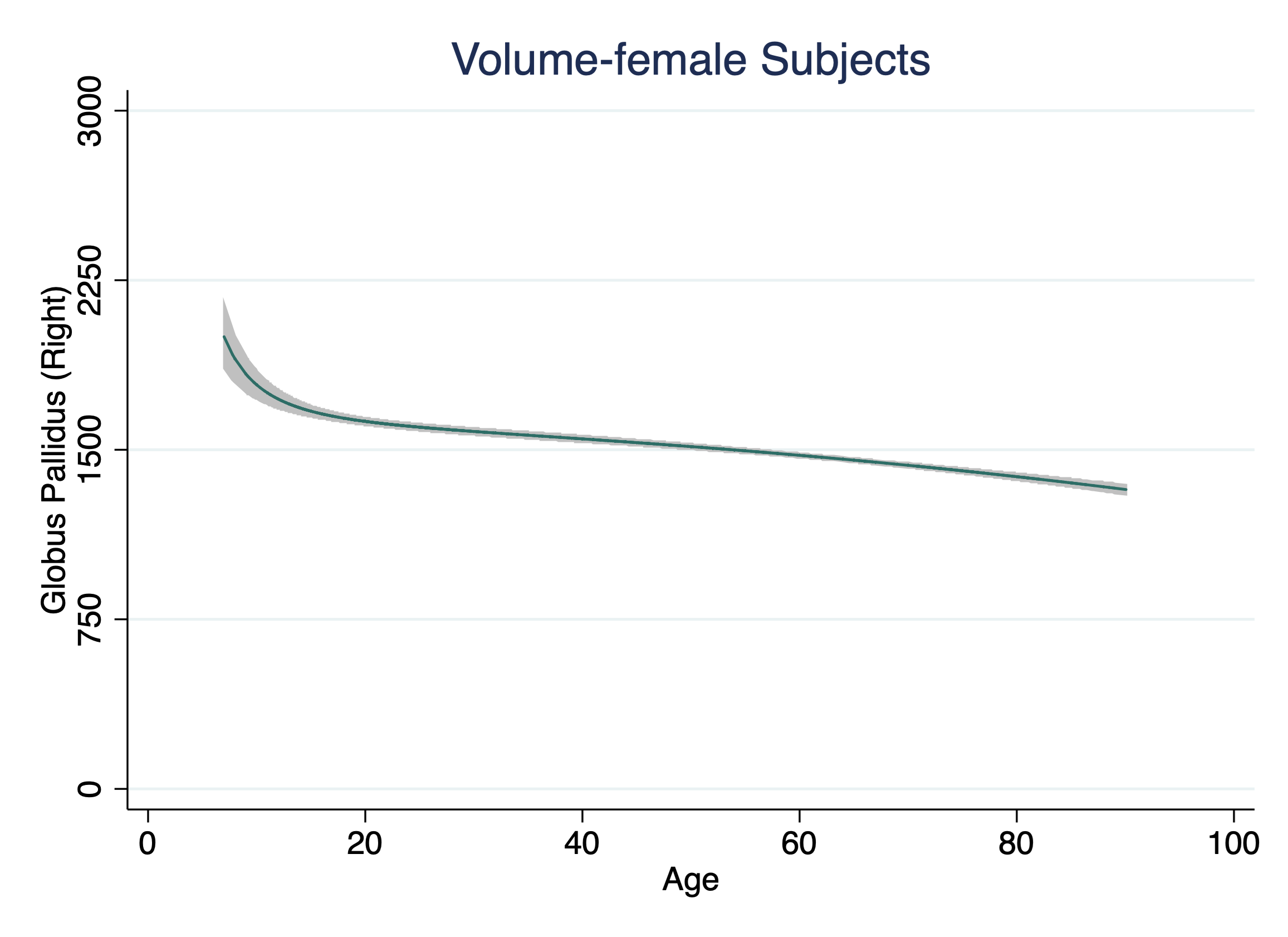

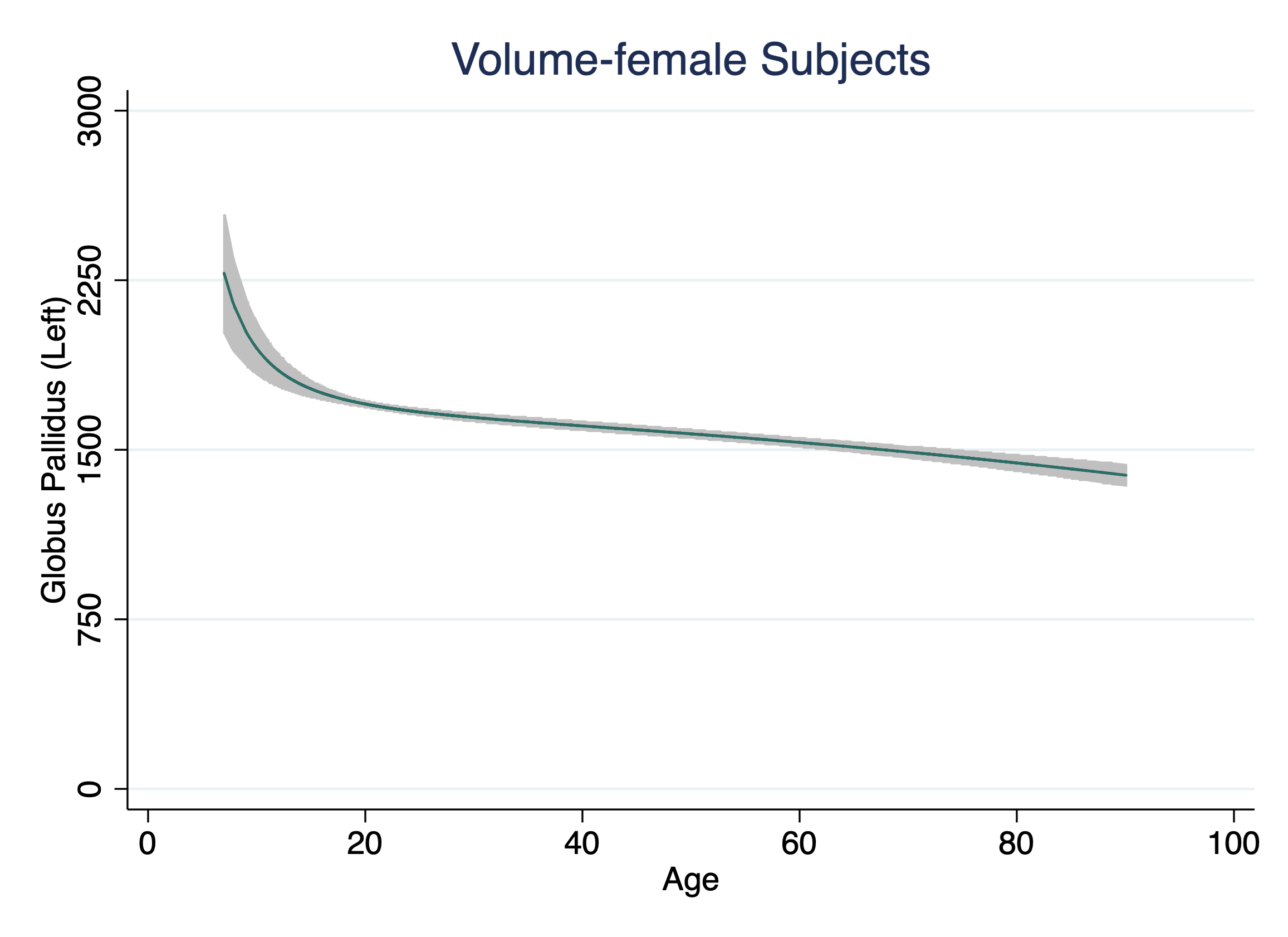

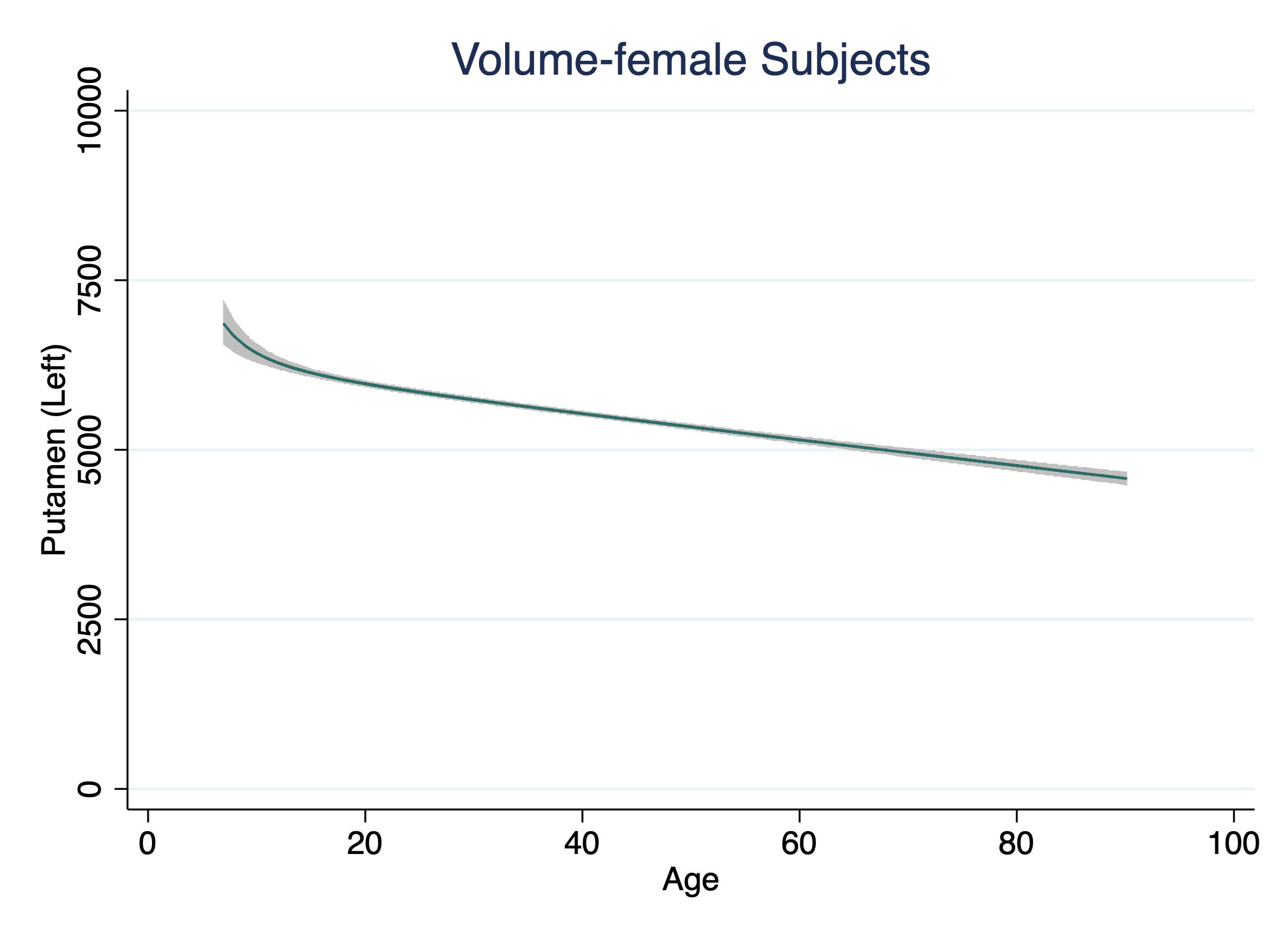

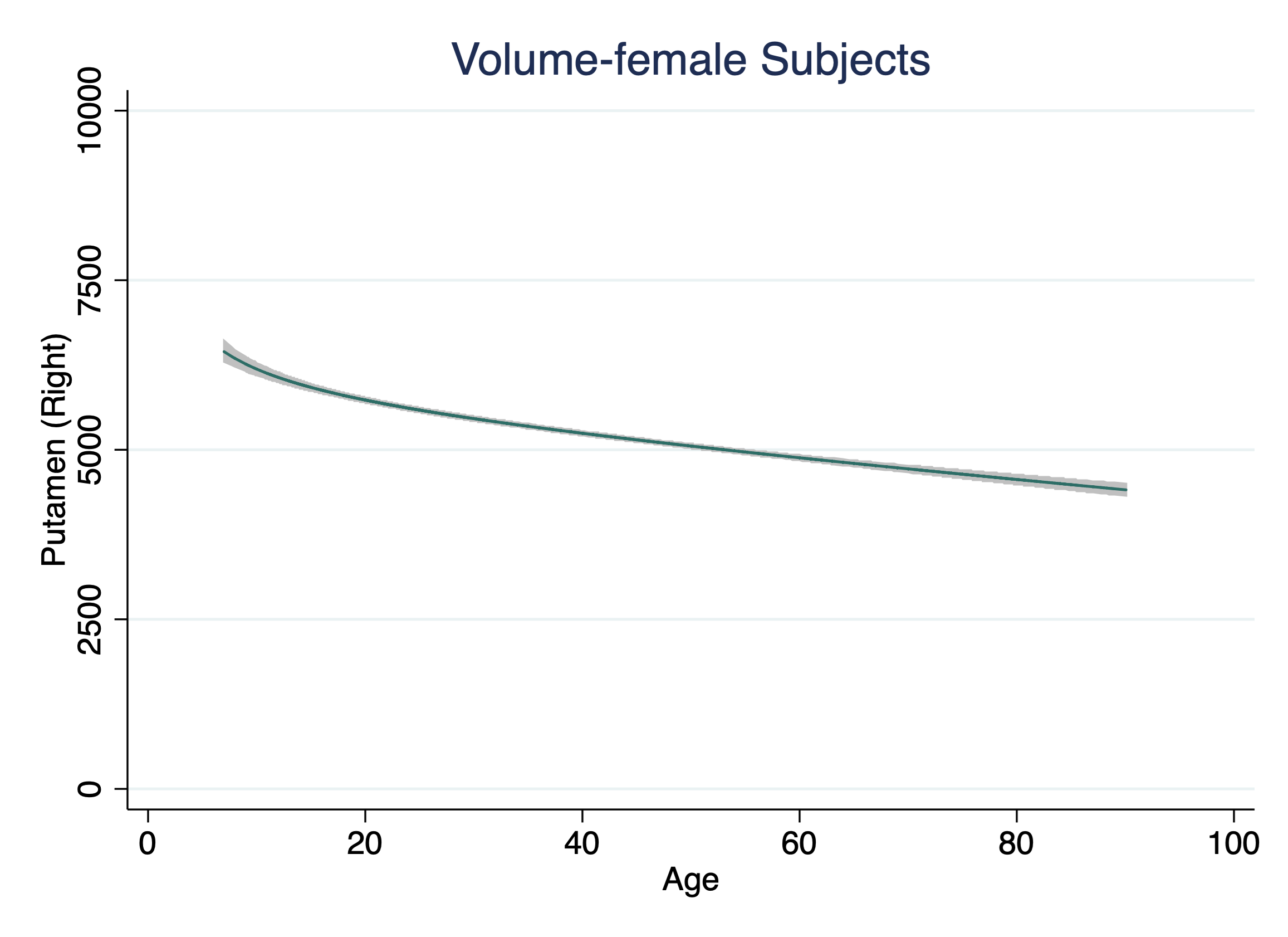

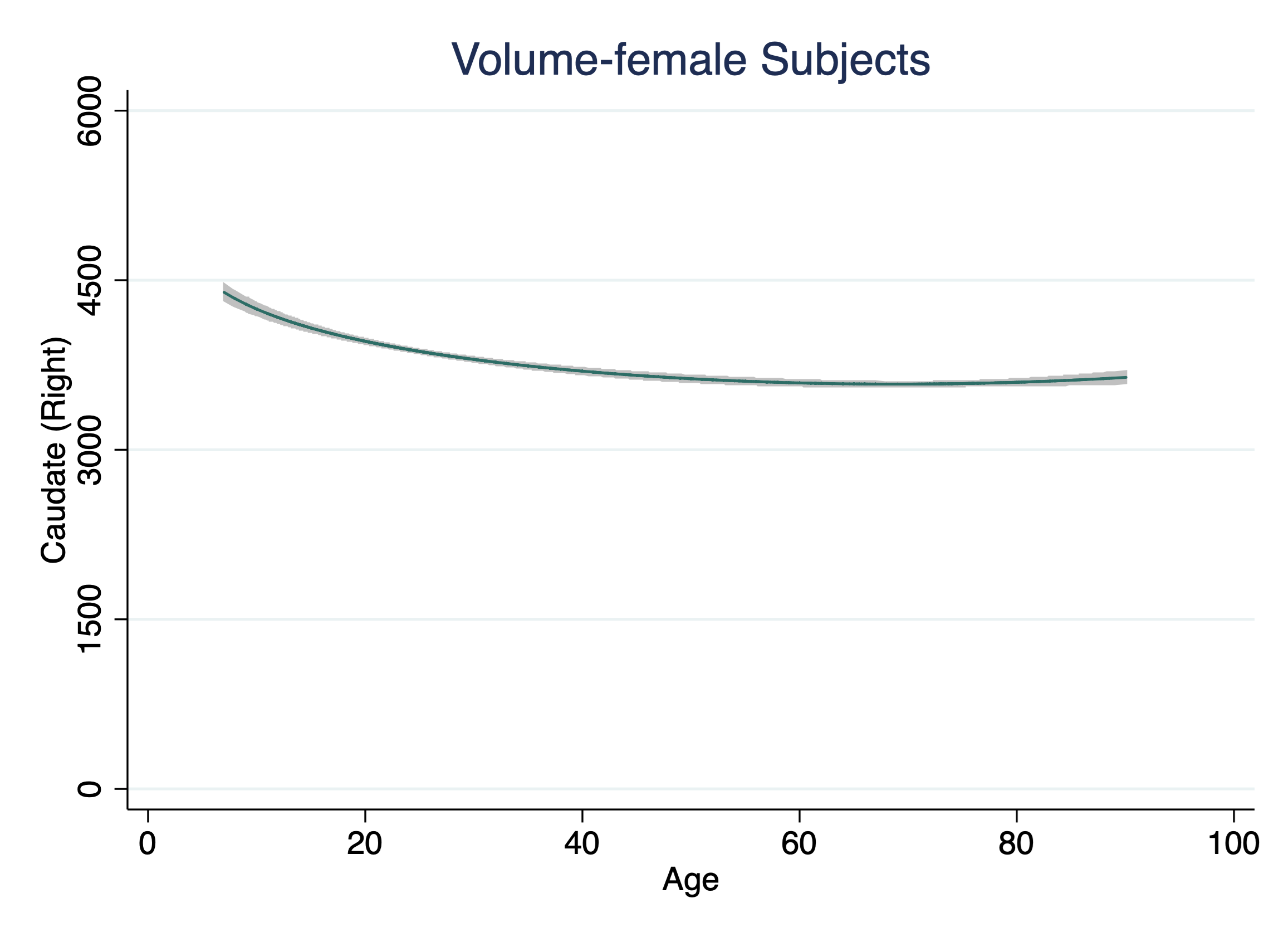

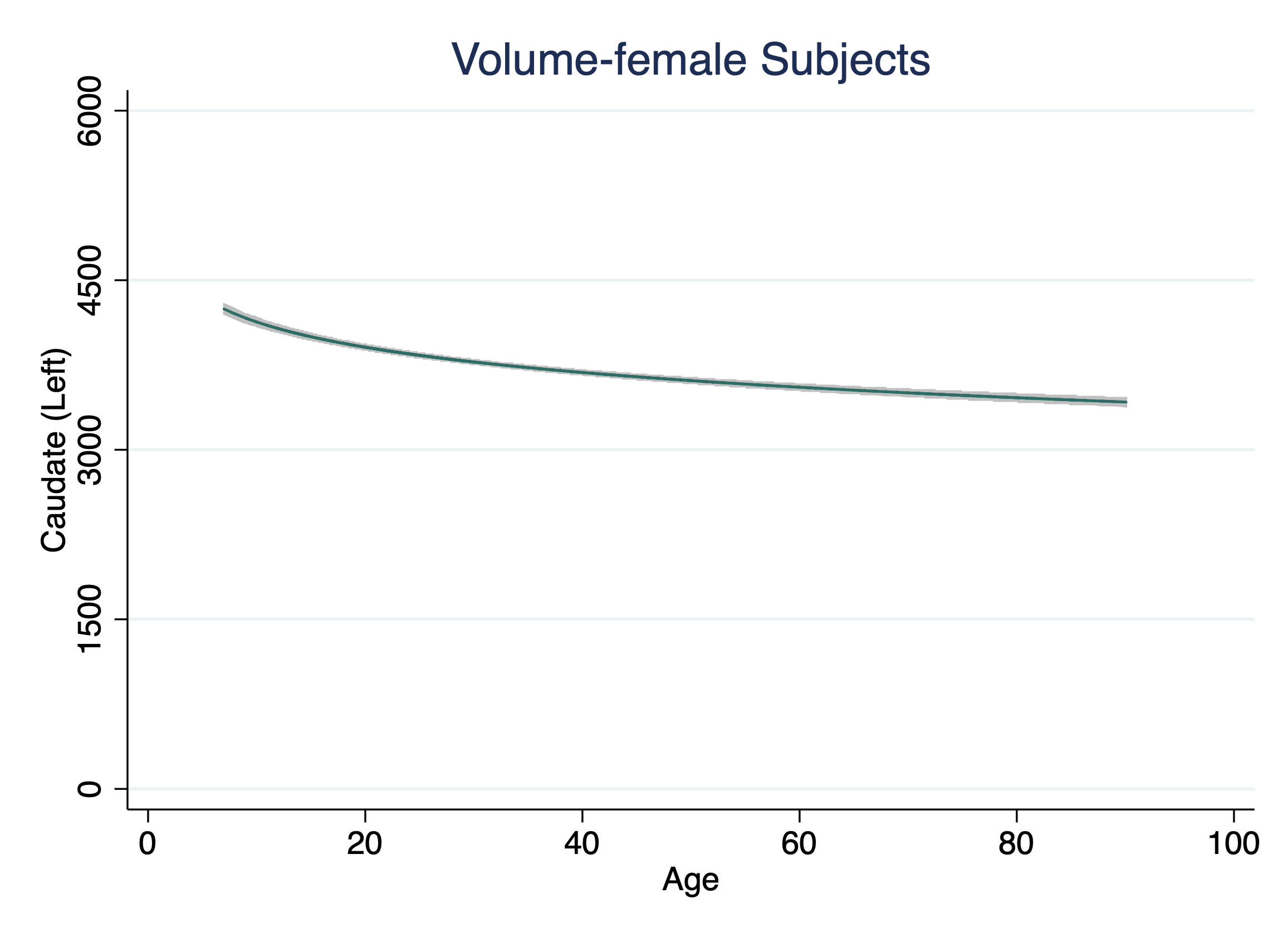


**Figure S4. Age-related Trajectories in Nucleus Accumbens**


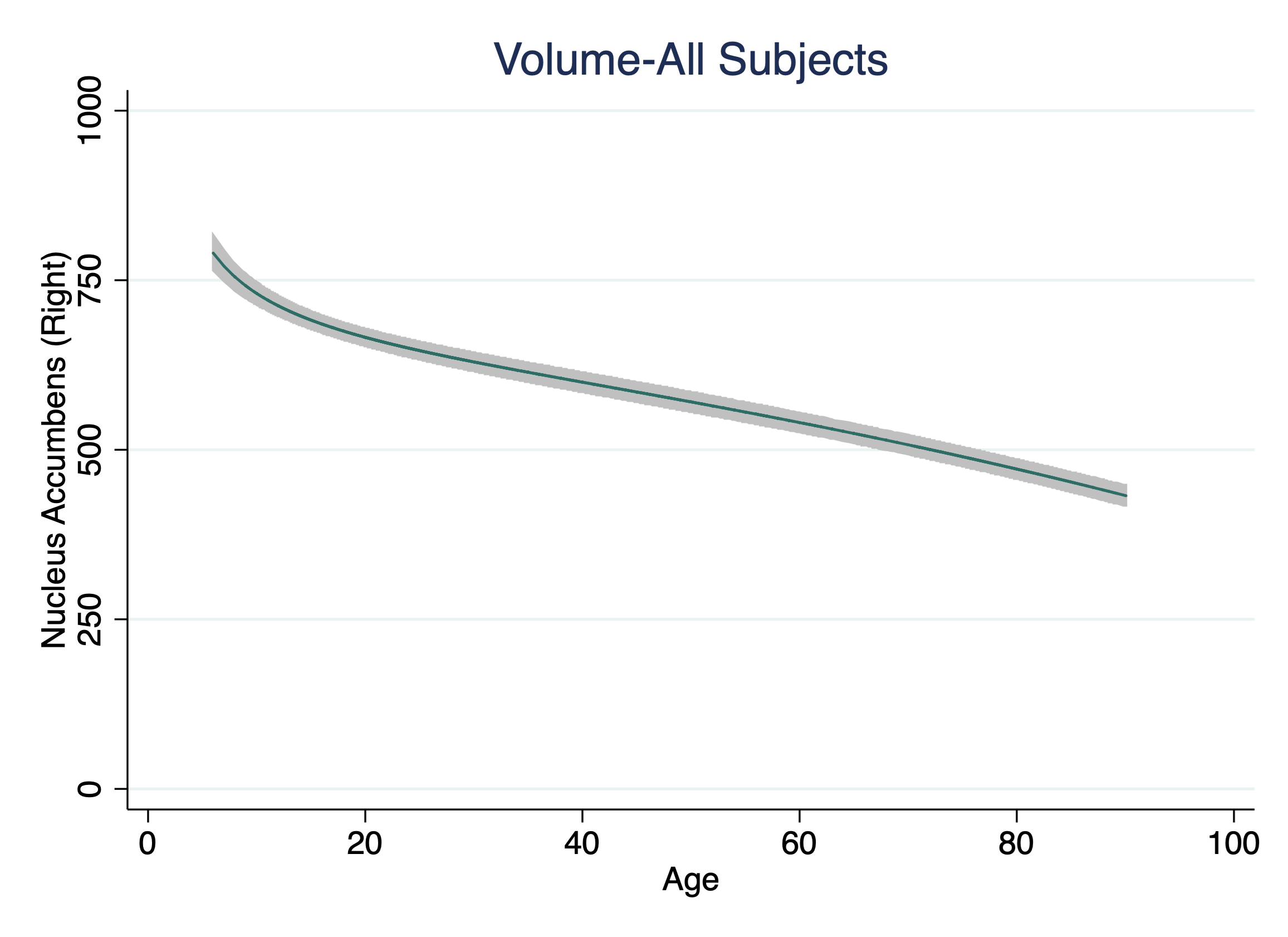

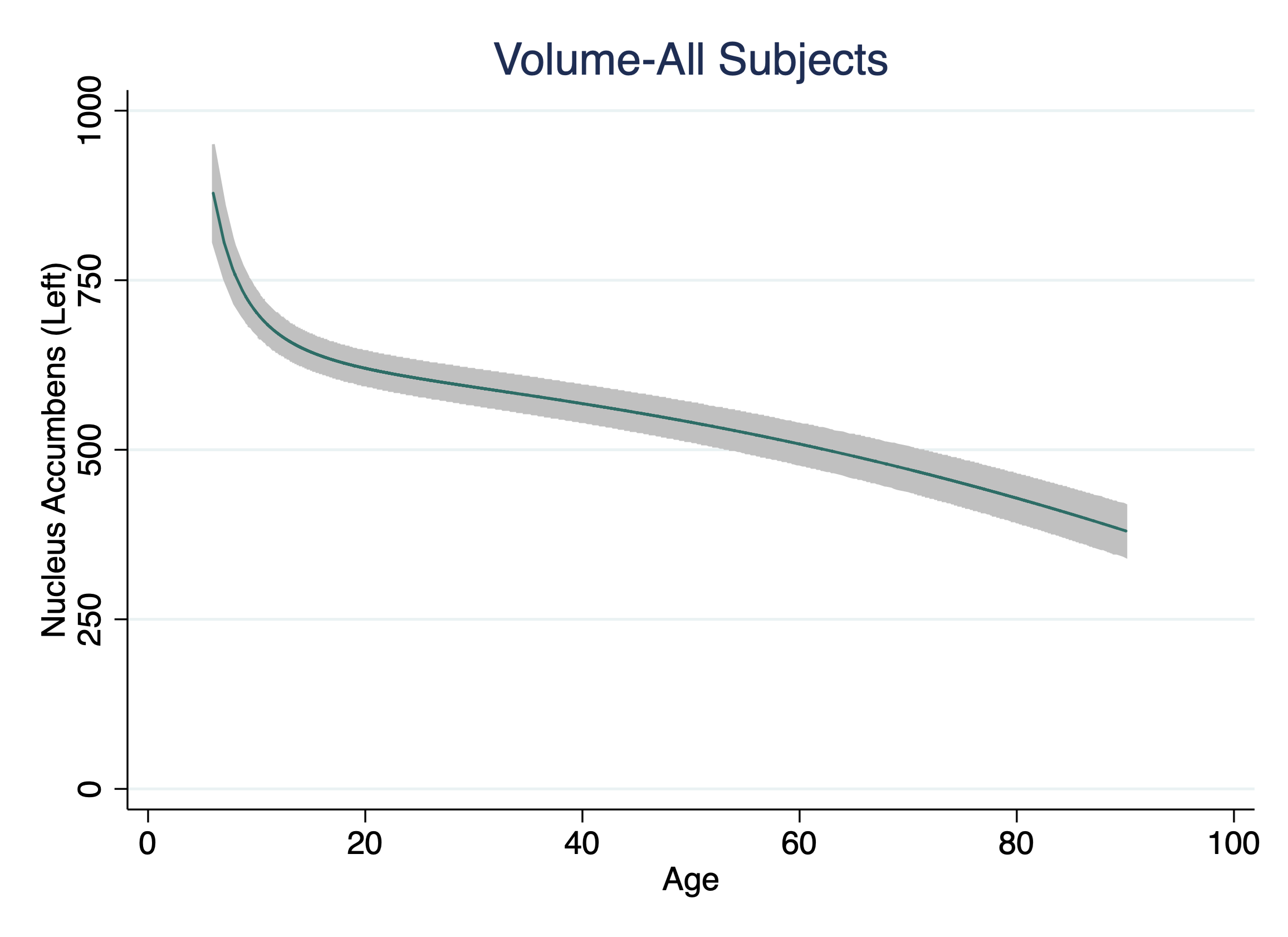

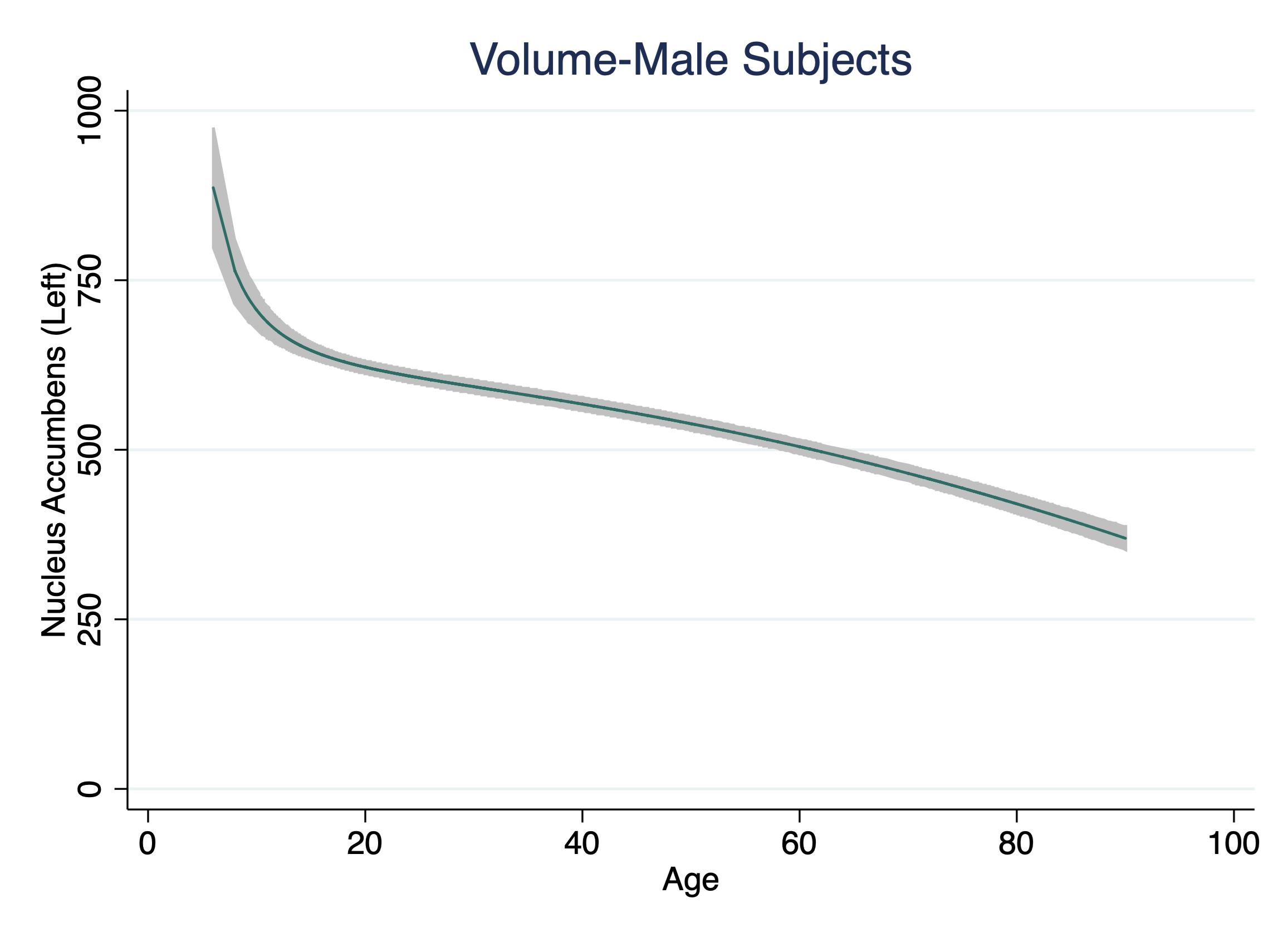

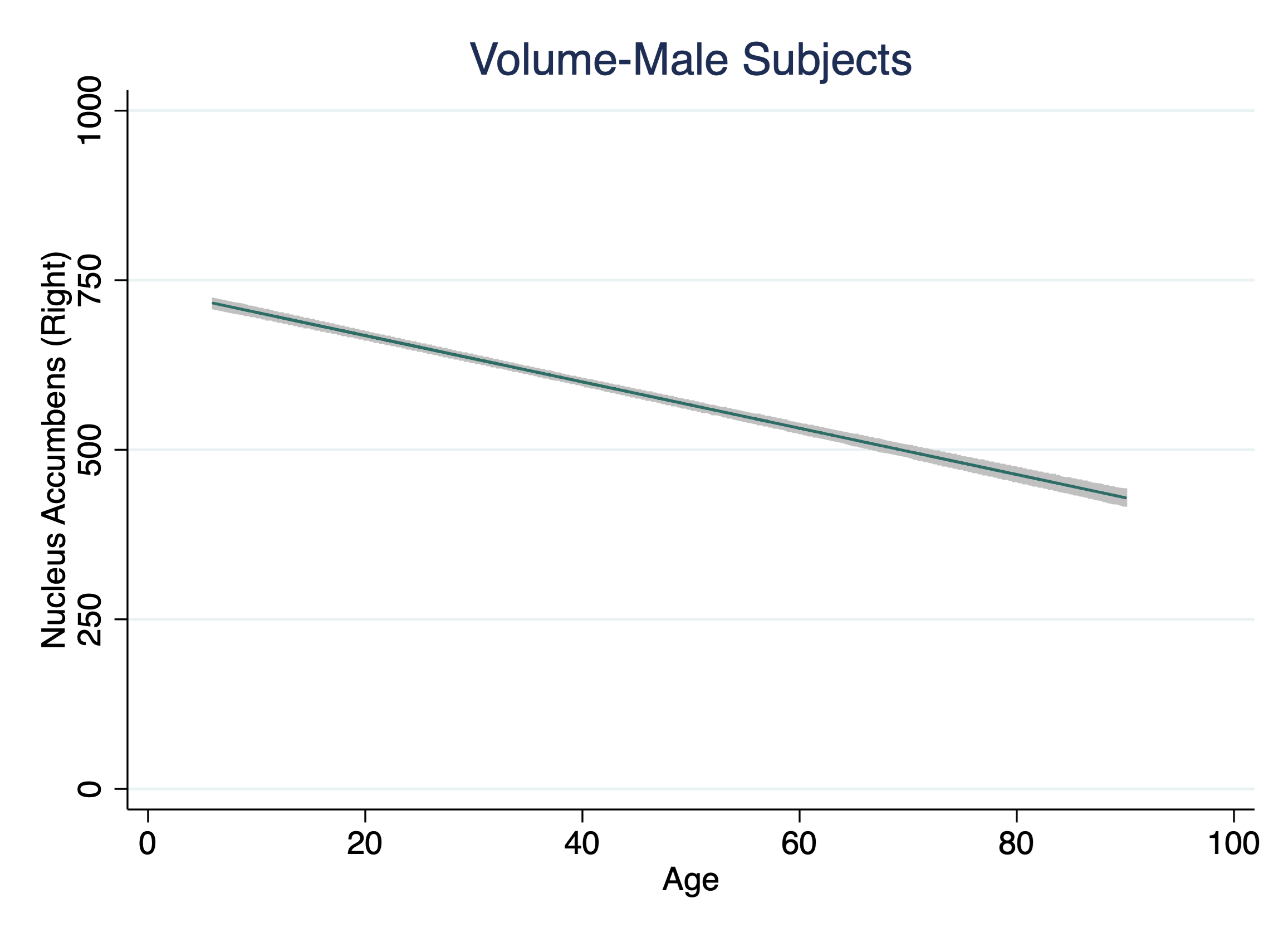

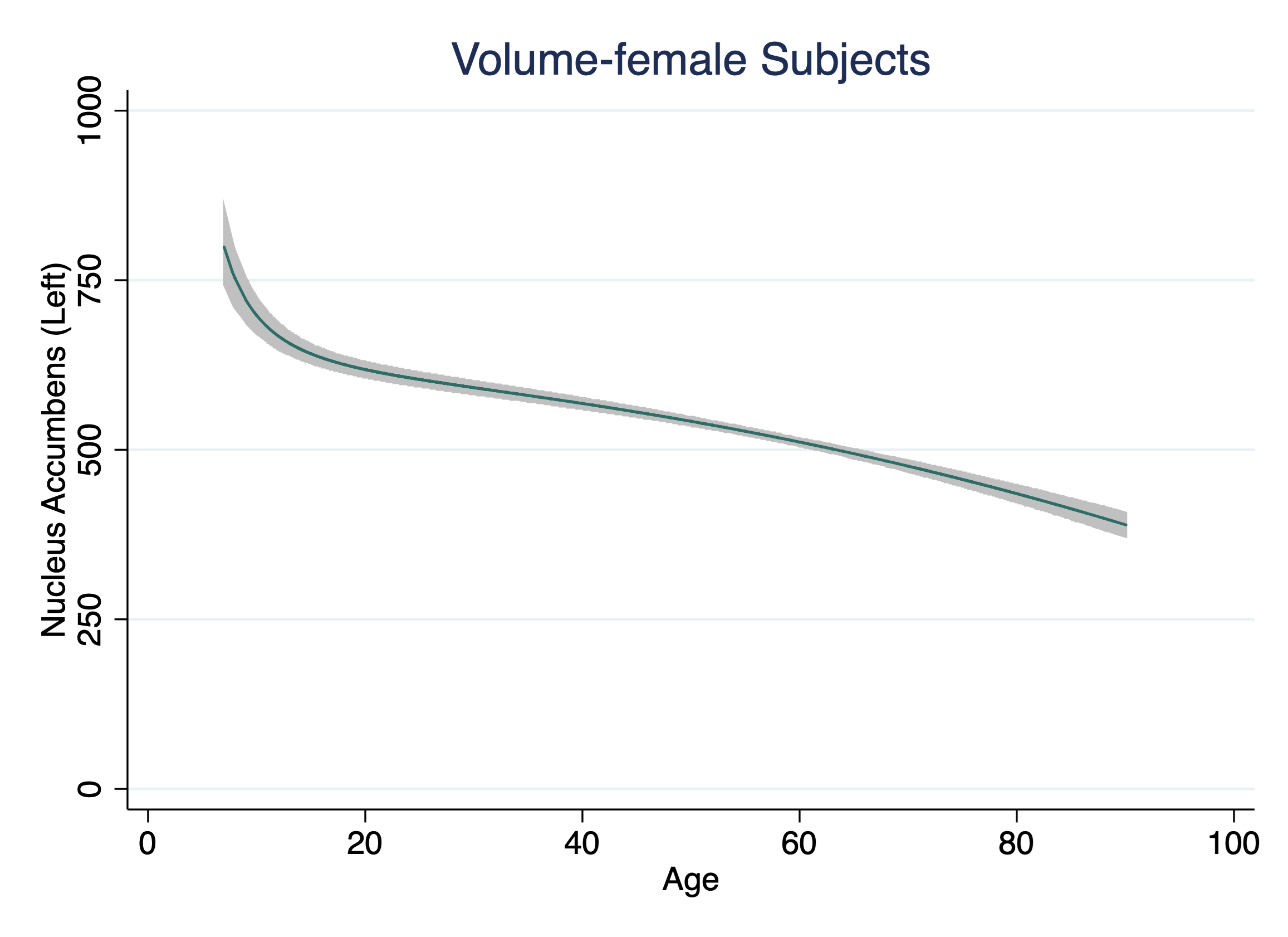

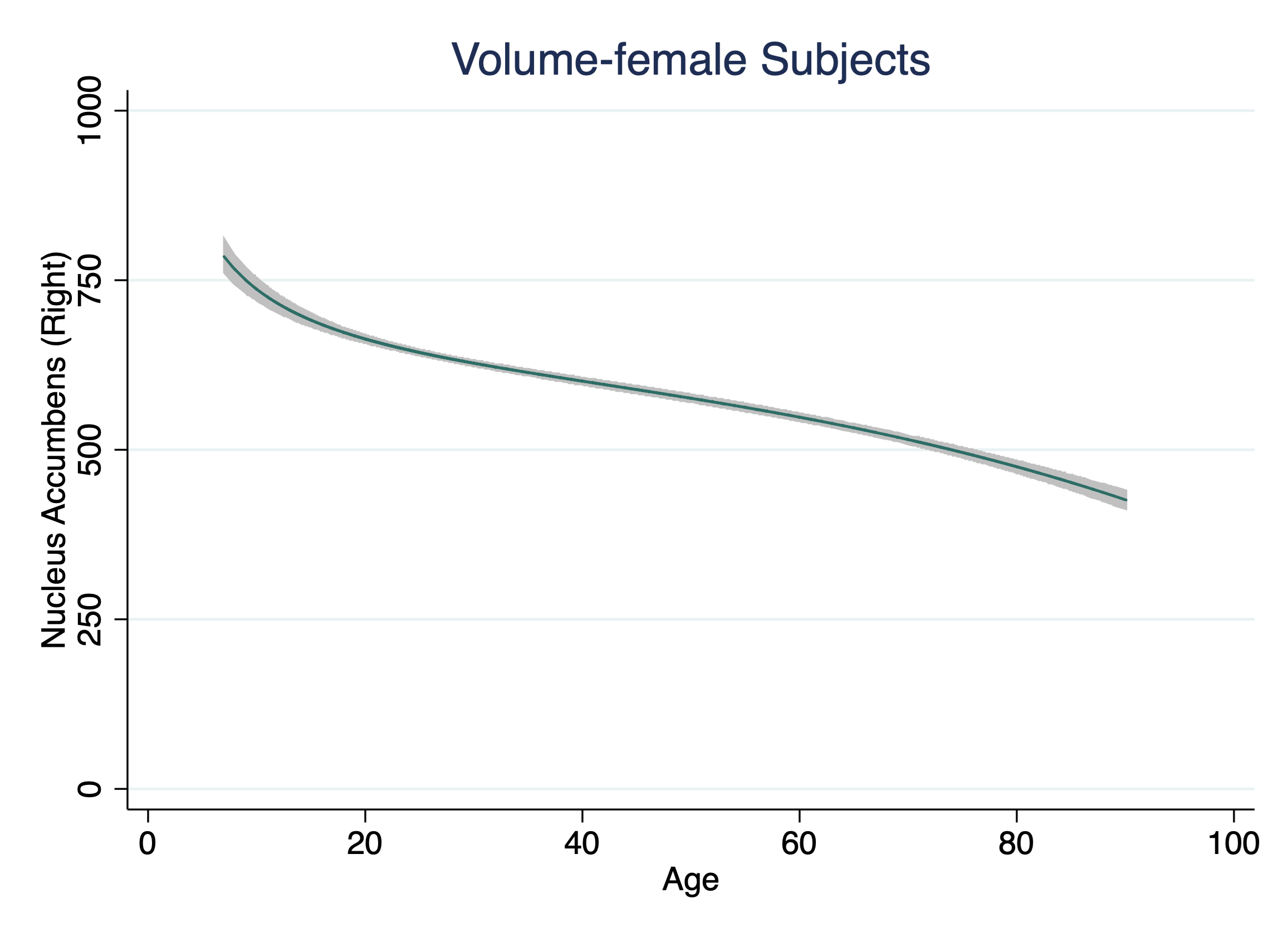


**Figure S5. Age-related Trajectories in Thalamus, Hippocampus, and Amygdala in Males**


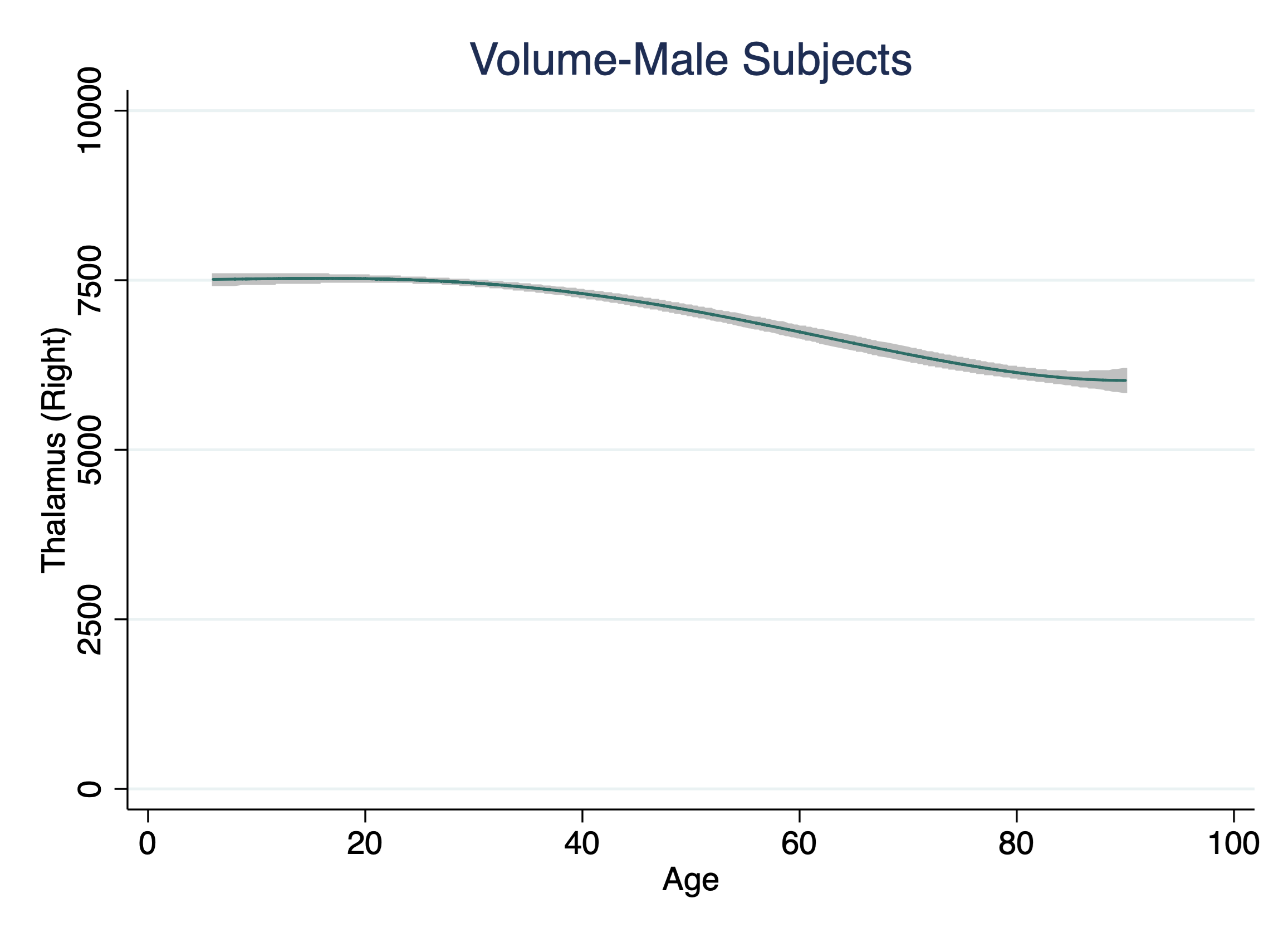

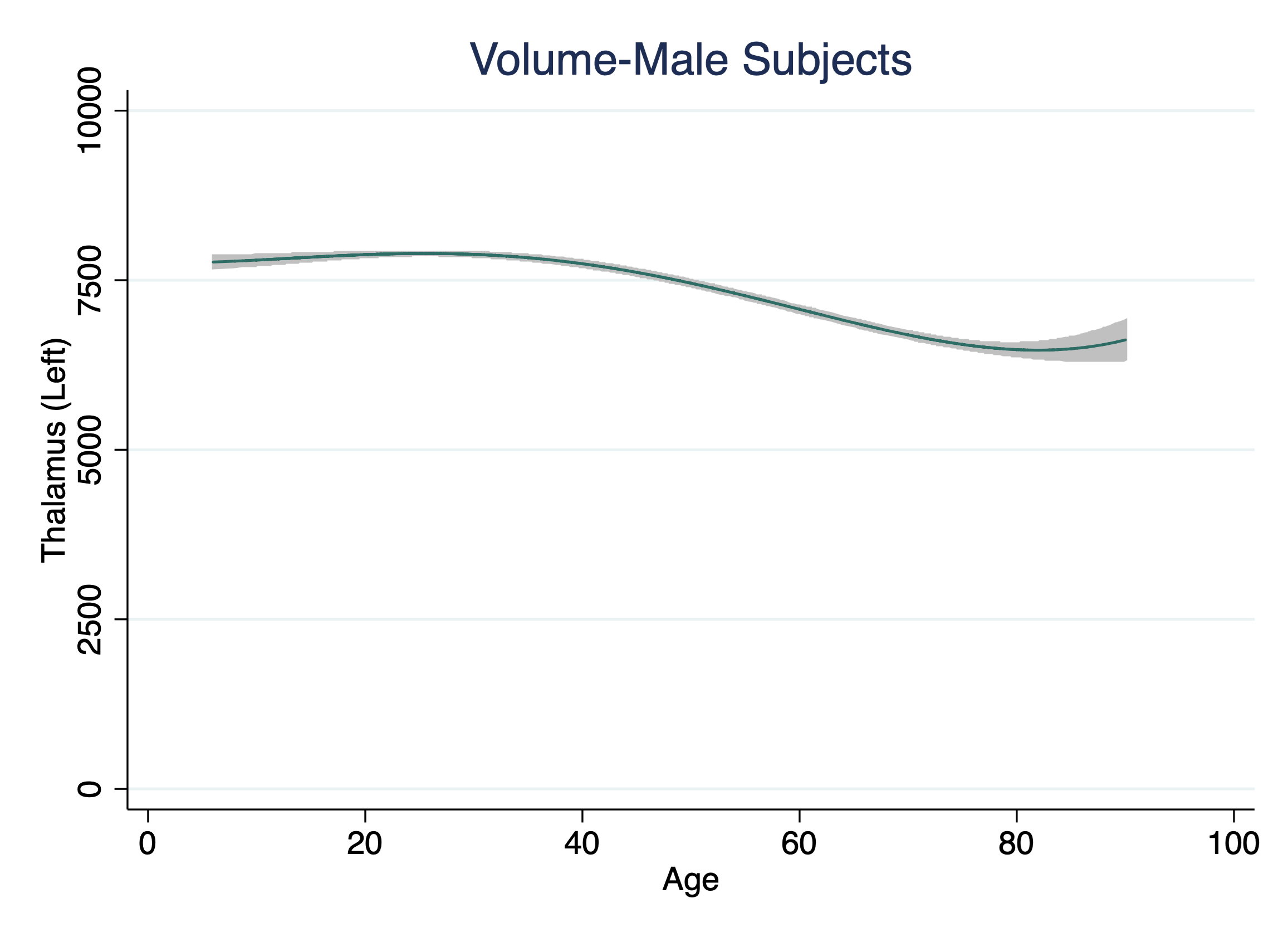

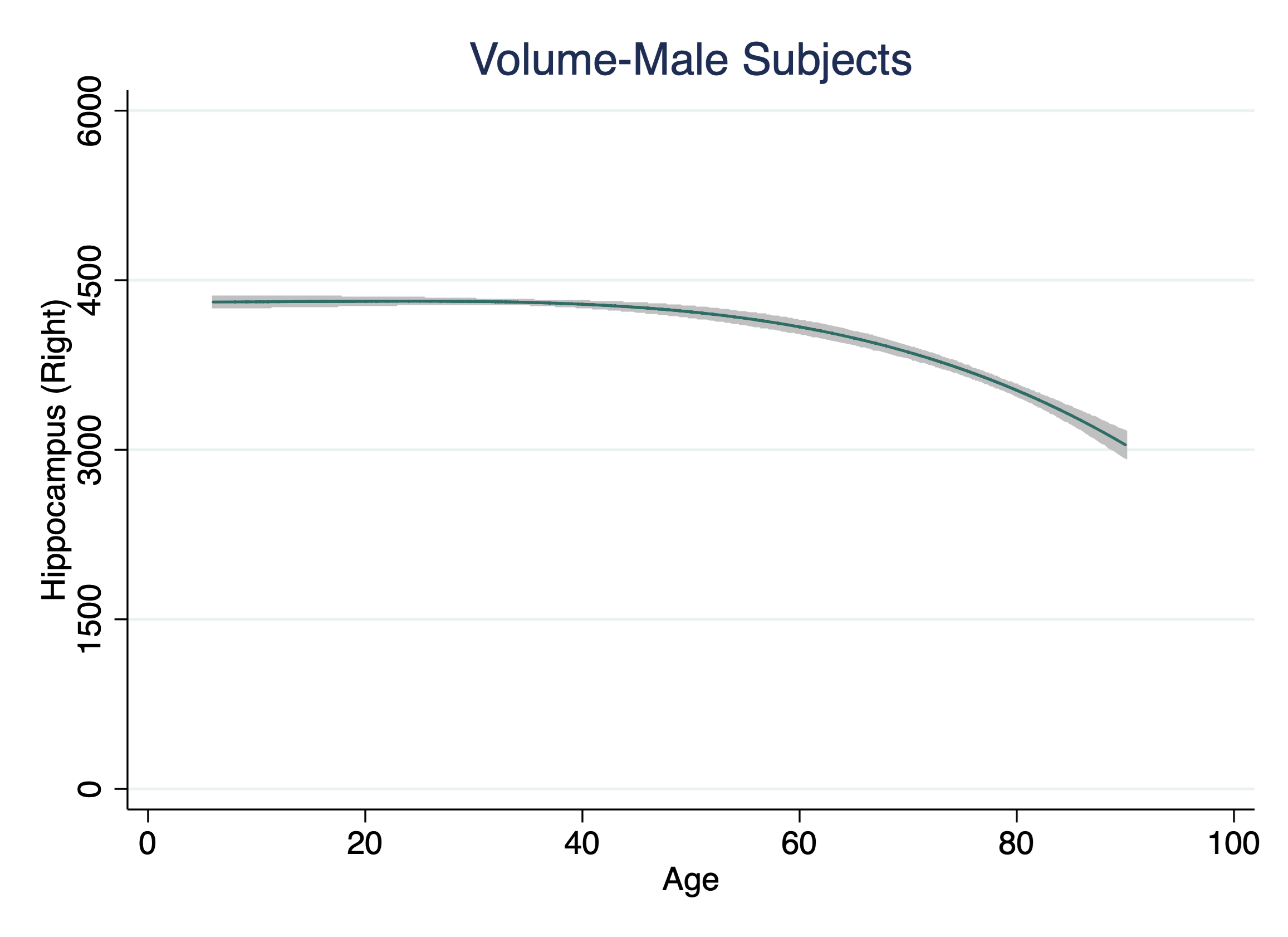

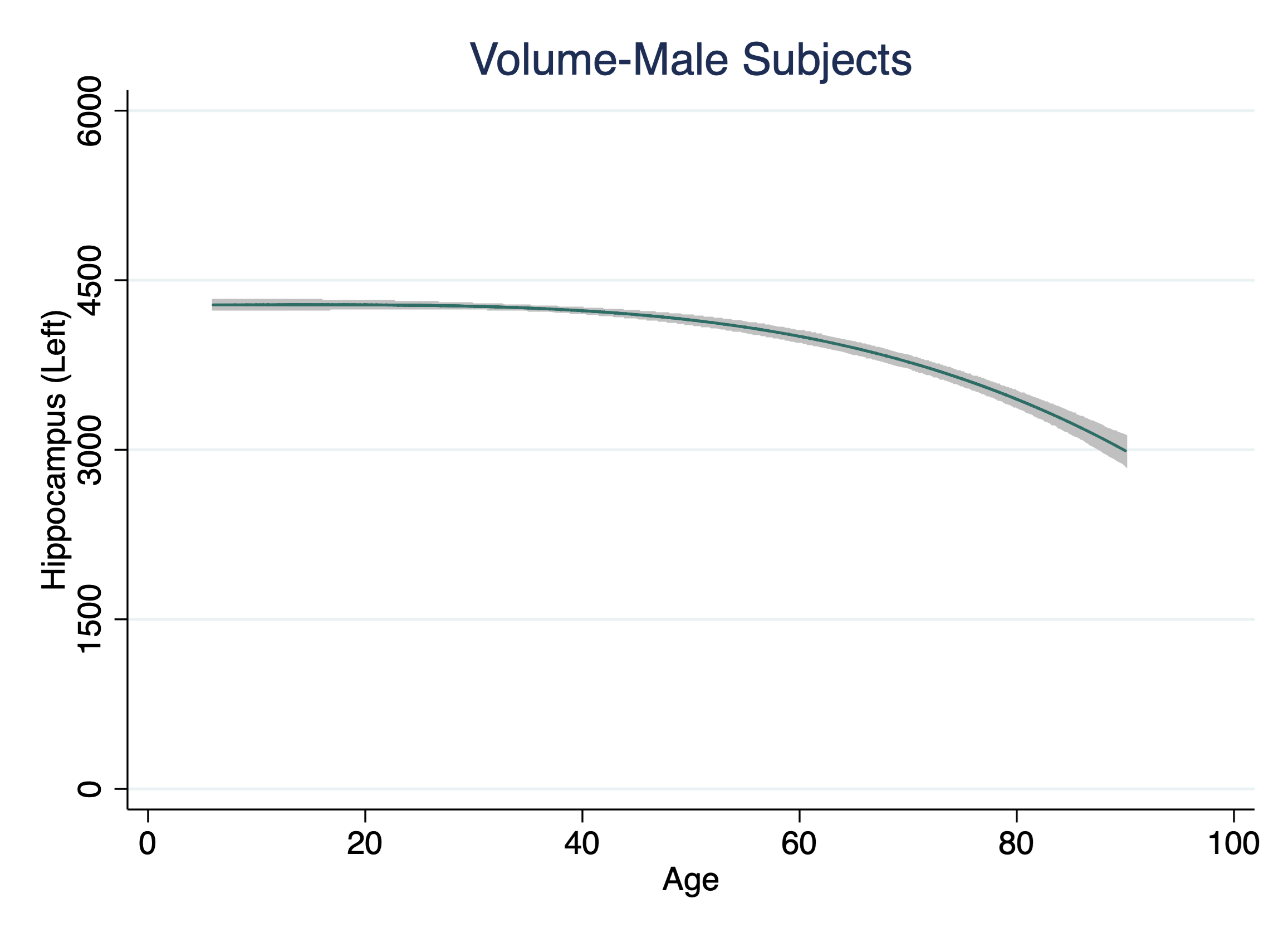

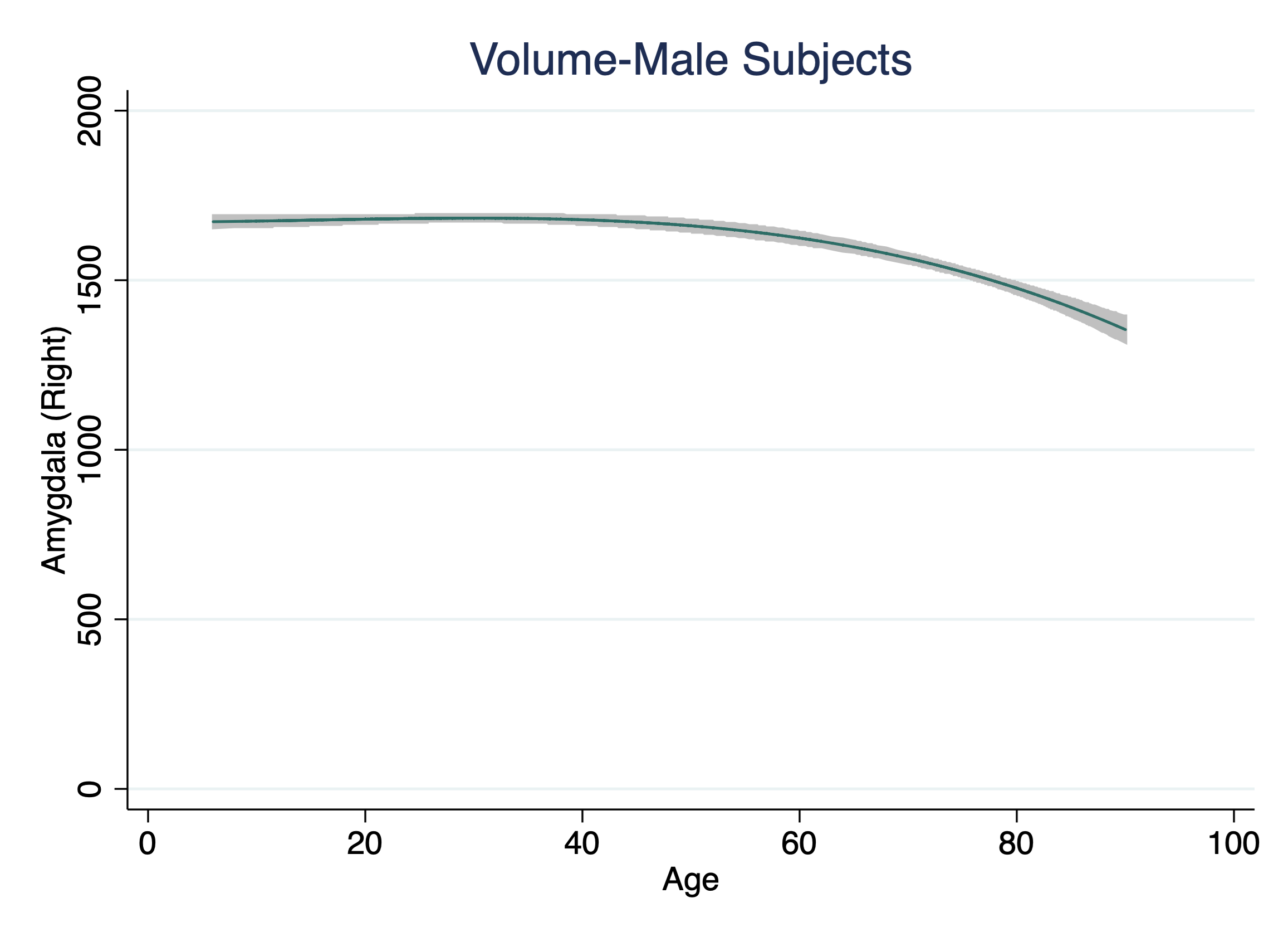

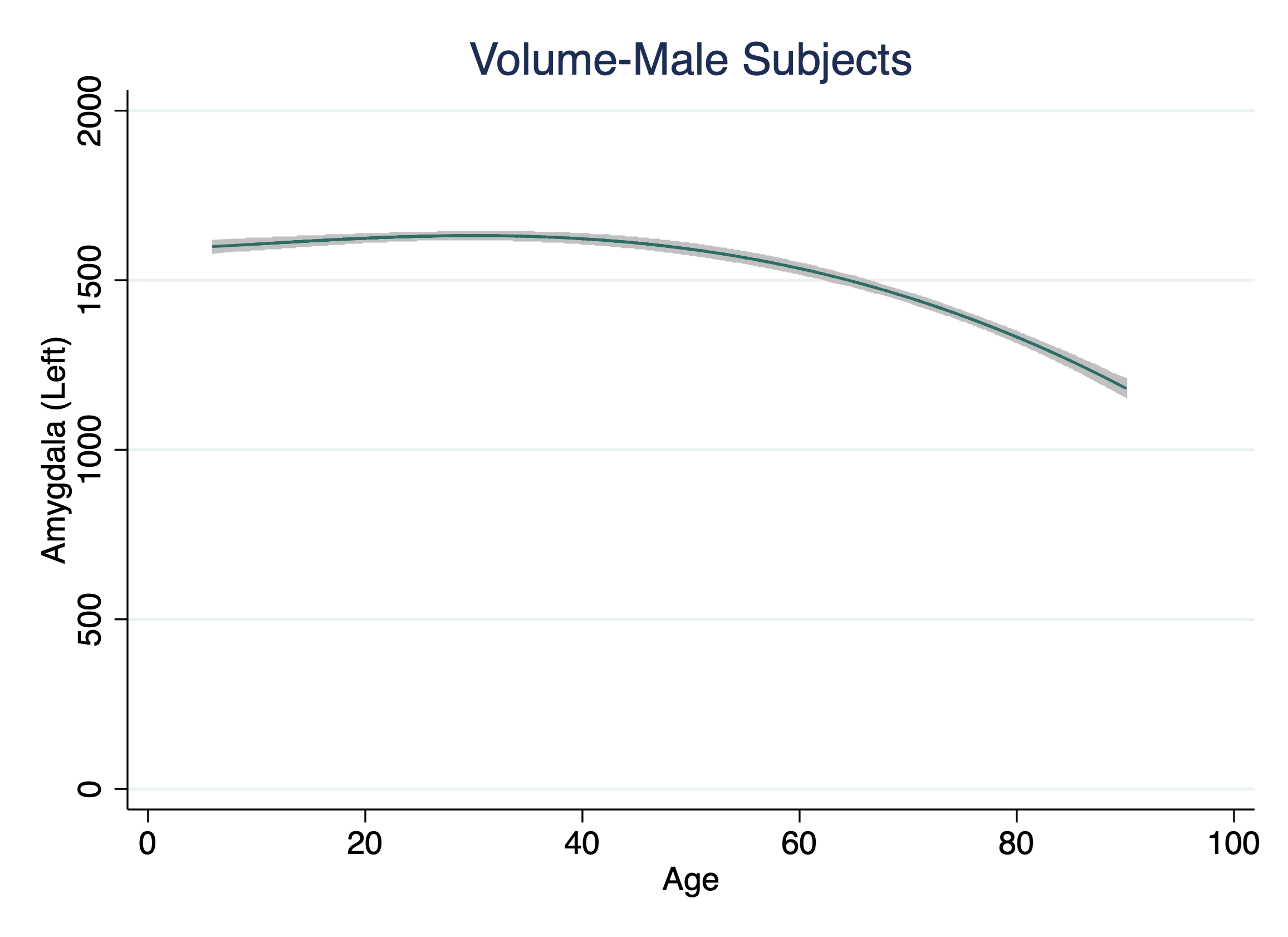


**Figure S6. Age-related Trajectories in Thalamus, Hippocampus, and Amygdala in Females**


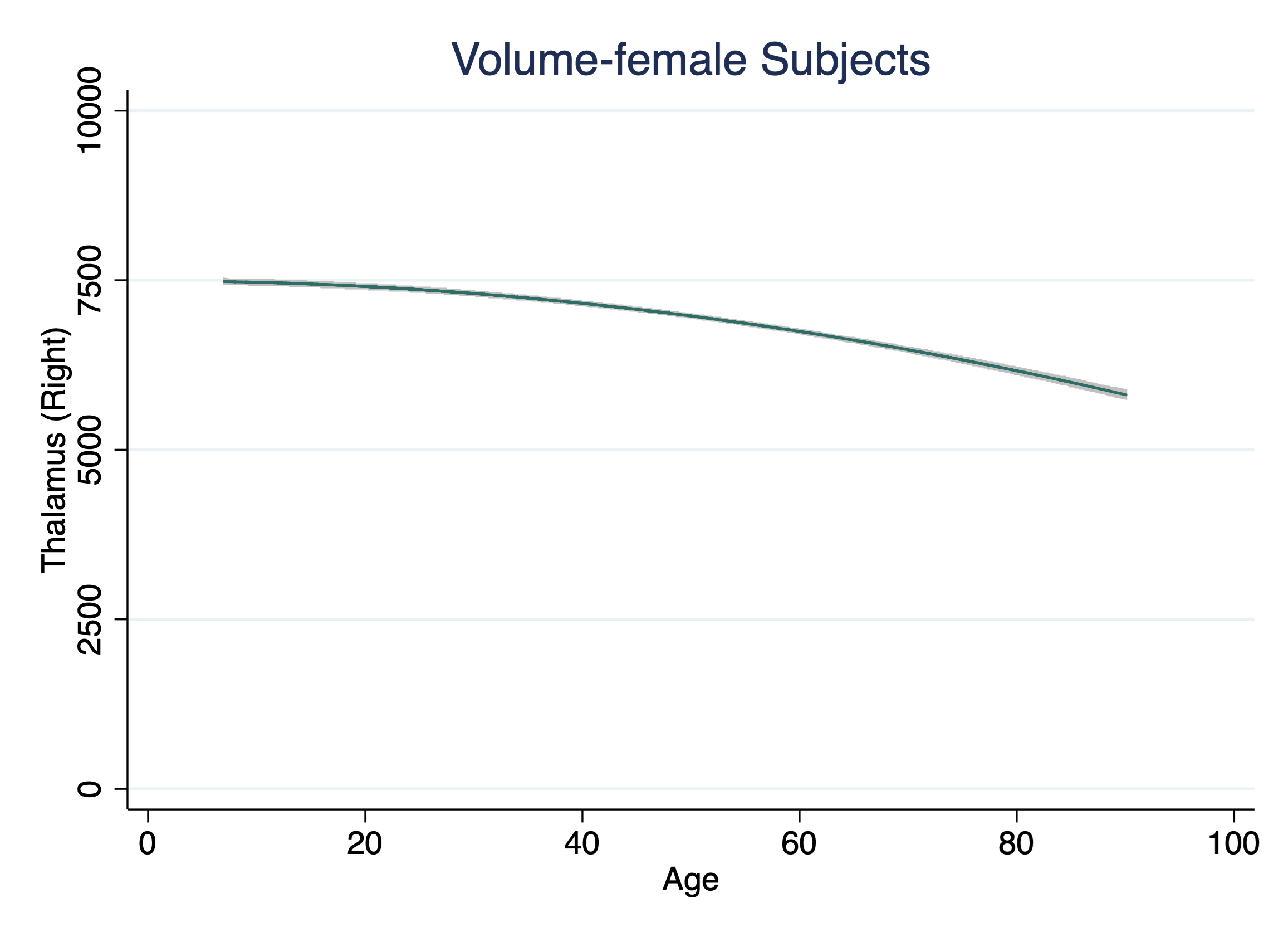

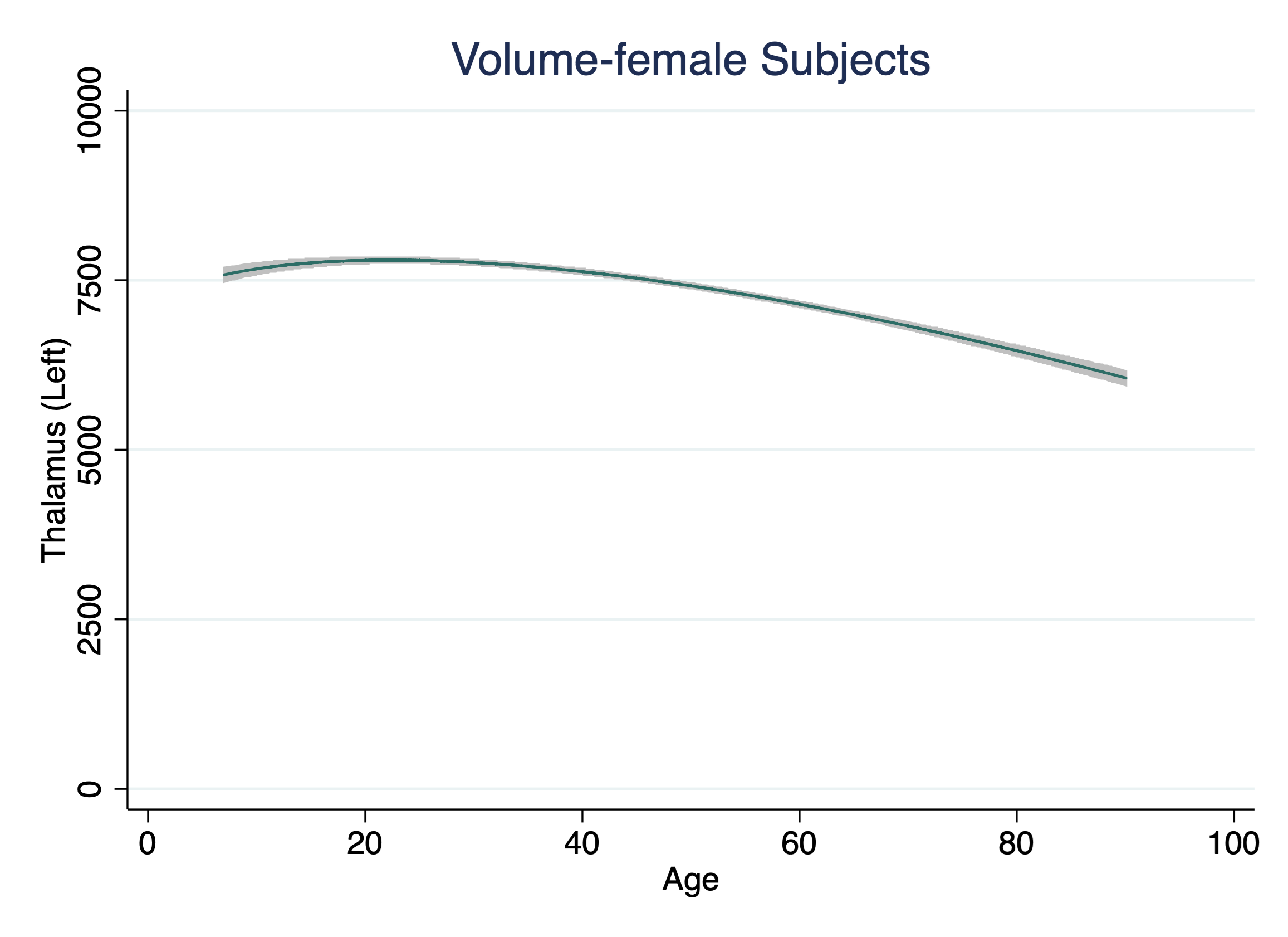

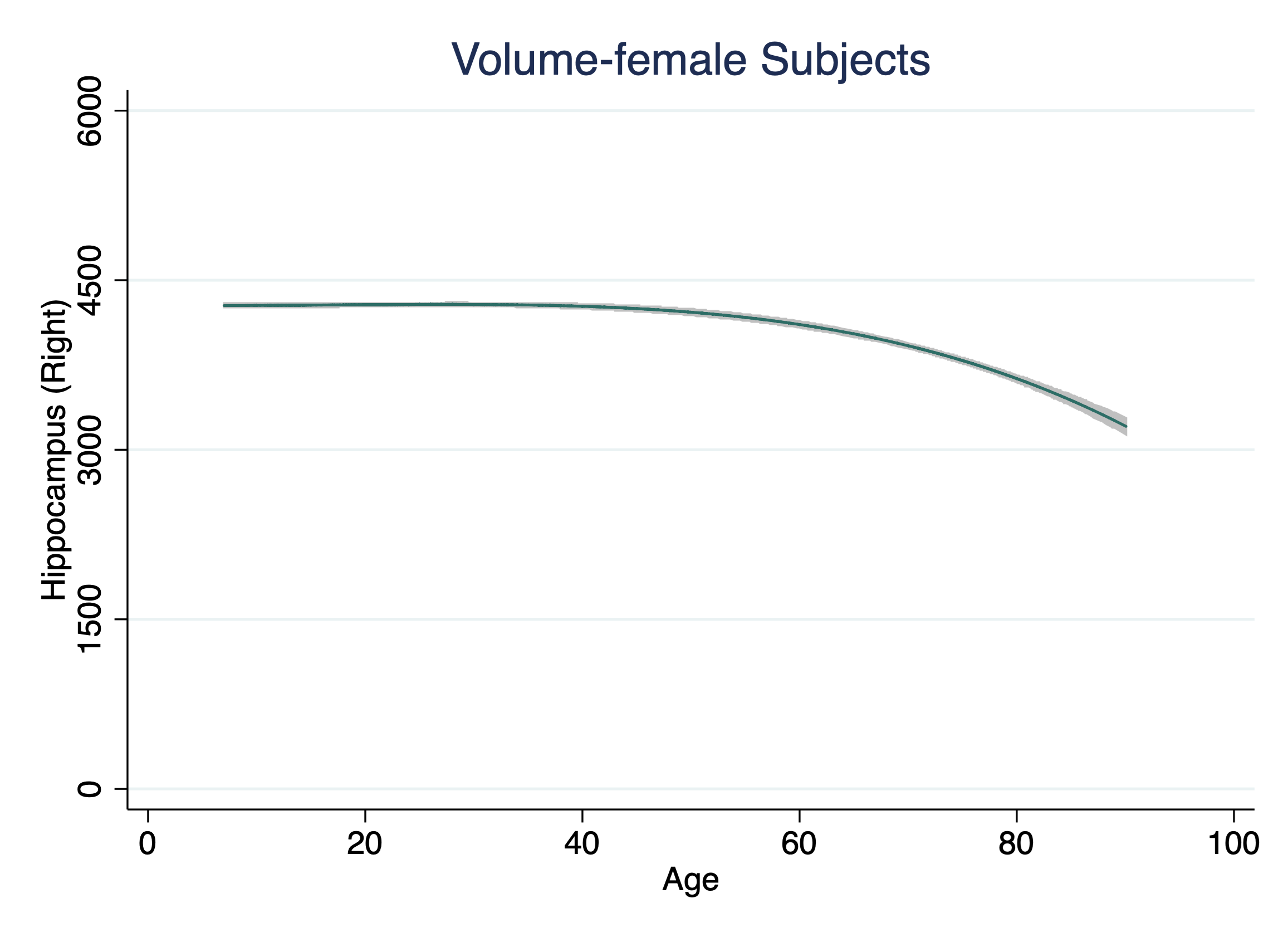

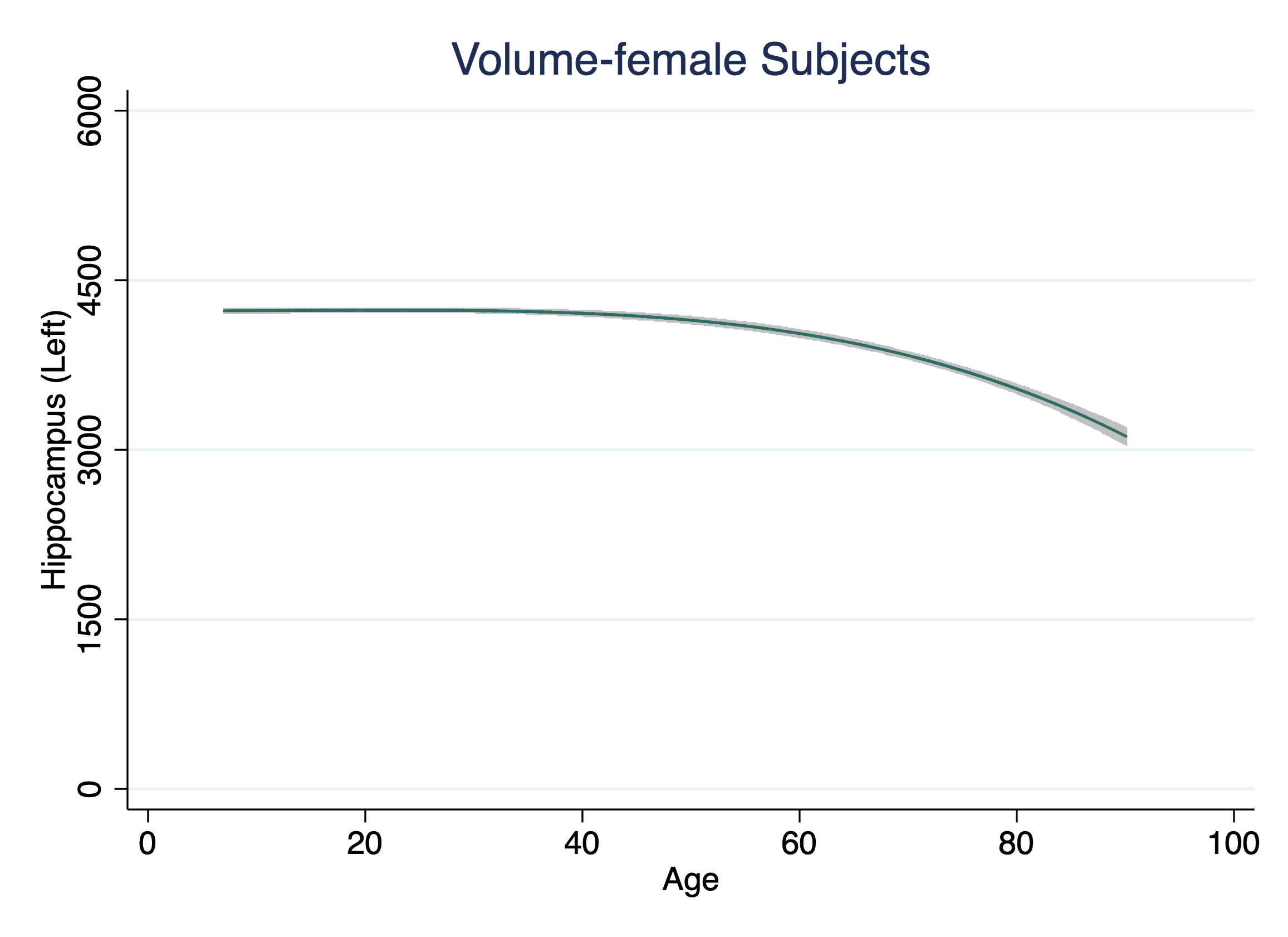

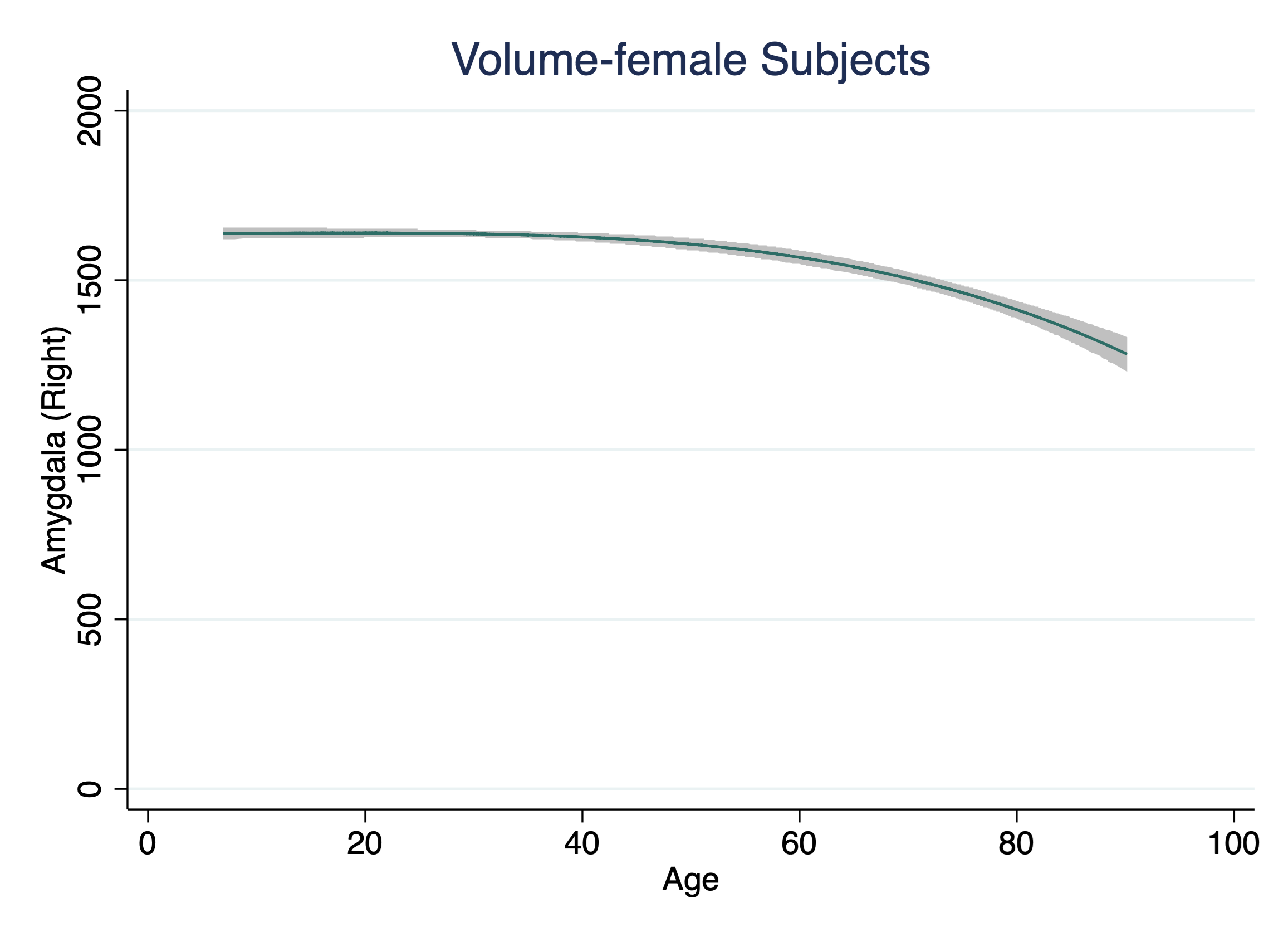

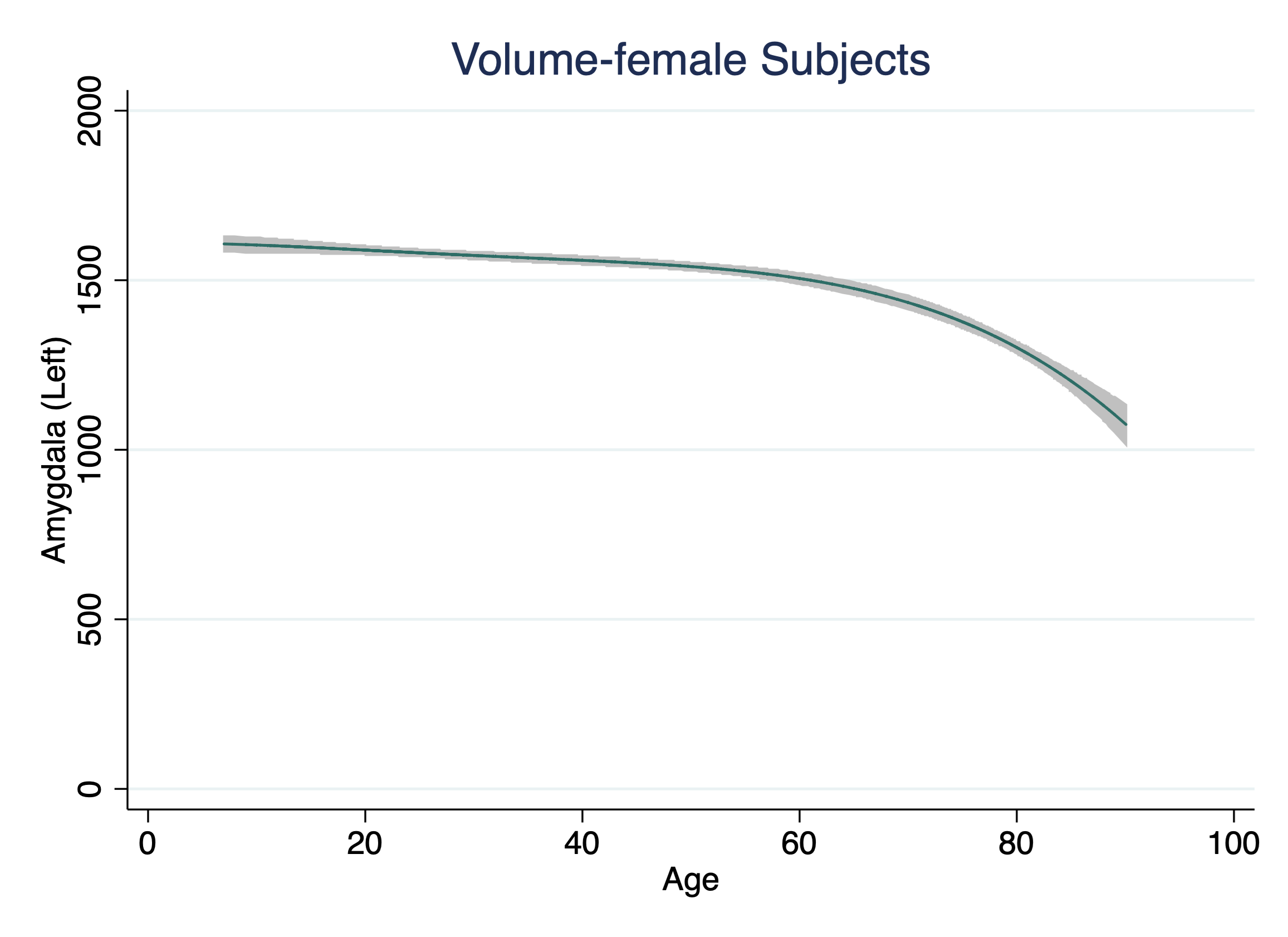


**Figure S7. Age-related Trajectories in Lateral Ventricles**


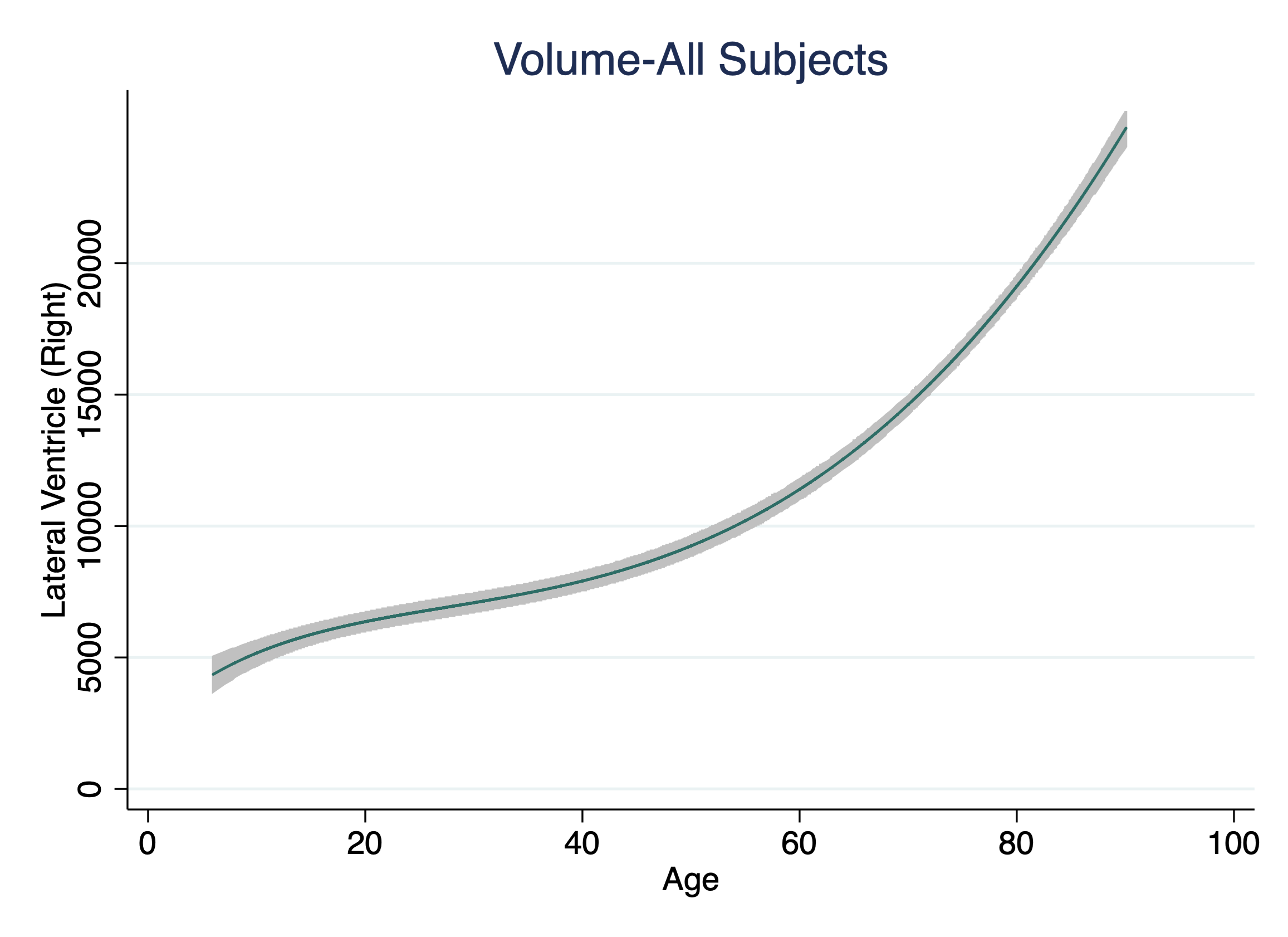

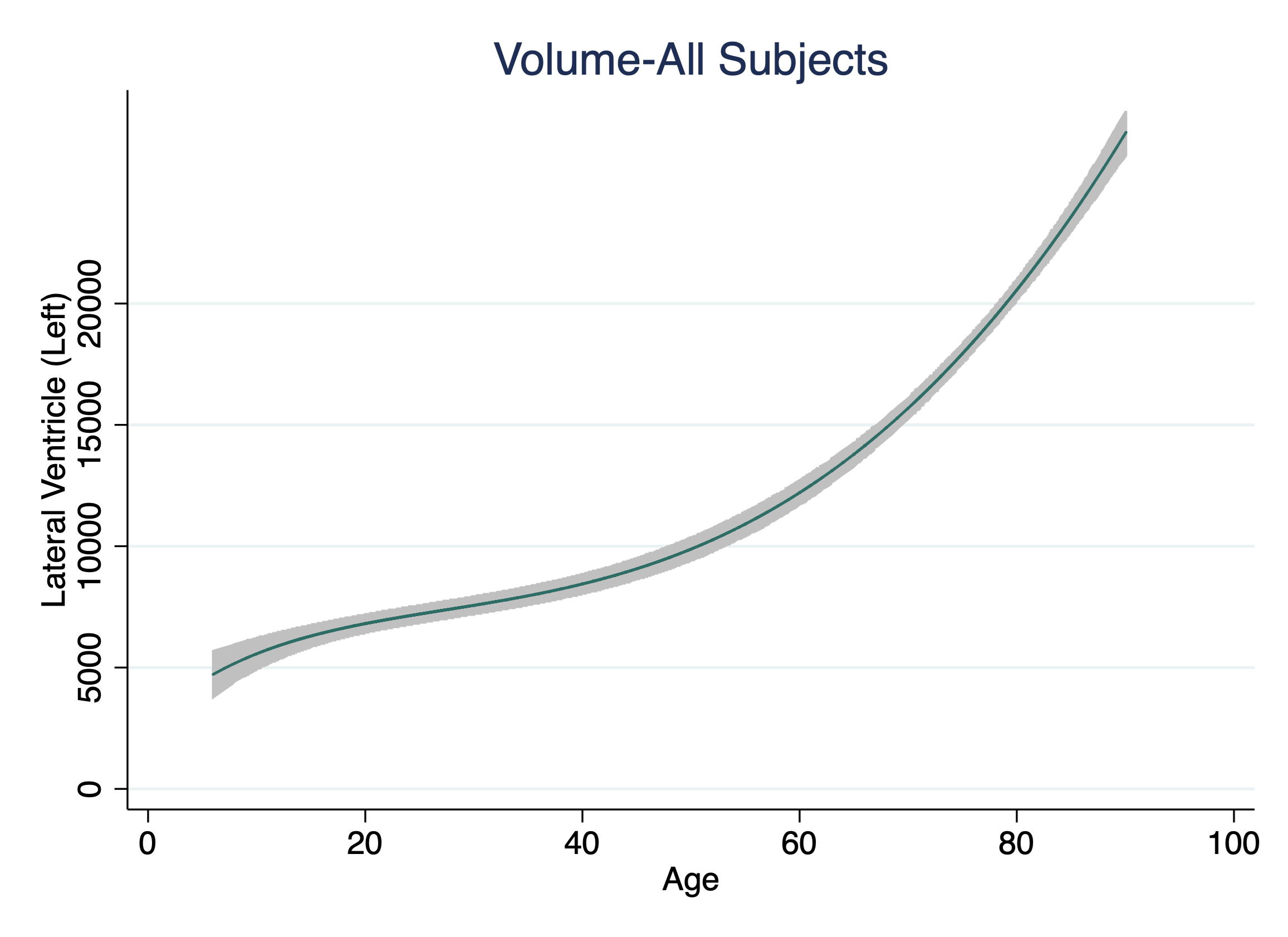

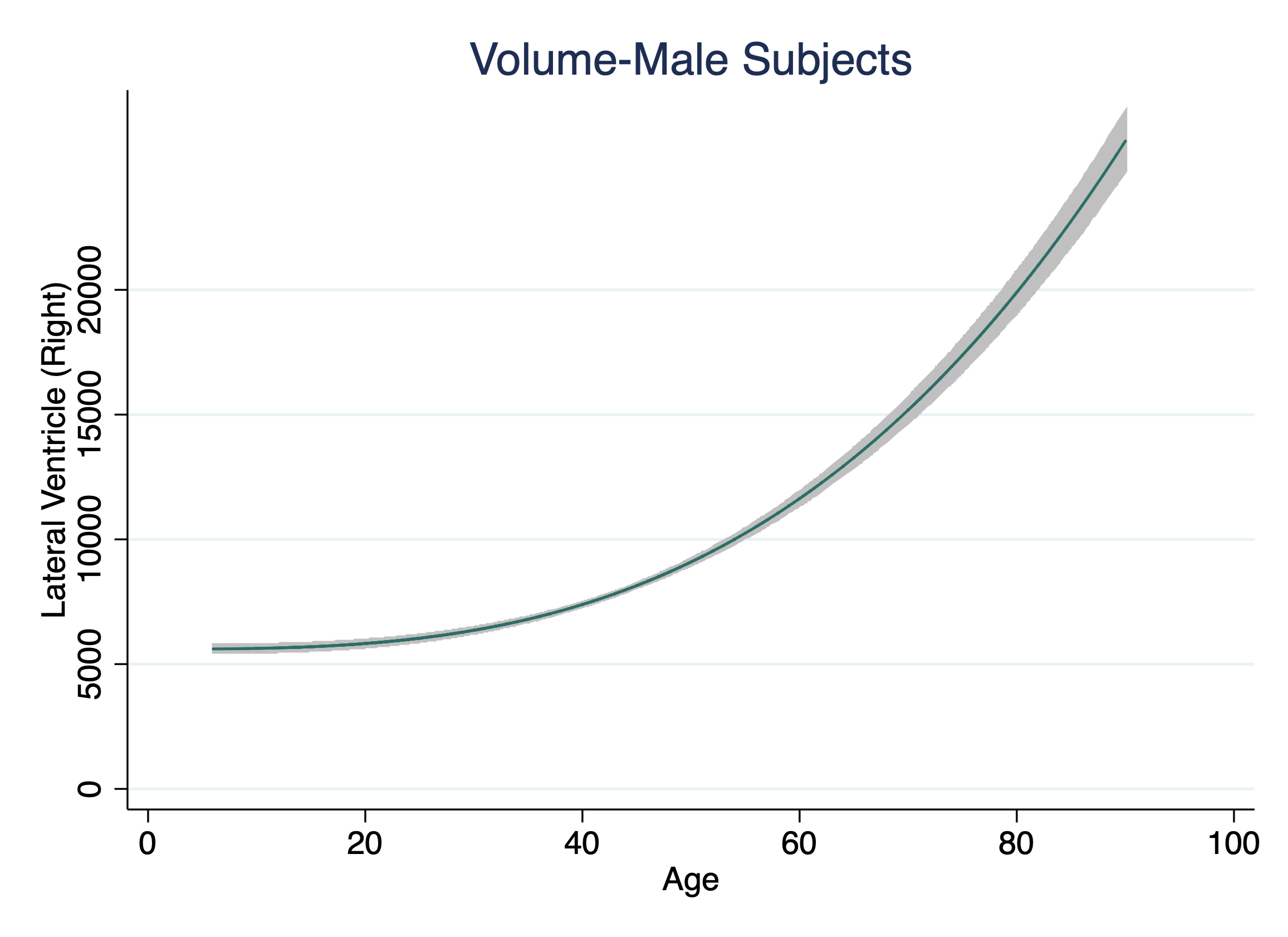

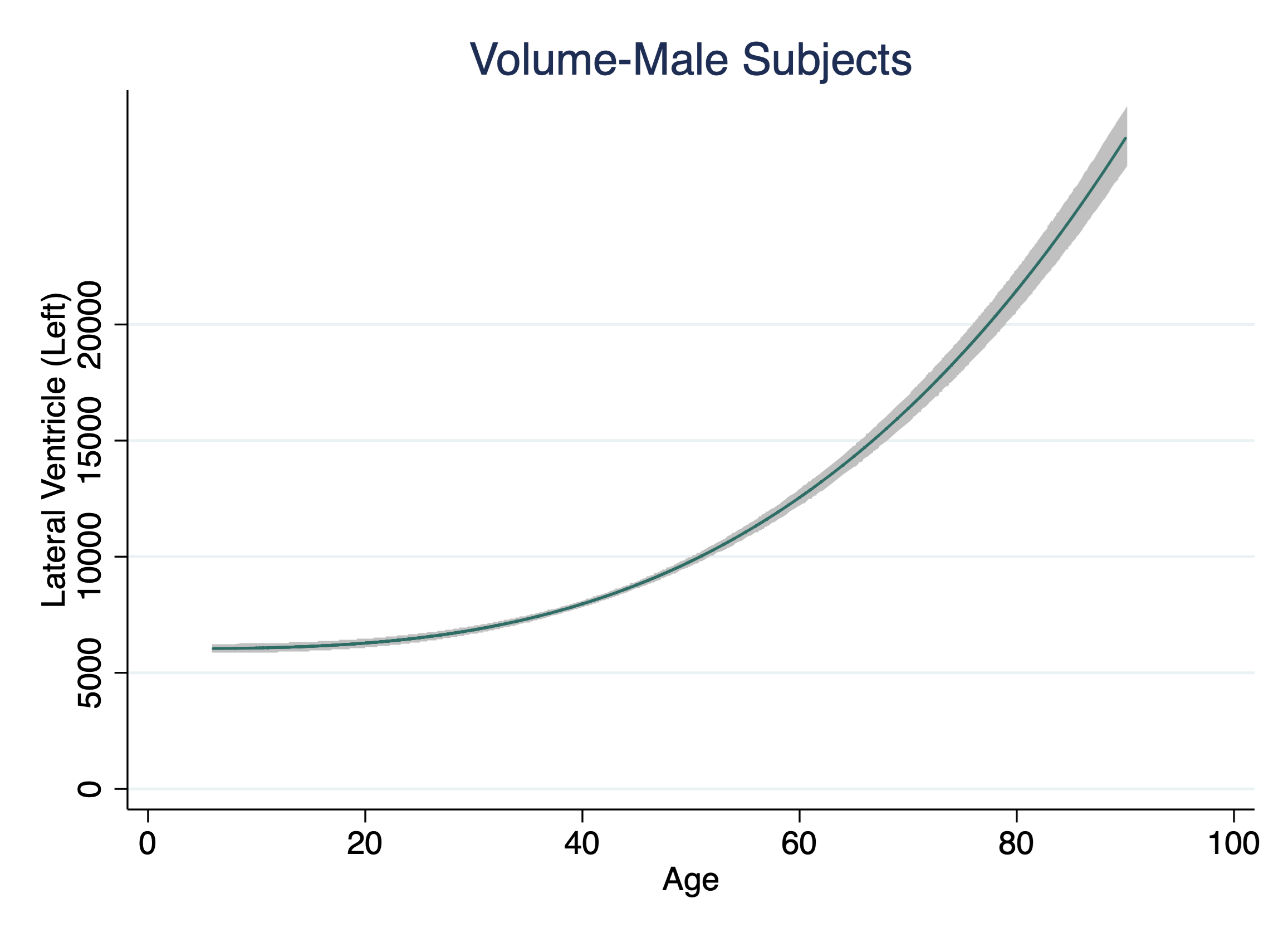

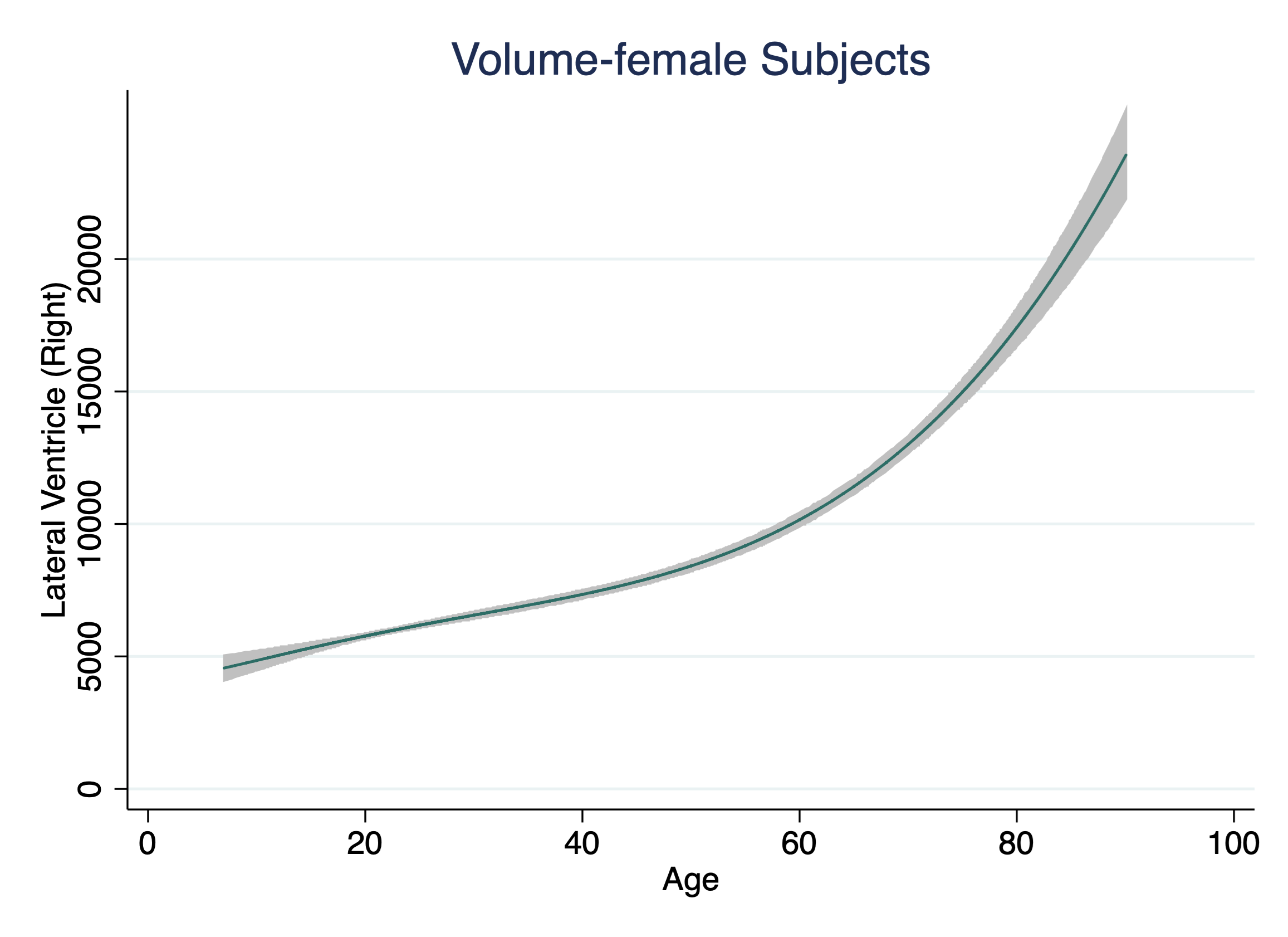

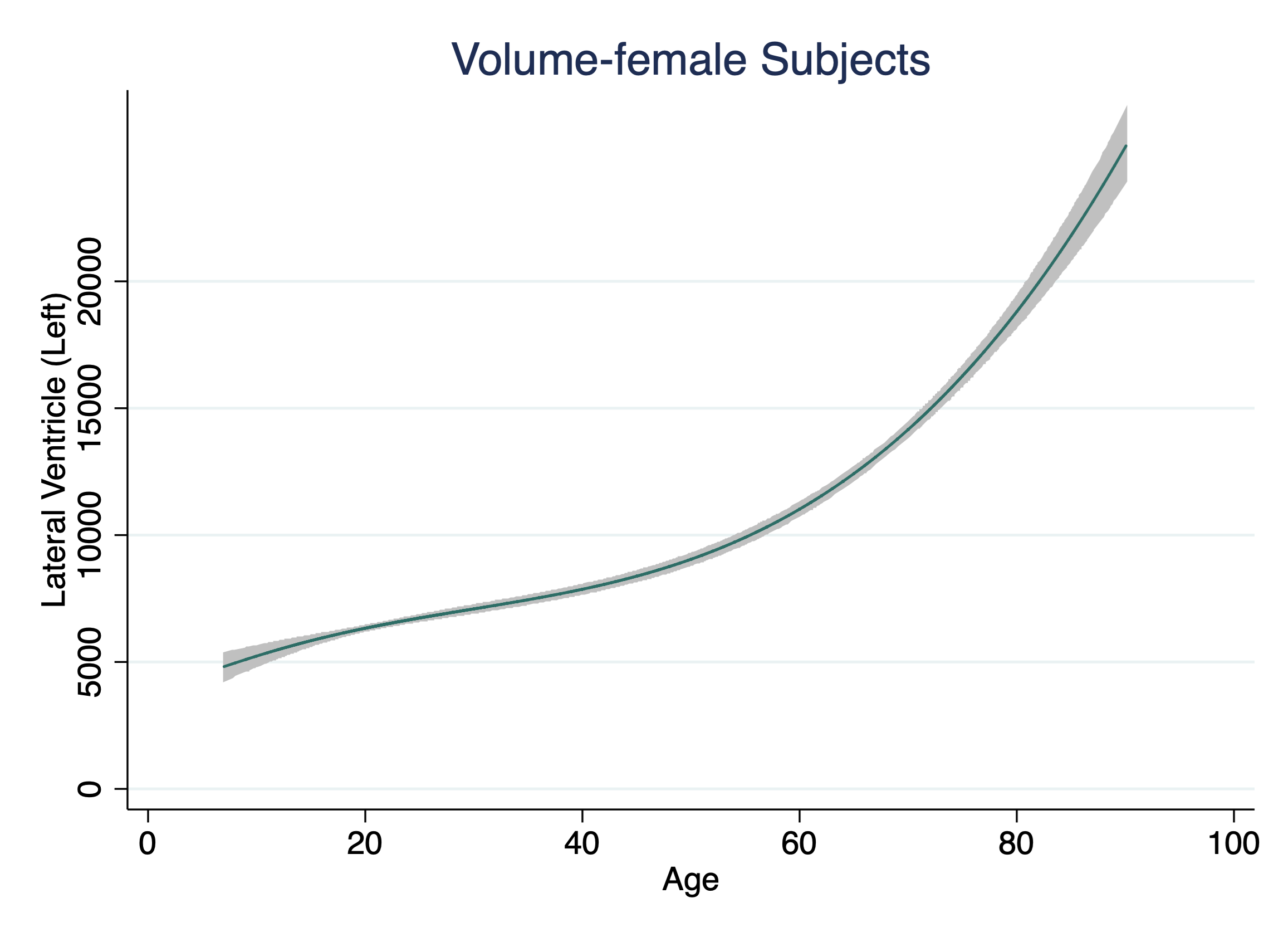


| **Table S1.** Screening Process and Eligibility Criteria, Scanner, Image Acquisition Parameters and Image Segmentation Software | | | | | |
| --- | --- | --- | --- | --- | --- |
| **Sample** | **Screening Process** | **Eligibility Criteria** | **Magnet strength/ Scanner Vendor** | **Acquisition parameters** | **Freesurfer version** |
| **ADHD-NF** | KSADS | No head trauma, no neurological and psychiatric history, no lifetime alcohol or substance abuse, no previous or current use of psychotropic medication, IQ>75. | 3T Siemens Tim Trio | T1-weighted 3D MPRAGE; TR/TE/TI/FA=2300ms/3030ms/900ms/9^o^;  Image matrix= 256 × 256; 192 sagittal slices; voxel size=1mm^3^ | 5.3 |
| **AMC** | Personal Interview | No head trauma, no medical, neurological or psychiatric history, no lifetime alcohol or substance abuse, no previous or current use of psychotropic medication, IQ>75. No history of any psychiatric disorders in 1st degree family members. | 3T Philips Intera | T1-weighted 3D MPRAGE; TE range= 3.5-4.6ms, TR range= 9-9.663ms, FA= | 5.1 |
| **Barcelona 1.5T** | KSADS | No head trauma, no medical, neurological or psychiatric history, no lifetime alcohol or substance abuse, no previous or current use of psychotropic medication, IQ>75. No history of any psychiatric disorders in 1st degree family members. | 1.5 T General Electric Signa | T1-weighted; image matrix = 256 x 256; 128 slices; voxel size 1 x 1 x 1 mm^3^. | 5.3 |
| **Barcelona 3T** | KSADS | No head trauma, no medical, neurological or psychiatric history, no lifetime alcohol or substance abuse, no previous or current use of psychotropic medication, IQ>75. No history of any psychiatric disorders in 1st degree family members. | 3 T Siemens MAGNETOM TIM Trio | T1-weighted; image matrix = 256 x 256; 240 slices; voxel size 1 x 1 x 1 mm^3^. | 5.3 |
| **Betula** | Personal Interview | No head trauma, no medical, neurological or psychiatric history, no lifetime alcohol or substance abuse, no previous or current use of psychotropic medication. | 3T General Electric Discovery  MR750 | T1-weighted MPRAGE; TR/TE/TI/FA=8.1240 ms/3.2000 ms/450ms/ 12°; image matrix = 256x256 | 5.3 |
| **BIG** | Questionnaire for psychiatric history | No head trauma, no medical, neurological or psychiatric history, no lifetime alcohol or substance abuse, no previous or current use of psychotropic medication, IQ>70. No history of any psychiatric disorders in 1st or 2^nd^ degree family members. | 1,5 T Siemens Sonata and Avanto and 3 T Siemens Trio, TimTrio and Skyra | T1-weighted 3D MPRAGE; TR/TE/TI/sagittal slices = 1940-2730 ms/850-110 ms/2.92-4.58 ms; 176-192 sagittal slices; voxel size= 1.0x1.0x1.0 mm^3^ | 5.3 |
| **BIL&GIN** | Personal Interview | No head trauma, no current neurological or psychiatric disorders, no current use of psychotropic medication, IQ>70. | 3T Phillips ACHIEVA | T1 - weighted 3D; TR/TE/TI/FA=20 ms/4.6 ms/800ms/ 10°; turbo field echo factor = 65; sense factor = 2; matrix size = 256x256x180mm 3 ; voxel size= 1.0x1.0x1.0 mm^3^ | 5.3 |
| **Bonn** | Personal interview | No head trauma, no medical, neurological or psychiatric history, no previous or current use of psychotropic medication. | 3T Siemens Trio | TR/TE/FA= 1570-1660ms/2.75-3.42ms/8-9° | NA |
| **BrainSCALE** | Personal interview | No head trauma, no medical, neurological or psychiatric history, no lifetime alcohol or substance abuse, no previous or current use of psychotropic medication. | 1.5T Philips Achieva | T1-weighted 3D SPGR; TR/TE/ FA= 30 ms/4.6 ms/30°; image matrix=256x256; 160**–**180 contiguous coronal slices; voxel size=1 x 1 x 1.2 mm^3^ | 5.1 |
| **BRCATLAS** | Telephone interview | No head trauma, no medical, neurological or psychiatric history, no lifetime alcohol or substance abuse, no mild cognitive impairment, no previous or current use of psychotropic medication, IQ>75. | 3T GE Signa | T1-weighted 3D; TR/TE/TI/FA= 6.9 ms/2.8 ms/650 ms/8°; Image matrix = 256 x 256 x 180mm^3^; voxel size=1mm^3^ |  |
| **CAMH** | SCID | No head trauma, no neurological or psychiatric history, no alcohol or substance abuse preceding 6 months, no previous or current use of psychotropic medication, IQ>75. No history of any psychotic disorders in 1st degree family members. | 1.5 T GE (echospeed) | 124 ﻿Axial inversion recovery–prepared spoiled gradient recall images, 1.5-mm-thick slice acquisition TE/TR/TI/FA=5.3ms/12.3ms/300.0ms/20°. | 5.3 |
| **Cardiff** | MINI | No head trauma, no medical history, including neurological and psychiatric history, no alcohol or substance abuse in the preceding 6 months, no previous or current use of psychotropic medication. | 3T General Electric Signa | T1-weighted 3D FSPGR; TR/TE/TI/FA=7.9ms/3.0ms/450ms/20^o^;  Image matrix= 256 × 192 x 172; voxel size=1mm^3^ | 5.3 |
| **CODE (1-5)** | SCID | No head trauma, no medical, neurological or psychiatric history, no lifetime alcohol or substance abuse, no previous or current use of psychotropic medication, IQ>75. No history of any psychiatric disorders in 1st degree family members. | 3T Siemens Trio (4 CODE sites); 3T Philips Achieva (1 CODE site) | Siemens: T1 mprage, 1mm isotropic voxels, 12 channel head coil, TR=1900ms, TE=2.52ms, 170/192 slices.  Philips: T1 3D-TFE, 1mm isotropic voxels, 8 channel head coil, TR=8.3ms, TE=3.8ms, 170 slices | 5.3 |
| **CEG** | Teacher and parent  Conners' | No head trauma, no medical history, including neurological and psychiatric history, no alcohol or substance abuse in the preceding 6 months, no previous or current use of psychotropic medication. IQ>75 | 3T General Electric Signa | T1-weighted 3D SPGR; TR/TE/ FA=2000ms/30ms/90^o^;  Image matrix= 128 × 128; 43 slices | 5.3 |
| **CIAM** | SCID | No head trauma or psychiatric history, no previous or current use of psychotropic medication, IQ>75. | 3T Siemens Allegra | T1-weighted 3D MPRAGE; TR/TE/TI/FA= 2530 ms/1.53, 3.21, 4.89, 6.57/2.91 ms/ ms/7^o^; image matrix= 256x256; 128 sagittal slices; voxel size= 1.3x1.0x1.3 mm^3^ | 5.3 |
| **CLiNG** | Personal Interview | No head trauma, no medical, neurological or psychiatric history, no lifetime alcohol or substance abuse, no previous or current use of psychotropic medication, IQ>75. No history of any psychiatric disorders in 1st degree family members. | 3T Siemens Tim Trio | T1-weighted 3D MPRAGE; TR/TE/TI/FA=2250 ms/3.26 ms/900 ms/9°; image matrix = 256 x 256; 192 sagittal slices; voxel size= 1 mm^3^ | 5.3 |
| **COMPULS/TS EUROTRAIN** | KSADS | No head trauma, no medical, neurological or psychiatric history, no alcohol or substance abuse preceding 6 months, no previous or current use of psychotropic medication, IQ>75. No history of any psychiatric disorders in 1^st^ or 2^nd^ degree family members. | 3T Siemens Tim Trio and Prisma | T1-weighted 3D MPRAGE; TR/TE/FA=2300 ms/2.98 ms/9°; image matrix = 256 x 256; 176 sagittal slices; voxel size= 1x1x1.2 mm^3^ | 5.3 |
| **Edinburgh** | SCID-NP | No head trauma, no medical, neurological or psychiatric history, no previous or  current use of psychotropic medication, IQ>75. No history of any psychiatric  disorders in 1st and 2nd degree family members. | 1.5T Siemens Magnetom Essenza 1.5T General Electric Signa | T1-weighted MPRAGE; TR/TE/TI/FA =10 ms/4 ms/200 ms/8o; 128  contiguous coronal slices; voxel size=1.25 x 1.25 x 1.20 mm  T1-weighted MPRAGE; TR/TE/TI/FA =10 ms/4 ms/500 ms/8o; image matrix  = 192 x 192; 180 coronal slices; voxel size=1.25 x 1.25 x 1.2 mm3 | 5.3 |
| **ENIGMA-HIV** | MINI | No head trauma, no medical, neurological or psychiatric history, no alcohol or substance abuse preceding 6 months, no previous or current use of psychotropic medication, IQ>75. No mild cognitive impairment | 3T Siemens Allegra | T1-weighted MPRAGE; TR/TE/TI/FA=2400 ms/2.38 ms/1000 ms/ 8°; 162 slices; voxel size= 1 mm^3^ | 5.1 |
| **ENIGMA-OCD (3T OCD control)** | MINI-Plus | No head trauma, no medical, neurological or psychiatric history, no lifetime alcohol or substance abuse, no previous or current use of psychotropic medication, IQ>75. No cognitive impairment | 3T Siemens Allegra | T1-weighted 3D MPRAGE; TR/TE/TI/FA=2300 ms/3.93 ms/ 1100 ms/12^o^; image matrix =256×240; 160 contiguous sagittal slices; voxel size=1.3 x 1 x 1 mm^3^ | 5.3 |
| **ENIGMA-OCD (van den Heuvel 1.5T)** | SCID-I | No medical or psychiatric history | 1.5T Siemens Sonata | T1-weighted 3D MPRAGE; TR/TE/TI/FA=2700 ms/4 ms/ 950 ms/8^o^; image matrix =256×192; 160 slices; voxel size= 1 mm^3^ | 5.3 |
| **ENIGMA-OCD (van den Heuvel 3T)** | SCID-I | No medical or psychiatric history | 3T General Electric Signa | T1-weighted 3D MPRAGE; image matrix =256×256; 172 slices; voxel size= 1x0.977x0.977 mm^3^ | 5.3 |
| **ENIGMA-OCD (Huyser)** | Personal Interview | No head trauma, no medical, neurological or psychiatric history, no lifetime alcohol or substance abuse, no previous or current use of psychotropic medication, IQ>75. | 3T Phillips Intera | T1-weighted 3D MPRAGE; TR/TE/ FA=9.69 ms/4.60 ms/8^o^; image matrix =256×256; 182 slices; voxel size=1 x 1 x 1.2 mm^3^ | 5.3 |
| **ENIGMA-OCD (Mataix-Cols)** | SCID | No head trauma, no neurological or psychiatric history, no lifetime alcohol or substance abuse, no previous or current use of psychotropic medication, IQ>75. | 1.5T General Electric Signa | T1-weighted 3D SPGR; TR/TE/FA= 14.8 ms/ 1.7 ms/ 20º; image matrix=  256 x 256 x 124; voxel size: 0.94 x 0.94 x 1.50 mm | 5.3 |
| **ENIGMA-OCD (Nakao)** | Personal Interview | No head trauma, no neurological or psychiatric history, no lifetime alcohol or substance abuse. | 3T Phillips Achieva | T1-weighted 3D TFE; TR/TE/TI/FA=8.2 ms/3.8ms/1026 ms/8^o^; image matrix =240×240; 190 slices; voxel size=1 mm^3^ | 5.3 |
| **ENIGMA-OCD (IDIBELL)** | SCID-I/NP | No head trauma, no medical, neurological or psychiatric history, no alcohol or substance abuse in the preceding 6 months, no previous or current use of psychotropic medication, IQ>70. No history of any psychiatric disorders in 1st or 2^nd^ degree family members | 1.5 T General Electric Signa | T1-weighted 3D FSPGR; TR/TE/ FA=11.8ms/4.2ms/90^o^;  Image matrix= 256 × 256 x 130; voxel size=1.2mm^3^ | 5.3 |
| **FBIRN** | SCID-I/NP | No head trauma, no medical, neurological or psychiatric history, no alcohol or substance abuse in the preceding 5 years, no previous or current use of psychotropic medication, IQ>75. No history of any Axis-I psychotic disorders in 1st degree family members. | 3T Siemens Tim Trio or General Electric Discovery MR750 | T1-weighted SPGR; TR/TE/TI/FA=2300 ms/2.94 ms/1100 ms/9^o^; image matrix=256×256x160; voxel size=0.86x0.86x1.2mm^3^; sagittal plane acquisition | 5.1 |
| **FIDMAG** | Personal interview; structured interview in part of the sample | No head trauma, no medical, neurological or psychiatric history, no lifetime alcohol or substance abuse, no previous or current use of psychotropic medication, IQ>70. | 1.5 T General Electric Signa | T1-weighted MPRAGE; TR/TE/FA=2000 ms/4 ms/ 9^o^; image matrix=512 x 512; 180 contiguous sagittal slices; voxel size=0.56 x 0.56 x 1 mm3 | 5.3 |
| **GSP** | Structured phone screen and study specific self-report battery and clinical screen | No head trauma, no medical, neurological or psychiatric history, no lifetime alcohol or substance abuse, no current use of psychotropic medication, normal brain anatomy following brain scan. | 3T Siemens Tim Trio | T1-weighted 3D multi-echo MPRAGE; TR/TE/TI/FA =2200 ms/1.54-7 ms/  1100/7 ^o^; voxel size=1.2x1.2x1.2 mm | 4.5 |
| **HUBIN** | SCID-I | No head trauma, no medical, neurological or psychiatric history, no lifetime alcohol or substance abuse, no previous or current use of psychotropic medication, IQ>75. No history of any psychiatric disorders in 1st degree family members. | 1.5 T General Electric Signa | T1-weighted SPGR; TR/TE/FA= 24 ms/6 ms/35 ^o^; 124 coronal slices; voxel size 0.86 x 0.86 x 1.50 mm3. | 5.3 |
| **HMS** | Personal Interview | No head trauma, no medical, neurological or psychiatric history, no lifetime alcohol or substance abuse, no previous or current use of psychotropic medication, IQ>75. No history of any psychiatric disorders in 1st degree family members. | 1.5T Siemens Magnetom Sonata | T1-weighted 3D MPRAGE; TR/TE/TI/FA=1900 ms/4.0 ms/700 ms/15°; image matrix = 256 x 256; 176 consecutive sagittal slices; voxel size=1 mm^3^ | 5.3 |
| **IDIVAL (1) + (2)** | CASH | No head trauma, no medical, neurological or psychiatric history, no lifetime alcohol or substance abuse, no previous or current use of psychotropic medication, IQ>75. No history of any psychiatric disorders in 1st degree family members. | 3T Siemens Alegra, Phillips Achieva  1.5T General Electric Signa | T1-weighted SPGR; TR/TE/FA=24 ms/5 ms/ 5^o^; image matrix=256x192; T1-weighted SPGR; TR/TE/FA=3000 ms/3.9 ms/8^o^; image matrix=256x256;  voxel size=1mm^3^; sagittal plane acquisition | 5.3 |
| **IDIVAL (3)** | Personal Interview | No lifetime history of Axis I psychiatric disorders, no mild cognitive | 3T Phillips Achieva | T1-weighted SPGR; TR/TE/FA=3000 ms/4.6 ms/8^o^; image matrix=321x312; voxel size=1mm^3^; sagittal plane acquisition | 5.3 |
| **IMAGEN** | DAWBA questionnaire clinician interview | No head trauma, no medical, neurological or psychiatric history, no previous or current use of psychotropic medication. IQ>75 | 3T Siemens Verio and TimTrio, Philips Achieva, General Electric Signa Excite, and Signa HDx | T1-weighted 3D MPRAGE; TR/TE/TI/FA=2300ms/3030ms/900ms/9^o^;  Image matrix= 256 × 256;192 sagittal slices; voxel size=1mm^3^ | 5.3 |
| **IMH** | SCID-I/NP | No head trauma, no medical history, neurological or psychiatric history, no lifetime alcohol or substance abuse, as well as no previous or current use of psychotropic medication, IQ>75. No cognitive impairment | 3T Phillips Achieva | T1-weighted 3D MPRAGE; TR/TE/FA= 7.2ms/ 3.8ms/8^o^; image matrix=256 x 256; 180 axial slices; voxel size=0.9mm^3^ | 5.3 |
| **IMpACT** | SCID-I (and SCID-II) | No head trauma, no medical, neurological or psychiatric history, no alcohol or substance abuse in the preceding 6 months, no previous or current use of psychotropic medication, IQ>70. No history of any psychiatric disorders in 1st or 2^nd^ degree family members. | 1.5 T Siemens | T1-weighted 3D-MPRAGE; TR/TE/TI/FA =2730 ms/2.95 ms/1000 ms; 176 consecutive sagittal slices; voxel size= 1 mm^3^ | 3.5 |
| **Indiana 1.5T** | Personal Interview | No head trauma, no medical, neurological or psychiatric history, no lifetime alcohol or substance abuse, no previous or current use of psychotropic medication, IQ>75. | 1.5T General Electric Signa Horizon LX | T1-weighted 3D SPGR; TR/TE/FA=25 ms/3 ms/ 45^o^; image matrix= 256 x  256; 124 contiguous coronal slices | 5.1 |
| **Indiana 3T** | Personal interview  Structured phone screen | No head trauma, no medical, neurological or psychiatric history, no alcohol or substance abuse in the preceding 6 months, no previous or current use of psychotropic medication, IQ>75. | 3T Siemens Skyra | T1-weighted MPRAGE; TR/TE/FA=2300 ms/2.95 ms/ 9^o^; image matrix=256  x 240; 176 contiguous sagittal slices | 5.1 |
| **Johns Hopkins** | Personal Interview | No head trauma, no medical, neurological or psychiatric history, no alcohol or substance abuse preceding 6 months, never prescribed with psychotropic medication, IQ>75. No mild cognitive impairment | 1.5T General Electric Signa | T1-weighted SPGR; TR/TE/FA=35 ms/5 ms/ 45^o^; image matrix=256x256; 124 slices | 5.3 |
| **KaSP** | MINI | No head trauma, no medical, neurological or psychiatric history, no lifetime alcohol or substance abuse, no previous or current use of psychotropic medication, IQ>75. No history of any psychiatric disorders in 1^st^ or 2^nd^ degree family members. | 3T General Electric | T1-weighted SPGR; TR/TI/FA=7.904 ms/450 ms/12 ^o^; image matrix= 256 x 256 mm^3^; 145 sagittal slices ; voxel size=0.934 x 0.934 x 1.2 mm3 | 5.3 |
| **Leiden** | Self-report | No psychiatric or neurological disorders, no use of psychotropic medications | 3T Philips Achieva | T1-weighted 3D SPGR; TR/TE = 9.76 ms/4.59 ms; image matrix=256x256; 160**–**180 contiguous coronal slices; voxel size=0.875x 0.875 x 1.2 mm^3^ | 5.3 |
| **MAS** | Personal interview | No head trauma, no diagnosis of dementia, schizophrenia, bipolar disorder no psychotic symptoms, no neurological disorder, no mild cognitive impairment, IQ>75. | 3T Philips Achieva Quasar Dual | TR/TE = 6.39 ms/2.9 ms; 190 coronal slices; voxel size = 1mm^3^ | 5.3 |
| **MCIC** | SCID, SCID-I/NP, CASH | No head trauma, no medical, neurological or psychiatric history, no lifetime alcohol or substance abuse, no previous or current use of psychotropic medication, IQ>75. | 1.5T Siemens Sonata-3T Siemens Trio | T1-weighted MPRAGE sequence; TR/TE/TI/FA=2530 ms/4.76 ms/1100  ms/20^o^; image matrix=256×256×128 cm; voxel size=0.625 mm^3^ | 5.3 |
| **Melbourne** | SCID-I | No head trauma, no neurological or psychiatric history, no lifetime alcohol or substance abuse, no previous or current use of psychotropic medication. No history of any psychiatric disorders in 1^st^ or 2^nd^ degree family members. | 3T GE Signa Excite | 3D BRAVO sequence 140; TR/TE/FA=7900 ms/3000 ms/13º; FOV=256 mm; matrix=256 x 256 | 5.3 |
| **Meth-CT** | SCID DSM-IV | No head trauma, no medical, neurological or psychiatric history, no lifetime alcohol or substance abuse, no previous or current use of psychotropic medication, IQ>70. | 3T Siemens Allegra | T1-weighted 3D MPRAGE; TR/graded TE/FA=2530 ms/ 1.53, 3.21, 4.89, 6.57 ms/ 7^o^; 160 contiguous sagittal slices; voxel size=1 x 1 *x 1 mm3 | 5.3 |
| **MHRC** | Personal interview | No head trauma, no medical history, including neurological and psychiatric history. No Family History of neurological or psychiatric disorders | 3Т Philips Achieva | T1-weighted TFE; TR/TE/ FA=8.2ms/3.7ms/8^o;^; voxel size=0.83 x 0.83 x 1 mm^3^ | 5.3 |
| **Muenster** | SCID | No head trauma, no medical, neurological or psychiatric history, no lifetime alcohol or substance abuse, no previous or current use of psychotropic medication, IQ>75. | 3T Phillips Intera | T1 weighted TFE: TR/TE/FA= 7.4 ms/3.4 ms/9°; image matrix = 256x204x160mm^3^; voxel size=0.5mm^3^; sagittal plane acquisition | 5.3 |
| **NCNG** | Personal interview | No head trauma, no medical, neurological or psychiatric history, no lifetime alcohol or substance abuse, no mild cognitive impairment, no previous or current use of  psychotropic medication, IQ>84. | 1.5T Siemens Avanto  1.5T Siemens Sonata | T1-weighted 3D MPRAGE; TR/TE/TI/FA= 2400 ms/3.61 ms/1000 ms/8°; image matrix=192x192; 160 sagittal slices; voxel size=1.25 mm^3^  T1-weighted 3D MPRAGE; TR/TE/TI/FA= 2730 ms/3.43 ms/1000 ms/7°; image matrix=256x256; 128 sagittal slices; voxel size=1 mm^3^ | 4.5 |
| **NESDA** | CIDI | No lifetime history of Axis-I diagnoses, no lifetime medical or neurological morbidity including hypertension, no lifetime substance dependence, no substance abuse in the preceding year, no medication use. | 3T Philips Achieva  SENSE-6 to 8 channel head coil | T1-weighted 3D MPRAGE; TR/TE/FA= 9 ms/3.5 ms/8^o^; image matrix=256x256; 170 sagittal slices; voxel size=1mm^3^ | 5.0 |
| **NeuroIMAGE** | KSADS-PL | No head trauma, no mild cognitive impairment, neurological or psychiatric history, no previous or current use of psychotropic medication, IQ>75. No history of any psychiatric disorders in 1st and 2nd degree family members. | 1.5 T Siemens AVANTO (Donders Centre for Cognitive Neuroimaging)  1.5 T Siemens SONATA (VU University Amsterdam) | MPRAGE 176 sagittal slices, repetition time=2,730ms, echo time=2.95ms, voxel size=1.0x1.0x1.0mm, field of view=256 mm | 5.3 |
| **Neuroventure** | DAWBA and BSI | No head trauma, no medical, neurological or psychiatric history, no lifetime alcohol or substance abuse, no previous or current use of psychotropic medication, IQ>75. | 3T SIEMENS TrioTim | T1-weighted 3D MPRAGE; TR/TE/ FA= 2300 ms/2.96 ms/9^o^; image matrix= 256x256; voxel size= 1.0x1.0x1.0 mm^3^ | 5.3 |
| **NTR (1)** | DISC-IV | No head trauma, no medical, neurological or psychiatric history, no lifetime alcohol or substance abuse, no mild cognitive impairment, no previous or current use of psychotropic medication, IQ>75. | 1.5T Siemens Sonata | T1-weighted 3D MPRAGE; TR/TE/TI/FA=1900 ms/3.93 ms/1100 ms/ 15°; image matrix=256 x 224; 160 sagittal slices; voxel size=1 mm^3^ | 5.1 |
| **NTR (2)** | MINI, BDI, STAI, STAS, YBOCS | No head trauma, no previous or current use of psychotropic medication, normal IQ. | 3T Philips Intera | T1-weighted 3D MPRAGE; TR/TE/FA=9.64 ms/4.60 ms/8 ^o^; image matrix=256 x 256; 182 coronal slices; voxel size=1 x1x1.2 mm^3^ | 5.1 |
| **NTR (3)** | CIDI, MADRS, BDI, STAI | No current psychiatric disorder, no current use of psychotropic medication, normal IQ. | 1.5 T Siemens Sonata | T1-weighted 3D MPRAGE; TR/TE/TI/FA= 15 ms/7 ms/300 ms/8°; image matrix=256x176; 160 coronal slices; voxel size=1x1x1.5 mm^3^ | 5.1 |
| **NU** | SCID | No head trauma, no medical, neurological or psychiatric history, no lifetime alcohol or substance abuse, no previous or current use of psychotropic medication, IQ>75. No history of any psychiatric disorders in 1st degree family members. | 1.5T SIEMENS Vision | T1-weighted 3D MPRAGE; TR/TE/TI/FA=2200 ms/4.13 ms/766 ms/13°; voxel size =0.8mm^3^; axial plane acquisition. | 5.3 |
| **NUIG** | SCID | No head trauma, no neurological or psychiatric history, no alcohol or substance abuse preceding 6 months, no previous or current use of psychotropic medication, IQ>75. No history of any psychiatric disorders in 1st degree family members. | Siemens Magnetom Symphony 1.5T | 3D, T1-weighted MPRAGE 4 channel head coil, FOV 230mm, TR/TE/: 1140ms/4.38ms, matrix size 256 x 256, interpolated to 512 x 512, yielding an in-plane voxel size of 0.45mm x 0.45mm^2^, slice thickness 0.9mm. | 5.1 |
| **NYU** | SCID-NP for DSM-IV | No head trauma, no medical history, including neurological and psychiatric history, no lifetime alcohol or substance abuse, no previous or current use of psychotropic medication. IQ>75. | 3T Siemens Allegra | T1-weighted 3D MPRAGE; TR/TE/TI/FA=2530ms/3.25ms/1100ms/7^o^ | 5.3 |
| **OATS (1-4)** | Personal interview | No head trauma, no current diagnosis of a psychotic disorder, no neurological disorder, no malignancy (other than skin cancer) or other severe medical comorbidity, no mild cognitive impairment, IQ>75. | 1.5T Philips Gyroscan, Siemens Magnetom Avanto, Siemens Sonata; 3T Philips Achieva Quasar Dual, a | T1-weighted 3D acquisition; TR/TE/TI/FA=15370 ms/3.24 ms/780 ms/8^o^; 144 slices; voxel size=1 x 1 x 1.5 mm^3^ | 5.3 |
| **OLIN** | SCID I | No head trauma, no medical, neurological or psychiatric history, no alcohol or substance abuse preceding 6 months, never prescribed with psychotropic medication, IQ>75. | 3T Siemens Alegra | T1-weighted 3D MPRAGE; TR/TE/TI/FA= 2300 ms/2.91 ms/900 ms/9^o^; image matrix= 256x240x192; 160 sagittal slices; voxel size= 1.0x1.0x1.2 mm^3^ | 5.1 |
| **PING** | Personal interview | No lifetime history of major developmental, psychiatric, or neurological disorders, brain injury, or other medical conditions that affect development. Individuals born  earlier than 36 weeks of gestational age were excluded. | 3T Philips Achieva  3T GE SIGNA  3T Siemens TrioTim  3T Siemens TrioTim  3T General Electric Discovery  MR750 | T1-weighted 3D IR-GRE; TR/TE/TI/FA= 8.1 ms/3.5 ms/640 ms/9^o^ | 5.3 |
| **QTIM** | CIDI | No head trauma, no medical history, neurological and psychiatric history, no alcohol or substance abuse in the preceding 6 months, no antidepressant medication or medication affecting cognition. | 4T Bruckner | T1-weighted 3D MPRAGE: TR/TE/TI/FA = 1500 ms/3.35 ms/ 700 ms/ 8°; image matrix= 256 × 256 × 256 or 256 × 256 × 240; 256 coronal slices; voxel size= 0.9 mm^3^ | 5.1 |
| **Oxford** | KSADS | No head trauma, no medical, neurological or psychiatric history, no lifetime alcohol or substance abuse, no previous or current use of psychotropic medication, IQ>75. | 1.5T Siemens Sonata | T1-weighted 3D MPRAGE; TR/TE =12 ms/5.6 ms; image matrix =256×240x 208 mm^3^; voxel size=1 mm^3^ | 5.3 |
| **Sao Paulo (1)** | SCID | No head trauma, neurological or psychiatric history, no lifetime alcohol or substance abuse. | 1.5T Siemens Espree | T1-weighted 3D MPRAGE; TR/TE/TI/FA=2400 ms/3.65 ms/ 0 ms/8^o^; 160 contiguous sagittal slices; voxel size=1.3 x 1.3x 1.2 mm3 | 5.3 |
| **Sao Paulo (3)** | SCID | No head trauma, neurological or psychiatric history, no lifetime alcohol or substance abuse. IQ>75 | 1.5T General Electric Signa | T1-weighted FSPGR  ; TR/TE/TI/FA=21.7 ms/52 ms /20^o^; 124 axial slices; voxel size= 0.86 x 0.86 x 1.5 mm3 | 5.3 |
| **SCORE** | BPRS | No head trauma, no medical, neurological or psychiatric history, no lifetime history of alcohol or substance abuse, no previous or current use of psychotropic medication, IQ>75. No history of any psychiatric disorders in 1st degree family members. | 3T Siemens Magnetom Verio | T1-weighted 3D-MPRAGE; TR/TE/TI/FA =2000 ms/3.37 ms/1000 ms/8^o^; image matrix=256x256x176; 176 consecutive sagittal slices; voxel size= 1 mm^3^ | 6.0 |
| **SHIP-2** | Personal Interview | No head trauma, no neurological and psychiatric history, no risky alcohol consumption (cut-offs: males: >= 60g alcohol per day, females: >=30 g alcohol per day) in the preceding 30 days, no current use of psychotropic medication. Exclusion of school leavers without degree. Exclusion of strong MRI artifacts and inhomogeneities | 1.5T Siemens Avanto | T1-weighted 3D MPRAGE; TR/TE/ FA=1900ms/3.4ms/15^o^; voxel size=1mm^3^ | 5.1 |
| **SHIP-TREND** | Personal Interview | No head trauma, no neurological and psychiatric history, no risky alcohol consumption (cut-offs: males: >= 60g alcohol per day, females: >=30 g alcohol per day) in the preceding 30 days, no current use of psychotropic medication. Exclusion of school leavers without degree. Exclusion of strong MRI artifacts and inhomogeneities | 1.5T Siemens Avanto | T1-weighted 3D MPRAGE; TR/TE/ FA=1900ms/3.4ms/15^o^; voxel size=1 mm^3^ | 5.1 |
| **Stages-Dep** | SCID-I | No head trauma, no medical, neurological or psychiatric history, no lifetime alcohol or substance abuse, no previous or current use of psychotropic medication, IQ>75. No history of any psychiatric disorders in 1st degree family members. | 3T Phillips Achieva | T1-weighted 3D-MPRAGE; TR/TE/TI/FA =6.7 ms/3.2 ms/200 ms/88^o^; °; image matrix = 288 x 288; 170 consecutive sagittal slices; voxel size= 0.896×0.896×1.2 mm^3^ | 5.1 |
| **Stanford** | SCID | No head trauma, no medical, neurological or psychiatric history, no lifetime alcohol or substance abuse, no previous or current use of psychotropic medication. no mild cognitive impairment. | 1.5T General Electric Signa Excite | T1-weighted SPGR; TR/TE/TI/FA=8.3-10.3 ms/1.7-3.0 ms/300 ms/15^o^; image matrix= 256 x 192; 176 contiguous sagittal slices; voxel size=0.86x0.86x1.5 mm^3^; sagittal plan acquisition | 5.3 |
| **StrokeMRI** | Personal interview | No head trauma, no medical, neurological or psychiatric history, no lifetime alcohol or substance abuse, no previous or current use of psychotropic medication, IQ>75. | 3T General Electric Signa HDxt | T1-weighted FSPGR; TR/TE/TI/FA=7.8 s/2.956 ms/450 ms/12°; 170 slices; voxel size= 1.0x1.0x1.2 mm | 5.3 |
| **Sydney** | SCID | No head trauma, no medical history, neurological or psychiatric history, no alcohol or substance abuse preceding 6 months, as well as no previous or current use of psychotropic medication, IQ>75. | 3T General Electric Discovery MR750 | T1-weighted 3D MPRAGE; TR/TE/FA= 7264ms/ 2784ms/15^o^; image matrix  =256 x 256 x 196; voxel size=0.9mm^3^ | 5.1 |
| **TOP** | PRIME-MD | No head trauma, no organic or other psychotic disorder (ICD codes 290-299), no substance abuse in the preceding 6 months, no previous or current use of psychotropic medication, IQ>75. No history of any psychiatric disorders in 1st degree family members. | 1.5T Siemens Magnetom Sonata | T1-weighted SPGR; TR/TE/TI/FA=2730 ms/3.93 ms/1000 ms/71^o^; voxel size = 1.33x0.94x1mm^3^; sagittal plane acquisition | 5.3 |
| **Tuebingen** | SCID I and II | No head trauma, no medical history, neurological or psychiatric history, no lifetime alcohol or substance abuse as well as no previous or current use of psychotropic medication, IQ>75. No history of any psychiatric disorders in 1st degree family members. | 1.5T Siemens Avanto | T1-weighted 3D MPRAGE; TR/TE/FA= 2250ms/ 3.93ms/8^o^; image matrix  =256 x 256; voxel size=1mm^3^ | 5.3 |
| **UMCU** | CASH | No head trauma, no medical, neurological or psychiatric history, no lifetime alcohol or substance abuse, no previous or current use of psychotropic medication, IQ>75. No history of any psychiatric disorders in 1st degree family members. | 1.5T Philips Intera and Achieva | T1-weighted 3D FFE; TE/TR/FA= 4.6 ms/0 ms**/ 0˚;** 160-180 contiguous coronal slices; voxel size=1x1x1.2 mm^3^ | 5.1 |
| **UNIBA** | SCID-NP | No head trauma, no medical, neurological or psychiatric history, no lifetime alcohol or substance abuse, no previous or current use of psychotropic medication, IQ>75. No history of any psychiatric disorders in 1st degree family members. | 3T General Electric | T1-weighted 3D SPGR; TE/FA = min full/ 6°; image matrix= 256×256 x124 | 5.3 |
| **UPENN** | SCID | No head trauma, no medical history, including neurological and psychiatric history, no alcohol or substance abuse preceding 6 months, no previous or current use of psychotropic medication, IQ>75. No history of any psychiatric disorders in 1st degree family members. | 3T Siemens Tim Trio | T1-weighted 3D MPRAGE; TR/TE/TI/FA=1810 ms/3.51 ms/1100 ms/9^o^; image matrix= 256 × 192;160 axial slices | 5.3 |
| **Yale** | KSADS-PL | No head trauma, neurological or psychiatric history, no alcohol or substance abuse in the preceding 6 months, no previous or current use of psychotropic medication, IQ>75. | 3T General Electric Signa | T1-weighted 3D MPRAGE; image matrix =256×256; voxel size=0.976 x 0.976 x 1 mm3 | 5.3 |


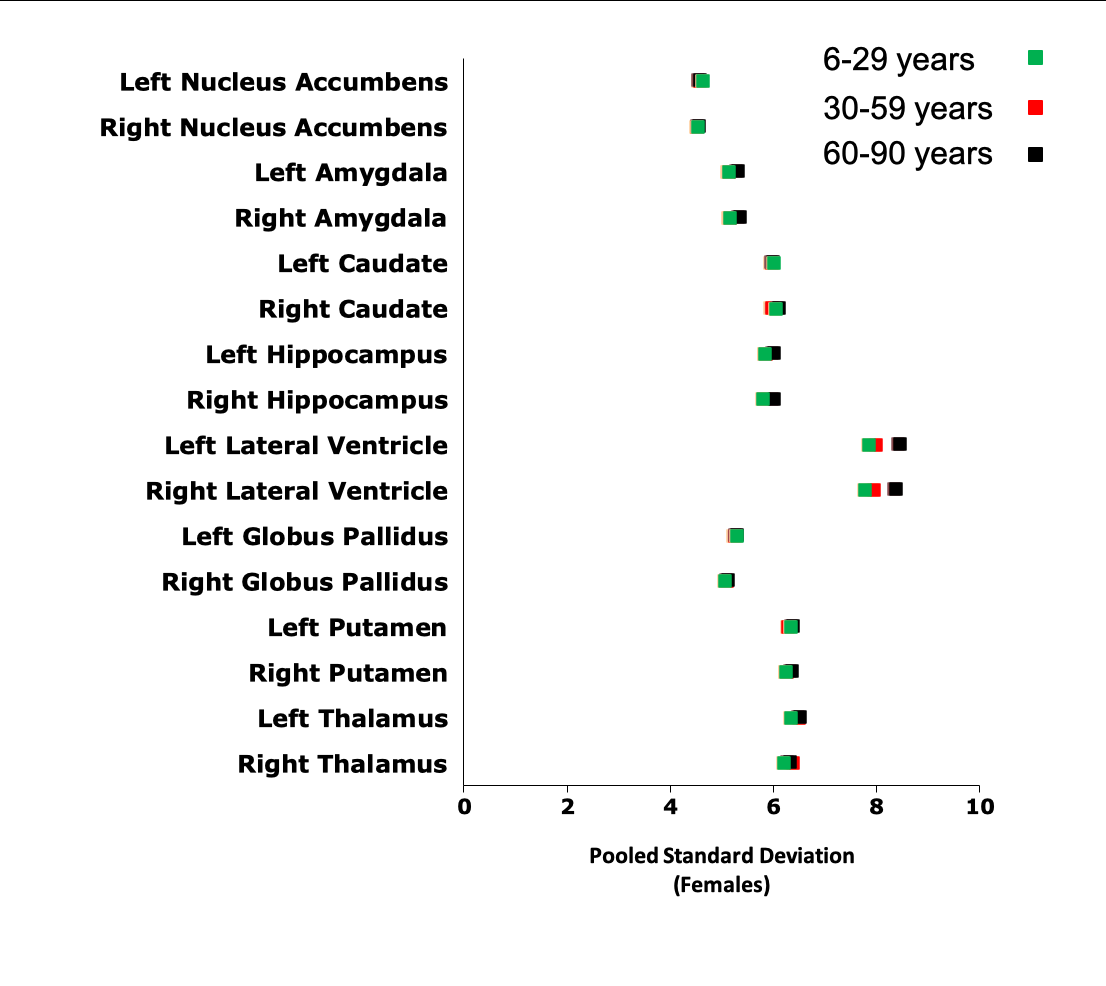


**Females**


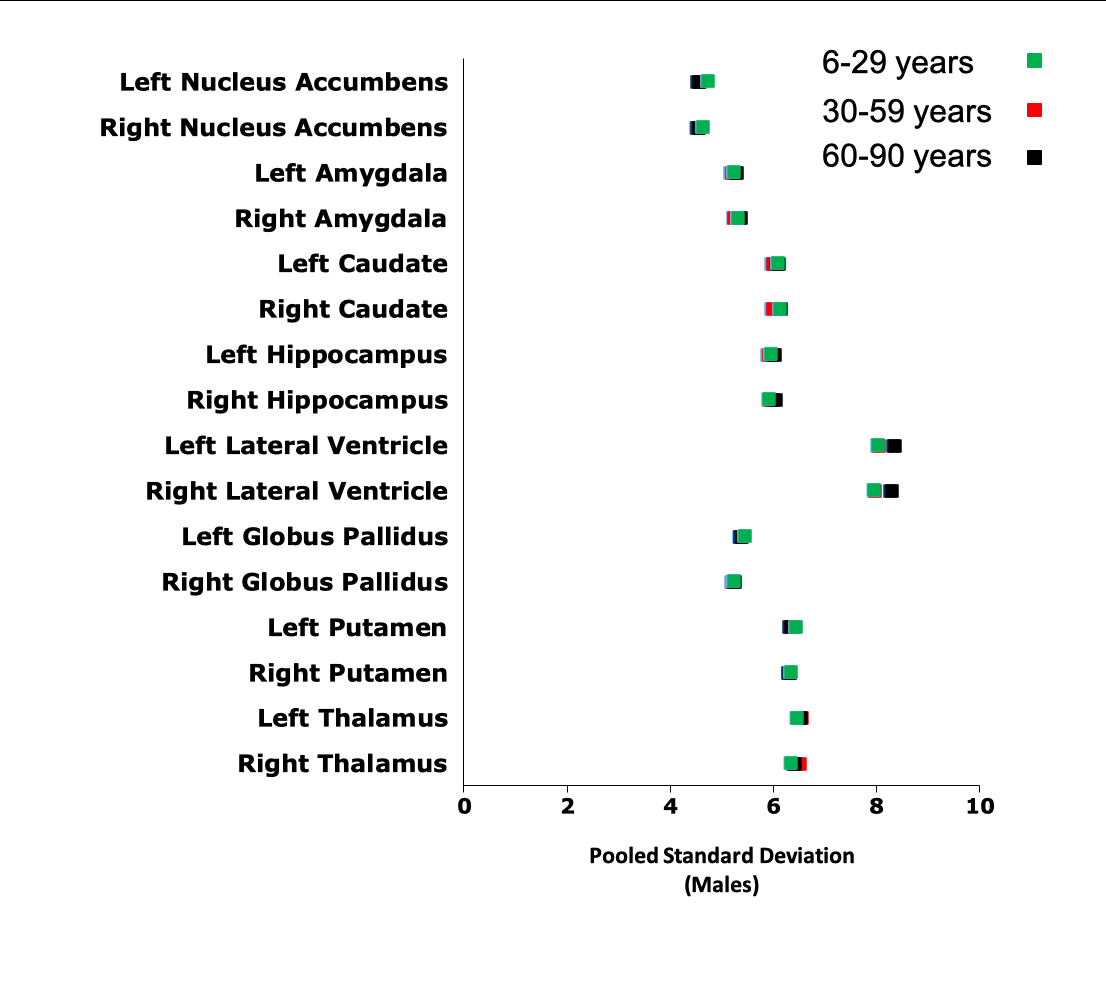


**Males**

**Figure S8. Meta-analysis of the Pooled Standard Deviations of the Volume of each Subcortical Structure Stratified by Sex; there is a significant overlap within each age bracket**

| **Table S2. Age at Maximum Fitted Value for Each Subcortical Volume** | | |
| --- | --- | --- |
| **Region** | **Age (years) at**  **maximum volume**  **Males Only** | **Age (years) at**  **maximum volume**  **Females Only** |
| **Left Lateral Ventricle** | 90 | 90 |
| **Right Lateral Ventricle** | 90 | 90 |
| **Left Thalamus** | 25 | 22 |
| **Right Thalamus** | 16 | 7 |
| **Left Amygdala** | 30 | 7 |
| **Right Amygdala** | 30 | 18 |
| **Left Hippocampus** | 14 | 23 |
| **Right Hippocampus** | 24 | 27 |
| **Left Caudate** | 6 | 7 |
| **Right Caudate** | 6 | 7 |
| **Left Putamen** | 6 | 7 |
| **Right Putamen** | 6 | 7 |
| **Left Nucleus Accumbens** | 6 | 7 |
| **Right Nucleus Accumbens** | 6 | 7 |
| **Left Globus Pallidus** | 6 | 7 |
| **Right Globus Pallidus** | 6 | 7 |

| **Table S3. Variance Explained by Age in Fractional Polynomial Model** | |
| --- | --- |
| **Region** | **R-Squared** |
| **Left Lateral Ventricle** | 0.43 |
| **Right Lateral Ventricle** | 0.43 |
| **Left Thalamus** | 0.26 |
| **Right Thalamus** | 0.29 |
| **Left Caudate** | 0.14 |
| **Right Caudate** | 0.14 |
| **Left Putamen** | 0.35 |
| **Right Putamen** | 0.38 |
| **Left Globus Pallidus** | 0.20 |
| **Right Globus Pallidus** | 0.20 |
| **Left Hippocampus** | 0.22 |
| **Right Hippocampus** | 0.20 |
| **Left Amygdala** | 0.15 |
| **Right Amygdala** | 0.09 |
| **Left Nucleus Accumbens** | 0.23 |
| **Right Nucleus Accumbens** | 0.28 |

| **Table S4. Pearson's Correlation Coefficient Between Age and Subcortical Volumes** | | | | | | | | | |
| --- | --- | --- | --- | --- | --- | --- | --- | --- | --- |
|  | **All Participants** | | | **Males Only** | | | **Females Only** | | |
| **Region** | **6-29 years** | **30-59 years** | **60-90 years** | **6-29 years** | **30-59 years** | **60-90 years** | **6-29 years** | **30-59 years** | **60-90 years** |
| **Left Lateral Ventricle** | 0.099 | 0.256 | 0.498 | 0.106 | 0.275 | 0.494 | 0.092 | 0.237 | 0.525 |
| **Right Lateral Ventricle** | 0.097 | 0.263 | 0.509 | 0.1 | 0.29 | 0.506 | 0.094 | 0.232 | 0.535 |
| **Left Thalamus** | -0.002 | -0.292 | -0.275 | -0.006 | -0.297 | -0.222 | 0.001 | -0.284 | -0.327 |
| **Right Thalamus** | -0.048 | -0.333 | -0.311 | -0.063 | -0.328 | -0.279 | -0.035 | -0.336 | -0.347 |
| **Left Caudate** | -0.161 | -0.224 | -0.041 | -0.171 | -0.237 | -0.05 | -0.152 | -0.21 | -0.034 |
| **Right Caudate** | -0.178 | -0.225 | 0 | -0.194 | -0.249 | -0.018 | -0.164 | -0.197 | 0.017 |
| **Left Putamen** | -0.225 | -0.381 | -0.203 | -0.252 | -0.422 | -0.243 | -0.206 | -0.33 | -0.167 |
| **Right Putamen** | -0.253 | -0.404 | -0.182 | -0.266 | -0.442 | -0.215 | -0.248 | -0.356 | -0.151 |
| **Left Globus Pallidus** | -0.225 | -0.261 | -0.194 | -0.25 | -0.272 | -0.209 | -0.212 | -0.243 | -0.18 |
| **Right Globus Pallidus** | -0.185 | -0.234 | -0.268 | -0.213 | -0.209 | -0.265 | -0.164 | -0.262 | -0.271 |
| **Left Hippocampus** | -0.033 | -0.119 | -0.48 | -0.038 | -0.119 | -0.471 | -0.029 | -0.117 | -0.494 |
| **Right Hippocampus** | -0.029 | -0.1 | -0.472 | -0.04 | -0.114 | -0.478 | -0.02 | -0.082 | -0.472 |
| **Left Amygdala** | -0.017 | -0.11 | -0.379 | 0.005 | -0.128 | -0.356 | -0.038 | -0.08 | -0.398 |
| **Right Amygdala** | -0.016 | -0.086 | -0.288 | -0.008 | -0.105 | -0.253 | -0.025 | -0.054 | -0.32 |
| **Left Nucleus Accumbens** | -0.152 | -0.275 | -0.29 | -0.158 | -0.276 | -0.263 | -0.147 | -0.274 | -0.317 |
| **Right Nucleus Accumbens** | -0.19 | -0.295 | -0.254 | -0.179 | -0.318 | -0.203 | -0.2 | -0.269 | -0.304 |

| **Table S5. Inter-individual Variation in Subcortical Volumes** | | | | | | | |
| --- | --- | --- | --- | --- | --- | --- | --- |
| **Region** | **Inter-individual variation**  **Mean (standard deviation)** | | |  | **P value for sex differences** | | |
|  | **6-29**  **years** | **30-59**  **years** | **60-90**  **years** | **Unadjusted P value for F test** | **6-29 years** | **30-59 years** | **60-90 years** |
| **Left Lateral Ventricle** | 2655 (2686) | 3053 (2791) | 4262 (3922) | **0** | 0 | 0 | 0.57 |
| **Right Lateral Ventricle** | 2456 (2456) | 2835 (2721) | 3982 (3564) | **0** | 0 | 0 | 0.33 |
| **Left Thalamus** | 490 (384) | 537 (422) | 538 (464) | **0.001** | 0 | 0.59 | 0.01 |
| **Right Thalamus** | 441 (353) | 469 (375) | 470 (435) | 0.46 | 0 | **0.0003** | **0.002** |
| **Left Caudate** | 329 (251) | 317 (248) | 330 (299) | 0.26 | **0.001** | 0.87 | 0.003 |
| **Right Caudate** | 335 (258) | 318 (252) | 348 (305) | 0.06 | 0.09 | 0.18 | 0.15 |
| **Left Putamen** | 453 (347) | 441 (349) | 465 (378) | 0.17 | 0.01 | 0.38 | 0.18 |
| **Right Putamen** | 416 (323) | 416 (329) | 438 (369) | 0.70 | 0 | 0.06 | 0.47 |
| **Left Globus Pallidus** | 164 (132) | 166 (131) | 170 (136) | 0.23 | **0.0001** | **0.0002** | 0.02 |
| **Right Globus Pallidus** | 132 (109) | 136 (112) | 136 (114) | 0.34 | 0 | 0.02 | 0.01 |
| **Left Hippocampus** | 274 (224) | 296 (233) | 327 (268) | **0** | 0 | 0.01 | 0.29 |
| **Right Hippocampus** | 267 (213) | 290 (225) | 316 (272) | **0** | **0.0003** | 0.03 | **0.002** |
| **Left Amygdala** | 139 (108) | 147 (113) | 161 (133) | **0.0003** | 0.09 | 0.21 | 0.06 |
| **Right Amygdala** | 144 (112) | 151 (116) | 165 (135) | **0** | 0 | 0.13 | 0.01 |
| **Left Nucleus Accumbens** | 81 (62) | 79 (62) | 87 (67) | 0.06 | **0.002** | 0.13 | 0.31 |
| **Right Nucleus Accumbens** | 74 (57) | 76 (59) | 80 (62) | 0.01 | 0 | 0.10 | 0.005 |

| **Table S6. Centile Values for Subcortical Volumes – All participants** | | | | | | | | | | |
| --- | --- | --- | --- | --- | --- | --- | --- | --- | --- | --- |
| **Region** | **Age** | **C0.4** | **C2** | **C10** | **C25** | **C50** | **C75** | **C90** | **C98** | **C99.6** |
| **Left Lateral Ventricle** | 6 | 461.8519555 | 894.7297485 | 1748.846917 | 2713.851644 | 4196.186199 | 6299.316444 | 9004.812873 | 14321.6895 | 20981.14186 |
|  | 10 | 561.0399805 | 1050.324586 | 1978.673552 | 2996.510817 | 4528.639848 | 6675.224504 | 9427.54924 | 14876.44619 | 21828.67015 |
|  | 15 | 715.4569463 | 1286.0452 | 2315.923872 | 3401.829901 | 4993.420511 | 7185.901198 | 9983.318919 | 15571.07122 | 22871.34034 |
|  | 20 | 888.9811434 | 1553.142386 | 2693.295259 | 3846.94594 | 5491.542958 | 7721.674292 | 10565.22146 | 16350.43842 | 24205.64415 |
|  | 30 | 1215.960024 | 2019.670411 | 3315.683683 | 4566.08221 | 6299.199965 | 8626.091416 | 11627.8828 | 18002.44114 | 27344.38312 |
|  | 40 | 1869.557414 | 2676.35326 | 3999.164773 | 5324.589078 | 7214.595761 | 9766.800728 | 12971.91369 | 19266.30836 | 27361.5774 |
|  | 50 | 2599.426811 | 3450.042558 | 4869.016995 | 6321.753795 | 8421.330955 | 11252.71481 | 14731.1009 | 21190.8785 | 28777.9865 |
|  | 60 | 3449.187985 | 4537.874475 | 6297.124079 | 8044.572191 | 10505.29721 | 13746.15838 | 17655.78692 | 24813.48612 | 33157.23626 |
|  | 70 | 4751.263108 | 6590.900139 | 9184.469438 | 11450.15491 | 14358.16912 | 17980.58459 | 22329.80348 | 30792.95134 | 42051.50959 |
|  | 80 | 7044.493425 | 10147.75941 | 13786.3353 | 16466.68403 | 19530.11093 | 23101.03015 | 27362.52749 | 36184.29895 | 49514.24824 |
|  | 90 | 12153.54983 | 15349.39789 | 18936.89909 | 21522.53713 | 24401.65401 | 27597.12142 | 31132.00635 | 37561.90865 | 45660.63769 |
| **Right Lateral Ventricle** | 6 | 380.6197808 | 805.5153755 | 1625.525122 | 2517.118742 | 3845.271719 | 5701.417357 | 8107.60877 | 13028.3138 | 19651.85246 |
|  | 10 | 474.1462813 | 951.069876 | 1839.135925 | 2783.129691 | 4169.824593 | 6090.051536 | 8568.746614 | 13639.77406 | 20504.13844 |
|  | 15 | 618.8601629 | 1166.728518 | 2144.184888 | 3154.636947 | 4612.813582 | 6607.227291 | 9164.678772 | 14388.69522 | 21491.73496 |
|  | 20 | 786.8394028 | 1404.542059 | 2465.99618 | 3536.609385 | 5057.834092 | 7116.877516 | 9743.972126 | 15111.33364 | 22456.03872 |
|  | 30 | 1179.613725 | 1896.214011 | 3067.482844 | 4217.165621 | 5827.179853 | 7986.688869 | 10729.28423 | 16328.85332 | 24029.03632 |
|  | 40 | 1711.545223 | 2492.125266 | 3749.379342 | 4985.992793 | 6725.376585 | 9056.759659 | 11989.08167 | 17830.38971 | 25556.79432 |
|  | 50 | 2260.744832 | 3127.19154 | 4520.004582 | 5897.299612 | 7845.870061 | 10465.35871 | 13752.36112 | 20232.45603 | 28646.87116 |
|  | 60 | 2953.891102 | 4047.833663 | 5758.404864 | 7407.735288 | 9694.294009 | 12716.62315 | 16466.61884 | 23813.85707 | 33362.62971 |
|  | 70 | 4394.015349 | 6051.171844 | 8403.95084 | 10474.21693 | 13145.00238 | 16480.72118 | 20483.37651 | 28235.19702 | 38455.58347 |
|  | 80 | 6832.900078 | 9478.857911 | 12713.54551 | 15199.05329 | 18110.49757 | 21518.23425 | 25505.37666 | 33336.53503 | 44217.72505 |
|  | 90 | 9234.513271 | 13086.09097 | 17184.8523 | 19968.61653 | 22979.77037 | 26357.1005 | 30304.64271 | 38386.7179 | 50502.38467 |
| **Left**  **Thalamus** | 6 | 6448.50376 | 6728.597024 | 7102.338261 | 7408.470428 | 7764.611095 | 8141.513452 | 8502.459662 | 8998.258009 | 9416.920071 |
|  | 10 | 6428.189665 | 6719.430468 | 7105.499815 | 7420.452633 | 7786.454956 | 8174.46643 | 8547.745517 | 9064.82136 | 9506.650013 |
|  | 15 | 6384.88139 | 6692.588764 | 7095.668131 | 7421.997394 | 7800.257818 | 8202.177071 | 8591.609354 | 9138.543188 | 9615.165506 |
|  | 20 | 6316.372477 | 6643.596263 | 7065.455404 | 7403.403019 | 7793.607483 | 8209.203645 | 8615.449725 | 9196.06732 | 9714.981165 |
|  | 30 | 6198.417059 | 6560.338179 | 7017.002855 | 7377.715911 | 7792.269814 | 8235.783487 | 8675.359671 | 9320.649385 | 9919.834371 |
|  | 40 | 6036.882576 | 6409.032118 | 6878.970882 | 7250.659333 | 7678.662342 | 8137.92618 | 8594.859242 | 9269.331239 | 9899.936234 |
|  | 50 | 5773.215044 | 6145.384764 | 6614.435276 | 6984.92209 | 7411.331138 | 7869.049282 | 8325.012432 | 8999.705223 | 9632.721959 |
|  | 60 | 5459.266532 | 5827.288739 | 6287.203852 | 6647.946239 | 7061.161704 | 7503.306441 | 7943.204662 | 8594.525817 | 9207.052492 |
|  | 70 | 4987.658147 | 5433.320063 | 5937.861947 | 6305.44238 | 6711.13182 | 7143.563793 | 7587.728463 | 8298.024494 | 9048.615606 |
|  | 80 | 4139.749705 | 4910.263826 | 5601.108456 | 6016.459718 | 6432.801279 | 6875.808113 | 7378.176245 | 8385.295707 | 9864.9464 |
|  | 90 | 4029.099738 | 4785.753696 | 5467.660053 | 5879.856622 | 6293.510175 | 6731.461339 | 7222.25562 | 8183.943707 | 9549.455184 |
| **Right Thalamus** | 6 | 6455.230736 | 6705.233687 | 7044.685462 | 7326.370743 | 7656.536558 | 8006.982756 | 8341.878579 | 8798.13088 | 9178.035797 |
|  | 10 | 6357.461758 | 6613.823429 | 6959.33071 | 7244.552735 | 7578.020595 | 7931.910054 | 8270.883624 | 8735.244438 | 9125.187659 |
|  | 15 | 6221.422491 | 6492.503744 | 6850.439762 | 7141.725059 | 7479.975376 | 7838.887917 | 8185.048639 | 8666.857674 | 9081.355456 |
|  | 20 | 6071.786172 | 6376.343104 | 6760.28997 | 7062.72407 | 7408.572292 | 7775.575079 | 8135.376377 | 8655.202232 | 9128.281464 |
|  | 30 | 5768.312664 | 6200.822319 | 6676.811247 | 7016.223675 | 7386.388246 | 7779.361263 | 8184.787245 | 8842.560478 | 9554.04948 |
|  | 40 | 5727.801884 | 6106.505345 | 6552.516721 | 6886.796719 | 7259.759802 | 7654.665836 | 8050.295064 | 8652.218087 | 9244.566369 |
|  | 50 | 5510.825843 | 5866.654705 | 6295.527548 | 6622.150855 | 6988.868539 | 7376.001299 | 7758.99258 | 8327.024043 | 8865.838731 |
|  | 60 | 5219.433621 | 5587.143591 | 6018.162839 | 6339.576415 | 6696.304697 | 7071.594748 | 7444.829002 | 8007.078906 | 8553.633807 |
|  | 70 | 4721.172061 | 5215.991092 | 5716.263885 | 6049.762826 | 6400.068793 | 6767.507265 | 7152.353256 | 7805.816229 | 8562.859893 |
|  | 80 | 3925.856321 | 4741.227943 | 5401.318189 | 5765.740538 | 6113.832697 | 6478.000785 | 6897.146643 | 7776.210641 | 9146.980588 |
|  | 90 | 4325.592123 | 4798.356999 | 5264.653294 | 5569.407249 | 5885.309856 | 6213.522422 | 6555.355611 | 7133.537414 | 7800.683431 |
| **Left**  **Caudate** | 6 | 3062.444473 | 3342.828942 | 3694.127676 | 3965.07933 | 4263.973662 | 4563.805221 | 4837.243022 | 5194.139011 | 5480.980941 |
|  | 10 | 2997.169966 | 3263.567984 | 3599.385243 | 3860.151482 | 4149.86898 | 4443.002387 | 4712.838679 | 5069.119901 | 5359.194221 |
|  | 15 | 2919.034278 | 3170.317462 | 3488.905043 | 3738.064143 | 4017.182666 | 4302.602463 | 4568.527111 | 4925.116052 | 5220.647832 |
|  | 20 | 2854.898809 | 3094.154237 | 3398.636485 | 3638.141205 | 3908.496619 | 4187.897242 | 4451.541035 | 4811.116338 | 5115.148562 |
|  | 30 | 2770.687985 | 2995.501105 | 3281.623733 | 3508.012483 | 3766.651839 | 4039.354285 | 4303.628052 | 4678.107622 | 5010.061483 |
|  | 40 | 2680.122907 | 2902.222648 | 3180.394748 | 3398.761697 | 3648.77063 | 3915.814885 | 4180.726124 | 4571.053205 | 4935.655973 |
|  | 50 | 2577.615944 | 2805.184314 | 3081.835971 | 3294.88237 | 3537.658513 | 3799.63932 | 4066.22448 | 4478.067108 | 4889.323964 |
|  | 60 | 2483.776283 | 2737.571202 | 3029.90839 | 3246.110804 | 3487.75339 | 3748.680147 | 4020.144465 | 4461.230613 | 4936.259811 |
|  | 70 | 2345.795836 | 2669.038423 | 3007.190025 | 3238.551907 | 3485.697271 | 3747.946726 | 4024.279877 | 4494.582262 | 5039.395316 |
|  | 80 | 2050.714375 | 2534.605648 | 2965.381031 | 3224.142669 | 3480.416757 | 3743.578575 | 4024.102995 | 4528.300286 | 5161.296223 |
|  | 90 | 1501.637947 | 2300.196817 | 2906.300091 | 3209.133028 | 3478.591044 | 3744.184799 | 4034.342107 | 4600.075546 | 5386.093034 |
| **Right**  **Caudate** | 6 | 3131.587877 | 3418.211551 | 3778.988004 | 4057.790803 | 4365.049222 | 4672.069802 | 4950.243831 | 5309.615651 | 5594.636684 |
|  | 10 | 3061.66463 | 3330.597454 | 3672.610455 | 3939.67137 | 4236.930016 | 4537.244385 | 4812.384752 | 5172.450474 | 5461.981522 |
|  | 15 | 2976.728978 | 3226.616569 | 3547.775375 | 3801.431696 | 4087.086372 | 4379.631398 | 4651.527515 | 5013.548117 | 5310.224845 |
|  | 20 | 2905.139046 | 3140.192771 | 3444.586917 | 3687.232677 | 3963.34659 | 4249.826963 | 4519.978984 | 4886.32596 | 5192.859308 |
|  | 30 | 2815.278087 | 3032.351181 | 3315.291696 | 3543.504768 | 3807.579378 | 4088.254479 | 4360.885063 | 4745.81455 | 5083.85433 |
|  | 40 | 2715.415653 | 2928.531077 | 3202.886393 | 3423.070444 | 3678.817579 | 3954.327937 | 4228.066051 | 4629.050543 | 4998.833137 |
|  | 50 | 2610.168211 | 2831.69561 | 3108.345003 | 3325.846638 | 3576.611652 | 3848.257474 | 4123.348091 | 4541.851382 | 4949.477429 |
|  | 60 | 2513.738114 | 2765.97143 | 3063.72473 | 3287.926979 | 3540.463355 | 3812.539348 | 4091.972646 | 4533.713112 | 4990.565684 |
|  | 70 | 2362.785988 | 2691.005057 | 3041.211522 | 3284.3071 | 3545.039586 | 3819.658157 | 4103.46203 | 4569.048518 | 5081.176256 |
|  | 80 | 2049.519052 | 2560.378915 | 3018.856583 | 3296.379081 | 3571.217608 | 3850.308876 | 4141.087298 | 4643.041771 | 5239.989229 |
|  | 90 | 1475.181351 | 2329.057819 | 2996.971947 | 3331.434871 | 3627.366797 | 3914.852646 | 4221.558111 | 4796.842482 | 5557.709816 |
| **Left**  **Putamen** | 6 | 5204.98834 | 5593.482684 | 6085.145885 | 6467.154866 | 6890.300998 | 7315.467299 | 7702.81297 | 8206.376223 | 8608.408903 |
|  | 10 | 4975.395582 | 5352.378859 | 5828.528241 | 6198.109864 | 6607.515092 | 7019.326286 | 7395.271952 | 7885.654317 | 8278.898791 |
|  | 15 | 4701.977618 | 5065.415789 | 5523.051122 | 5877.672744 | 6270.468381 | 6666.125269 | 7028.329408 | 7502.99593 | 7886.012393 |
|  | 20 | 4491.320954 | 4845.859851 | 5290.583247 | 5634.447957 | 6015.205265 | 6399.283443 | 6751.968163 | 7216.603184 | 7594.198396 |
|  | 30 | 4316.050793 | 4672.201986 | 5114.268197 | 5453.911629 | 5829.413106 | 6209.29209 | 6560.628081 | 7029.437549 | 7417.1379 |
|  | 40 | 4113.447658 | 4471.700719 | 4909.545247 | 5242.653571 | 5609.779947 | 5982.29866 | 6329.924352 | 6801.581362 | 7200.768273 |
|  | 50 | 3855.017564 | 4216.438019 | 4648.498104 | 4972.457464 | 5327.62257 | 5689.137628 | 6030.381235 | 6503.728615 | 6916.959238 |
|  | 60 | 3610.13325 | 3991.057946 | 4432.752419 | 4757.165084 | 5110.006944 | 5470.459321 | 5815.906935 | 6309.651782 | 6759.235295 |
|  | 70 | 3302.880697 | 3725.934885 | 4196.929769 | 4533.17877 | 4894.802627 | 5265.971692 | 5629.173267 | 6170.234685 | 6692.357483 |
|  | 80 | 2960.492197 | 3460.619305 | 3988.5641 | 4351.051806 | 4734.699278 | 5130.879947 | 5529.834825 | 6159.380872 | 6817.745852 |
|  | 90 | 2583.078064 | 3213.896979 | 3836.520611 | 4241.650715 | 4660.561326 | 5096.428609 | 5553.033632 | 6333.850318 | 7246.797333 |
| **Right**  **Putamen** | 6 | 5201.685061 | 5506.303304 | 5900.024479 | 6214.460525 | 6574.263525 | 6951.288327 | 7311.627036 | 7809.814628 | 8236.769641 |
|  | 10 | 4962.07778 | 5265.791776 | 5656.23927 | 5966.774468 | 6321.2289 | 6692.210015 | 7046.877263 | 7538.19172 | 7960.747163 |
|  | 15 | 4674.61585 | 4977.554615 | 5364.102846 | 5669.775395 | 6017.477985 | 6380.805608 | 6728.311541 | 7211.047388 | 7628.283026 |
|  | 20 | 4438.917145 | 4743.476563 | 5128.7904 | 5431.494097 | 5774.464696 | 6132.181765 | 6474.483761 | 6951.467296 | 7366.012039 |
|  | 30 | 4191.467988 | 4510.777566 | 4906.310897 | 5212.029976 | 5555.034382 | 5911.090797 | 6252.110509 | 6730.72332 | 7152.072224 |
|  | 40 | 3932.242521 | 4265.65662 | 4667.785101 | 4972.329178 | 5309.926219 | 5658.453331 | 5992.833033 | 6466.6868 | 6890.890263 |
|  | 50 | 3667.788925 | 4018.159961 | 4426.928691 | 4728.823946 | 5058.704842 | 5397.25848 | 5723.109266 | 6191.061617 | 6619.305819 |
|  | 60 | 3405.390672 | 3787.00591 | 4214.445007 | 4520.654222 | 4849.668901 | 5185.342392 | 5510.225328 | 5985.448345 | 6433.152777 |
|  | 70 | 3098.144917 | 3538.740811 | 4008.053553 | 4331.941438 | 4673.083313 | 5019.073498 | 5356.826318 | 5863.272905 | 6358.685383 |
|  | 80 | 2735.324398 | 3281.285124 | 3827.106379 | 4186.416403 | 4555.617919 | 4927.580124 | 5294.992944 | 5863.915233 | 6447.448971 |
|  | 90 | 2300.180689 | 3022.094398 | 3691.902183 | 4107.098616 | 4520.488326 | 4933.589051 | 5347.98548 | 6016.417293 | 6743.263513 |
| **Left**  **Pallidum** | 6 | 1458.503664 | 1611.251674 | 1788.615308 | 1919.81557 | 2064.73902 | 2216.944718 | 2368.574106 | 2598.243621 | 2823.351595 |
|  | 10 | 1363.713599 | 1512.856323 | 1686.203542 | 1814.464122 | 1956.050335 | 2104.503175 | 2252.003602 | 2474.473224 | 2691.30013 |
|  | 15 | 1255.16412 | 1399.906568 | 1568.259132 | 1692.807137 | 1830.137574 | 1973.781486 | 2115.993959 | 2329.2965 | 2535.6827 |
|  | 20 | 1172.385469 | 1313.785168 | 1478.205783 | 1599.729759 | 1733.487742 | 1872.976422 | 2010.512688 | 2215.552823 | 2412.416384 |
|  | 30 | 1115.606895 | 1257.752433 | 1422.435614 | 1543.620357 | 1676.282012 | 1813.570332 | 1947.651626 | 2144.872814 | 2331.117827 |
|  | 40 | 1054.480313 | 1199.659105 | 1367.148398 | 1489.811101 | 1623.338768 | 1760.466918 | 1893.14619 | 2085.798415 | 2264.890347 |
|  | 50 | 987.1217384 | 1133.22151 | 1300.717704 | 1422.617421 | 1554.439734 | 1688.68181 | 1817.303437 | 2001.633789 | 2170.340605 |
|  | 60 | 969.3963866 | 1117.802301 | 1286.468297 | 1408.247952 | 1538.935462 | 1670.808454 | 1795.877617 | 1972.763772 | 2132.186411 |
|  | 70 | 909.0797686 | 1065.244477 | 1241.026725 | 1366.875595 | 1500.875556 | 1634.865125 | 1760.685287 | 1936.397221 | 2092.47575 |
|  | 80 | 774.5969852 | 953.5761669 | 1152.000816 | 1292.326137 | 1440.218916 | 1586.494933 | 1722.327375 | 1909.472227 | 2073.221757 |
|  | 90 | 620.1108492 | 846.670487 | 1092.006358 | 1261.986995 | 1438.377424 | 1610.26036 | 1767.661493 | 1981.138297 | 2164.840864 |
| **Right Pallidum** | 6 | 1430.319015 | 1546.368157 | 1683.301775 | 1786.445139 | 1902.665246 | 2027.888319 | 2156.46496 | 2359.633338 | 2569.73742 |
|  | 10 | 1351.333779 | 1464.769242 | 1597.771035 | 1697.412606 | 1809.260214 | 1929.442071 | 2052.662477 | 2247.270675 | 2448.561759 |
|  | 15 | 1264.133715 | 1374.997139 | 1503.91187 | 1599.815764 | 1706.939408 | 1821.640831 | 1939.025219 | 2124.30818 | 2316.015672 |
|  | 20 | 1202.127204 | 1312.430636 | 1439.586728 | 1533.491553 | 1637.848042 | 1749.182224 | 1862.907629 | 2042.318409 | 2228.026818 |
|  | 30 | 1153.202808 | 1269.594139 | 1401.289323 | 1497.035797 | 1602.295983 | 1713.746728 | 1827.158457 | 2005.908123 | 2191.138462 |
|  | 40 | 1096.583292 | 1219.211953 | 1355.132259 | 1452.273642 | 1557.831519 | 1668.703822 | 1781.089242 | 1958.096554 | 2141.801589 |
|  | 50 | 1032.87303 | 1161.945104 | 1301.760572 | 1399.827594 | 1505.061579 | 1614.657754 | 1725.31127 | 1899.512971 | 2080.663029 |
|  | 60 | 989.1892857 | 1128.731177 | 1276.045783 | 1377.259981 | 1484.408256 | 1594.99776 | 1706.207666 | 1881.258244 | 2063.737278 |
|  | 70 | 926.3869121 | 1075.729663 | 1228.867945 | 1331.709375 | 1438.995814 | 1548.677408 | 1658.529008 | 1831.471537 | 2012.286645 |
|  | 80 | 843.9560222 | 1001.755315 | 1158.308984 | 1260.819304 | 1366.077297 | 1472.605175 | 1578.865019 | 1746.242747 | 1921.851212 |
|  | 90 | 790.954227 | 965.4476954 | 1132.277341 | 1238.502228 | 1345.719145 | 1453.078715 | 1559.733048 | 1727.889394 | 1905.029392 |
| **Left**  **Hippocampus** | 6 | 3209.830065 | 3538.235306 | 3860.271045 | 4069.194823 | 4280.565316 | 4488.822548 | 4688.706726 | 4985.63953 | 5274.968176 |
|  | 10 | 3210.781104 | 3535.618644 | 3858.769005 | 4070.493114 | 4285.469859 | 4496.831244 | 4698.134298 | 4992.826859 | 5274.371985 |
|  | 15 | 3204.041788 | 3525.414024 | 3850.301773 | 4065.488114 | 4284.80481 | 4499.842245 | 4702.79512 | 4994.901829 | 5267.686082 |
|  | 20 | 3179.085091 | 3498.384397 | 3825.710921 | 4044.523928 | 4268.191285 | 4486.855718 | 4691.470516 | 4981.382614 | 5246.49005 |
|  | 30 | 3116.95536 | 3440.130027 | 3778.684837 | 4008.157873 | 4243.529345 | 4472.151249 | 4682.69826 | 4972.730913 | 5228.223952 |
|  | 40 | 3054.217157 | 3380.625619 | 3727.940243 | 3965.573035 | 4209.627824 | 4445.109407 | 4658.926459 | 4946.468001 | 5191.896344 |
|  | 50 | 2993.277262 | 3320.904668 | 3673.317833 | 3915.895667 | 4164.967921 | 4403.707643 | 4617.789538 | 4899.847028 | 5134.268672 |
|  | 60 | 2841.214691 | 3181.383599 | 3547.911866 | 3800.147789 | 4058.161647 | 4303.396756 | 4520.547021 | 4801.278181 | 5029.119615 |
|  | 70 | 2504.917523 | 2882.976389 | 3283.669914 | 3556.116592 | 3831.835581 | 4090.510785 | 4316.162673 | 4602.226822 | 4829.128277 |
|  | 80 | 2052.820835 | 2483.900327 | 2924.949439 | 3217.73673 | 3509.024316 | 3777.747012 | 4008.308376 | 4294.927219 | 4517.400869 |
|  | 90 | 1606.310368 | 2089.462455 | 2565.354839 | 2871.709562 | 3170.282446 | 3440.70951 | 3668.917794 | 3947.469996 | 4159.528416 |
| **Right**  **Hippocampus** | 6 | 3446.052089 | 3661.469099 | 3914.272171 | 4101.584866 | 4306.117797 | 4514.95312 | 4714.07258 | 4995.609279 | 5248.034315 |
|  | 10 | 3430.061551 | 3654.181825 | 3915.01952 | 4106.961027 | 4315.506639 | 4527.593007 | 4729.266961 | 5013.87968 | 5268.787955 |
|  | 15 | 3400.413931 | 3635.745877 | 3906.611899 | 4104.132285 | 4317.34664 | 4533.065944 | 4737.486876 | 5025.297219 | 5282.736403 |
|  | 20 | 3353.27216 | 3599.809079 | 3880.205856 | 4082.713039 | 4299.820227 | 4518.303919 | 4724.612246 | 5014.396144 | 5273.28325 |
|  | 30 | 3271.2435 | 3544.823485 | 3847.733458 | 4061.902664 | 4288.148336 | 4513.254065 | 4724.2578 | 5019.246109 | 5282.195413 |
|  | 40 | 3174.65865 | 3480.784859 | 3809.146713 | 4035.75661 | 4271.268243 | 4502.736198 | 4718.042132 | 5017.65387 | 5284.225228 |
|  | 50 | 3041.693556 | 3390.229554 | 3749.918281 | 3991.244541 | 4237.488784 | 4476.270575 | 4696.566374 | 5001.68405 | 5272.711028 |
|  | 60 | 2822.968991 | 3225.192331 | 3620.588534 | 3877.031721 | 4133.208983 | 4377.879753 | 4601.572589 | 4909.830406 | 5183.194139 |
|  | 70 | 2484.861213 | 2949.994172 | 3380.828859 | 3648.980726 | 3910.27181 | 4155.52208 | 4377.468667 | 4681.605368 | 4950.823021 |
|  | 80 | 2067.789722 | 2599.301759 | 3063.606166 | 3339.139281 | 3600.034278 | 3840.139906 | 4054.985028 | 4347.601859 | 4606.134361 |
|  | 90 | 1651.118602 | 2230.699887 | 2724.145218 | 3003.32851 | 3259.569845 | 3490.409728 | 3694.49297 | 3970.734402 | 4214.403041 |
| **Left**  **Amygdala** | 6 | 1136.194941 | 1245.505854 | 1378.141267 | 1478.892501 | 1590.518801 | 1705.278868 | 1814.548737 | 1967.468687 | 2102.114189 |
|  | 10 | 1145.495335 | 1254.704083 | 1387.494147 | 1488.601099 | 1600.913871 | 1716.763379 | 1827.491075 | 1983.226191 | 2121.154711 |
|  | 15 | 1151.234848 | 1259.903032 | 1392.362156 | 1493.503692 | 1606.215009 | 1722.957055 | 1835.073573 | 1993.764461 | 2135.365634 |
|  | 20 | 1146.433235 | 1254.208896 | 1385.828052 | 1486.561424 | 1599.129817 | 1716.15989 | 1829.054396 | 1989.810281 | 2134.28961 |
|  | 30 | 1134.664629 | 1244.099771 | 1377.587714 | 1479.747464 | 1594.073108 | 1713.314842 | 1828.905094 | 1994.741395 | 2145.231288 |
|  | 40 | 1110.546039 | 1225.60089 | 1364.969706 | 1471.040296 | 1589.332113 | 1712.48395 | 1831.864639 | 2003.47448 | 2159.778552 |
|  | 50 | 1064.412673 | 1187.668016 | 1334.29559 | 1444.16195 | 1565.218829 | 1689.931928 | 1809.841866 | 1980.970472 | 2135.89525 |
|  | 60 | 978.0071728 | 1117.699151 | 1276.606425 | 1391.278178 | 1513.974521 | 1637.015781 | 1752.640716 | 1913.907877 | 2056.722738 |
|  | 70 | 804.9612075 | 992.0285441 | 1186.27888 | 1317.183696 | 1450.609368 | 1578.90727 | 1695.431918 | 1852.715576 | 1987.847409 |
|  | 80 | 528.6658953 | 785.5743947 | 1031.81151 | 1182.634586 | 1326.606796 | 1457.978731 | 1572.700776 | 1722.256726 | 1846.963958 |
|  | 90 | 349.0550664 | 597.7200569 | 875.8065097 | 1040.420254 | 1188.660238 | 1317.212917 | 1425.342563 | 1561.936578 | 1672.968571 |
| **Right**  **Amygdala** | 6 | 1220.487846 | 1317.097432 | 1440.797638 | 1538.944478 | 1650.948976 | 1768.424624 | 1881.232101 | 2038.707716 | 2175.562942 |
|  | 10 | 1219.150822 | 1317.007335 | 1442.182282 | 1541.502128 | 1654.978032 | 1774.29706 | 1889.300898 | 2050.750372 | 2192.072155 |
|  | 15 | 1214.596914 | 1313.894115 | 1440.719056 | 1541.328985 | 1656.434586 | 1777.849467 | 1895.433453 | 2061.724331 | 2208.668469 |
|  | 20 | 1204.050887 | 1304.548272 | 1432.641149 | 1534.190848 | 1650.492589 | 1773.532745 | 1893.255987 | 2063.845814 | 2216.069758 |
|  | 30 | 1190.954062 | 1294.169965 | 1425.010481 | 1528.498095 | 1647.202815 | 1773.52155 | 1897.659777 | 2077.419596 | 2241.292455 |
|  | 40 | 1176.759591 | 1284.09693 | 1418.495667 | 1523.87603 | 1644.290406 | 1772.524127 | 1899.244328 | 2084.924906 | 2257.156202 |
|  | 50 | 1137.449503 | 1254.135348 | 1396.330633 | 1505.323287 | 1627.889628 | 1756.918557 | 1883.666513 | 2069.186342 | 2241.87933 |
|  | 60 | 1054.637489 | 1190.271077 | 1347.901053 | 1463.977201 | 1590.690879 | 1720.888435 | 1846.624477 | 2028.334004 | 2196.063224 |
|  | 70 | 915.2287776 | 1087.008763 | 1272.241839 | 1400.841004 | 1535.550589 | 1669.518662 | 1796.030694 | 1975.870694 | 2140.078891 |
|  | 80 | 714.477325 | 940.826773 | 1162.31484 | 1305.125847 | 1447.826302 | 1584.999943 | 1711.923799 | 1890.273587 | 2052.467631 |
|  | 90 | 507.6301665 | 783.5132412 | 1043.730732 | 1199.666132 | 1347.972067 | 1485.851758 | 1611.245516 | 1786.398716 | 1946.160484 |
| **Left Nucleus**  **Accumbens** | 6 | 509.3369909 | 560.4184841 | 629.4269536 | 686.7857237 | 754.6437111 | 828.0265489 | 900.071784 | 1002.300364 | 1091.950146 |
|  | 10 | 458.3792538 | 508.751442 | 576.4644938 | 632.5205011 | 698.671797 | 770.128433 | 840.3183835 | 940.162862 | 1028.106402 |
|  | 15 | 406.0645393 | 456.1769606 | 522.9965844 | 577.9434024 | 642.5049003 | 712.0848747 | 780.4457886 | 877.9926835 | 964.423105 |
|  | 20 | 373.0829286 | 424.3734726 | 491.9933426 | 547.0725277 | 611.3761519 | 680.4094219 | 748.1820008 | 845.1494629 | 931.5865044 |
|  | 30 | 349.2403228 | 404.1216383 | 474.1379547 | 529.6124416 | 593.1225965 | 660.3665002 | 725.9519993 | 819.8330895 | 904.1453579 |
|  | 40 | 309.3652364 | 369.4266387 | 444.2740041 | 502.3737539 | 567.8927645 | 636.4631127 | 702.8809435 | 797.6673148 | 882.8753486 |
|  | 50 | 272.2874076 | 333.6828459 | 409.4274302 | 467.585028 | 532.414372 | 599.302284 | 663.0643628 | 752.2097612 | 830.4676983 |
|  | 60 | 252.9652083 | 313.6505376 | 388.2519102 | 445.179573 | 507.9983186 | 571.7605516 | 631.2414436 | 711.8677256 | 780.0074334 |
|  | 70 | 180.7827393 | 251.5697303 | 337.9710893 | 403.2580129 | 474.4331524 | 545.465816 | 610.3700521 | 695.9001277 | 765.7832906 |
|  | 80 | 96.70762039 | 176.5210051 | 275.8783393 | 349.4228883 | 427.8446346 | 504.271982 | 572.4526354 | 659.7782337 | 728.9196727 |
|  | 90 | 56.14584401 | 128.7813802 | 231.0222546 | 306.3907411 | 384.8817681 | 459.4456231 | 524.3976837 | 605.4521442 | 667.9165314 |
| **Right Nucleus**  **Accumbens** | 6 | 520.2835117 | 572.0952781 | 639.8024443 | 694.2143861 | 756.5557293 | 821.6701392 | 883.4405385 | 967.7234992 | 1038.663622 |
|  | 10 | 492.3327807 | 543.6601531 | 610.6250437 | 664.3649907 | 725.8748013 | 790.075919 | 850.9619036 | 934.0476094 | 1004.017239 |
|  | 15 | 459.7529342 | 510.582179 | 576.7524395 | 629.7545035 | 690.3376418 | 753.5112302 | 813.397383 | 895.1279804 | 964.0019979 |
|  | 20 | 432.4273376 | 483.1945903 | 549.1341752 | 601.8479698 | 662.0174585 | 724.6988756 | 784.0942044 | 865.1682407 | 933.5398196 |
|  | 30 | 393.5582945 | 445.8903059 | 513.535994 | 567.3897757 | 628.6809067 | 692.404365 | 752.7411936 | 835.1414278 | 904.7536324 |
|  | 40 | 356.5435666 | 411.0184329 | 480.9478296 | 536.2810783 | 598.9709491 | 663.9223504 | 725.2985028 | 809.0611655 | 879.8758114 |
|  | 50 | 316.2260633 | 373.6368969 | 446.3749232 | 503.2652046 | 567.1363895 | 632.798314 | 694.4904212 | 778.3128495 | 848.9814379 |
|  | 60 | 273.4829908 | 335.4499014 | 412.2238826 | 471.133594 | 536.3047312 | 602.4507556 | 663.9887162 | 746.9097447 | 816.38217 |
|  | 70 | 227.8520501 | 296.662306 | 379.0444006 | 440.5399904 | 507.2118142 | 573.7365293 | 634.8331285 | 716.2724036 | 783.9440501 |
|  | 80 | 176.4863346 | 254.1511658 | 343.155199 | 407.1938945 | 474.8592009 | 540.9816308 | 600.7898235 | 679.5289723 | 744.3596607 |
|  | 90 | 125.5274097 | 211.3059302 | 308.0423107 | 374.8989652 | 443.4540063 | 508.8724462 | 567.0588543 | 642.6648769 | 704.3411915 |

| **Table S7. Centile Values for Subcortical Volumes in Males** | | | | | | | | | | |
| --- | --- | --- | --- | --- | --- | --- | --- | --- | --- | --- |
| **Region** | **Age** | **C0.4** | **C2** | **C10** | **C25** | **C50** | **C75** | **C90** | **C98** | **C99.6** |
| **Left**  **Lateral**  **Ventricle** | 6 | 387.0341483 | 747.7458825 | 1529.590799 | 2495.891861 | 4084.389304 | 6438.690321 | 9497.069649 | 15319.11225 | 22083.30252 |
|  | 10 | 469.4135503 | 870.1070923 | 1711.845436 | 2730.007865 | 4380.696222 | 6805.501484 | 9944.139215 | 15927.35755 | 22923.05365 |
|  | 15 | 589.9165877 | 1044.971143 | 1966.624997 | 3052.825239 | 4783.765896 | 7297.842374 | 10536.60367 | 16720.81967 | 24010.67094 |
|  | 20 | 734.4263364 | 1250.058675 | 2258.871759 | 3417.796993 | 5232.800655 | 7838.129412 | 11177.04747 | 17561.15312 | 25149.05668 |
|  | 30 | 1084.32676 | 1730.268654 | 2916.625203 | 4214.811374 | 6180.03309 | 8935.796947 | 12431.41521 | 19138.41729 | 27258.7732 |
|  | 40 | 1506.154999 | 2290.846348 | 3651.924377 | 5074.435341 | 7158.713875 | 10016.70425 | 13609.41229 | 20544.24135 | 29134.69746 |
|  | 50 | 2143.265524 | 3133.186351 | 4752.707936 | 6365.083626 | 8646.063434 | 11698.11612 | 15496.08119 | 22873.79996 | 32248.53619 |
|  | 60 | 3342.382923 | 4694.231748 | 6758.402859 | 8696.51393 | 11322.33947 | 14726.15403 | 18895.57196 | 27006.98639 | 37547.62145 |
|  | 70 | 5497.09661 | 7405.51768 | 10090.45994 | 12440.91912 | 15465.15856 | 19236.98927 | 23761.95863 | 32538.09596 | 44156.27957 |
|  | 80 | 8799.650934 | 11397.71831 | 14731.76072 | 17430.48237 | 20713.15125 | 24644.8925 | 29266.74777 | 38221.9742 | 50328.55302 |
|  | 90 | 12888.2044 | 16214.83154 | 20095.72739 | 22993.4485 | 26330.83778 | 30191.75544 | 34680.5429 | 43491.60124 | 55854.02524 |
| **Right**  **Lateral**  **Ventricle** | 6 | 336.0282848 | 660.6972485 | 1366.792306 | 2244.498739 | 3707.767202 | 5937.879193 | 8959.537912 | 15120.96895 | 22953.82835 |
|  | 10 | 420.3927699 | 788.9949825 | 1559.134947 | 2488.469746 | 4005.028322 | 6278.317694 | 9325.521698 | 15499.82106 | 23342.8108 |
|  | 15 | 546.4238729 | 974.6136596 | 1829.087162 | 2824.874954 | 4408.955663 | 6736.31631 | 9815.284417 | 16005.53507 | 23861.41105 |
|  | 20 | 693.4670613 | 1184.994366 | 2126.648085 | 3189.837471 | 4842.014953 | 7224.976698 | 10339.84757 | 16559.95199 | 24453.4472 |
|  | 30 | 1026.638353 | 1644.537447 | 2751.117203 | 3935.271558 | 5703.639645 | 8176.263192 | 11346.86592 | 17623.95396 | 25627.88632 |
|  | 40 | 1393.644148 | 2142.719243 | 3417.666523 | 4726.576993 | 6622.968881 | 9216.441513 | 12504.77193 | 19017.09978 | 27439.76209 |
|  | 50 | 1936.659697 | 2881.431031 | 4413.945413 | 5925.560771 | 8052.656204 | 10901.20013 | 14477.51005 | 21577.84728 | 30921.14663 |
|  | 60 | 2977.675858 | 4256.446065 | 6208.825305 | 8038.624447 | 10516.61837 | 13737.60692 | 17710.06186 | 25546.48303 | 35944.29621 |
|  | 70 | 4955.734116 | 6735.239874 | 9250.988731 | 11460.18577 | 14307.55346 | 17861.66671 | 22124.65593 | 30379.4894 | 41269.79532 |
|  | 80 | 8154.368606 | 10523.55698 | 13602.20521 | 16120.55317 | 19198.56949 | 22877.99214 | 27160.85204 | 35276.08098 | 45864.6518 |
|  | 90 | 12203.10255 | 15119.84876 | 18621.55349 | 21303.14658 | 24429.7376 | 28036.29249 | 32144.94558 | 39843.4295 | 49883.09905 |
| **Left**  **Thalamus** | 6 | 6440.882403 | 6774.288808 | 7199.211104 | 7529.320013 | 7892.3134 | 8251.580389 | 8571.939037 | 8975.592933 | 9285.555808 |
|  | 10 | 6391.108422 | 6718.515289 | 7147.87217 | 7490.671813 | 7876.856484 | 8268.51438 | 8625.60496 | 9085.984148 | 9447.345718 |
|  | 15 | 6361.852786 | 6675.249484 | 7099.983418 | 7450.588962 | 7858.124703 | 8285.369144 | 8687.439448 | 9223.911494 | 9659.639009 |
|  | 20 | 6340.27254 | 6644.68058 | 7065.691827 | 7420.821206 | 7842.561019 | 8295.371453 | 8731.813967 | 9330.264724 | 9830.347728 |
|  | 30 | 6136.863285 | 6498.966224 | 6986.25425 | 7385.406115 | 7845.786392 | 8324.250949 | 8770.603935 | 9360.264547 | 9834.275118 |
|  | 40 | 6021.292589 | 6381.457282 | 6867.736085 | 7267.389576 | 7729.789472 | 8211.941251 | 8663.150659 | 9261.258145 | 9743.684799 |
|  | 50 | 5805.914634 | 6126.245416 | 6577.109501 | 6964.553157 | 7433.23252 | 7946.897273 | 8452.35256 | 9162.163761 | 9770.328391 |
|  | 60 | 5446.35923 | 5789.148375 | 6247.702364 | 6621.00501 | 7049.034137 | 7491.060002 | 7900.895012 | 8438.682497 | 8868.084896 |
|  | 70 | 4627.654852 | 5136.649366 | 5752.623353 | 6210.684031 | 6697.29501 | 7163.861391 | 7568.932194 | 8066.427093 | 8439.756051 |
|  | 80 | 4698.928834 | 5046.115403 | 5537.015783 | 5960.255394 | 6473.138027 | 7035.494007 | 7588.299685 | 8362.395068 | 9022.600418 |
|  | 90 | 5441.414268 | 5586.902136 | 5815.14093 | 6040.968242 | 6367.601212 | 6831.567763 | 7479.584354 | 9082.288694 | 11800.6391 |
| **Right**  **Thalamus** | 6 | 6509.958 | 6791.534402 | 7151.572781 | 7439.183443 | 7772.373962 | 8130.873632 | 8487.190574 | 9010.42047 | 9495.371468 |
|  | 10 | 6347.448023 | 6647.418553 | 7026.587227 | 7326.589679 | 7671.798875 | 8041.449482 | 8407.97777 | 8946.103245 | 9445.798945 |
|  | 15 | 6146.883598 | 6473.081774 | 6878.544074 | 7194.896377 | 7555.358263 | 7938.640803 | 8317.349951 | 8873.126502 | 9390.491437 |
|  | 20 | 5976.227025 | 6333.683886 | 6769.140551 | 7103.249149 | 7479.496278 | 7876.211137 | 8266.512375 | 8838.884663 | 9373.058951 |
|  | 30 | 5732.67599 | 6167.975491 | 6671.309901 | 7041.387002 | 7446.190825 | 7864.510521 | 8272.178101 | 8869.858022 | 9432.062362 |
|  | 40 | 5512.65103 | 6009.334054 | 6550.476464 | 6930.498406 | 7334.745729 | 7746.368317 | 8147.283243 | 8743.14427 | 9317.991484 |
|  | 50 | 5212.737096 | 5744.529737 | 6292.002481 | 6660.819822 | 7045.095131 | 7435.113883 | 7820.589053 | 8414.131684 | 9016.954532 |
|  | 60 | 4855.152588 | 5426.316643 | 5985.015629 | 6347.570599 | 6719.921765 | 7100.935119 | 7489.379776 | 8124.815963 | 8826.101761 |
|  | 70 | 4400.391358 | 5032.818345 | 5624.175488 | 5994.333241 | 6370.478589 | 6762.618517 | 7182.522791 | 7935.518966 | 8878.505726 |
|  | 80 | 4011.474412 | 4708.150068 | 5331.486059 | 5705.47945 | 6080.621941 | 6481.851647 | 6941.529247 | 7881.313475 | 9305.376338 |
|  | 90 | 3705.767278 | 4479.370856 | 5137.671191 | 5509.830016 | 5874.113219 | 6274.152207 | 6772.873017 | 7991.453702 | 10460.02019 |
| **Left**  **Caudate** | 6 | 3048.889921 | 3322.503325 | 3670.992412 | 3944.01802 | 4249.46905 | 4560.405414 | 4847.972545 | 5229.148185 | 5540.373947 |
|  | 10 | 2983.108271 | 3248.844477 | 3587.133599 | 3852.408769 | 4149.889567 | 4453.964177 | 4736.753154 | 5114.583721 | 5426.104704 |
|  | 15 | 2902.506223 | 3159.559192 | 3486.179276 | 3742.365698 | 4030.380936 | 4326.301802 | 4603.549629 | 4978.049295 | 5291.0857 |
|  | 20 | 2829.326254 | 3079.480485 | 3396.229352 | 3644.448434 | 3924.060046 | 4212.831872 | 4485.54722 | 4858.469309 | 5175.117707 |
|  | 30 | 2722.24672 | 2965.003575 | 3268.41987 | 3504.718297 | 3771.382624 | 4049.610891 | 4317.159582 | 4693.983684 | 5026.658329 |
|  | 40 | 2622.741767 | 2864.622906 | 3160.008213 | 3386.883938 | 3642.415089 | 3911.747573 | 4176.510881 | 4564.178803 | 4925.062426 |
|  | 50 | 2514.117887 | 2764.665106 | 3059.713074 | 3280.939199 | 3528.406106 | 3792.013781 | 4058.59474 | 4470.236855 | 4883.094535 |
|  | 60 | 2416.251059 | 2693.591932 | 3003.359987 | 3226.958423 | 3473.70785 | 3739.642431 | 4019.047118 | 4484.307029 | 5004.30123 |
|  | 70 | 2296.695157 | 2626.77588 | 2969.583251 | 3203.158421 | 3454.897831 | 3729.839736 | 4034.488641 | 4600.909609 | 5345.925027 |
|  | 80 | 2099.036787 | 2520.918444 | 2920.879612 | 3171.245484 | 3430.73946 | 3718.548565 | 4062.715353 | 4818.335063 | 6106.54265 |
|  | 90 | 1764.782176 | 2339.522214 | 2837.065398 | 3113.713739 | 3382.999033 | 3687.133987 | 4093.841835 | 5248.594515 | 8300.964194 |
| **Right**  **Caudate** | 6 | 3175.320489 | 3437.417866 | 3777.005335 | 4045.877078 | 4347.58805 | 4653.528584 | 4933.513403 | 5297.574965 | 5587.021232 |
|  | 10 | 3091.412525 | 3346.222825 | 3677.646598 | 3941.220491 | 4238.389942 | 4541.437782 | 4820.467893 | 5185.985026 | 5478.979063 |
|  | 15 | 2989.920186 | 3236.442749 | 3558.362716 | 3815.66496 | 4107.440608 | 4407.160442 | 4685.379103 | 5053.580466 | 5352.166369 |
|  | 20 | 2898.290293 | 3137.799237 | 3451.396364 | 3703.085251 | 3990.041194 | 4286.990693 | 4565.045097 | 4937.251336 | 5243.127077 |
|  | 30 | 2780.612752 | 3008.051303 | 3305.392677 | 3544.774423 | 3819.837096 | 4108.362193 | 4383.498338 | 4761.542595 | 5082.378998 |
|  | 40 | 2693.386867 | 2913.125245 | 3196.080931 | 3422.136544 | 3682.156029 | 3957.656102 | 4225.300751 | 4604.641909 | 4940.217368 |
|  | 50 | 2596.598594 | 2821.174378 | 3101.20279 | 3320.29174 | 3570.686421 | 3838.058069 | 4103.62591 | 4496.173862 | 4864.655805 |
|  | 60 | 2494.420854 | 2748.287328 | 3047.565142 | 3272.559256 | 3525.523006 | 3797.387017 | 4075.760454 | 4513.915302 | 4964.521837 |
|  | 70 | 2334.355125 | 2657.718502 | 3005.74021 | 3249.616946 | 3515.063603 | 3801.860957 | 4109.265305 | 4643.840465 | 5280.466764 |
|  | 80 | 2039.907693 | 2511.196159 | 2954.548905 | 3230.410027 | 3513.409804 | 3820.647389 | 4175.406615 | 4904.271632 | 6014.933349 |
|  | 90 | 1419.75829 | 2212.682164 | 2857.241202 | 3189.312943 | 3496.131393 | 3831.354257 | 4271.914063 | 5484.918118 | 8378.61899 |
| **Left**  **Putamen** | 6 | 5275.396377 | 5706.977339 | 6245.755552 | 6658.215804 | 7108.179672 | 7552.255967 | 7949.183128 | 8453.421366 | 8845.846911 |
|  | 10 | 5011.419727 | 5423.291637 | 5938.265296 | 6333.288931 | 6765.244673 | 7192.847549 | 7576.380484 | 8065.769882 | 8448.58675 |
|  | 15 | 4713.727595 | 5103.874291 | 5592.35428 | 5967.855744 | 6379.622592 | 6788.829327 | 7157.565878 | 7630.949654 | 8003.897924 |
|  | 20 | 4499.159818 | 4874.850419 | 5345.520091 | 5707.927311 | 6106.387266 | 6503.953926 | 6863.997468 | 7329.376115 | 7699.012499 |
|  | 30 | 4305.361876 | 4673.884295 | 5134.789093 | 5490.162658 | 5882.669897 | 6277.562703 | 6639.274722 | 7114.502861 | 7499.635861 |
|  | 40 | 4100.871443 | 4465.55589 | 4918.4574 | 5266.871591 | 5652.874306 | 6044.6416 | 6408.476196 | 6896.767908 | 7303.405678 |
|  | 50 | 3808.657095 | 4167.709339 | 4607.08695 | 4942.516756 | 5314.410805 | 5695.379982 | 6055.345599 | 6552.42458 | 6982.285433 |
|  | 60 | 3581.076973 | 3949.933391 | 4389.837607 | 4720.443187 | 5085.945884 | 5464.093641 | 5829.45299 | 6354.232708 | 6832.948979 |
|  | 70 | 3351.401562 | 3746.024466 | 4197.698224 | 4527.919871 | 4889.952255 | 5268.526529 | 5645.371542 | 6217.71058 | 6781.787808 |
|  | 80 | 3085.034018 | 3529.553345 | 4007.903314 | 4342.21334 | 4702.583352 | 5083.844989 | 5479.359443 | 6130.920007 | 6850.984787 |
|  | 90 | 2721.312048 | 3253.078999 | 3778.142897 | 4120.129439 | 4477.824869 | 4861.244695 | 5283.242595 | 6068.490708 | 7101.792189 |
| **Right**  **Putamen** | 6 | 5196.91273 | 5546.775849 | 5992.56834 | 6343.025167 | 6737.567683 | 7143.175059 | 7523.054374 | 8035.443466 | 8462.687848 |
|  | 10 | 4943.07681 | 5286.323314 | 5721.680477 | 6062.822355 | 6446.263476 | 6840.380969 | 7209.967276 | 7709.994022 | 8128.86072 |
|  | 15 | 4654.290237 | 4990.752376 | 5414.772535 | 5745.521977 | 6116.450366 | 6497.603989 | 6855.669733 | 7342.18209 | 7752.369399 |
|  | 20 | 4436.002914 | 4770.088059 | 5188.047874 | 5512.38229 | 5875.185719 | 6247.86944 | 6598.679706 | 7077.637524 | 7484.411878 |
|  | 30 | 4187.374226 | 4528.633134 | 4947.991018 | 5269.274709 | 5626.380981 | 5992.866459 | 6339.470242 | 6818.166924 | 7231.876258 |
|  | 40 | 3934.763393 | 4283.487533 | 4702.282359 | 5017.893893 | 5365.834956 | 5722.497667 | 6061.879692 | 6537.618765 | 6958.074218 |
|  | 50 | 3641.180096 | 4001.447484 | 4421.808569 | 4732.114324 | 5070.769031 | 5417.553096 | 5750.289241 | 6225.930567 | 6658.691716 |
|  | 60 | 3361.824284 | 3750.121462 | 4187.205323 | 4501.602843 | 4840.488603 | 5187.262165 | 5523.772956 | 6017.398575 | 6483.80463 |
|  | 70 | 3087.119525 | 3525.627355 | 3997.531171 | 4325.994411 | 4674.546087 | 5031.040106 | 5382.235383 | 5915.118672 | 6443.75847 |
|  | 80 | 2780.761369 | 3295.513584 | 3819.144328 | 4168.542237 | 4531.912775 | 4903.406492 | 5276.692257 | 5868.568754 | 6493.573835 |
|  | 90 | 2392.371334 | 3012.491844 | 3601.602457 | 3973.935557 | 4351.136625 | 4736.587442 | 5134.069531 | 5801.393408 | 6564.499653 |
| **Left**  **Pallidum** | 6 | 1472.79607 | 1631.634206 | 1810.078638 | 1938.568219 | 2077.856186 | 2222.110164 | 2364.627944 | 2579.567985 | 2790.03578 |
|  | 10 | 1374.763158 | 1534.158141 | 1714.249465 | 1844.463457 | 1985.947358 | 2132.612462 | 2277.438458 | 2495.400407 | 2708.087847 |
|  | 15 | 1263.606525 | 1423.336328 | 1605.023126 | 1737.028237 | 1880.835348 | 2030.047462 | 2177.265482 | 2398.200645 | 2612.811808 |
|  | 20 | 1175.313666 | 1333.494387 | 1514.365726 | 1646.259386 | 1790.192256 | 1939.548793 | 2086.678783 | 2306.687187 | 2519.2621 |
|  | 30 | 1097.222463 | 1249.648499 | 1424.806342 | 1552.913654 | 1692.741151 | 1837.438206 | 1979.175851 | 2189.056735 | 2389.191698 |
|  | 40 | 1041.387435 | 1193.332547 | 1368.803653 | 1497.516451 | 1638.03656 | 1783.075082 | 1924.393346 | 2131.733994 | 2327.012212 |
|  | 50 | 979.1068071 | 1126.616458 | 1297.537479 | 1423.123822 | 1560.143228 | 1701.110492 | 1837.67691 | 2036.165603 | 2220.80328 |
|  | 60 | 966.2705303 | 1111.834259 | 1280.750463 | 1404.895316 | 1540.122609 | 1678.690685 | 1812.100692 | 2004.128622 | 2180.544007 |
|  | 70 | 912.51532 | 1062.449132 | 1237.07327 | 1365.652304 | 1505.656268 | 1648.724057 | 1785.779694 | 1981.435131 | 2159.244409 |
|  | 80 | 785.5010648 | 949.214283 | 1141.155146 | 1283.033196 | 1437.679235 | 1595.483629 | 1746.086947 | 1959.61223 | 2151.850868 |
|  | 90 | 633.1688059 | 821.6618398 | 1044.374355 | 1209.643496 | 1389.938751 | 1573.614271 | 1748.22334 | 1994.080503 | 2213.3772 |
| **Right**  **Pallidum** | 6 | 1364.616213 | 1494.644741 | 1645.158661 | 1756.636713 | 1880.68189 | 2012.996089 | 2147.916945 | 2359.952682 | 2578.17603 |
|  | 10 | 1308.094344 | 1437.180414 | 1585.510402 | 1694.704503 | 1815.717297 | 1944.464329 | 2075.630471 | 2281.893222 | 2494.55656 |
|  | 15 | 1242.609153 | 1370.899924 | 1516.913035 | 1623.555833 | 1741.125314 | 1865.797205 | 1992.672061 | 2192.360947 | 2398.739306 |
|  | 20 | 1186.337481 | 1314.690195 | 1459.317502 | 1564.081131 | 1678.955342 | 1800.358223 | 1923.773211 | 2118.208541 | 2319.67218 |
|  | 30 | 1111.554511 | 1244.343494 | 1390.781378 | 1495.006926 | 1607.992793 | 1726.565803 | 1846.853477 | 2036.805759 | 2234.73734 |
|  | 40 | 1055.228775 | 1195.5763 | 1346.709906 | 1452.230728 | 1565.224535 | 1682.936319 | 1802.116296 | 1990.859032 | 2188.786588 |
|  | 50 | 997.7950771 | 1146.993216 | 1303.465342 | 1410.439136 | 1523.485168 | 1640.34489 | 1758.447096 | 1946.126227 | 2144.347236 |
|  | 60 | 944.9052918 | 1105.601782 | 1269.228735 | 1378.539419 | 1492.422493 | 1609.196175 | 1727.016465 | 1915.007367 | 2115.138491 |
|  | 70 | 873.2944307 | 1044.542584 | 1213.253475 | 1323.132727 | 1435.865219 | 1550.478506 | 1665.950286 | 1851.064695 | 2049.866947 |
|  | 80 | 792.8182698 | 974.9099623 | 1147.896273 | 1257.460165 | 1368.026261 | 1479.436808 | 1591.543286 | 1772.241569 | 1968.175101 |
|  | 90 | 717.9655303 | 914.2941917 | 1093.773407 | 1204.049116 | 1313.375014 | 1422.510405 | 1532.221046 | 1710.158056 | 1905.13088 |
| **Left Hippocampus** | 6 | 3275.564726 | 3575.178737 | 3888.247654 | 4100.889633 | 4321.581305 | 4541.464506 | 4751.804704 | 5058.770806 | 5349.499828 |
|  | 10 | 3255.09555 | 3555.68787 | 3873.196612 | 4090.412794 | 4316.355667 | 4540.912895 | 4754.207424 | 5061.459128 | 5347.395511 |
|  | 15 | 3224.605727 | 3527.159951 | 3850.53913 | 4073.4769 | 4305.857034 | 4536.060026 | 4752.877001 | 5060.489619 | 5340.984065 |
|  | 20 | 3190.309889 | 3494.969314 | 3823.887111 | 4052.084056 | 4290.276473 | 4525.431995 | 4745.141216 | 5052.487711 | 5327.520158 |
|  | 30 | 3132.847076 | 3440.372502 | 3777.613828 | 4013.777272 | 4260.553743 | 4502.462808 | 4725.155291 | 5028.917392 | 5291.855781 |
|  | 40 | 3071.303262 | 3382.332815 | 3726.915326 | 3969.517913 | 4222.795077 | 4469.2403 | 4693.076061 | 4991.802983 | 5243.166451 |
|  | 50 | 2987.857021 | 3307.755293 | 3663.741534 | 3914.694415 | 4175.89041 | 4427.986853 | 4654.076455 | 4950.026338 | 5193.026093 |
|  | 60 | 2796.163192 | 3132.99207 | 3506.318527 | 3768.310611 | 4039.26255 | 4298.193479 | 4527.429581 | 4822.0993 | 5058.76633 |
|  | 70 | 2445.279125 | 2813.926488 | 3214.928053 | 3492.423336 | 3776.011267 | 4043.38702 | 4276.663077 | 4571.065693 | 4802.597937 |
|  | 80 | 2018.752835 | 2437.986427 | 2877.277955 | 3173.629083 | 3471.076046 | 3746.730401 | 3983.33178 | 4276.422235 | 4502.341795 |
|  | 90 | 1573.229194 | 2052.683776 | 2532.75797 | 2845.489292 | 3152.225873 | 3430.87971 | 3665.967016 | 3951.92608 | 4168.269659 |
| **Right**  **Hippocampus** | 6 | 3506.338825 | 3701.389446 | 3948.294353 | 4142.161793 | 4361.410941 | 4589.272383 | 4806.210504 | 5106.229069 | 5364.506632 |
|  | 10 | 3474.41212 | 3676.967057 | 3931.152361 | 4129.269272 | 4352.099631 | 4582.668514 | 4801.551571 | 5103.736175 | 5363.753682 |
|  | 15 | 3427.693575 | 3640.19755 | 3903.732068 | 4107.105598 | 4334.192091 | 4567.828551 | 4788.822635 | 5093.319666 | 5355.263017 |
|  | 20 | 3373.819776 | 3596.939712 | 3870.055597 | 4078.556893 | 4309.567129 | 4545.828465 | 4768.496881 | 5074.766599 | 5338.282748 |
|  | 30 | 3293.149363 | 3542.315902 | 3838.184491 | 4058.553383 | 4298.543093 | 4540.882884 | 4767.637237 | 5078.697027 | 5346.817712 |
|  | 40 | 3201.387835 | 3482.79916 | 3804.750215 | 4037.649541 | 4286.381484 | 4534.145498 | 4764.390647 | 5079.89003 | 5352.993605 |
|  | 50 | 3053.09256 | 3378.53496 | 3733.881712 | 3982.077978 | 4241.274567 | 4495.675293 | 4730.564645 | 5052.640859 | 5333.384325 |
|  | 60 | 2800.869427 | 3188.009777 | 3585.458351 | 3851.136421 | 4121.28523 | 4382.037401 | 4621.240445 | 4949.97173 | 5239.277981 |
|  | 70 | 2392.243555 | 2860.070629 | 3302.873823 | 3582.693192 | 3858.11442 | 4118.826963 | 4356.387388 | 4684.118289 | 4976.11471 |
|  | 80 | 1913.136749 | 2481.645846 | 2978.187975 | 3271.543734 | 3549.333536 | 3806.50039 | 4039.26303 | 4362.300066 | 4654.624389 |
|  | 90 | 1460.939279 | 2098.321523 | 2647.245007 | 2951.479013 | 3227.749486 | 3477.540576 | 3702.365703 | 4017.27896 | 4307.920003 |
| **Left**  **Amygdala** | 6 | 1203.653862 | 1295.532176 | 1415.051776 | 1511.602898 | 1623.933339 | 1744.549341 | 1863.40471 | 2034.862839 | 2189.606029 |
|  | 10 | 1193.615377 | 1288.04844 | 1410.129853 | 1508.164773 | 1621.625341 | 1742.817703 | 1861.670635 | 2032.250654 | 2185.412601 |
|  | 15 | 1179.630125 | 1277.382798 | 1402.718759 | 1502.584453 | 1617.382919 | 1739.186198 | 1857.921008 | 2027.258234 | 2178.359842 |
|  | 20 | 1166.562868 | 1267.947676 | 1396.796037 | 1498.614829 | 1614.833479 | 1737.298009 | 1855.952781 | 2024.11986 | 2173.27242 |
|  | 30 | 1149.768149 | 1260.15 | 1397.694255 | 1504.443629 | 1624.47194 | 1749.156885 | 1868.484038 | 2035.540334 | 2182.014196 |
|  | 40 | 1118.737737 | 1240.11136 | 1388.009423 | 1500.547728 | 1625.086808 | 1752.57861 | 1873.117124 | 2039.923624 | 2184.669442 |
|  | 50 | 1052.940988 | 1187.127537 | 1346.380387 | 1464.874028 | 1593.74896 | 1723.662557 | 1844.982925 | 2010.988279 | 2153.657679 |
|  | 60 | 950.8252161 | 1100.700502 | 1272.682987 | 1397.238545 | 1530.033971 | 1661.634335 | 1782.916846 | 1946.957658 | 2086.606957 |
|  | 70 | 816.1512357 | 989.3575533 | 1178.997714 | 1311.625937 | 1449.644015 | 1583.726252 | 1705.474914 | 1868.093167 | 2005.169485 |
|  | 80 | 648.6814827 | 852.7830708 | 1062.94315 | 1203.332251 | 1345.106605 | 1479.633967 | 1599.7416 | 1757.982395 | 1889.988841 |
|  | 90 | 476.3429138 | 710.0322389 | 942.0023909 | 1089.216385 | 1232.782221 | 1365.424379 | 1481.681781 | 1632.658896 | 1757.294888 |
| **Right Amygdala** | 6 | 1232.614802 | 1328.989782 | 1457.564877 | 1563.029978 | 1686.132621 | 1817.183547 | 1943.714058 | 2119.610347 | 2270.382742 |
|  | 10 | 1226.053035 | 1323.112271 | 1452.184522 | 1557.817584 | 1680.983704 | 1812.1021 | 1938.842035 | 2115.488419 | 2267.499399 |
|  | 15 | 1217.343656 | 1315.335493 | 1445.062482 | 1550.893394 | 1674.102092 | 1805.262126 | 1932.240525 | 2109.857227 | 2263.537041 |
|  | 20 | 1209.626554 | 1308.82654 | 1439.461588 | 1545.62819 | 1668.990302 | 1800.280956 | 1927.582784 | 2106.332409 | 2261.903733 |
|  | 30 | 1204.270514 | 1307.864179 | 1442.371402 | 1550.526698 | 1675.442557 | 1808.102785 | 1937.014621 | 2119.366675 | 2280.018597 |
|  | 40 | 1187.097785 | 1297.972223 | 1438.88052 | 1550.267486 | 1677.517392 | 1811.821811 | 1942.245547 | 2127.725596 | 2292.94528 |
|  | 50 | 1136.757495 | 1259.310281 | 1410.044078 | 1526.04916 | 1656.178225 | 1791.845808 | 1922.931827 | 2109.713673 | 2277.526836 |
|  | 60 | 1050.870234 | 1192.187823 | 1357.753463 | 1480.259626 | 1614.058079 | 1750.99796 | 1882.168478 | 2069.072414 | 2238.358722 |
|  | 70 | 931.4520983 | 1102.658889 | 1289.509287 | 1420.424851 | 1558.453801 | 1696.479037 | 1827.384554 | 2014.213918 | 2185.419912 |
|  | 80 | 772.2583867 | 989.7773052 | 1204.829781 | 1344.853061 | 1486.097411 | 1623.603317 | 1752.85216 | 1938.490638 | 2111.804215 |
|  | 90 | 588.6165746 | 863.1200329 | 1113.699741 | 1263.410433 | 1406.800878 | 1542.445761 | 1669.201214 | 1853.795501 | 2031.064614 |
| **Left Nucleus**  **Accumbens** | 6 | 486.6956193 | 547.2878891 | 624.1840175 | 685.1651656 | 755.5017423 | 831.1077829 | 906.4779478 | 1017.99569 | 1122.472789 |
|  | 10 | 437.6064563 | 496.5835133 | 571.6571726 | 631.2984055 | 700.1239593 | 774.061662 | 847.6467559 | 956.1834356 | 1057.416141 |
|  | 15 | 387.5516377 | 445.0999842 | 518.5654333 | 577.0049019 | 644.4305868 | 716.7498391 | 788.5194157 | 893.8769966 | 991.5190147 |
|  | 20 | 357.6867249 | 415.0543803 | 488.3093264 | 546.5235667 | 613.5486563 | 685.192251 | 755.9725099 | 859.2095245 | 954.1223612 |
|  | 30 | 340.0184981 | 397.3917072 | 469.9457398 | 527.0055847 | 591.9982904 | 660.580999 | 727.3909017 | 823.1129365 | 909.333591 |
|  | 40 | 309.2997641 | 369.6309789 | 445.4598522 | 504.6520842 | 571.5175544 | 641.3528988 | 708.6032027 | 803.5408376 | 887.6084996 |
|  | 50 | 267.7308236 | 329.5416149 | 406.4885994 | 465.9476864 | 532.4376804 | 601.0713832 | 666.347931 | 757.1020681 | 836.1049141 |
|  | 60 | 238.5142156 | 300.6351962 | 376.6945521 | 434.5926932 | 498.4849016 | 563.5159217 | 624.5081264 | 707.9368116 | 779.3018636 |
|  | 70 | 168.5823234 | 241.0455744 | 328.4930159 | 394.0297595 | 465.3714799 | 536.9699832 | 603.2138326 | 692.4336052 | 767.5123102 |
|  | 80 | 89.56975547 | 169.8755095 | 270.1259956 | 343.9651882 | 422.7753079 | 500.3546333 | 570.900328 | 664.1924836 | 741.2749873 |
|  | 90 | 47.64995278 | 116.9955882 | 218.4524612 | 293.8126709 | 372.7321704 | 448.7729285 | 516.6142049 | 604.6046189 | 675.9531536 |
| **Right Nucleus**  **Accumbens** | 6 | 488.5508053 | 538.1377664 | 605.1333146 | 660.1392956 | 723.6335694 | 789.5992987 | 851.0577142 | 932.0565737 | 996.9651694 |
|  | 10 | 470.4475101 | 520.4068166 | 587.8583991 | 643.191329 | 707.0045774 | 773.232097 | 834.869009 | 916.0059564 | 980.9457408 |
|  | 15 | 448.2825117 | 498.7357776 | 566.7851109 | 622.540557 | 686.7593295 | 753.3118738 | 815.1624036 | 896.4501189 | 961.4049781 |
|  | 20 | 426.6189568 | 477.602188 | 546.2842196 | 602.4798491 | 667.1134647 | 733.9897177 | 796.0440036 | 877.4589124 | 942.4028443 |
|  | 30 | 384.6991347 | 436.8681558 | 506.9357157 | 564.0715871 | 629.5664113 | 697.0866688 | 759.5177791 | 841.1140666 | 905.9565142 |
|  | 40 | 344.3973255 | 397.9461965 | 469.5756444 | 527.733216 | 594.1247704 | 662.2720804 | 725.0250134 | 806.6859385 | 871.3060736 |
|  | 50 | 305.4301035 | 360.5997144 | 433.9979325 | 493.265449 | 560.585 | 629.3305593 | 692.3364058 | 773.926647 | 838.1905252 |
|  | 60 | 267.5511584 | 324.660084 | 400.0885321 | 460.5736512 | 528.8596678 | 598.1737137 | 661.359935 | 742.7399009 | 806.5125959 |
|  | 70 | 230.4777941 | 289.961723 | 367.7595843 | 429.5975312 | 498.9008481 | 568.7567533 | 632.051127 | 713.0828986 | 776.2338397 |
|  | 80 | 193.8429645 | 256.2953034 | 336.8966956 | 400.2500057 | 470.6285479 | 540.9962812 | 604.3212784 | 684.8623107 | 747.2609808 |
|  | 90 | 157.3210752 | 223.4422339 | 307.3791189 | 372.4369116 | 443.9525079 | 514.795597 | 578.0666132 | 657.9688328 | 719.4848101 |

| **Table S8. Centile Values for Subcortical Volumes in Females** | | | | | | | | | | | |
| --- | --- | --- | --- | --- | --- | --- | --- | --- | --- | --- | --- |
| **Region** |  | **Age** | **C0.4** | **C2** | **C10** | **C25** | **C50** | **C75** | **C90** | **C98** | **C99.6** |
| **Left**  **Lateral**  **Ventricle** | 12 | 6 | 545.1216169 | 1078.58624 | 2007.807114 | 2938.843923 | 4255.556466 | 6051.462899 | 8400.47564 | 13467.50553 | 20999.53335 |
|  | 13 | 10 | 723.4633652 | 1305.727128 | 2286.170501 | 3255.762634 | 4619.511333 | 6470.778291 | 8878.045583 | 14022.46007 | 21581.52107 |
|  | 14 | 15 | 989.7525114 | 1633.372379 | 2681.873864 | 3704.494128 | 5133.476758 | 7061.612927 | 9550.421669 | 14808.67226 | 22427.9925 |
|  | 15 | 20 | 1282.275244 | 1979.293903 | 3088.407675 | 4159.281278 | 5648.398736 | 7648.143487 | 10213.97226 | 15584.54542 | 23279.26416 |
|  | 16 | 30 | 1802.001931 | 2542.786093 | 3696.501898 | 4803.650601 | 6339.856433 | 8392.338734 | 10998.03942 | 16339.91599 | 23773.22836 |
|  | 17 | 40 | 2329.797276 | 3131.975958 | 4383.08966 | 5592.685408 | 7281.714966 | 9542.504783 | 12397.85207 | 18158.57313 | 25963.6407 |
|  | 18 | 50 | 2803.320408 | 3692.591795 | 5076.962634 | 6415.135918 | 8281.708237 | 10770.60964 | 13890.87072 | 20097.57239 | 28333.95115 |
|  | 19 | 60 | 3258.519329 | 4366.650239 | 6051.363505 | 7637.714444 | 9797.317162 | 12609.46698 | 16063.60688 | 22791.1779 | 31536.53951 |
|  | 20 | 70 | 4505.581063 | 6257.315748 | 8697.909526 | 10802.39707 | 13465.086 | 16720.56045 | 20541.40981 | 27727.61583 | 36862.02039 |
|  | 21 | 80 | 6656.04142 | 9563.034851 | 13010.14887 | 15577.68418 | 18500.48666 | 21814.07907 | 25557.79611 | 32550.14677 | 41620.41174 |
|  | 22 | 90 | 8811.943032 | 13317.9431 | 17753.71275 | 20564.37308 | 23456.64194 | 26567.35108 | 30079.33722 | 36967.68368 | 46678.45074 |
| **Right**  **Lateral**  **Ventricle** | 23 | 6 | 697.9018839 | 1184.928734 | 2002.552002 | 2818.427738 | 3975.878316 | 5552.244199 | 7591.517182 | 11872.17132 | 17972.4952 |
|  | 24 | 10 | 780.0281619 | 1313.725711 | 2200.032904 | 3075.93753 | 4307.292218 | 5966.824963 | 8088.869458 | 12471.59496 | 18592.425 |
|  | 25 | 15 | 899.2502846 | 1497.179323 | 2476.140437 | 3431.697873 | 4759.703868 | 6526.234296 | 8752.809001 | 13260.88226 | 19404.03226 |
|  | 26 | 20 | 1039.527543 | 1699.720371 | 2763.262323 | 3788.024732 | 5196.180568 | 7045.99998 | 9346.155154 | 13918.18209 | 20010.42963 |
|  | 27 | 30 | 1464.724322 | 2208.377019 | 3363.704053 | 4454.305478 | 5933.929054 | 7855.822406 | 10220.23411 | 14859.6413 | 20958.50131 |
|  | 28 | 40 | 2102.556549 | 2905.14381 | 4127.663102 | 5279.568013 | 6855.308246 | 8934.686693 | 11549.4154 | 16866.65043 | 24228.98988 |
|  | 29 | 50 | 2581.833494 | 3439.734066 | 4740.415873 | 5971.93022 | 7677.6894 | 9977.957081 | 12961.61535 | 19363.50964 | 28978.75645 |
|  | 30 | 60 | 2995.602464 | 4035.607576 | 5581.183267 | 7013.391798 | 8959.643389 | 11538.42341 | 14836.06236 | 21813.47455 | 32149.50445 |
|  | 31 | 70 | 4150.613955 | 5743.559961 | 7943.197156 | 9828.491109 | 12212.396 | 15145.18806 | 18635.39433 | 25380.4701 | 34311.93624 |
|  | 32 | 80 | 6121.033068 | 8719.865176 | 11902.65243 | 14340.66493 | 17154.63069 | 20339.2266 | 23869.21302 | 30183.79003 | 37860.71812 |
|  | 33 | 90 | 8203.891382 | 12140.78803 | 16399.08875 | 19334.3978 | 22478.99882 | 25837.62077 | 29417.8494 | 35625.57743 | 42967.85086 |
| **Left**  **Thalamus** | 34 | 6 | 6369.352705 | 6672.525908 | 7060.969906 | 7367.387838 | 7712.635762 | 8066.965738 | 8397.442038 | 8839.86761 | 9204.986596 |
|  | 35 | 10 | 6371.789847 | 6677.664262 | 7071.026355 | 7382.818878 | 7736.111445 | 8101.338231 | 8444.798302 | 8909.493388 | 9297.68345 |
|  | 36 | 15 | 6367.345442 | 6676.278251 | 7075.273964 | 7393.402747 | 7756.496955 | 8135.478 | 8495.874183 | 8990.661596 | 9411.106065 |
|  | 37 | 20 | 6349.753831 | 6660.998524 | 7064.587055 | 7388.285436 | 7760.556652 | 8153.171817 | 8531.174718 | 9058.778879 | 9515.993037 |
|  | 38 | 30 | 6292.453086 | 6606.209692 | 7015.924239 | 7348.452979 | 7737.219534 | 8157.002716 | 8573.082744 | 9177.714927 | 9728.166911 |
|  | 39 | 40 | 6149.315827 | 6467.234558 | 6881.159739 | 7218.152371 | 7616.060446 | 8053.794022 | 8499.367545 | 9173.922503 | 9822.004086 |
|  | 40 | 50 | 5894.439993 | 6222.765506 | 6641.500133 | 6977.968782 | 7373.983904 | 7812.720113 | 8267.349077 | 8979.489403 | 9698.578502 |
|  | 41 | 60 | 5551.666122 | 5909.348918 | 6344.220791 | 6680.888602 | 7068.754198 | 7495.263146 | 7940.864767 | 8658.425124 | 9416.985174 |
|  | 42 | 70 | 5096.697108 | 5533.537466 | 6017.116783 | 6364.223645 | 6746.280983 | 7157.905657 | 7591.265572 | 8316.921687 | 9136.036307 |
|  | 43 | 80 | 4428.860662 | 5078.803402 | 5685.774385 | 6065.04908 | 6451.087962 | 6856.445404 | 7296.274965 | 8103.625673 | 9148.468587 |
|  | 44 | 90 | 3190.304655 | 4449.183301 | 5358.774316 | 5808.385914 | 6211.356819 | 6622.986424 | 7103.925291 | 8159.193108 | 9881.654224 |
| **Right**  **Thalamus** | 45 | 6 | 6338.998475 | 6616.160682 | 6959.346937 | 7223.894687 | 7519.067284 | 7822.572016 | 8109.530104 | 8504.789585 | 8844.760628 |
|  | 46 | 10 | 6267.373802 | 6549.840058 | 6899.918879 | 7170.41001 | 7473.356509 | 7786.674462 | 8085.129629 | 8500.556037 | 8862.483534 |
|  | 47 | 15 | 6176.807047 | 6465.845935 | 6824.447561 | 7102.346227 | 7415.142371 | 7741.185635 | 8054.926672 | 8497.965278 | 8890.873841 |
|  | 48 | 20 | 6095.48745 | 6391.132691 | 6758.339524 | 7043.803218 | 7366.831073 | 7706.390947 | 8036.760109 | 8510.720821 | 8939.423954 |
|  | 49 | 30 | 5996.89494 | 6302.99646 | 6684.342166 | 6982.880608 | 7324.560825 | 7690.171475 | 8054.260052 | 8594.545694 | 9104.565413 |
|  | 50 | 40 | 5834.82364 | 6146.999562 | 6534.129279 | 6837.078229 | 7185.474338 | 7562.464708 | 7944.425825 | 8527.126006 | 9097.948745 |
|  | 51 | 50 | 5580.664721 | 5904.473325 | 6297.037731 | 6599.272034 | 6944.298233 | 7318.064809 | 7700.645941 | 8297.557314 | 8902.364784 |
|  | 52 | 60 | 5285.628159 | 5635.240678 | 6039.277294 | 6339.084374 | 6674.493007 | 7035.850839 | 7409.676676 | 8010.935153 | 8650.127677 |
|  | 53 | 70 | 4949.161955 | 5359.391395 | 5793.729217 | 6094.537235 | 6418.452787 | 6763.549439 | 7127.047517 | 7743.254413 | 8453.874495 |
|  | 54 | 80 | 4453.324053 | 5011.395749 | 5519.857851 | 5831.562782 | 6145.943924 | 6476.156955 | 6838.123013 | 7517.928026 | 8427.942311 |
|  | 55 | 90 | 3640.623925 | 4558.728984 | 5230.013402 | 5569.146239 | 5877.853682 | 6197.315422 | 6574.974774 | 7419.548052 | 8848.770322 |
| **Left**  **Caudate** | 56 | 6 | 3086.018579 | 3345.35412 | 3675.46552 | 3932.634552 | 4217.292372 | 4502.157772 | 4759.873281 | 5091.193698 | 5351.889427 |
|  | 57 | 10 | 3029.179945 | 3274.790158 | 3590.480312 | 3838.825151 | 4116.263142 | 4396.701132 | 4652.943302 | 4986.101441 | 5251.354251 |
|  | 58 | 15 | 2960.214583 | 3190.956274 | 3490.633539 | 3729.009354 | 3998.279902 | 4273.916331 | 4529.062579 | 4865.896268 | 5138.515327 |
|  | 59 | 20 | 2899.771146 | 3118.841356 | 3405.752002 | 3636.201865 | 3899.259134 | 4171.951577 | 4427.829294 | 4771.304416 | 5054.489837 |
|  | 60 | 30 | 2803.939825 | 3009.459932 | 3280.821677 | 3501.55643 | 3757.741291 | 4029.427961 | 4291.35277 | 4655.790141 | 4969.207745 |
|  | 61 | 40 | 2710.556764 | 2914.642774 | 3181.473735 | 3397.838057 | 3650.073204 | 3921.051926 | 4187.768019 | 4571.289819 | 4915.653691 |
|  | 62 | 50 | 2612.778202 | 2826.616706 | 3096.991755 | 3311.020101 | 3557.675817 | 3822.650687 | 4086.666388 | 4477.144977 | 4842.992194 |
|  | 63 | 60 | 2525.21406 | 2767.325637 | 3053.683028 | 3268.837875 | 3509.185638 | 3763.811818 | 4018.867707 | 4406.491355 | 4787.018659 |
|  | 64 | 70 | 2405.504903 | 2717.620089 | 3045.32649 | 3269.600484 | 3506.486654 | 3750.699409 | 3996.281252 | 4383.280082 | 4787.537383 |
|  | 65 | 80 | 2136.597197 | 2619.43241 | 3033.377768 | 3276.201666 | 3511.562351 | 3745.777704 | 3985.059543 | 4387.830853 | 4851.956342 |
|  | 66 | 90 | 1649.994031 | 2443.93574 | 3023.081379 | 3298.640279 | 3535.947792 | 3763.903572 | 4007.844426 | 4471.296121 | 5091.826211 |
| **Right**  **Caudate** | 67 | 6 | 3128.821569 | 3413.767022 | 3767.164882 | 4037.553196 | 4334.21322 | 4630.687773 | 4900.582875 | 5252.863919 | 5536.510082 |
|  | 68 | 10 | 3059.710774 | 3327.379391 | 3662.765672 | 3922.033432 | 4209.339917 | 4499.696445 | 4767.078136 | 5120.851171 | 5409.92627 |
|  | 69 | 15 | 2976.937913 | 3225.811075 | 3541.033238 | 3787.563608 | 4064.033319 | 4347.384881 | 4612.249012 | 4969.149 | 5266.752933 |
|  | 70 | 20 | 2910.289834 | 3143.826268 | 3442.273082 | 3678.134591 | 3945.700859 | 4223.865198 | 4488.032001 | 4851.195705 | 5161.015719 |
|  | 71 | 30 | 2826.278697 | 3039.235968 | 3314.391592 | 3535.318763 | 3791.075377 | 4064.445392 | 4332.818812 | 4718.627241 | 5065.793174 |
|  | 72 | 40 | 2736.807133 | 2944.802539 | 3211.308917 | 3424.834436 | 3673.323786 | 3942.626929 | 4212.859537 | 4615.158423 | 4994.281783 |
|  | 73 | 50 | 2641.054411 | 2859.671481 | 3131.93163 | 3345.480461 | 3591.249077 | 3857.07973 | 4125.979269 | 4534.691774 | 4932.525021 |
|  | 74 | 60 | 2541.902622 | 2791.539833 | 3086.743672 | 3308.70151 | 3557.159716 | 3821.431541 | 4087.694385 | 4496.018522 | 4901.611498 |
|  | 75 | 70 | 2404.540368 | 2724.461462 | 3070.036253 | 3311.440678 | 3568.622285 | 3833.075178 | 4095.319572 | 4496.964096 | 4900.054942 |
|  | 76 | 80 | 2135.709499 | 2619.028969 | 3063.248216 | 3338.154484 | 3609.686636 | 3875.410877 | 4133.042687 | 4526.592414 | 4925.922624 |
|  | 77 | 90 | 1718.366693 | 2473.302784 | 3088.287632 | 3414.568729 | 3707.336038 | 3977.975883 | 4235.430509 | 4632.193982 | 5044.75085 |
| **Left**  **Putamen** | 78 | 6 | 5115.166628 | 5467.16292 | 5911.579918 | 6256.124807 | 6637.054346 | 7019.087513 | 7366.547671 | 7817.464285 | 8176.853246 |
|  | 79 | 10 | 4921.592817 | 5270.645301 | 5710.129402 | 6050.253943 | 6426.069755 | 6803.12811 | 7146.539338 | 7593.374153 | 7950.825121 |
|  | 80 | 15 | 4687.429032 | 5032.811771 | 5465.916674 | 5800.235691 | 6169.292914 | 6539.76554 | 6877.835824 | 7319.368414 | 7674.442736 |
|  | 81 | 20 | 4495.104462 | 4838.220579 | 5266.415955 | 5595.91596 | 5959.22866 | 6324.129409 | 6657.8478 | 7095.569954 | 7449.72406 |
|  | 82 | 30 | 4304.947992 | 4652.300096 | 5080.380963 | 5407.084462 | 5766.134773 | 6127.127649 | 6459.0206 | 6898.975478 | 7260.345234 |
|  | 83 | 40 | 4102.78668 | 4457.439365 | 4886.990044 | 5211.030516 | 5565.449449 | 5922.173067 | 6252.418359 | 6696.422736 | 7068.581137 |
|  | 84 | 50 | 3884.370731 | 4248.297244 | 4678.627993 | 4998.015529 | 5344.92857 | 5694.484462 | 6021.063296 | 6468.561712 | 6854.021942 |
|  | 85 | 60 | 3643.158684 | 4026.577918 | 4465.338737 | 4783.721957 | 5126.172006 | 5471.657601 | 5798.422624 | 6257.882655 | 6668.547906 |
|  | 86 | 70 | 3301.211562 | 3735.574233 | 4211.429994 | 4546.359105 | 4901.855444 | 5261.171203 | 5606.864328 | 6110.592119 | 6584.199639 |
|  | 87 | 80 | 2885.501477 | 3428.49468 | 3990.560948 | 4370.259556 | 4766.089416 | 5167.306419 | 5562.629005 | 6167.955306 | 6778.024632 |
|  | 88 | 90 | 2390.384451 | 3125.852579 | 3837.00925 | 4291.758245 | 4754.19648 | 5224.749869 | 5704.029626 | 6490.230663 | 7361.531851 |
| **Right**  **Putamen** | 89 | 6 | 5158.08374 | 5417.335582 | 5764.484922 | 6046.741386 | 6369.446125 | 6706.170308 | 7025.629484 | 7454.953082 | 7805.386052 |
|  | 90 | 10 | 4946.077424 | 5210.732821 | 5563.48034 | 5848.47974 | 6171.954949 | 6509.183797 | 6830.998469 | 7265.819236 | 7622.205812 |
|  | 91 | 15 | 4685.885047 | 4957.140681 | 5316.516203 | 5604.514061 | 5928.339944 | 6265.493176 | 6589.558767 | 7030.283119 | 7393.295496 |
|  | 92 | 20 | 4456.962601 | 4734.209974 | 5099.101929 | 5388.978065 | 5711.688987 | 6047.080265 | 6371.656715 | 6815.73988 | 7183.141549 |
|  | 93 | 30 | 4184.554265 | 4476.896681 | 4855.786403 | 5150.983838 | 5472.563 | 5805.087296 | 6130.924062 | 6581.435665 | 6956.882468 |
|  | 94 | 40 | 3914.538332 | 4227.816641 | 4627.378742 | 4932.51562 | 5257.60852 | 5592.029882 | 5923.897609 | 6387.510919 | 6776.606519 |
|  | 95 | 50 | 3675.742075 | 4005.71465 | 4418.868265 | 4727.50897 | 5048.570344 | 5376.771703 | 5706.370595 | 6171.048368 | 6563.312956 |
|  | 96 | 60 | 3470.241765 | 3814.795079 | 4237.423018 | 4545.77495 | 4858.601376 | 5176.192918 | 5498.883164 | 5957.712787 | 6347.030443 |
|  | 97 | 70 | 3186.251451 | 3568.71887 | 4028.008398 | 4355.195791 | 4678.87946 | 5005.362934 | 5341.194992 | 5822.939924 | 6233.885576 |
|  | 98 | 80 | 2810.202653 | 3278.119213 | 3826.24627 | 4206.549326 | 4572.835478 | 4939.557995 | 5321.245922 | 5873.088933 | 6345.873198 |
|  | 99 | 90 | 2342.490471 | 2967.266576 | 3673.532058 | 4147.472526 | 4590.049794 | 5028.640168 | 5489.536454 | 6159.384652 | 6734.312771 |
| **Left**  **Pallidum** | 100 | 6 | 1454.531934 | 1599.160086 | 1770.478342 | 1898.37257 | 2038.832313 | 2183.010491 | 2321.111062 | 2517.281308 | 2693.947064 |
|  | 101 | 10 | 1360.753253 | 1500.292004 | 1665.264928 | 1788.169456 | 1922.854684 | 2060.729266 | 2192.38412 | 2378.643344 | 2545.58911 |
|  | 102 | 15 | 1252.607627 | 1386.270535 | 1543.86962 | 1660.952004 | 1788.886282 | 1919.388167 | 2043.511805 | 2218.222756 | 2373.891244 |
|  | 103 | 20 | 1174.959709 | 1305.625188 | 1459.217038 | 1572.971536 | 1696.887027 | 1822.825453 | 1942.125521 | 2109.184444 | 2257.154407 |
|  | 104 | 30 | 1119.458401 | 1255.364382 | 1413.937959 | 1530.554856 | 1656.732072 | 1783.971832 | 1903.500402 | 2069.140326 | 2214.124206 |
|  | 105 | 40 | 1057.085588 | 1198.28504 | 1361.498322 | 1480.519058 | 1608.317909 | 1736.109572 | 1855.110614 | 2018.27613 | 2159.414436 |
|  | 106 | 50 | 991.0108789 | 1138.035511 | 1305.988993 | 1427.244334 | 1556.328188 | 1684.233295 | 1802.255833 | 1962.341043 | 2099.191805 |
|  | 107 | 60 | 940.6015351 | 1097.389943 | 1273.862641 | 1399.761354 | 1532.498981 | 1662.737061 | 1781.770832 | 1941.466884 | 2076.392354 |
|  | 108 | 70 | 877.5156353 | 1044.215391 | 1228.399668 | 1357.959968 | 1493.08281 | 1624.262985 | 1742.970793 | 1900.468913 | 2031.994339 |
|  | 109 | 80 | 809.6330663 | 987.9690969 | 1180.575998 | 1313.824725 | 1451.112692 | 1582.882571 | 1700.895301 | 1855.719135 | 1983.523134 |
|  | 110 | 90 | 770.6948892 | 971.4569893 | 1182.58688 | 1325.843285 | 1471.450292 | 1609.503492 | 1731.820359 | 1890.476736 | 2019.950549 |
| **Right**  **Pallidum** | 111 | 6 | 1414.889417 | 1542.07547 | 1684.389797 | 1786.021142 | 1894.462225 | 2003.694389 | 2107.527633 | 2255.38053 | 2390.047039 |
|  | 112 | 10 | 1342.313387 | 1460.157532 | 1593.849646 | 1690.406198 | 1794.297294 | 1899.699331 | 2000.452731 | 2144.649152 | 2276.575162 |
|  | 113 | 15 | 1261.429599 | 1368.861463 | 1492.880721 | 1583.764045 | 1682.638051 | 1783.93064 | 1881.51596 | 2022.215543 | 2151.823758 |
|  | 114 | 20 | 1211.500509 | 1311.319357 | 1428.753517 | 1516.222983 | 1612.604683 | 1712.496105 | 1809.670098 | 1951.135747 | 2082.67308 |
|  | 115 | 30 | 1196.63002 | 1290.722693 | 1405.411355 | 1493.555335 | 1593.16722 | 1698.865149 | 1803.783146 | 1959.710571 | 2107.707862 |
|  | 116 | 40 | 1151.472934 | 1242.978579 | 1356.449188 | 1445.029752 | 1546.446621 | 1655.416266 | 1764.794726 | 1929.302608 | 2087.383548 |
|  | 117 | 50 | 1106.652381 | 1194.879394 | 1304.584033 | 1390.519167 | 1489.317221 | 1596.075821 | 1703.975281 | 1867.83548 | 2027.20796 |
|  | 118 | 60 | 1085.312439 | 1177.268616 | 1288.748696 | 1374.253656 | 1471.137023 | 1574.784841 | 1679.073207 | 1837.540986 | 1992.466087 |
|  | 119 | 70 | 1014.260372 | 1126.5381 | 1252.388558 | 1342.888597 | 1441.020782 | 1542.866754 | 1643.883352 | 1797.248192 | 1948.75733 |
|  | 120 | 80 | 830.1017252 | 1002.945774 | 1165.033735 | 1266.839673 | 1368.61167 | 1469.609205 | 1569.109587 | 1724.440996 | 1886.220144 |
|  | 121 | 90 | 554.4449578 | 862.5301734 | 1113.794211 | 1242.18147 | 1355.612099 | 1462.53852 | 1570.039328 | 1752.708392 | 1966.840124 |
| **Left**  **Hippocampus** | 122 | 6 | 3192.03296 | 3523.246922 | 3843.655417 | 4049.261466 | 4254.585701 | 4452.894293 | 4638.280817 | 4903.402312 | 5149.993729 |
|  | 123 | 10 | 3195.066412 | 3524.541564 | 3846.236393 | 4053.948472 | 4261.844444 | 4462.397901 | 4649.039754 | 4913.693068 | 5157.056855 |
|  | 124 | 15 | 3193.845292 | 3521.723766 | 3845.289594 | 4055.687459 | 4266.795252 | 4470.132789 | 4658.344678 | 4922.539135 | 5162.214817 |
|  | 125 | 20 | 3174.012053 | 3500.841472 | 3826.430472 | 4039.455039 | 4253.630522 | 4459.570871 | 4649.189692 | 4912.799299 | 5148.882483 |
|  | 126 | 30 | 3113.930317 | 3445.829336 | 3781.392327 | 4003.030087 | 4226.412294 | 4440.321677 | 4635.246053 | 4901.322052 | 5133.931507 |
|  | 127 | 40 | 3051.000549 | 3389.710865 | 3736.097105 | 3966.512472 | 4199.040907 | 4420.746109 | 4620.837787 | 4889.499426 | 5119.344416 |
|  | 128 | 50 | 2971.14628 | 3316.954188 | 3673.525898 | 3911.880012 | 4152.469906 | 4380.817909 | 4585.054946 | 4855.213331 | 5081.892022 |
|  | 129 | 60 | 2828.15887 | 3190.821153 | 3565.28118 | 3815.672369 | 4067.80261 | 4305.646388 | 4516.360566 | 4791.058815 | 5017.359496 |
|  | 130 | 70 | 2553.909684 | 2944.985185 | 3344.919466 | 3610.542536 | 3876.275059 | 4124.752637 | 4342.497594 | 4622.134645 | 4848.394625 |
|  | 131 | 80 | 2153.178662 | 2574.494779 | 2996.60652 | 3273.070995 | 3546.773121 | 3799.841028 | 4018.951874 | 4296.101381 | 4516.473992 |
|  | 132 | 90 | 1745.107121 | 2200.461005 | 2645.327395 | 2931.069803 | 3210.177682 | 3464.942235 | 3682.769454 | 3954.239124 | 4166.579161 |
| **Right**  **Hippocampus** | 133 | 6 | 3405.323534 | 3637.86611 | 3897.406012 | 4082.053405 | 4277.935393 | 4473.441756 | 4657.048316 | 4913.997021 | 5143.090079 |
|  | 134 | 10 | 3394.986242 | 3635.252412 | 3901.986403 | 4090.919837 | 4290.671501 | 4489.421528 | 4675.577639 | 4935.385904 | 5166.407873 |
|  | 135 | 15 | 3376.40102 | 3626.557723 | 3902.326049 | 4096.541867 | 4300.979756 | 4503.577071 | 4692.684794 | 4955.686582 | 5188.74108 |
|  | 136 | 20 | 3342.999534 | 3602.510042 | 3886.530105 | 4085.391042 | 4293.781006 | 4499.435986 | 4690.699983 | 4955.690909 | 5189.615336 |
|  | 137 | 30 | 3272.044372 | 3549.773102 | 3849.978472 | 4058.031795 | 4274.21508 | 4485.693709 | 4680.671046 | 4948.059983 | 5181.482321 |
|  | 138 | 40 | 3197.288693 | 3496.685662 | 3815.340835 | 4033.513466 | 4258.058737 | 4475.653343 | 4674.48159 | 4944.402121 | 5177.511804 |
|  | 139 | 50 | 3096.529262 | 3428.821596 | 3771.978517 | 4001.9106 | 4235.29125 | 4459.152331 | 4662.424808 | 4937.360485 | 5174.480115 |
|  | 140 | 60 | 2902.659269 | 3286.058092 | 3659.875052 | 3900.80089 | 4140.119808 | 4367.135424 | 4573.081303 | 4854.012298 | 5100.211718 |
|  | 141 | 70 | 2575.023149 | 3044.253206 | 3459.92501 | 3710.80199 | 3951.830938 | 4177.688215 | 4384.189147 | 4673.778372 | 4938.523429 |
|  | 142 | 80 | 2087.166858 | 2675.695914 | 3144.502228 | 3401.823964 | 3637.479239 | 3855.450608 | 4058.796326 | 4359.574064 | 4655.936804 |
|  | 143 | 90 | 1581.063982 | 2263.414933 | 2797.546553 | 3061.661659 | 3289.163986 | 3497.176888 | 3698.728833 | 4023.841164 | 4381.957269 |
| **Left**  **Amygdala** | 144 | 6 | 1083.018601 | 1204.015933 | 1345.06942 | 1448.431722 | 1559.458385 | 1670.026836 | 1772.141141 | 1910.207422 | 2027.439367 |
|  | 145 | 10 | 1106.628627 | 1225.185701 | 1364.425161 | 1467.178074 | 1578.297347 | 1689.828213 | 1793.702751 | 1935.629554 | 2057.576465 |
|  | 146 | 15 | 1130.226513 | 1245.520175 | 1382.054195 | 1483.643302 | 1594.414034 | 1706.698989 | 1812.419183 | 1958.87454 | 2086.717232 |
|  | 147 | 20 | 1136.995051 | 1248.2629 | 1380.966358 | 1480.444671 | 1589.772943 | 1701.688968 | 1808.239066 | 1957.989491 | 2090.923794 |
|  | 148 | 30 | 1123.398385 | 1230.265302 | 1358.349871 | 1455.044785 | 1562.34084 | 1673.732588 | 1781.675902 | 1937.184307 | 2079.459724 |
|  | 149 | 40 | 1101.182081 | 1212.717444 | 1344.842478 | 1443.779468 | 1553.206569 | 1666.961045 | 1777.85639 | 1939.554071 | 2090.047124 |
|  | 150 | 50 | 1058.369951 | 1181.783469 | 1323.635565 | 1427.284456 | 1540.01259 | 1655.804564 | 1767.983465 | 1931.298574 | 2083.695521 |
|  | 151 | 60 | 965.4344304 | 1113.26344 | 1273.074461 | 1384.28783 | 1501.115657 | 1617.78521 | 1728.552967 | 1887.21265 | 2033.428493 |
|  | 152 | 70 | 773.2579178 | 986.5564213 | 1190.508894 | 1320.03628 | 1448.101748 | 1570.002742 | 1681.784884 | 1837.284581 | 1977.190939 |
|  | 153 | 80 | 486.0997604 | 762.9372632 | 1025.483443 | 1175.77776 | 1313.10323 | 1436.141835 | 1544.405425 | 1690.261567 | 1818.378555 |
|  | 154 | 90 | 350.717871 | 585.8798481 | 864.9347528 | 1027.211404 | 1167.685522 | 1287.017596 | 1388.185224 | 1520.847511 | 1635.291622 |
| **Right**  **Amygdala** | 155 | 6 | 1210.941248 | 1300.118439 | 1413.907702 | 1503.550521 | 1604.75091 | 1709.195175 | 1807.47598 | 1940.880605 | 2052.955364 |
|  | 156 | 10 | 1215.112932 | 1308.367886 | 1426.229212 | 1518.532331 | 1622.572598 | 1730.221908 | 1832.187109 | 1972.261408 | 2091.905222 |
|  | 157 | 15 | 1214.295507 | 1313.542018 | 1436.963403 | 1532.617762 | 1640.07444 | 1751.626863 | 1858.342735 | 2007.711376 | 2138.677548 |
|  | 158 | 20 | 1198.963967 | 1303.737551 | 1431.787563 | 1529.902978 | 1639.706702 | 1754.088322 | 1864.686288 | 2022.66874 | 2165.193979 |
|  | 159 | 30 | 1176.333036 | 1279.577909 | 1407.475906 | 1506.666382 | 1618.780869 | 1736.671904 | 1851.595501 | 2017.123523 | 2167.658439 |
|  | 160 | 40 | 1174.842448 | 1271.06085 | 1395.462237 | 1495.110125 | 1609.833876 | 1731.299198 | 1849.019639 | 2015.098663 | 2161.053145 |
|  | 161 | 50 | 1151.369553 | 1250.574738 | 1379.629124 | 1483.190043 | 1602.053016 | 1726.844573 | 1846.194666 | 2011.114763 | 2152.205336 |
|  | 162 | 60 | 1076.977241 | 1193.143272 | 1337.477981 | 1448.640872 | 1572.045076 | 1697.70501 | 1814.915201 | 1973.198952 | 2106.022288 |
|  | 163 | 70 | 912.7378544 | 1083.548063 | 1265.488694 | 1390.335736 | 1519.067559 | 1643.940748 | 1758.026289 | 1912.682842 | 2045.926155 |
|  | 164 | 80 | 557.2242869 | 866.5992107 | 1137.260692 | 1284.413213 | 1417.415506 | 1540.289733 | 1656.337818 | 1832.714197 | 2013.019998 |
|  | 165 | 90 | 375.6423394 | 665.1482319 | 1003.786131 | 1174.298522 | 1308.234264 | 1432.599264 | 1574.438394 | 1881.880392 | 2341.098861 |
| **Left**  **Nucleus**  **Accumbens** | 166 | 6 | 497.9355322 | 543.8842851 | 608.8664247 | 664.8825546 | 732.7345011 | 807.0868525 | 880.1303222 | 982.3468014 | 1069.479548 |
|  | 167 | 10 | 460.6164344 | 505.5428009 | 568.7646679 | 623.0028975 | 688.4257116 | 759.8271641 | 829.7344596 | 927.2631165 | 1010.203527 |
|  | 168 | 15 | 419.3481465 | 463.6740272 | 525.4411668 | 577.9737559 | 640.9162187 | 709.2422683 | 775.9166889 | 868.8097429 | 947.8855279 |
|  | 169 | 20 | 388.8887849 | 434.3118218 | 496.5309261 | 548.7089293 | 610.63514 | 677.4649896 | 742.5957466 | 833.6887 | 911.9388769 |
|  | 170 | 30 | 352.456032 | 405.760535 | 474.5615799 | 529.6142131 | 593.0932573 | 660.6653376 | 726.770418 | 821.4876372 | 906.4556302 |
|  | 171 | 40 | 313.1964837 | 371.6514849 | 444.3167328 | 500.6521369 | 564.1905699 | 630.7914336 | 695.4871894 | 788.2582942 | 872.1845604 |
|  | 172 | 50 | 285.9677343 | 342.7363299 | 414.5903765 | 470.7726703 | 533.9056577 | 598.9661817 | 660.3299599 | 744.21395 | 815.4920325 |
|  | 173 | 60 | 255.6383801 | 314.5821178 | 389.9797523 | 449.0770298 | 515.0094027 | 581.7332646 | 642.9353055 | 723.1023961 | 787.6112271 |
|  | 174 | 70 | 194.1408832 | 261.5937435 | 346.0183587 | 410.8751122 | 481.8811823 | 552.2088639 | 615.2569457 | 695.5562277 | 758.1659176 |
|  | 175 | 80 | 110.6678365 | 189.7155307 | 286.6122592 | 358.2094607 | 434.1925762 | 507.3519122 | 571.3628401 | 650.89285 | 711.4172584 |
|  | 176 | 90 | 54.2953935 | 127.2445293 | 234.5591702 | 313.688571 | 395.0490775 | 470.9554487 | 535.6830354 | 614.2030371 | 672.6932235 |
| **Right**  **Nucleus**  **Accumbens** | 177 | 6 | 529.2730042 | 576.3561206 | 639.835888 | 691.6159882 | 750.8096493 | 813.0210155 | 872.8943697 | 954.448558 | 1021.754033 |
|  | 178 | 10 | 502.6840424 | 549.6651341 | 612.8583539 | 664.2359621 | 722.7458771 | 784.1711427 | 843.3977911 | 924.1924992 | 990.931091 |
|  | 179 | 15 | 471.0453143 | 517.9313115 | 580.8046173 | 631.7072561 | 689.3941621 | 749.8668213 | 808.3063186 | 888.1698658 | 954.2072736 |
|  | 180 | 20 | 443.0801108 | 490.0911868 | 552.942645 | 603.6152519 | 660.7633654 | 720.5866732 | 778.5328969 | 857.8689612 | 923.542 |
|  | 181 | 30 | 402.5961083 | 451.1755019 | 515.7729052 | 567.4488927 | 625.1942064 | 685.5075826 | 744.2363907 | 824.993747 | 892.0330717 |
|  | 182 | 40 | 367.3395952 | 418.3243721 | 485.6617408 | 539.0376519 | 598.0617102 | 659.500224 | 719.5761037 | 802.4431534 | 871.3445087 |
|  | 183 | 50 | 329.8004061 | 384.4735758 | 455.6815173 | 511.2362441 | 571.6865059 | 634.052609 | 694.9839535 | 778.826174 | 848.2704222 |
|  | 184 | 60 | 288.0760998 | 348.6706669 | 425.5528619 | 483.9897918 | 546.0753837 | 609.122697 | 670.2949528 | 753.7285825 | 822.1546626 |
|  | 185 | 70 | 236.9919819 | 306.4633455 | 390.6522703 | 452.1442806 | 515.3792782 | 578.1554368 | 638.3199268 | 719.2369259 | 784.6511621 |
|  | 186 | 80 | 173.8973594 | 257.0604975 | 351.1176132 | 415.8745757 | 479.6304671 | 541.0437975 | 598.8716922 | 675.2086037 | 735.8183122 |
|  | 187 | 90 | 116.3025037 | 209.7914938 | 316.1223775 | 385.156562 | 449.8743212 | 510.1520979 | 565.7635253 | 637.6802508 | 693.7059895 |

The process for delivering centile curves that could be reliably used as normative reference involves multiple steps relating to the assessment of measurement error and the exploration of their functional significance. This is the focus of ongoing research efforts by our group. We provide the centile curves here to enable other researchers in the field to undertake such analyses.

Below we provide initial evidence of the performance of the ENIGMA-Lifespan Centile values against centile values derived directly from the S1200 release of the (Human Connectome Project (HCP) (www.humanconnectome.org) (n=1113; age range 22-37 years ) and the stage-2 release of the Cambridge Centre for Ageing and Neuroscience Project (Cam-CAN Project) (www.mrc-cbu.cam.ac.uk) ( n=652; age range 18-87 years). These centile values were derived using the same approach as for the ENIGMA Lifespan centile curves.

These studies did not contribute data to the ENIGMA-Lifespan. Measurement error is an estimate of accuracy and was computed at each centile using the following formula |(C_E_-C_s_)/ C_s_| x 100; where C_E_ is the value of the ENIGMA Lifespan centile and C_s_ the value of any other sample at the same centile. In this illustrative example the error is below 10% and commonly around 4%.

| **Table S8. Percentage Measurement Error Per Centile** | | | | | | | | | | | | | |
| --- | --- | --- | --- | --- | --- | --- | --- | --- | --- | --- | --- | --- | --- |
| **Age** | **C0.4** | **C1** | **C2.5** | **C5** | **C10** | **C25** | **C50** | **C75** | **C90** | **C95** | **C97.5** | **C99** | **C99.6** |
| **Measurement Error in Cam-CAN** | | | | | | | | | | | | | |
| 20 | 3.54 | 0.94 | 0.95 | 1.98 | 2.72 | 3.40 | 3.95 | 4.01 | 3.31 | 2.65 | 2.00 | 1.21 | 0.53 |
| 30 | 2.82 | 0.81 | 0.68 | 1.50 | 2.11 | 2.73 | 3.33 | 3.50 | 2.89 | 2.29 | 1.69 | 0.94 | 0.27 |
| 40 | 2.78 | 0.90 | 0.50 | 1.29 | 1.90 | 2.56 | 3.24 | 3.47 | 2.89 | 2.28 | 1.65 | 0.85 | 0.11 |
| **Measurement Error in the HCP** | | | | | | | | | | | | | |
| 20 | 8.85 | 6.35 | 4.53 | 3.56 | 2.87 | 2.34 | 2.02 | 2.17 | 3.00 | 3.69 | 4.37 | 5.17 | 5.86 |
| 30 | 9.84 | 7.78 | 6.21 | 5.31 | 4.64 | 4.03 | 3.67 | 3.62 | 3.88 | 4.07 | 4.21 | 4.31 | 4.29 |
| 40 | 8.20 | 6.66 | 5.60 | 5.09 | 4.83 | 4.87 | 5.22 | 5.69 | 6.09 | 6.26 | 6.32 | 6.28 | 6.10 |
