# Supplementary material for: Subcortical volumes across the lifespan: Data from 18,605 healthy individuals aged 3–90 years
Source: Hum Brain Mapp. 2021 Feb 11;43(1):452–69. doi: 10.1002/hbm.25320 (PMC8675429; doi:10.1002/hbm.25320)
Supplement: Supplementary file 1 — Appendix S1: Supplementary Information [file HBM-43-452-s001.zip › HBM_25320_Supplemental Figures without ICV correction.pdf]

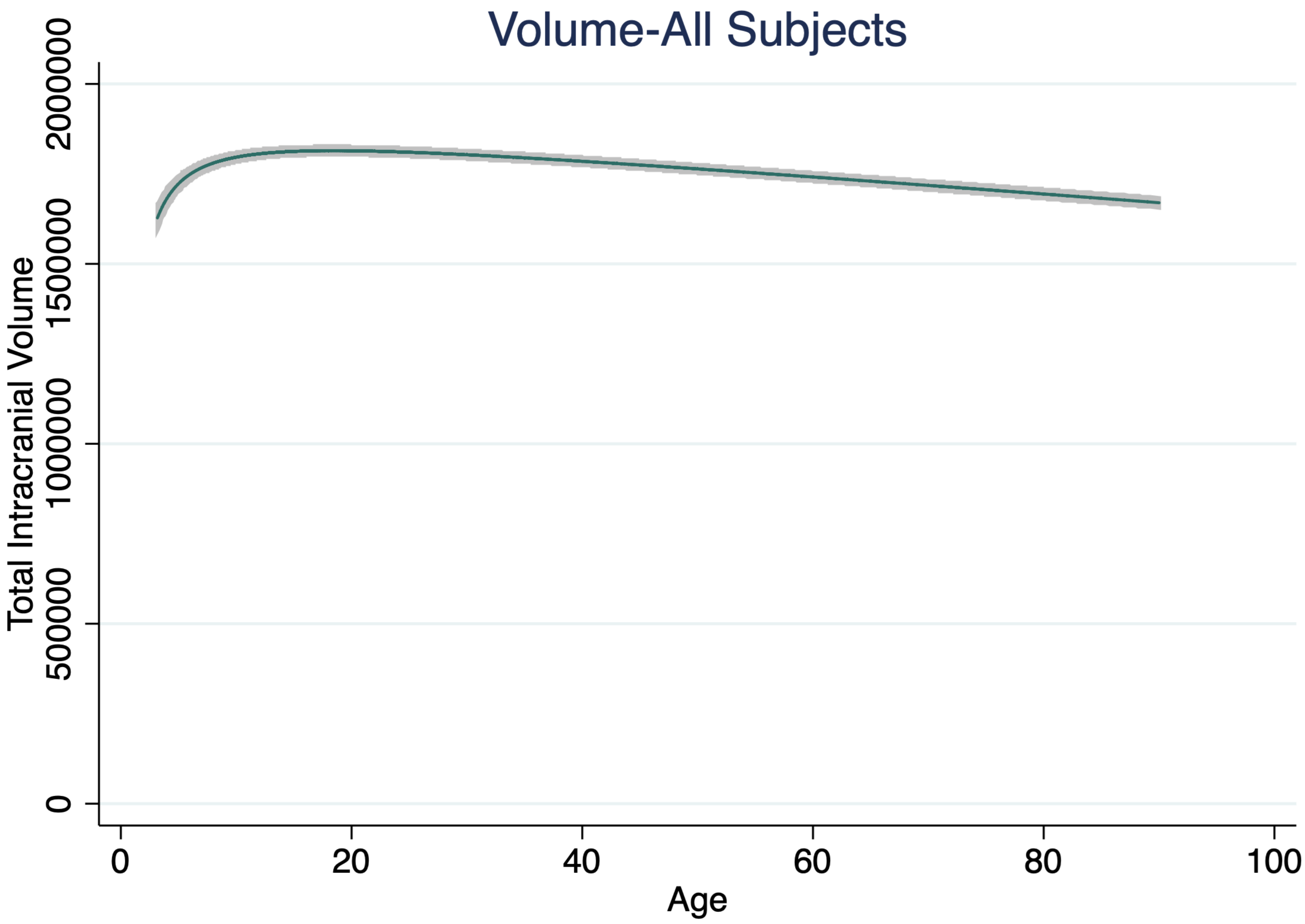
Volume-All Subjects
Total Intracranial Volume
0
500000
1000000
1500000
2000000
0
20
40
60
80
100
Age

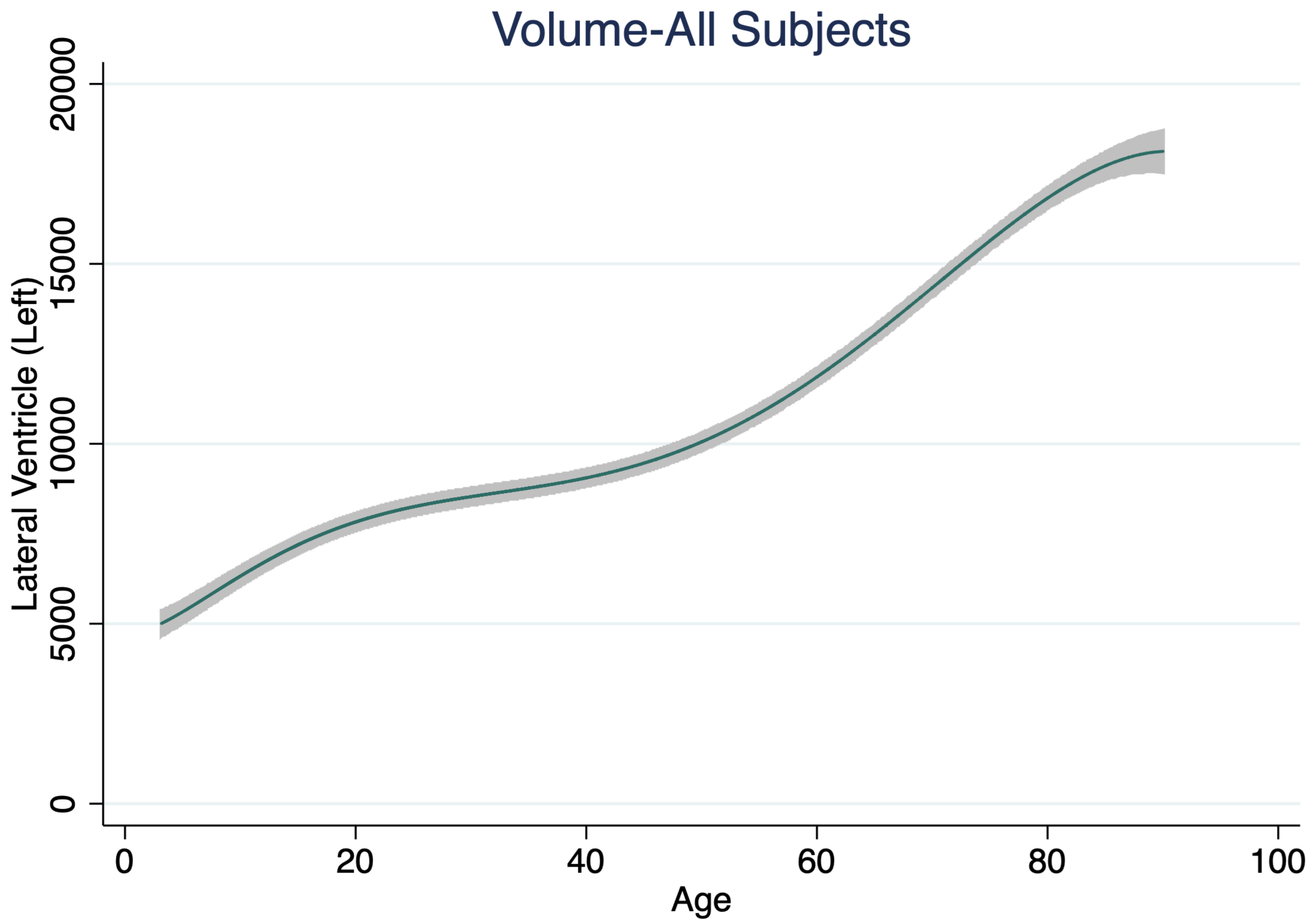
Volume-All Subjects
Lateral Ventricle (Left)
0
5000
10000
15000
20000
0
20
40
60
80
100
Age

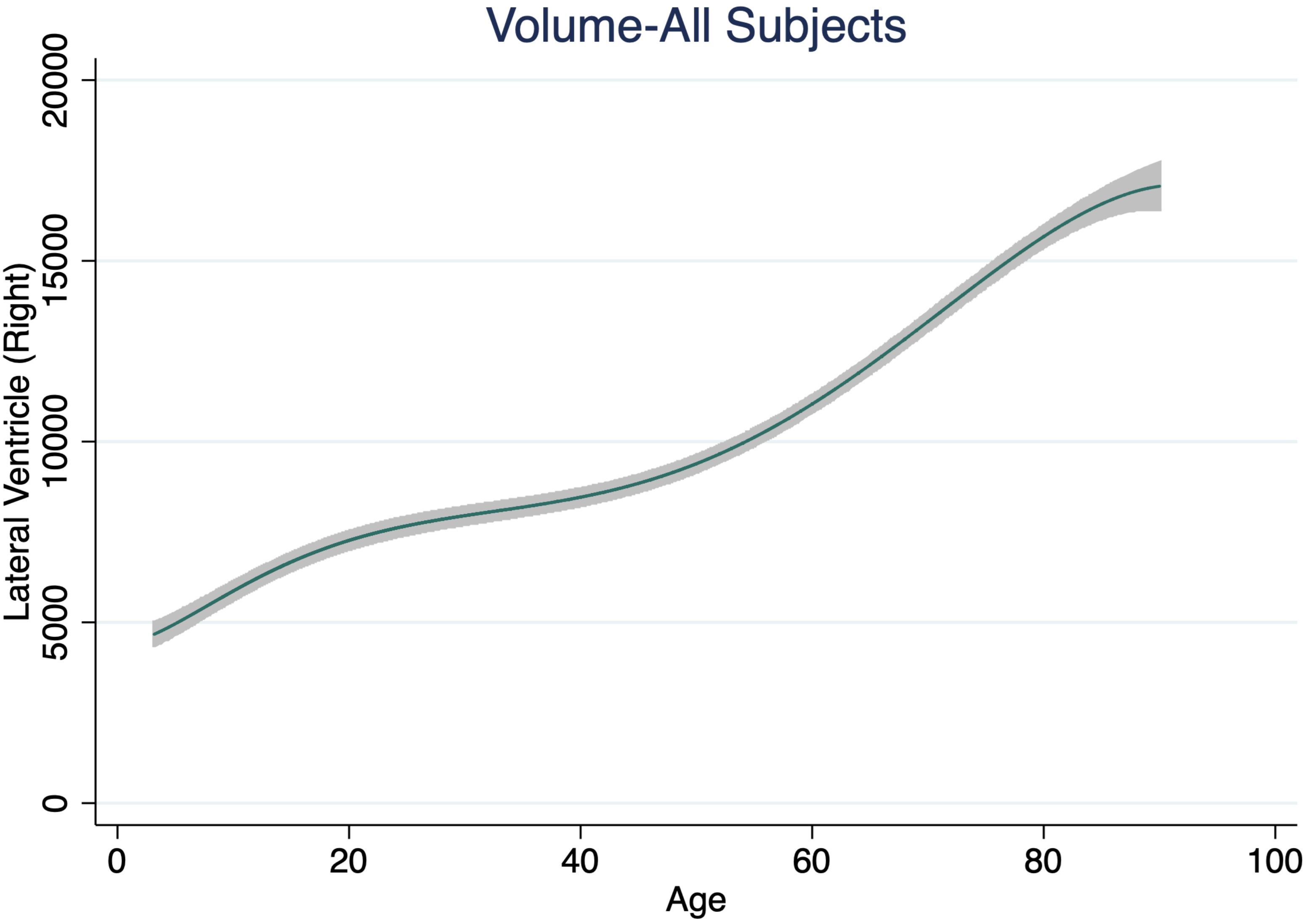
Volume-All Subjects
Lateral Ventricle (Right)
0
5000
10000
15000
20000
0
20
40
60
80
100
Age

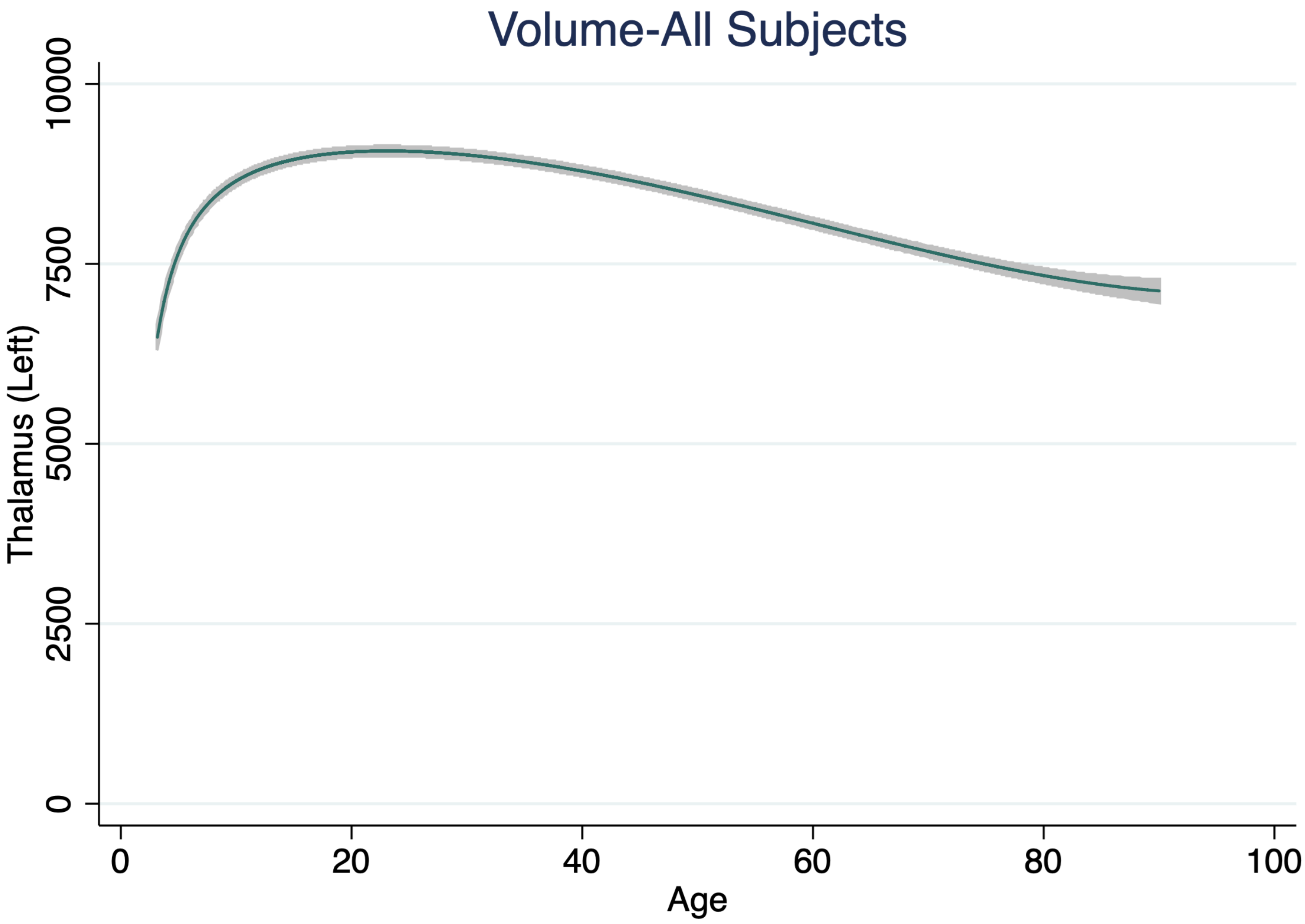

Volume-All Subjects
Thalamus (Left)
10000
7500
5000
2500
0
0
20
40
60
80
100
Age

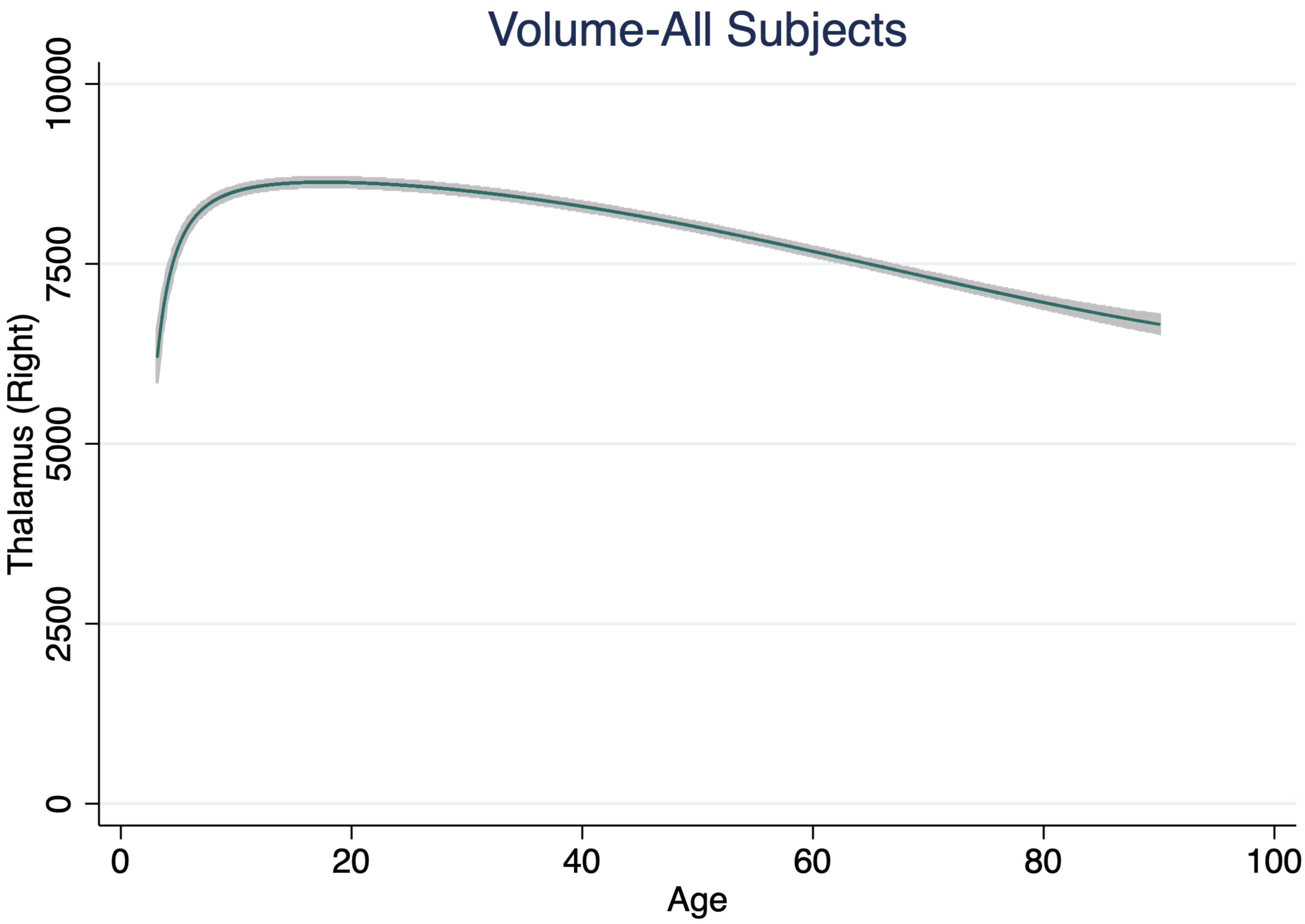

Volume-All Subjects
Thalamus (Right)
0
2500
5000
7500
10000
0
20
40
60
80
100
Age

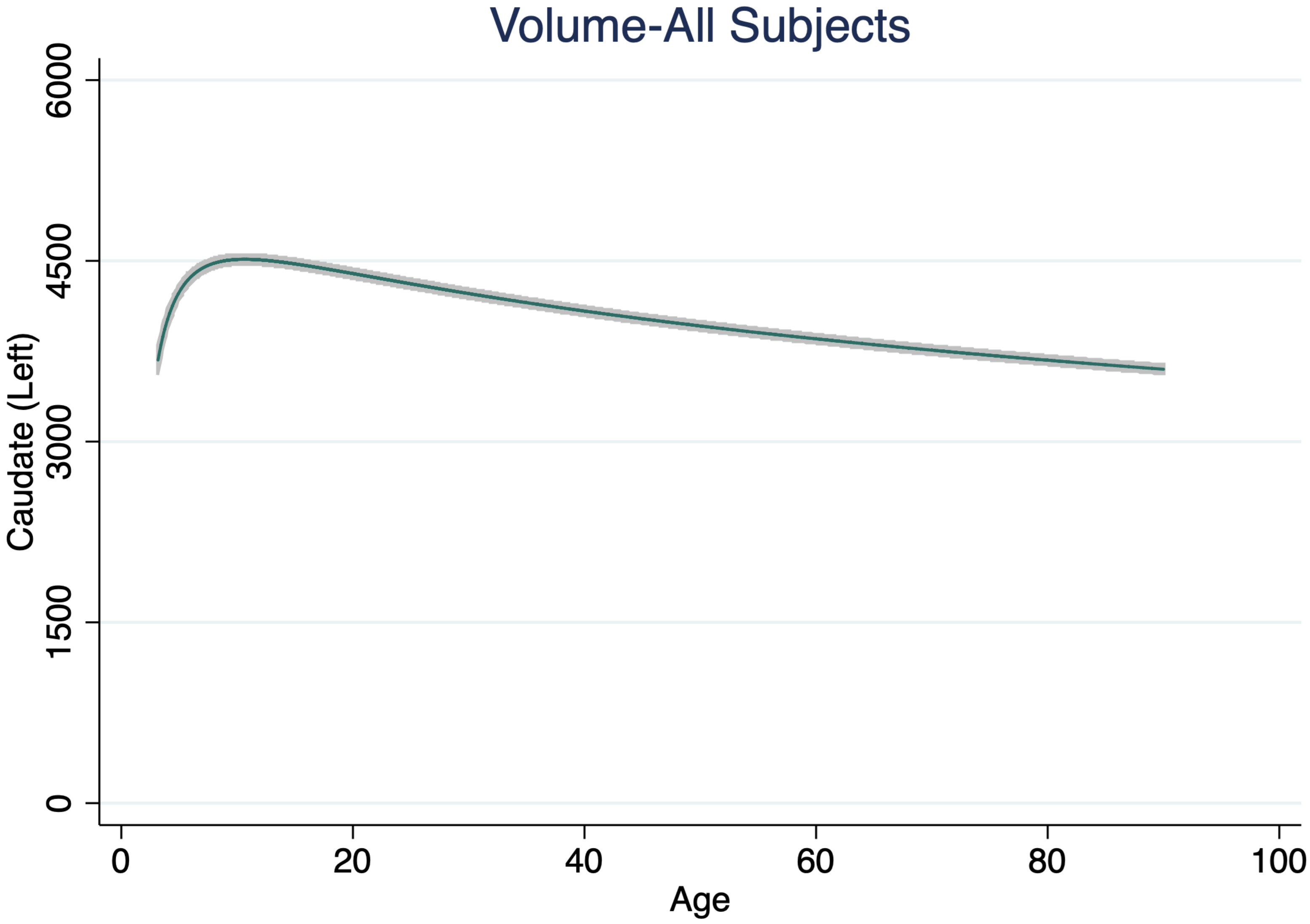

Volume-All Subjects
6000
4500
3000
1500
0
Caudate (Left)
0
20
40
60
80
100
Age

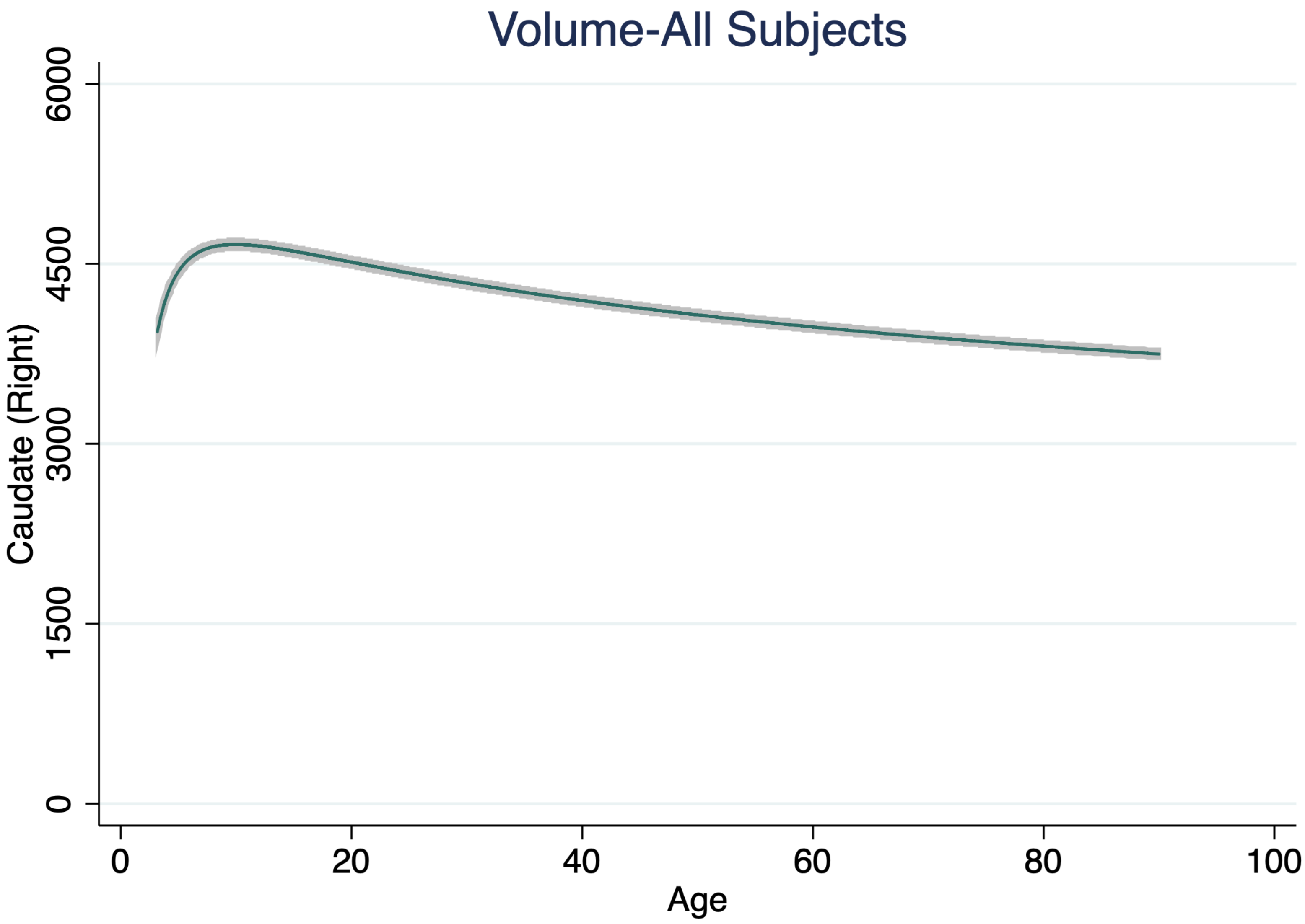
Volume-All Subjects
Caudate (Right)
6000
4500
3000
1500
0
0
20
40
60
80
100
Age

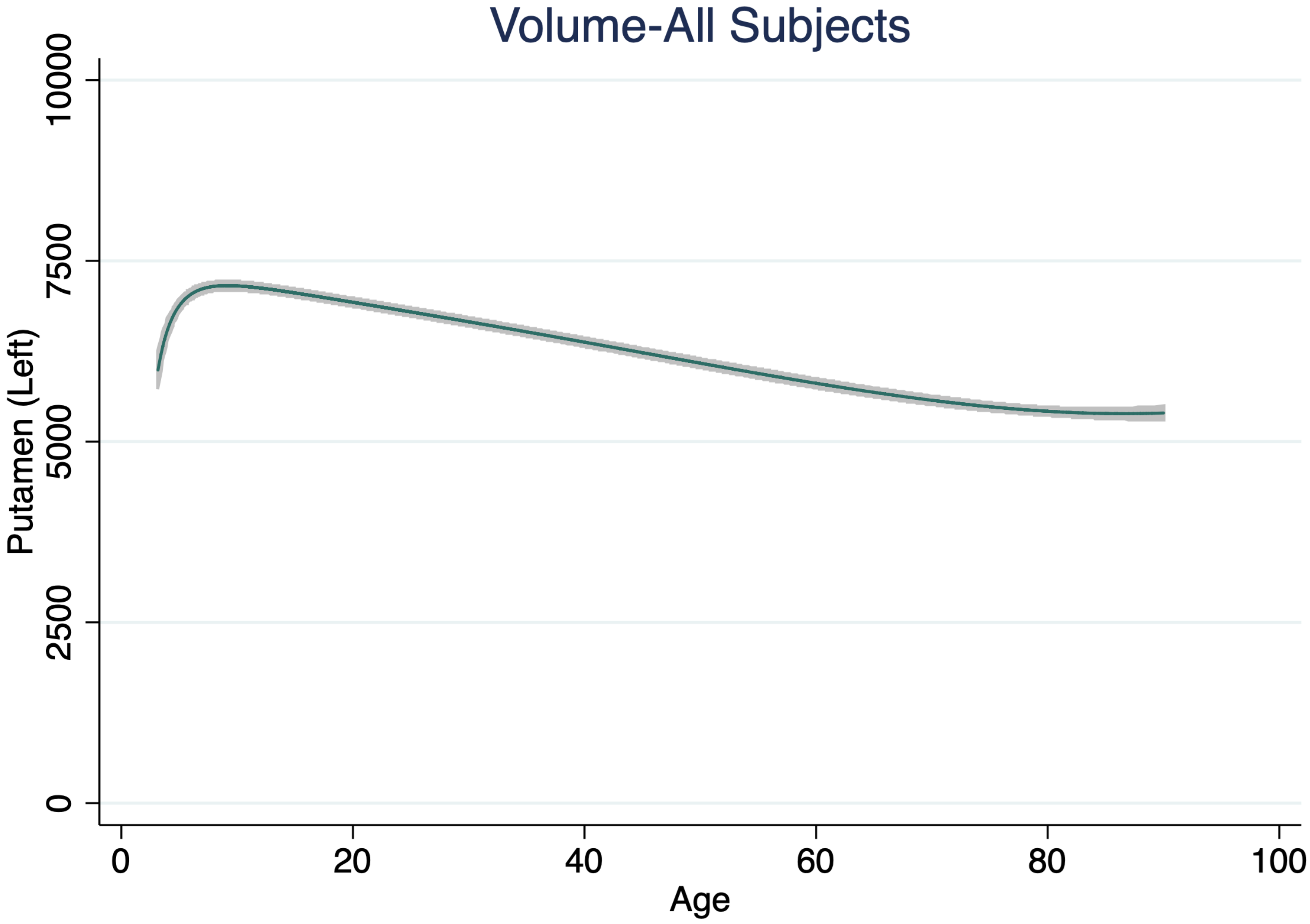

Volume-All Subjects
Putamen (Left)
10000
7500
5000
2500
0
0
20
40
60
80
100
Age

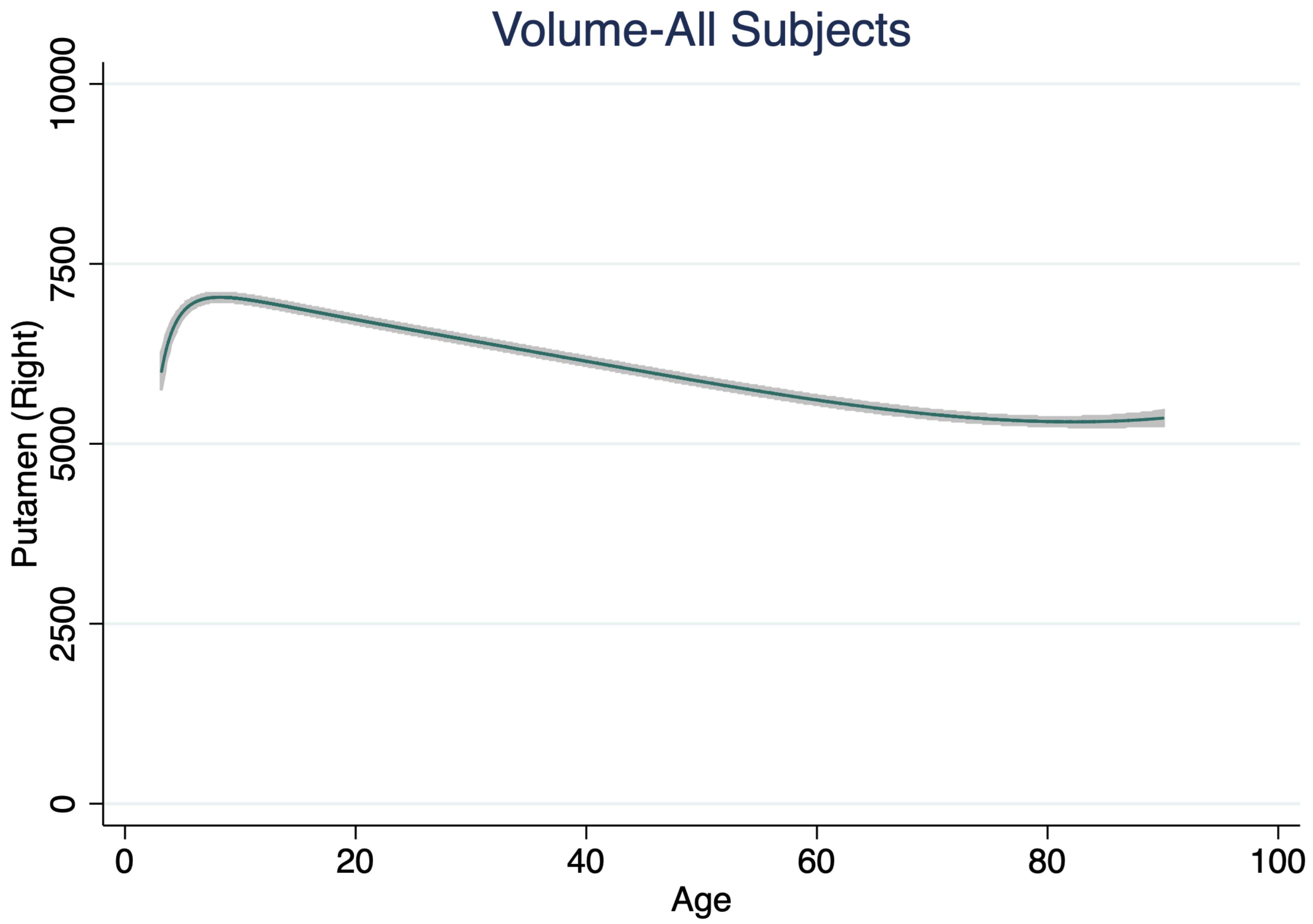
Volume-All Subjects
10000
7500
5000
2500
0
Putamen (Right)
0
20
40
60
80
100
Age

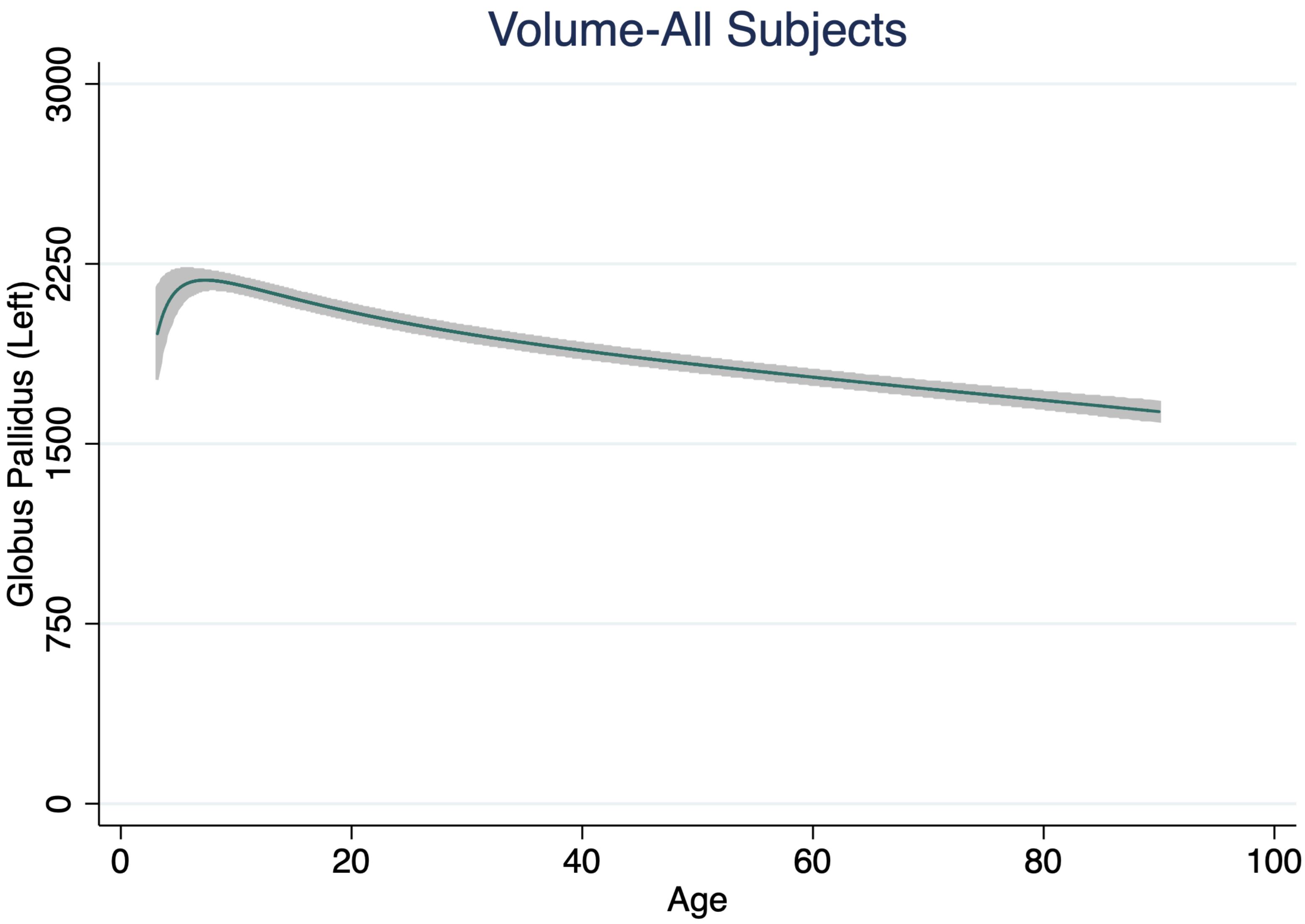

Volume-All Subjects
Globus Pallidus (Left)
0
750
1500
2250
3000
Age
0
20
40
60
80
100

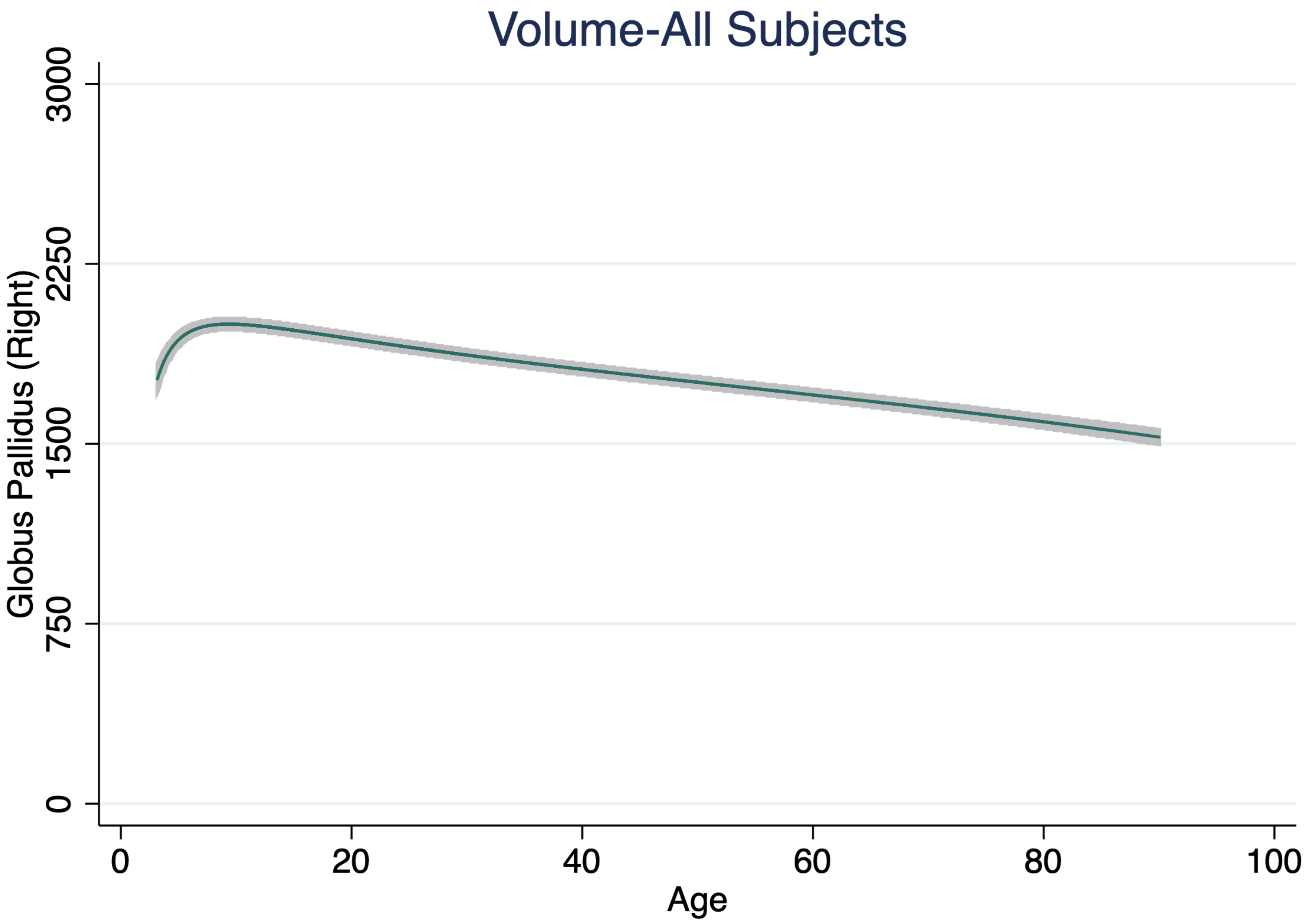
Volume-All Subjects
Globus Pallidus (Right)
0
750
1500
2250
3000
0
20
40
60
80
100
Age

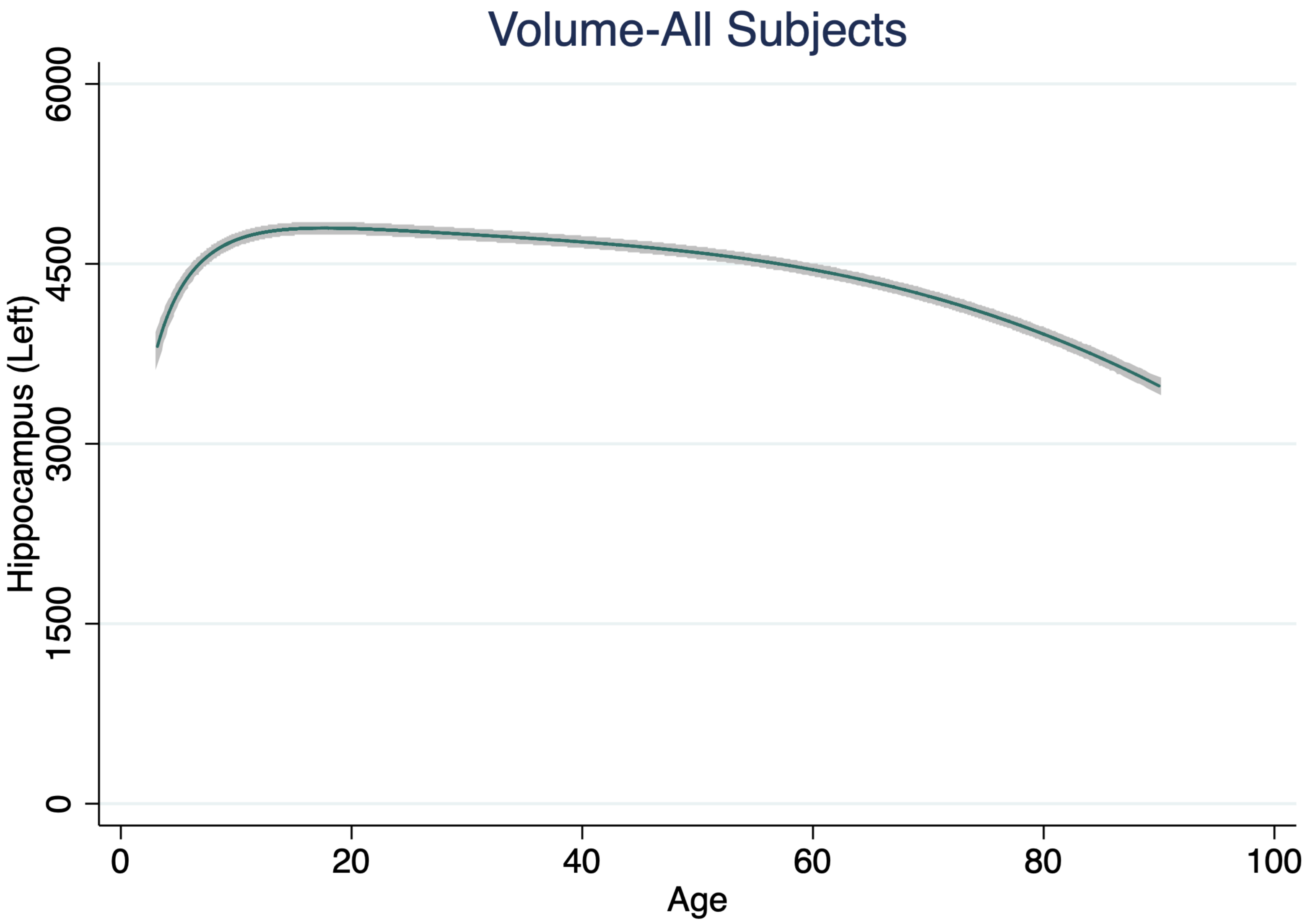

Volume-All Subjects
Hippocampus (Left)
6000
4500
3000
1500
0
0
20
40
60
80
100
Age

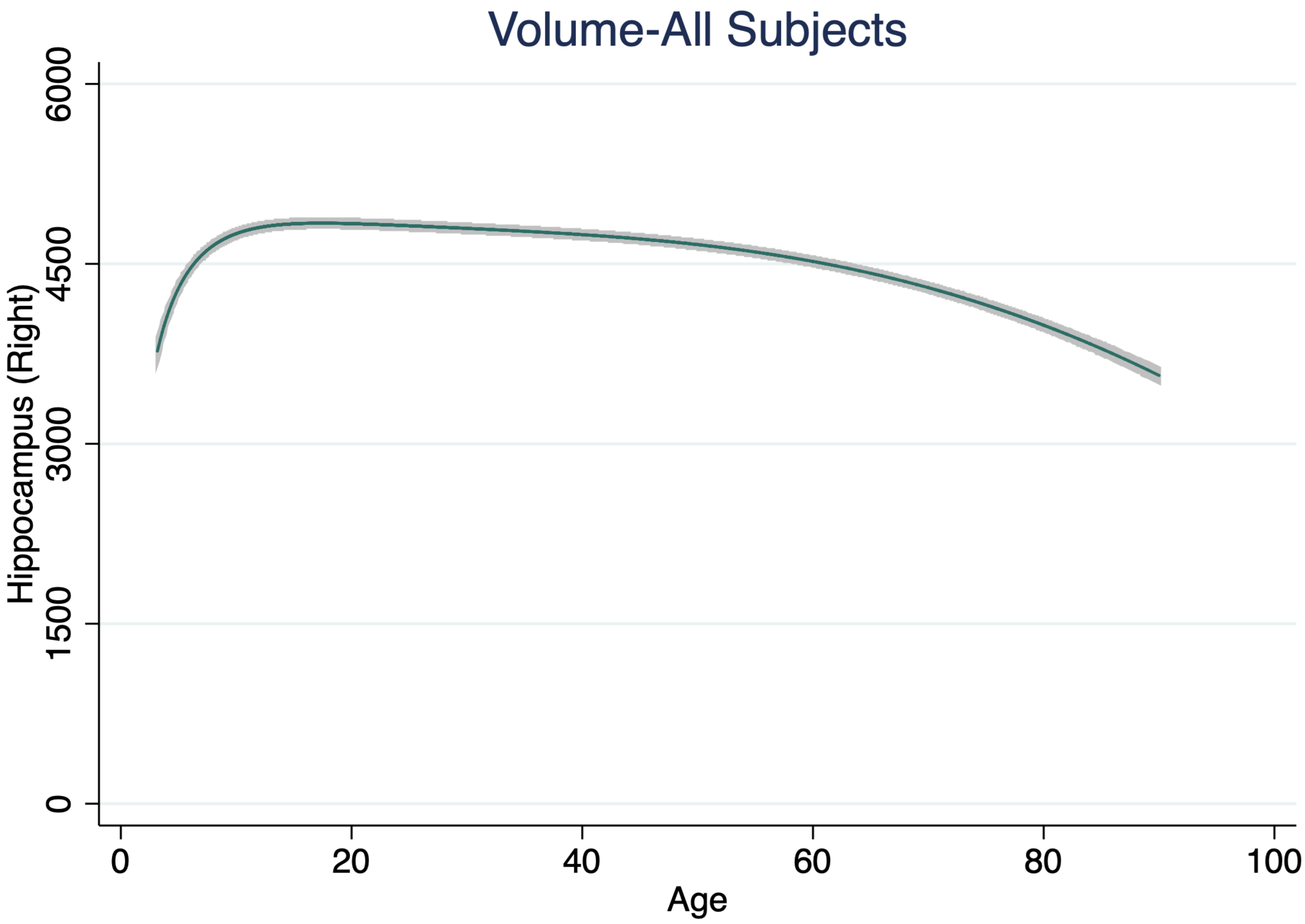
Volume-All Subjects
Hippocampus (Right)
0
1500
3000
4500
6000
Age
0
20
40
60
80
100

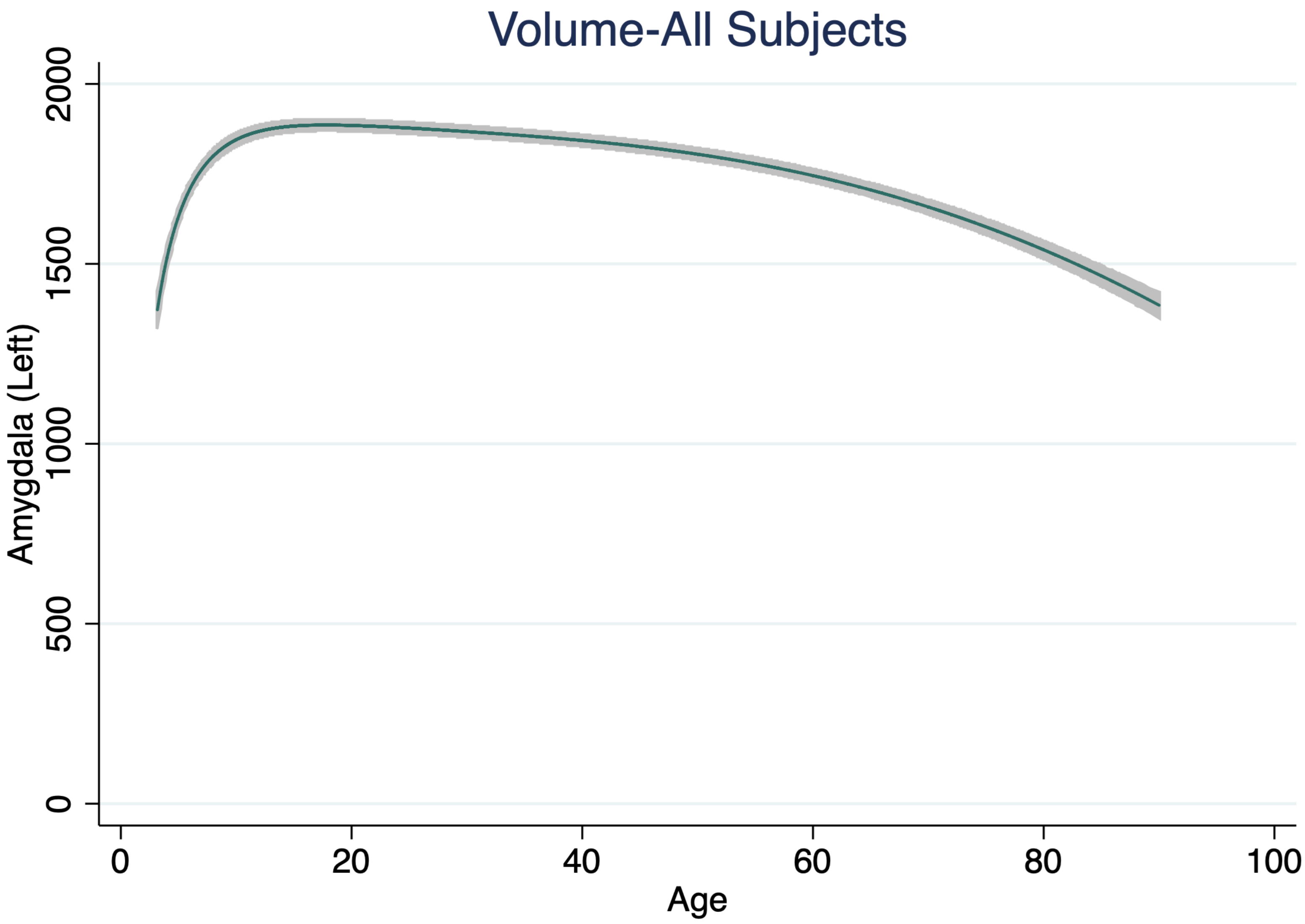
Volume-All Subjects
Amygdala (Left)
2000
1500
1000
500
0
0
20
40
60
80
100
Age

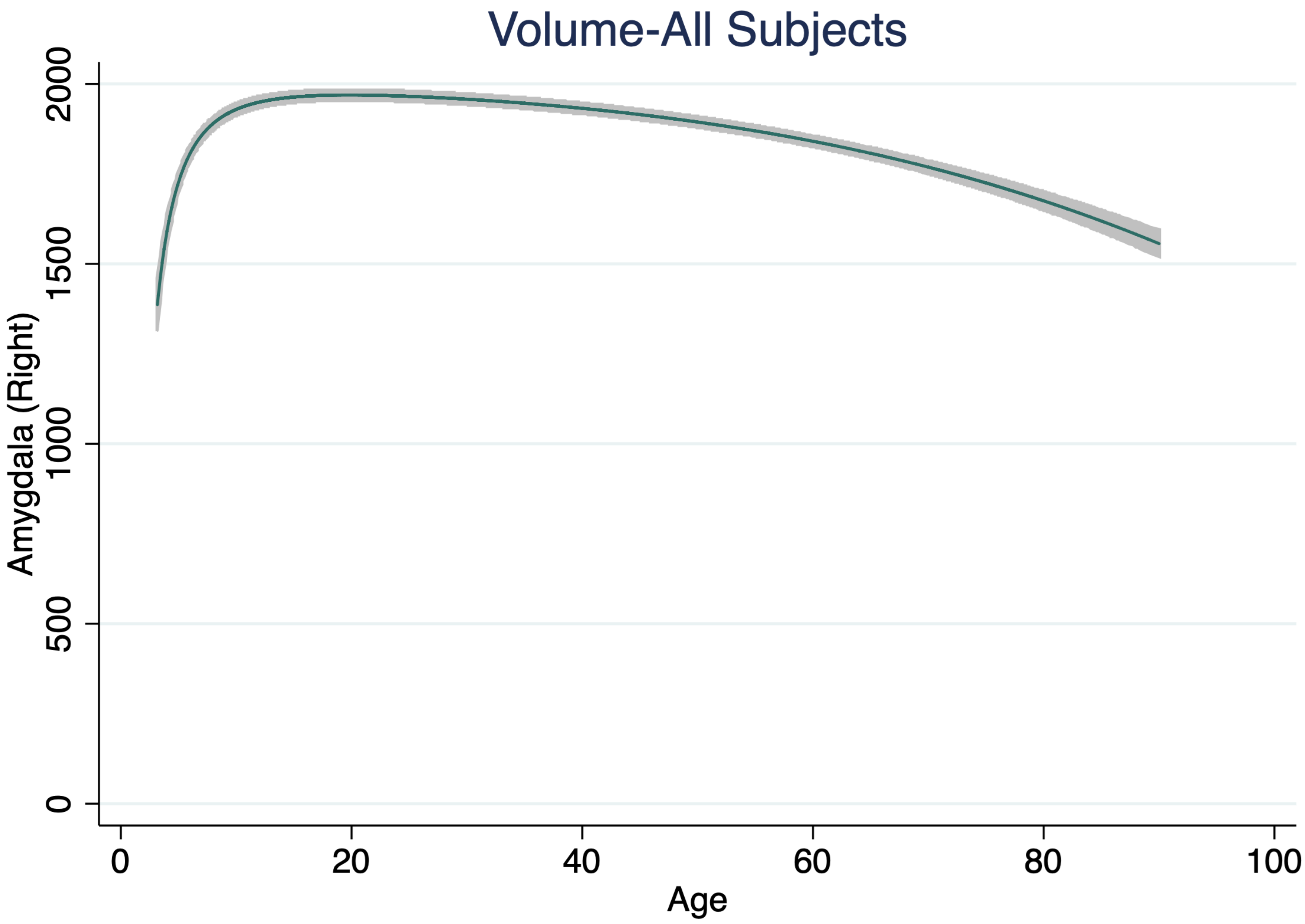

Volume-All Subjects
Amygdala (Right)
0
500
1000
1500
2000
0
20
40
60
80
100
Age

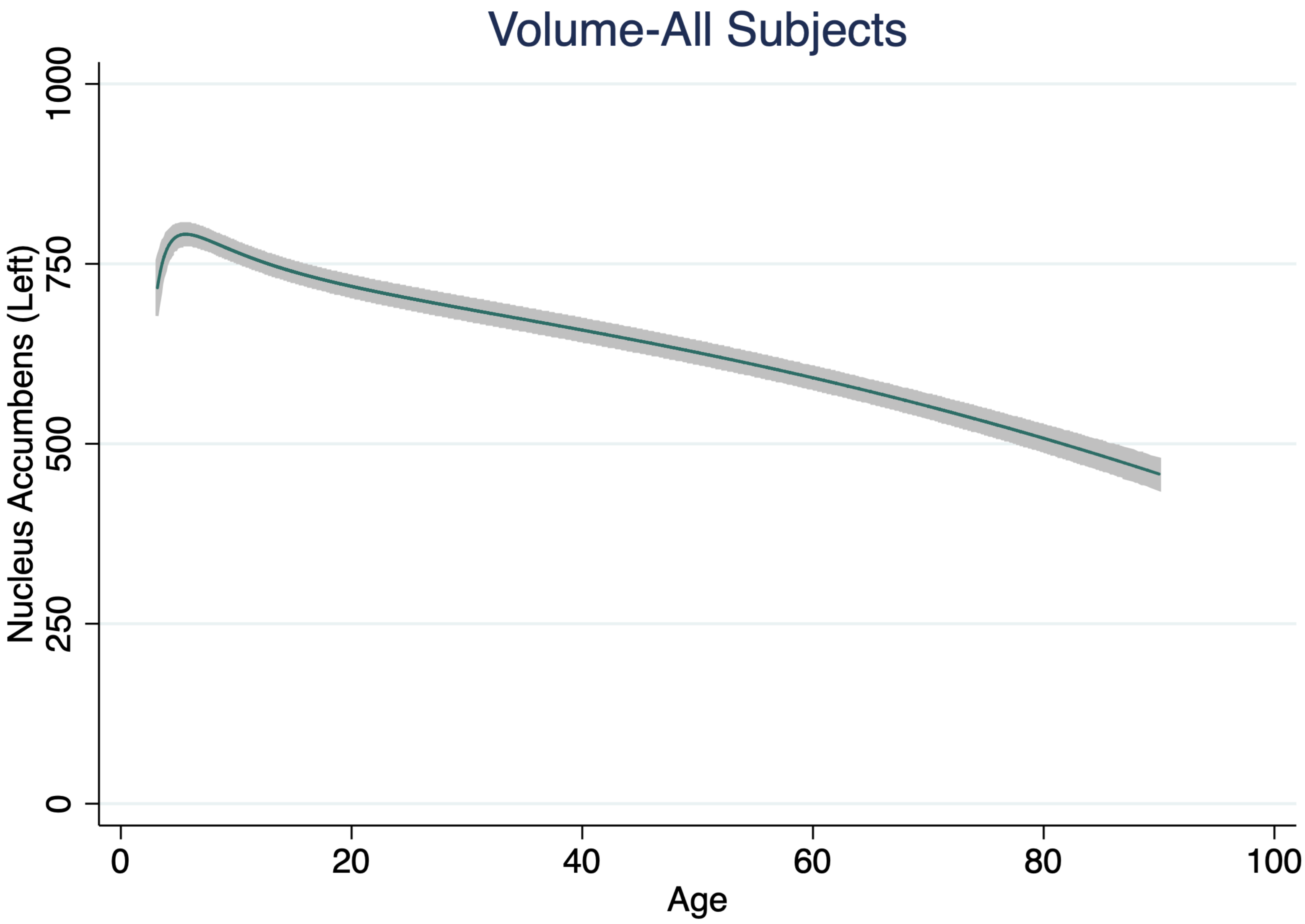
Volume-All Subjects
Nucleus Accumbens (Left)
1000
750
500
250
0
0
20
40
60
80
100
Age

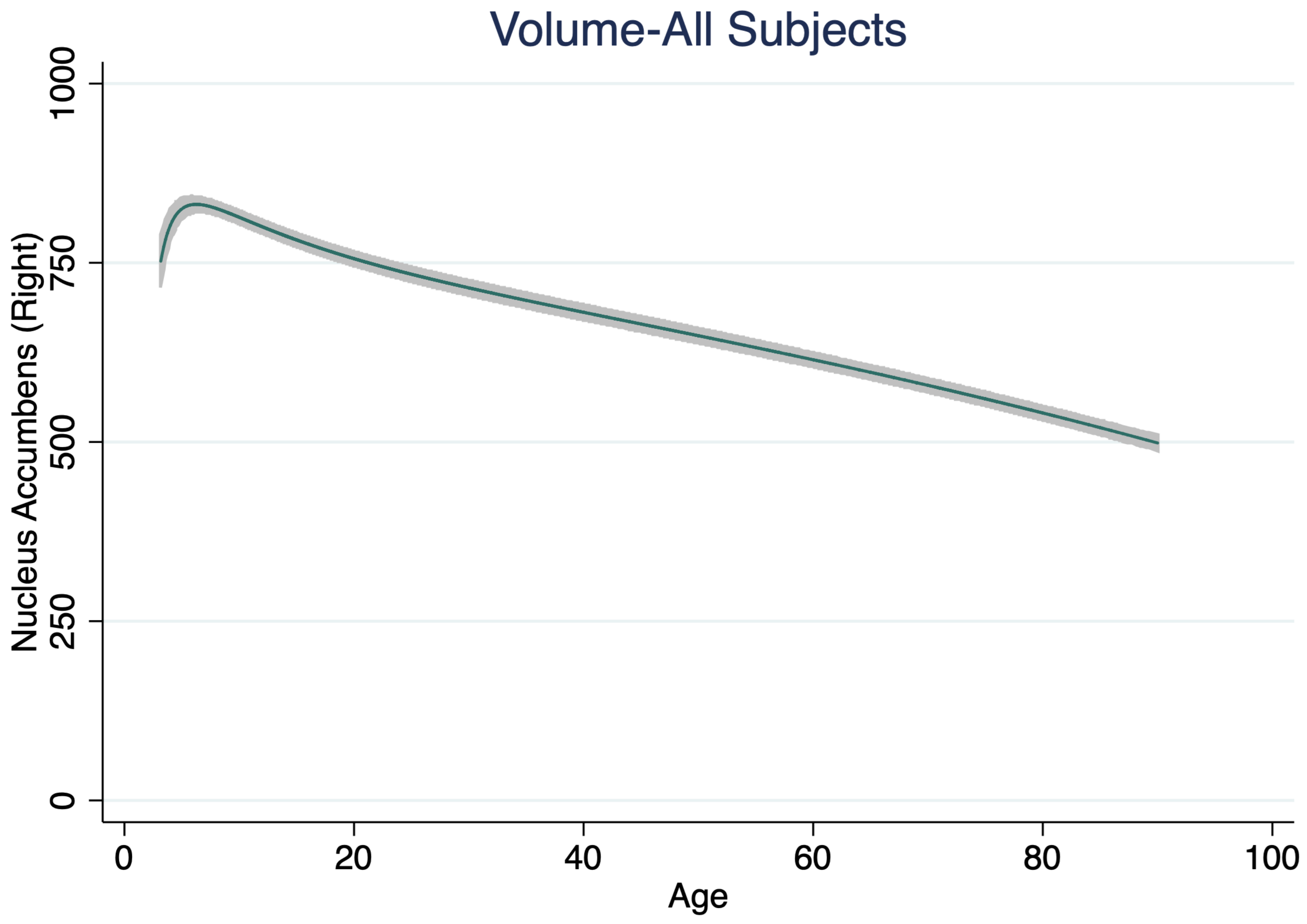
Volume-All Subjects
Nucleus Accumbens (Right)
1000
750
500
250
0
0
20
40
60
80
100
Age

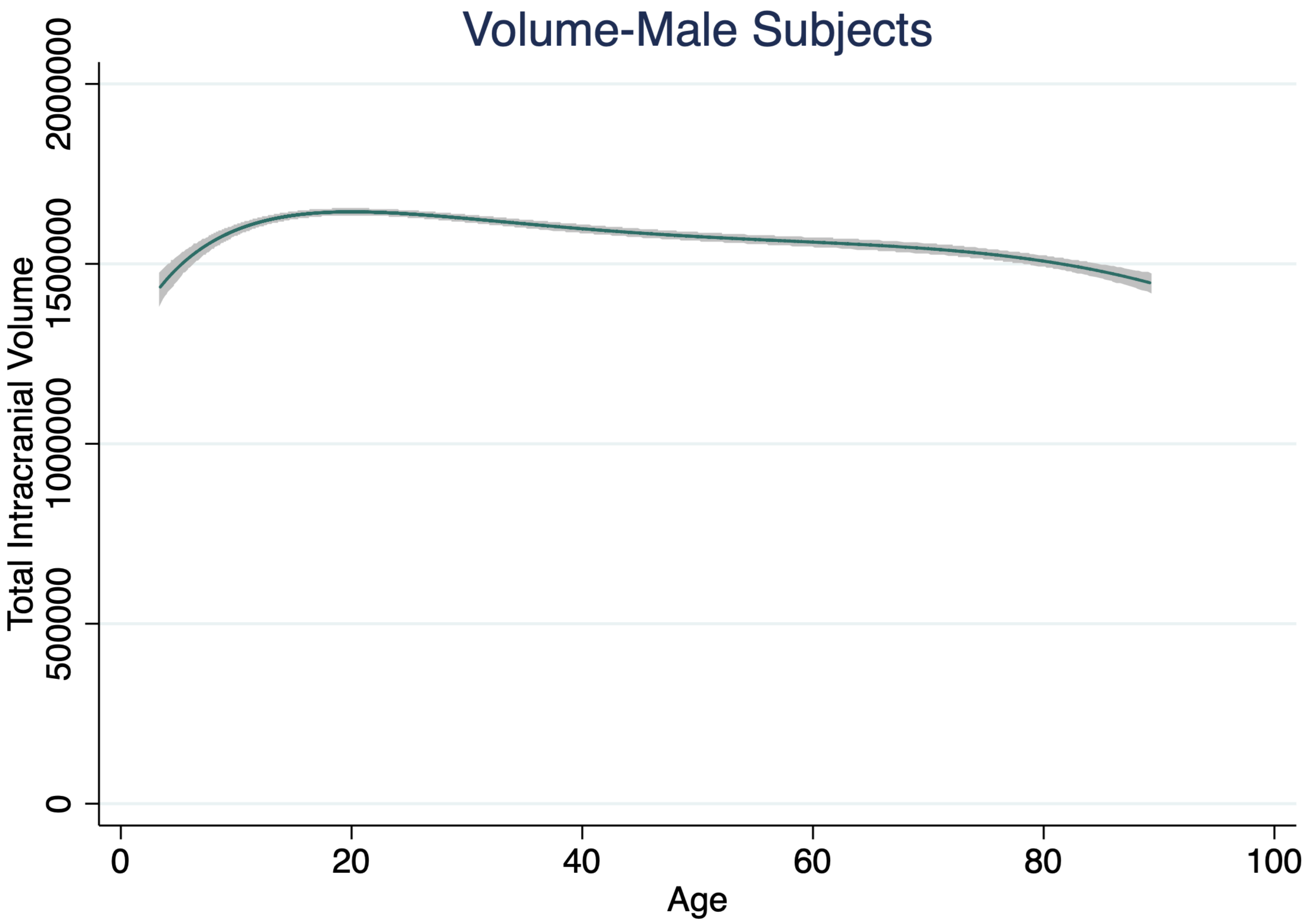
Volume-Male Subjects
Total Intracranial Volume
0
500000
1000000
1500000
2000000
0
20
40
60
80
100
Age

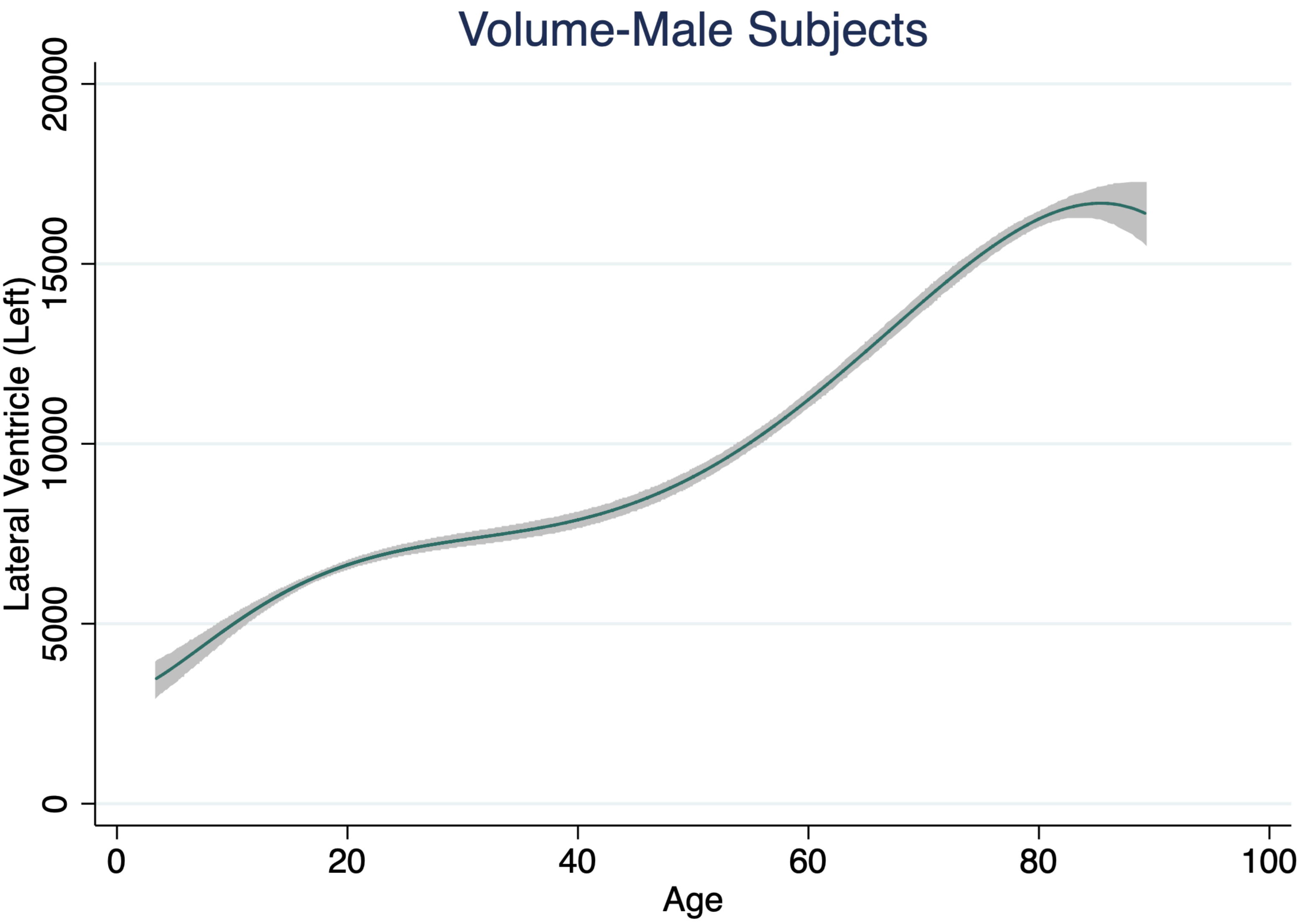
Volume-Male Subjects
Lateral Ventricle (Left)
0
5000
10000
15000
20000
0
20
40
60
80
100
Age

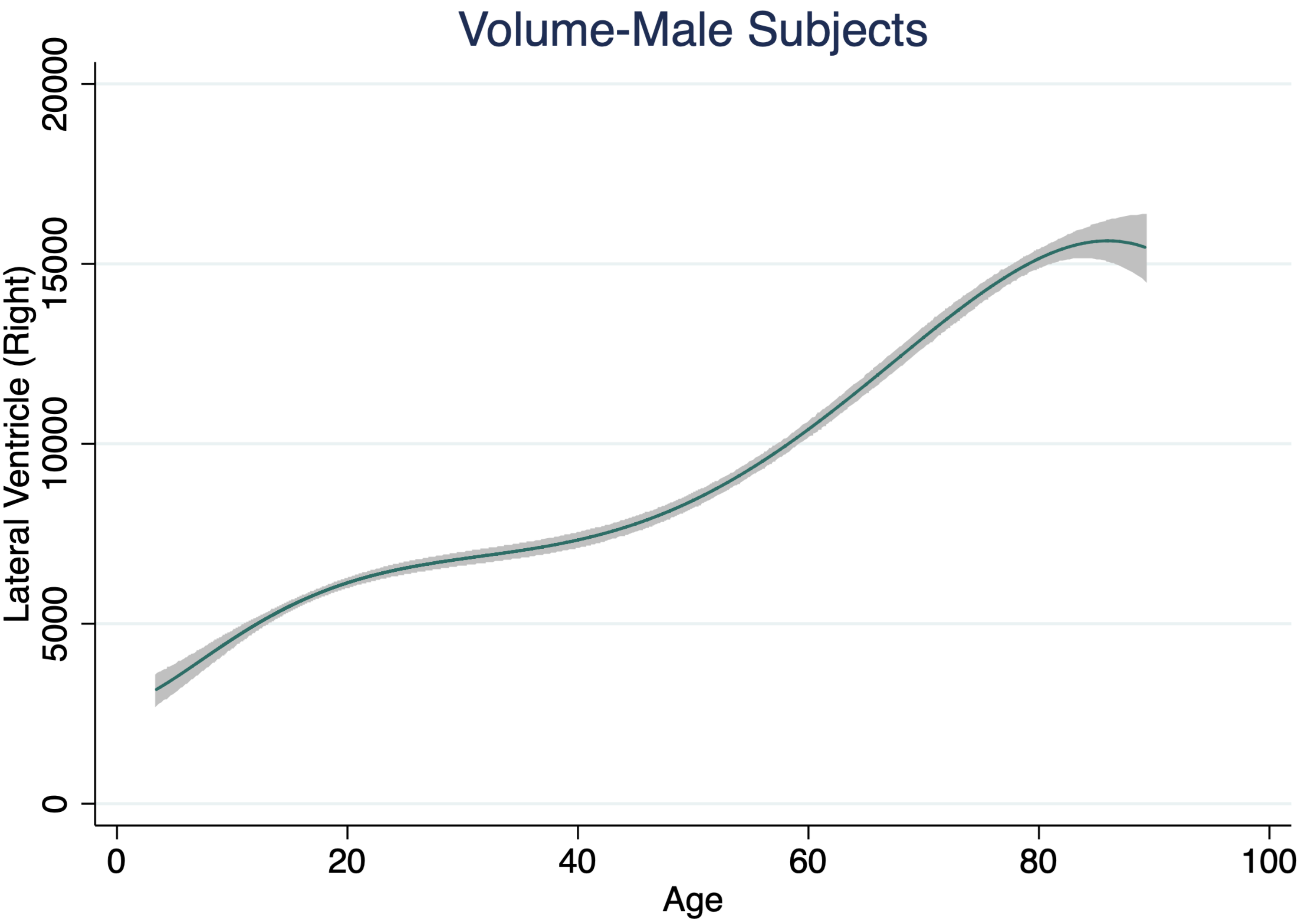

Volume-Male Subjects
Lateral Ventricle (Right)
0
5000
10000
15000
20000
0
20
40
60
80
100
Age

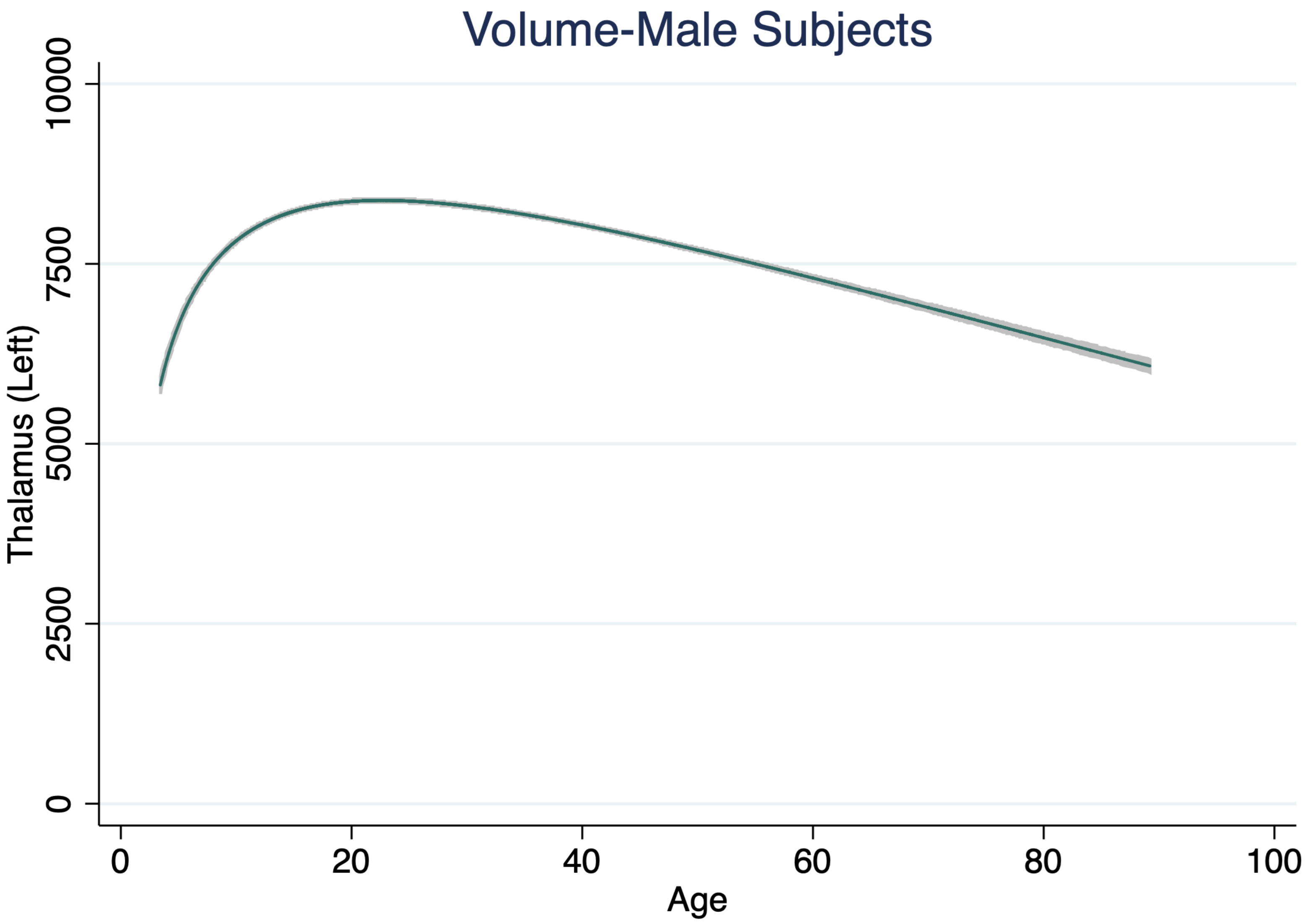

Volume-Male Subjects
Thalamus (Left)
10000
7500
5000
2500
0
0
20
40
60
80
100
Age

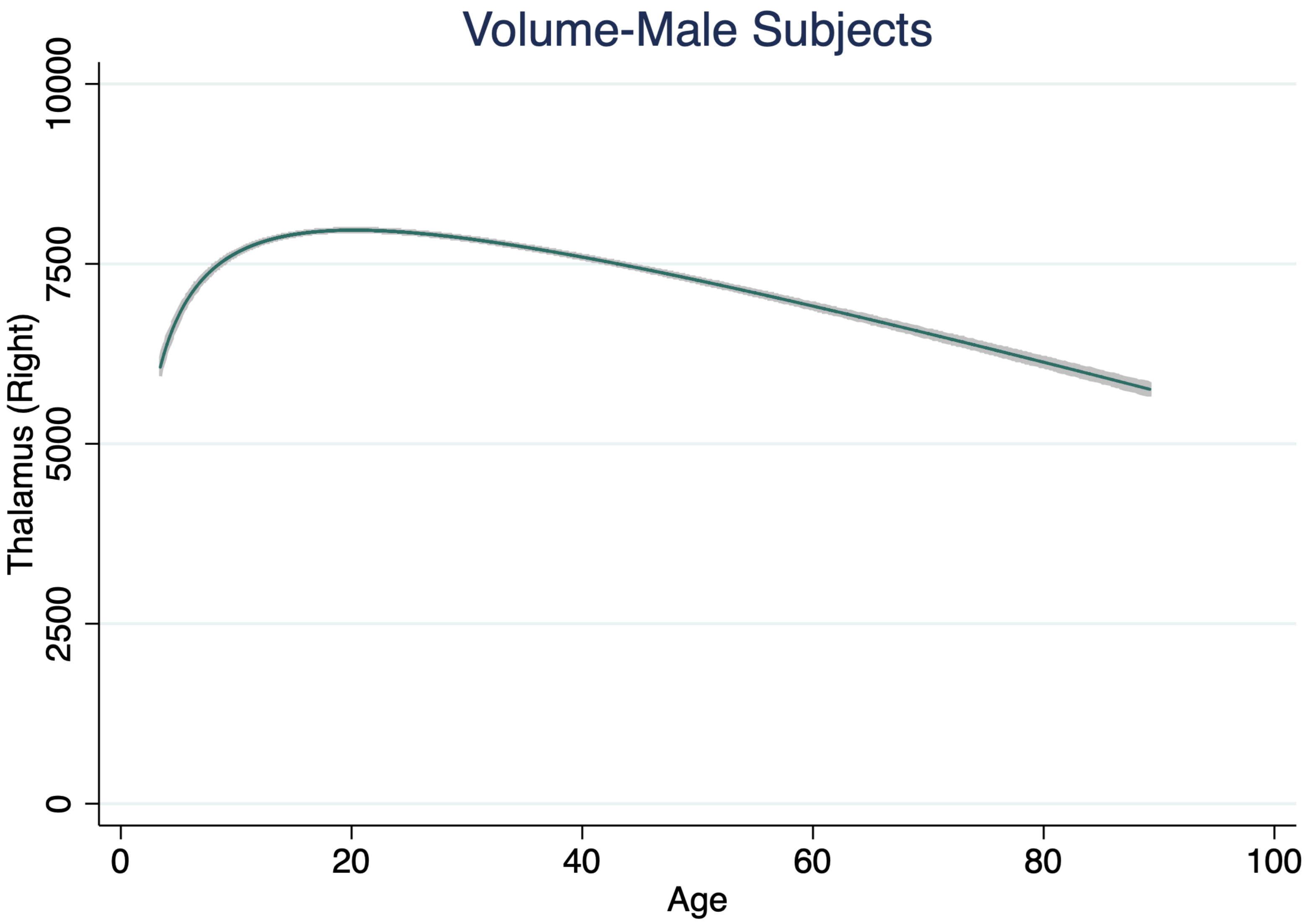

Volume-Male Subjects
Thalamus (Right)
10000
7500
5000
2500
0
0
20
40
60
80
100
Age

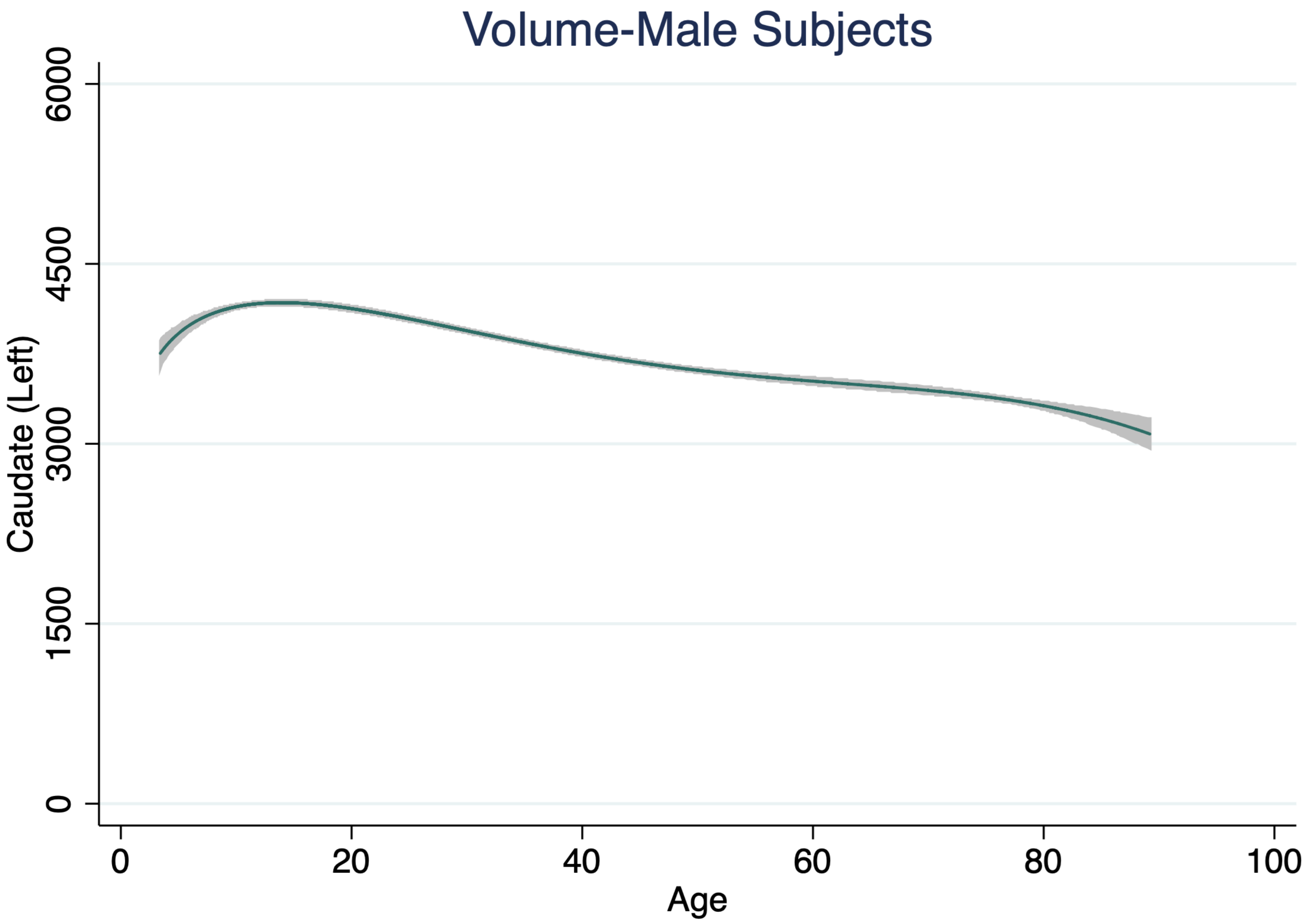
Volume-Male Subjects
6000
4500
3000
1500
0
Caudate (Left)
0
20
40
60
80
100
Age

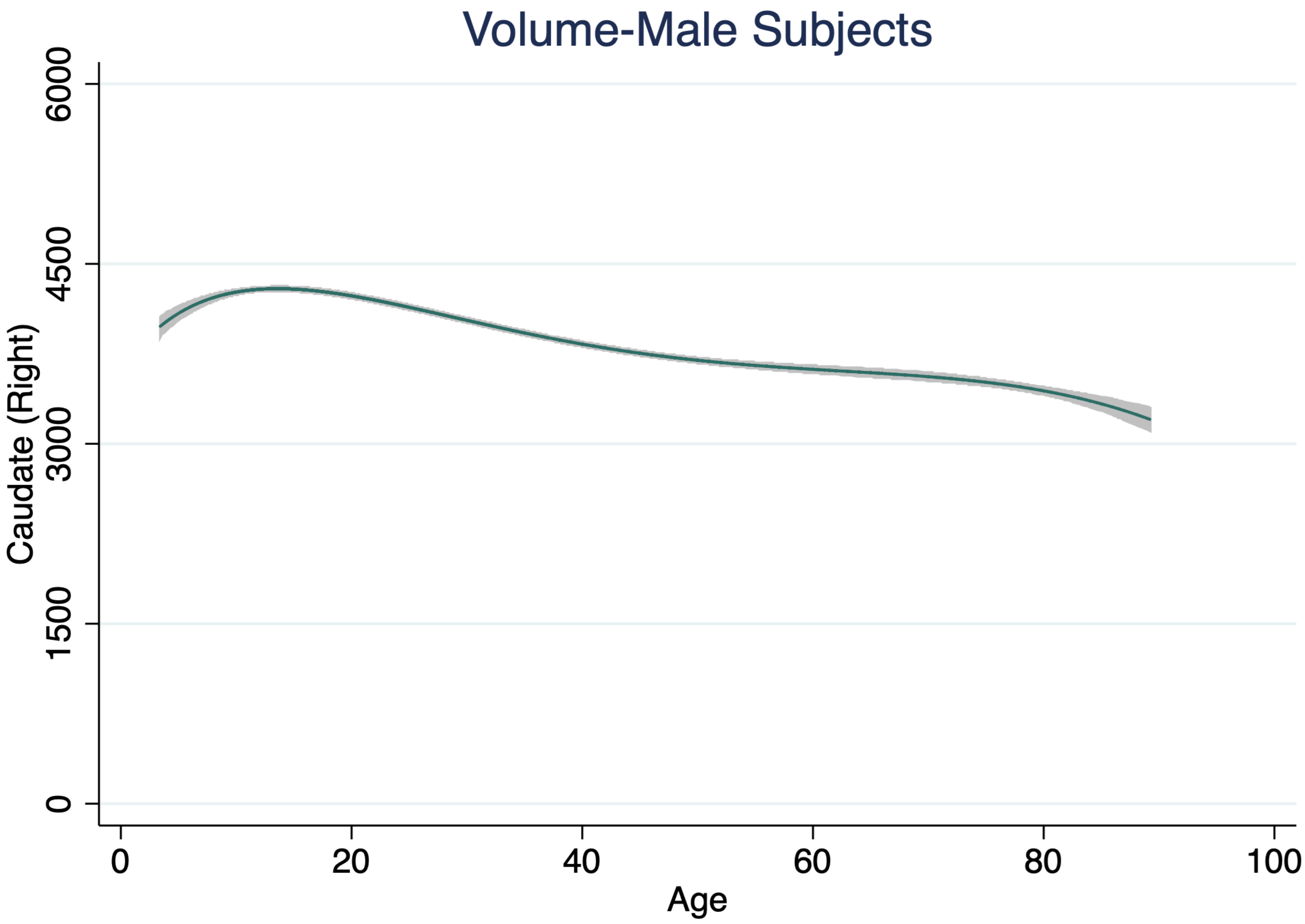
Volume-Male Subjects
Caudate (Right)
6000
4500
3000
1500
0
0
20
40
60
80
100
Age

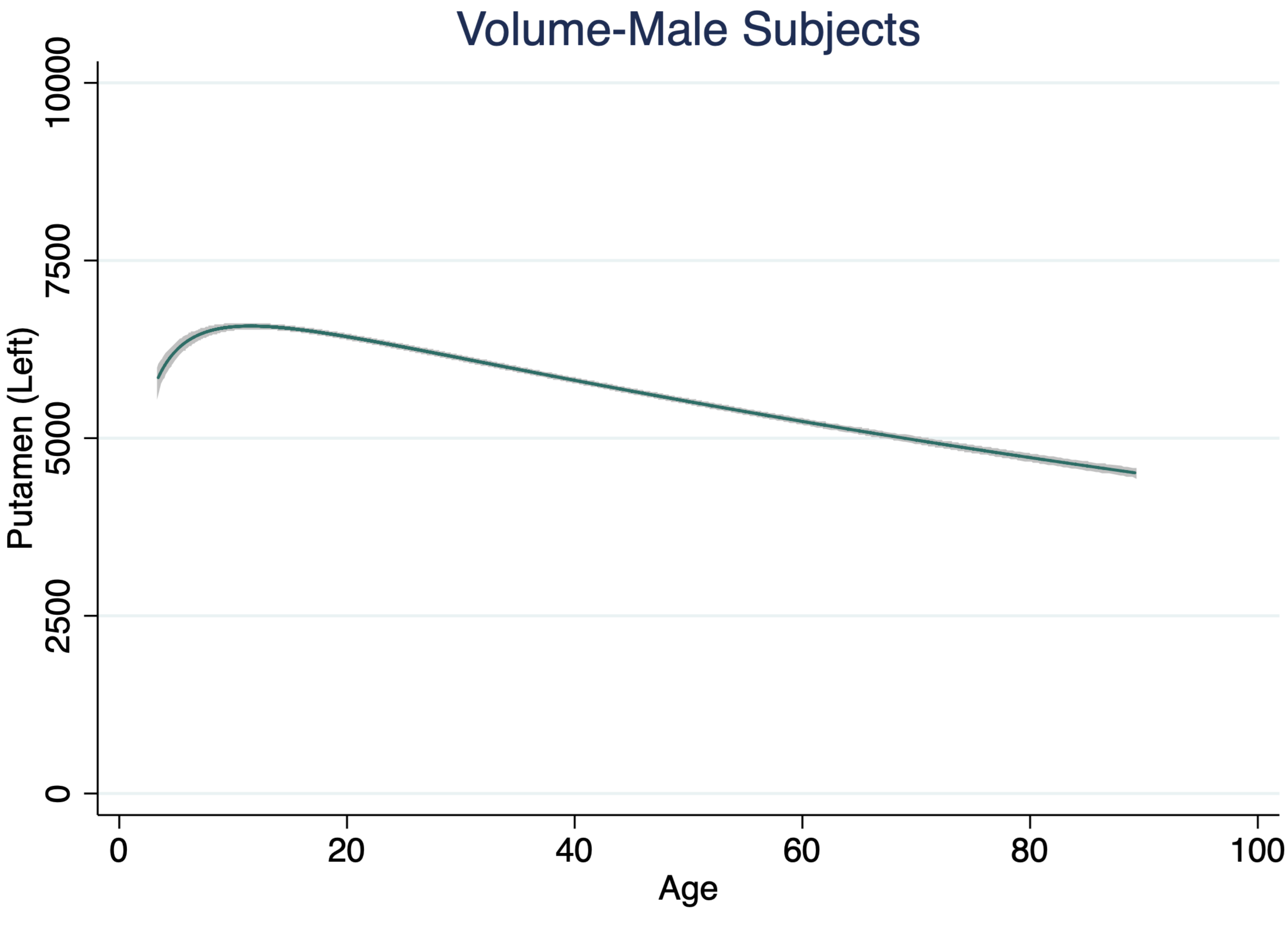
Volume-Male Subjects
Putamen (Left)
10000
7500
5000
2500
0
0
20
40
60
80
100
Age

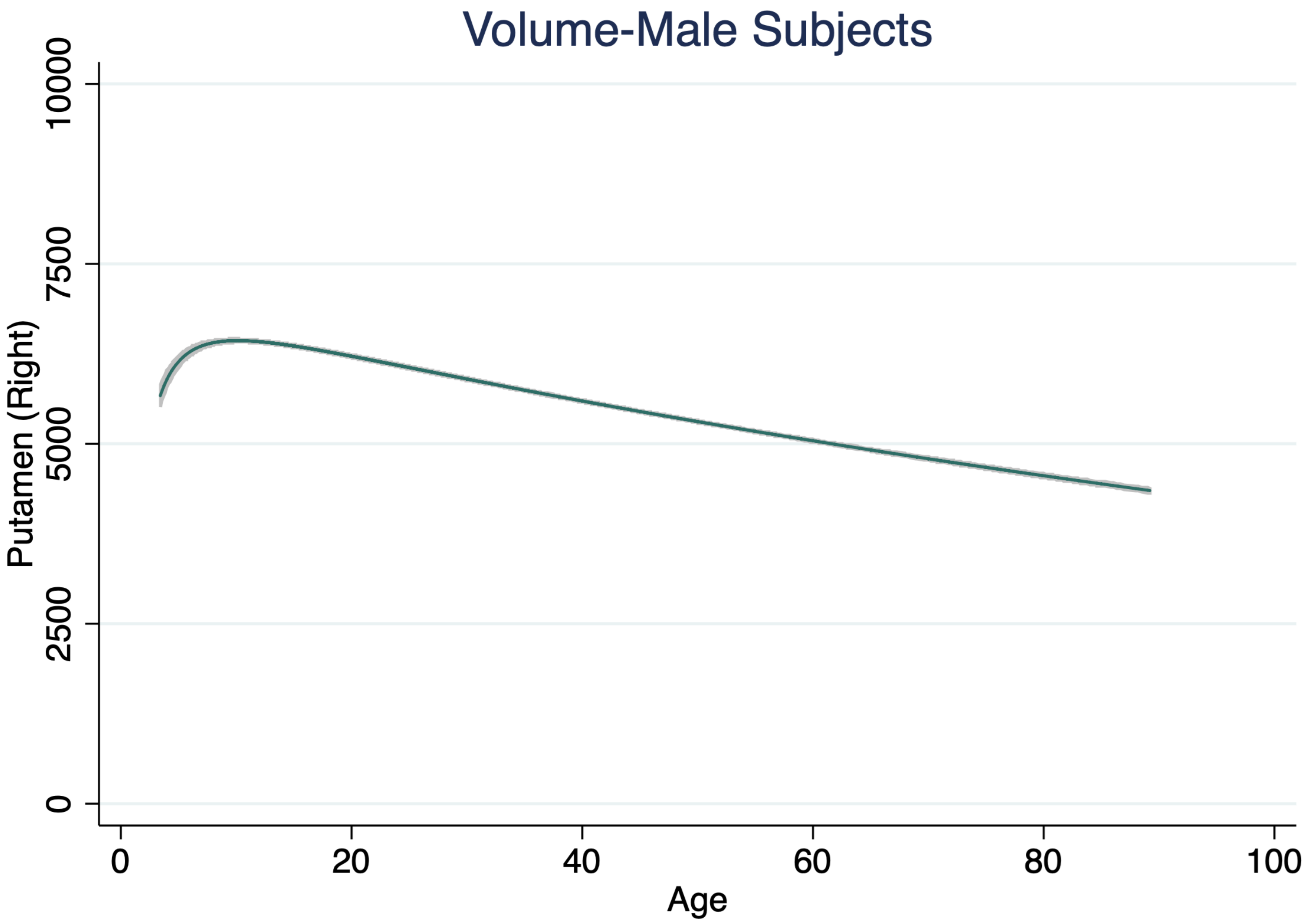

Volume-Male Subjects
Putamen (Right)
10000
7500
5000
2500
0
0
20
40
60
80
100
Age

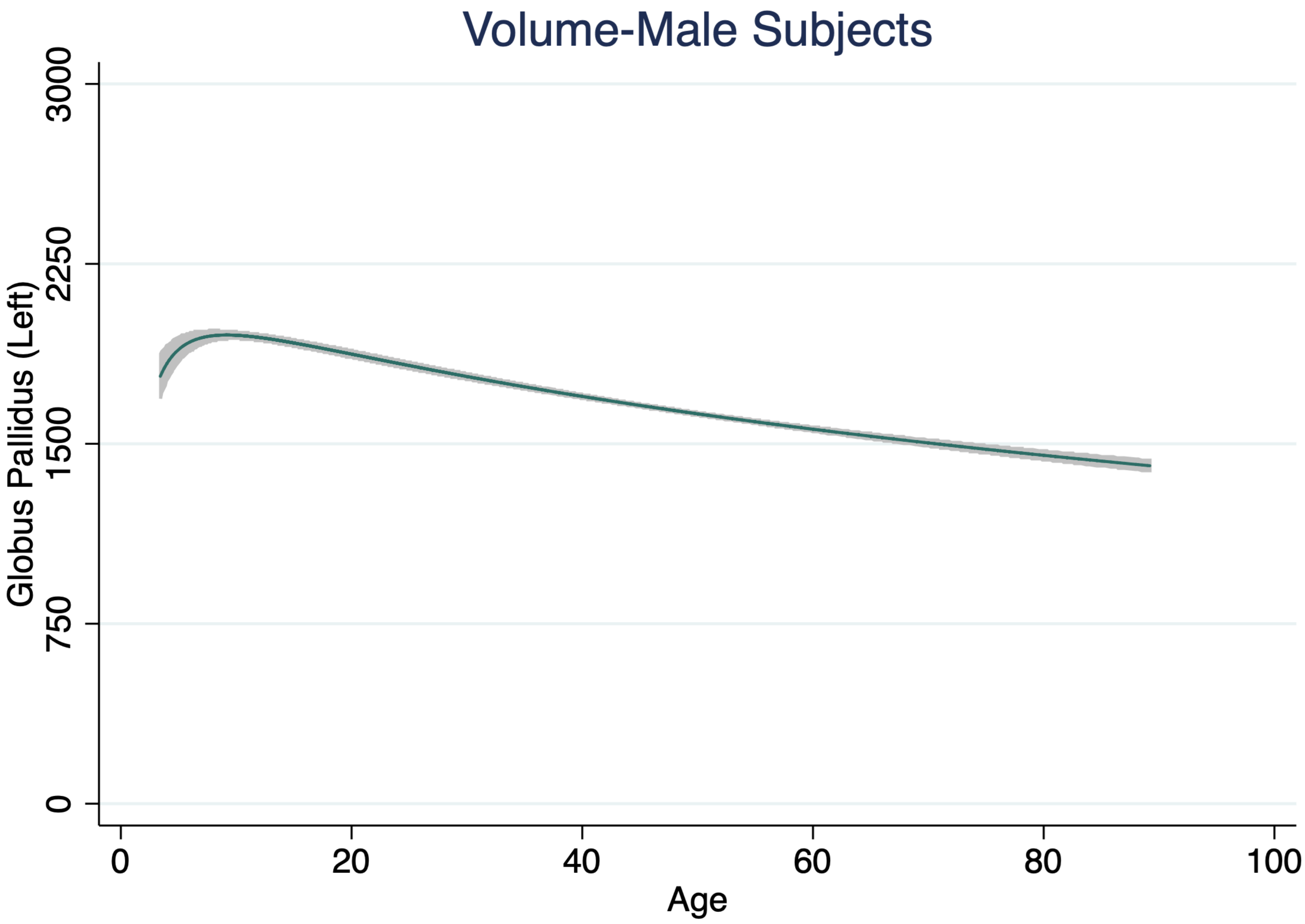

Volume-Male Subjects
Globus Pallidus (Left)
3000
2250
1500
750
0
0
20
40
60
80
100
Age

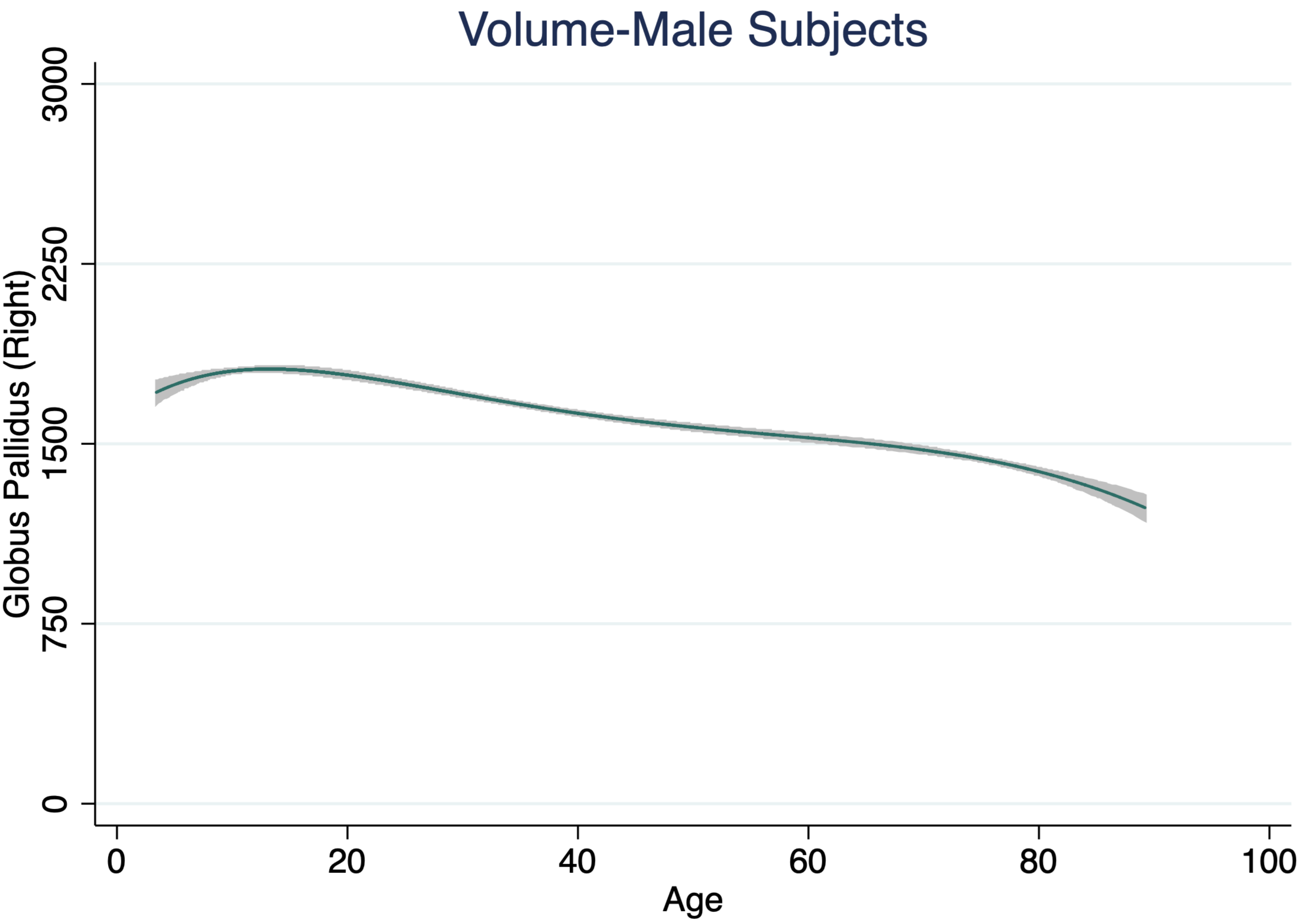
Volume-Male Subjects
Globus Pallidus (Right)
3000
2250
1500
750
0
0
20
40
60
80
100
Age

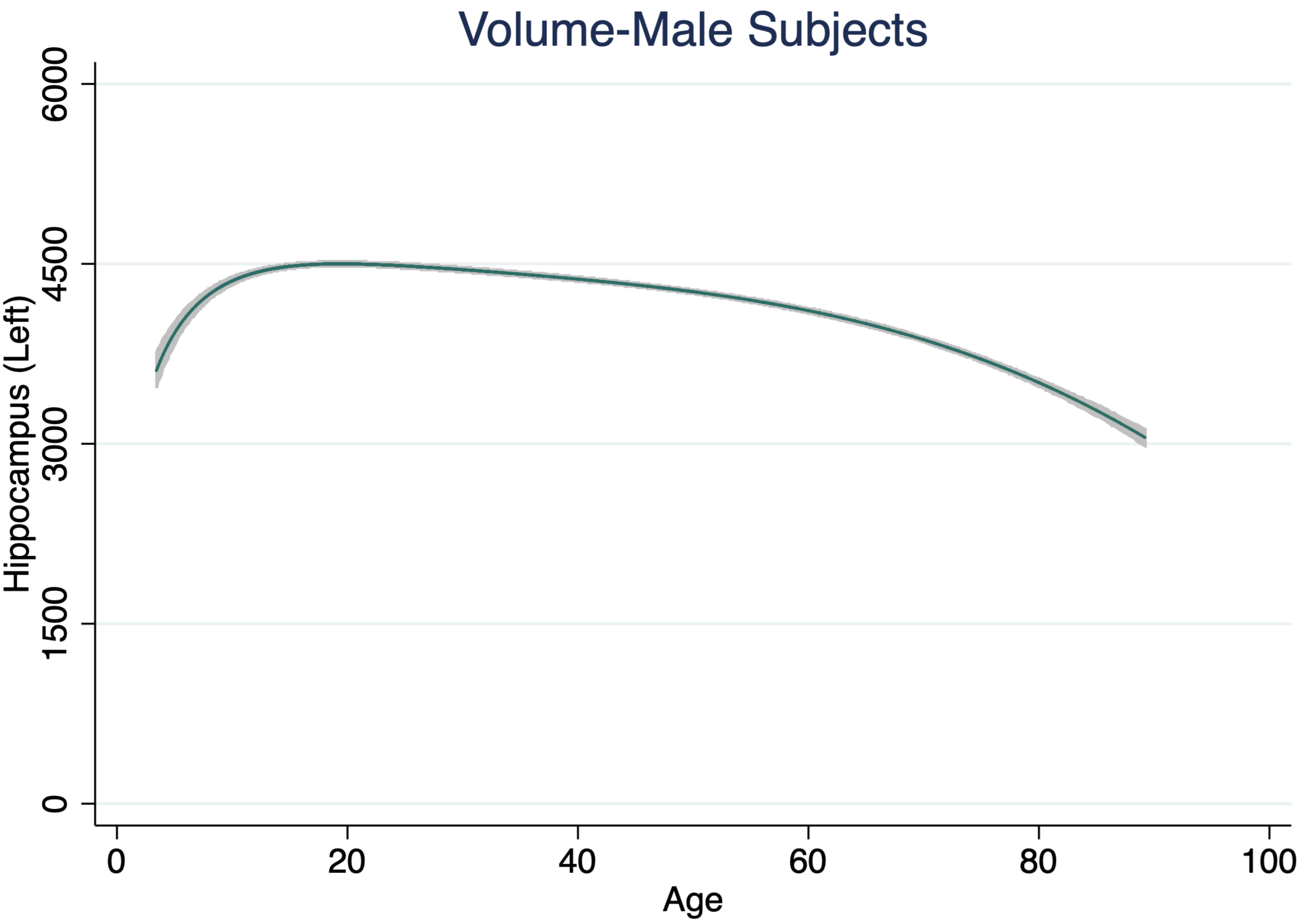
Volume-Male Subjects
Hippocampus (Left)
6000
4500
3000
1500
0
0
20
40
60
80
100
Age

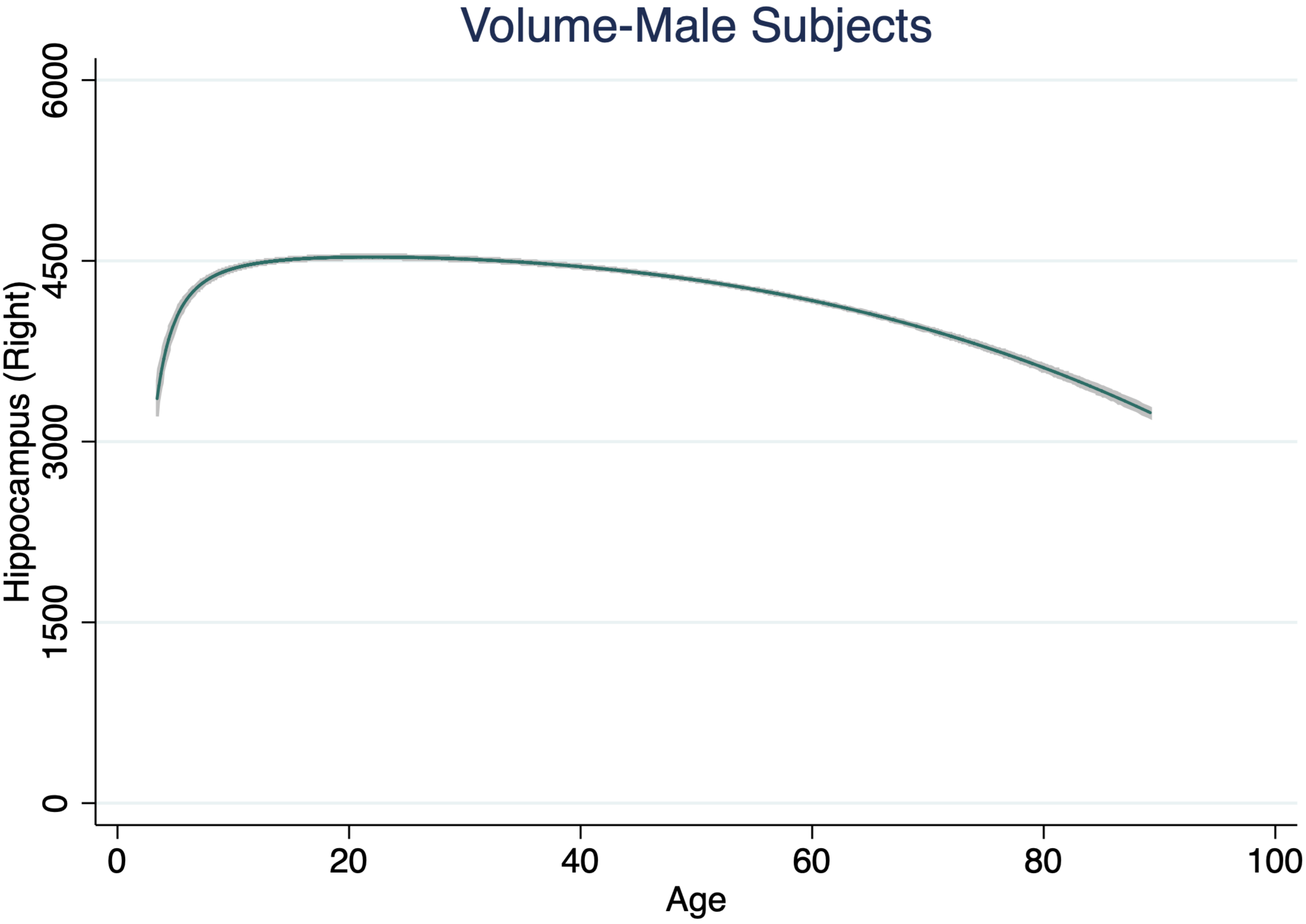

Volume-Male Subjects
Hippocampus (Right)
6000
4500
3000
1500
0
0
20
40
60
80
100
Age

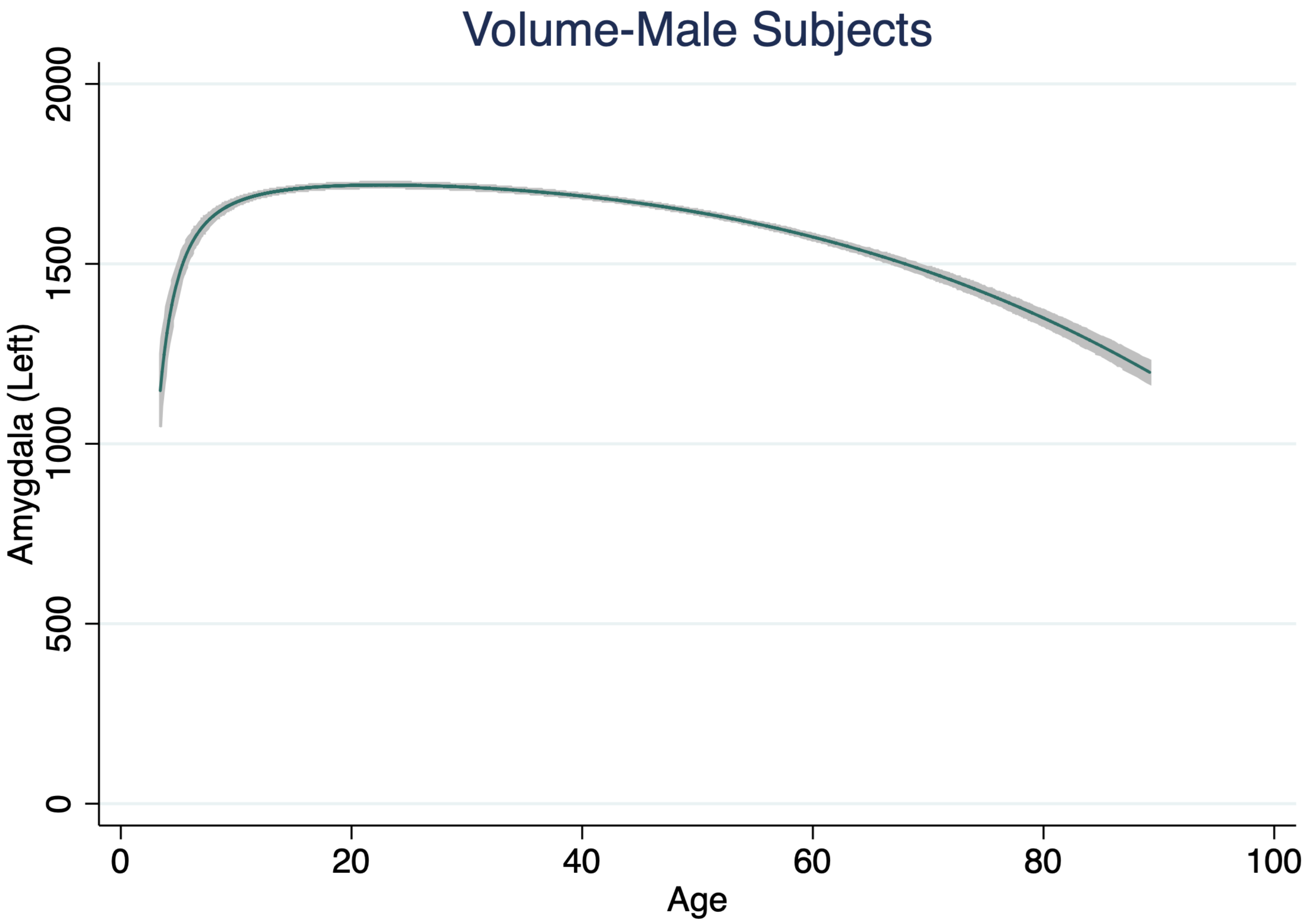
Volume-Male Subjects
Amygdala (Left)
0
500
1000
1500
2000
0
20
40
60
80
100
Age

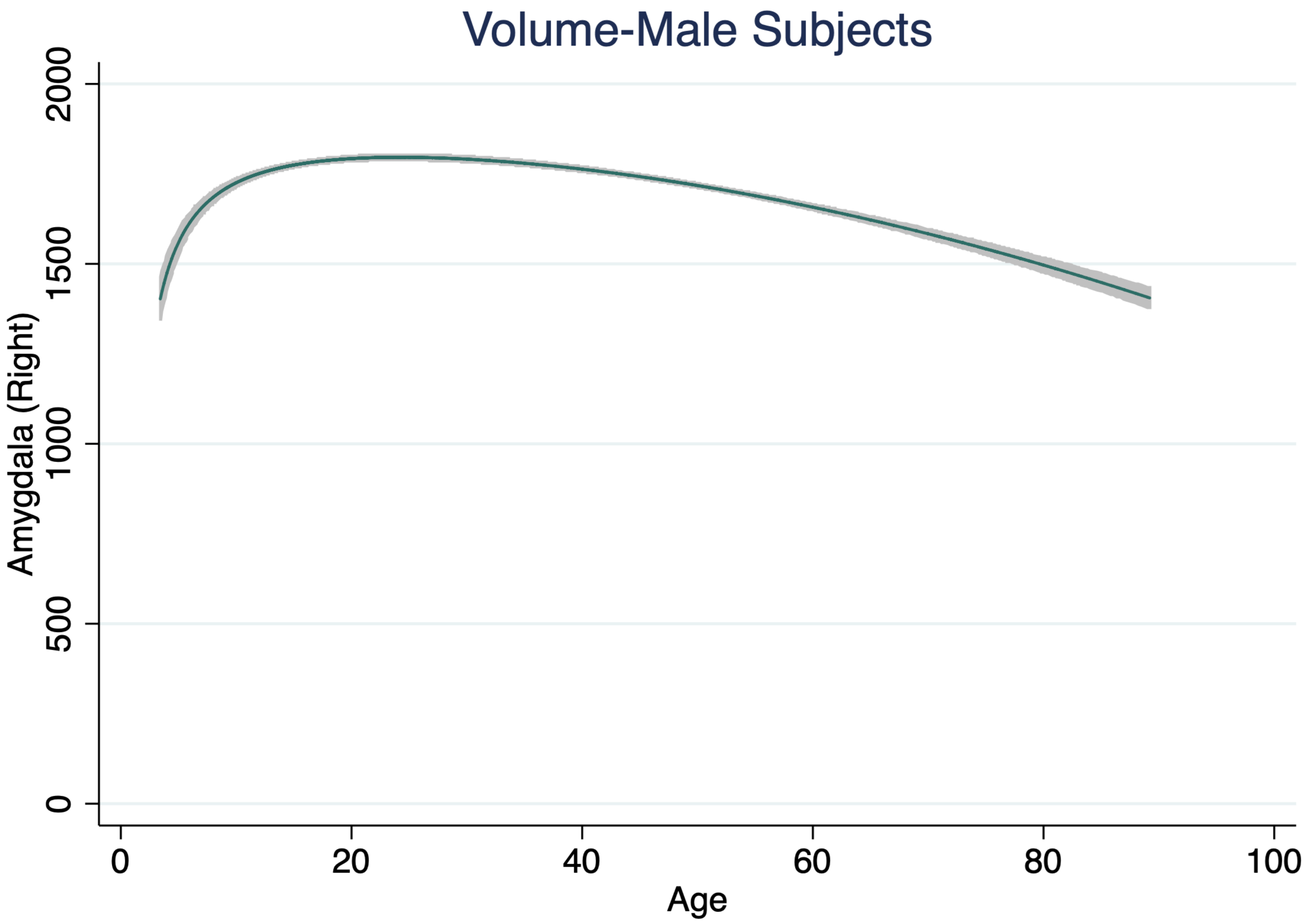
Volume-Male Subjects
Amygdala (Right)
2000
1500
1000
500
0
0
20
40
60
80
100
Age

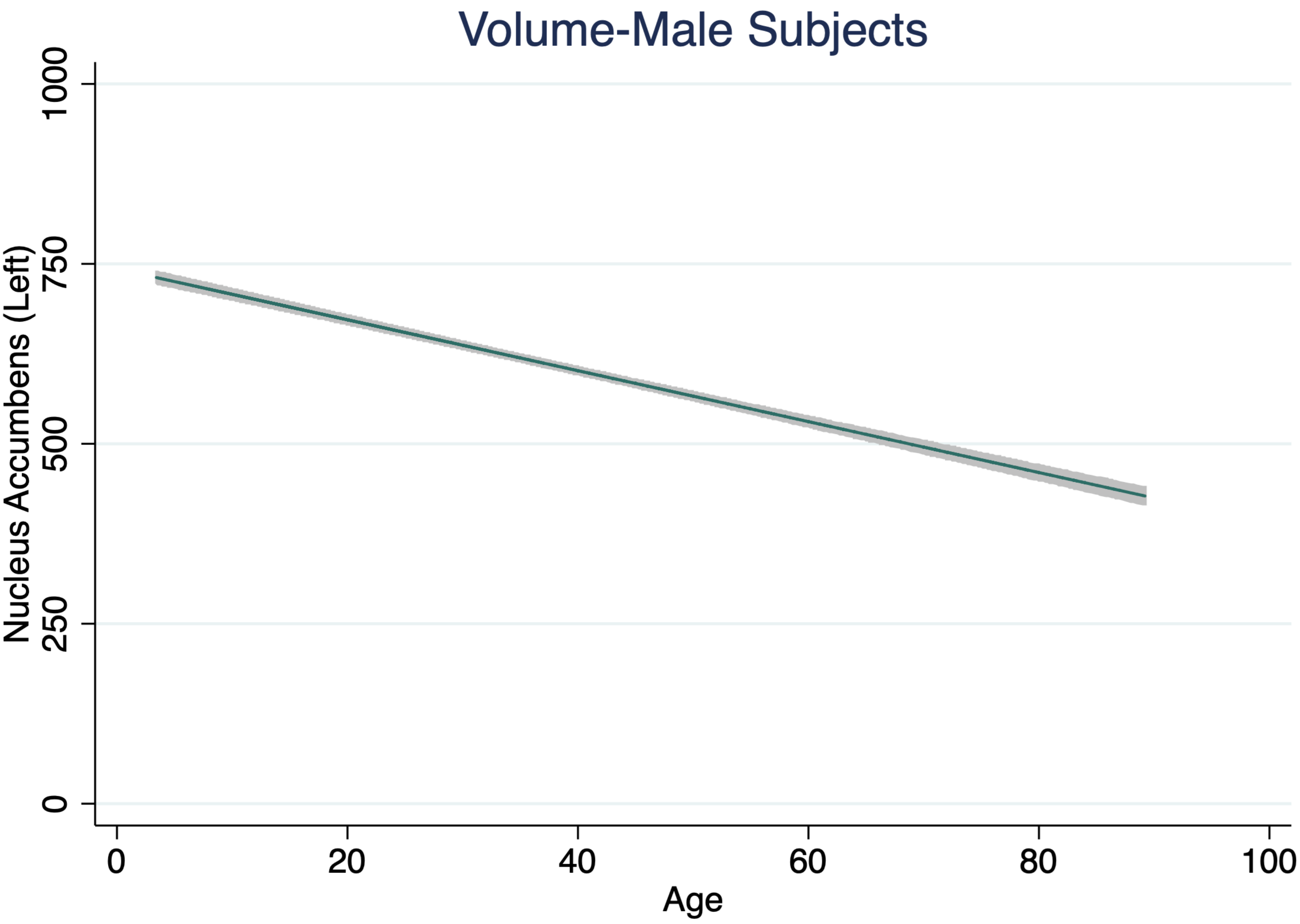
Volume-Male Subjects
Nucleus Accumbens (Left)
1000
750
500
250
0
0
20
40
60
80
100
Age

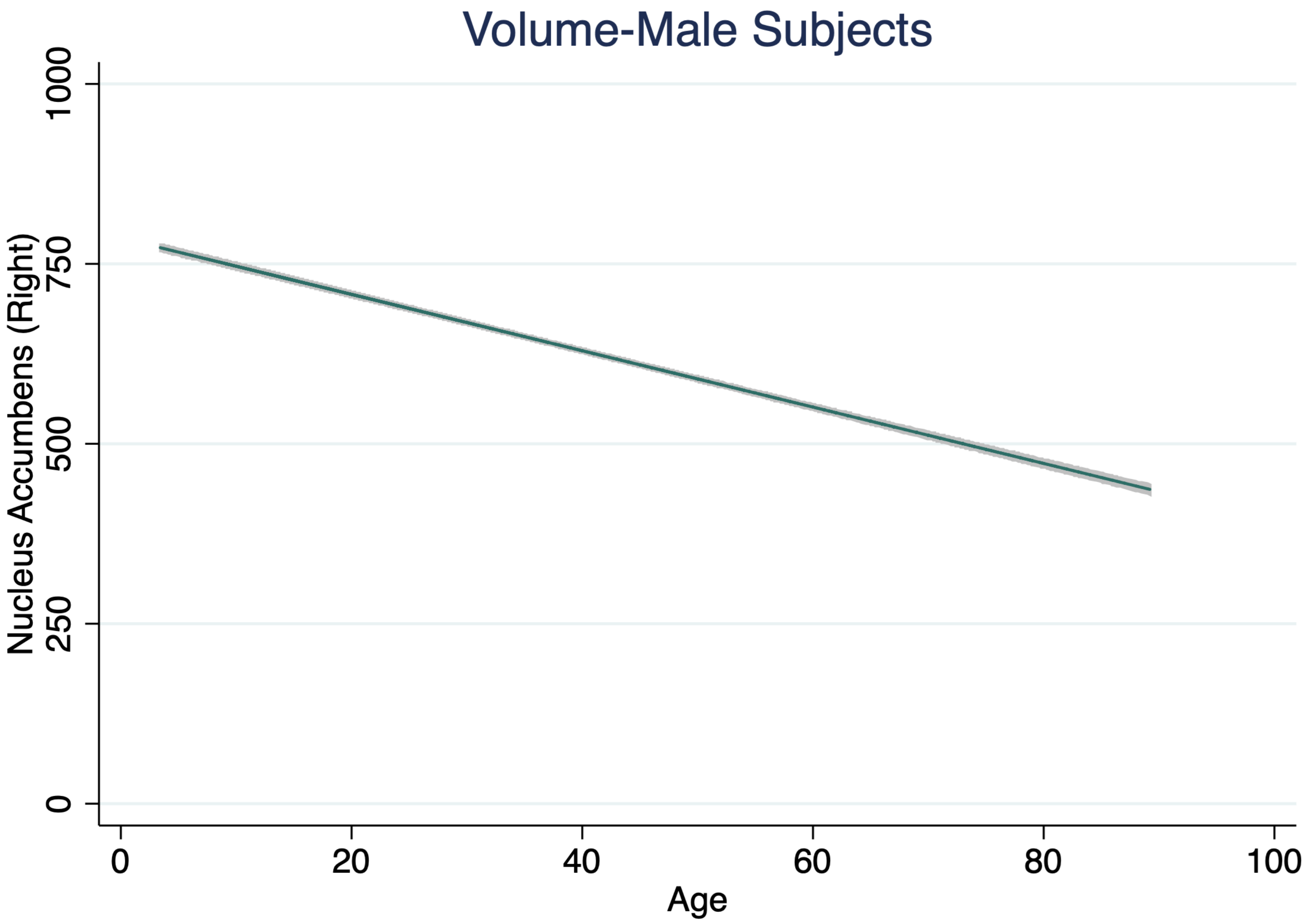
Volume-Male Subjects
Nucleus Accumbens (Right)
1000
750
500
250
0
0
20
40
60
80
100
Age

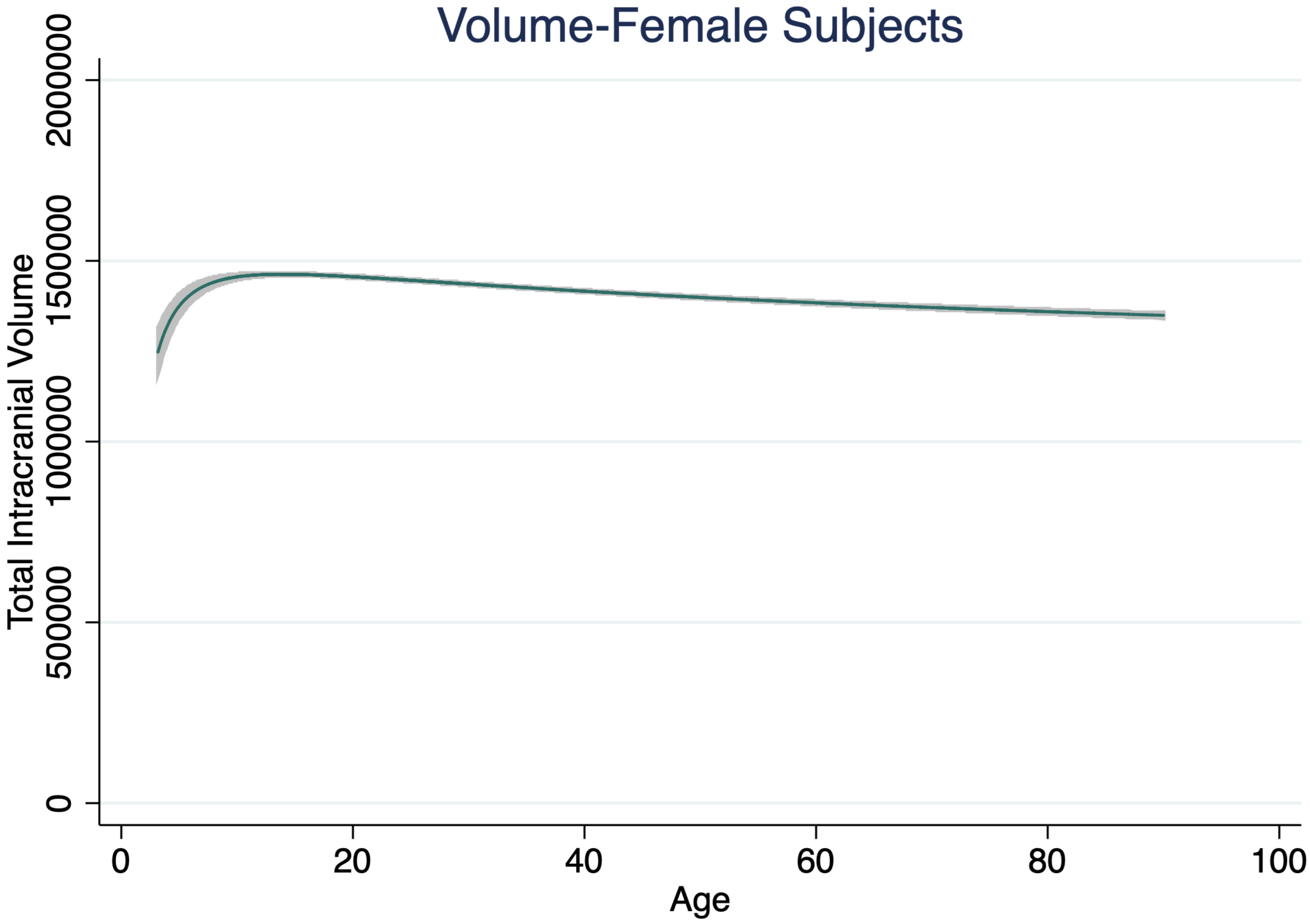
Volume-Female Subjects
Total Intracranial Volume
0
500000
1000000
1500000
2000000
0
20
40
60
80
100
Age

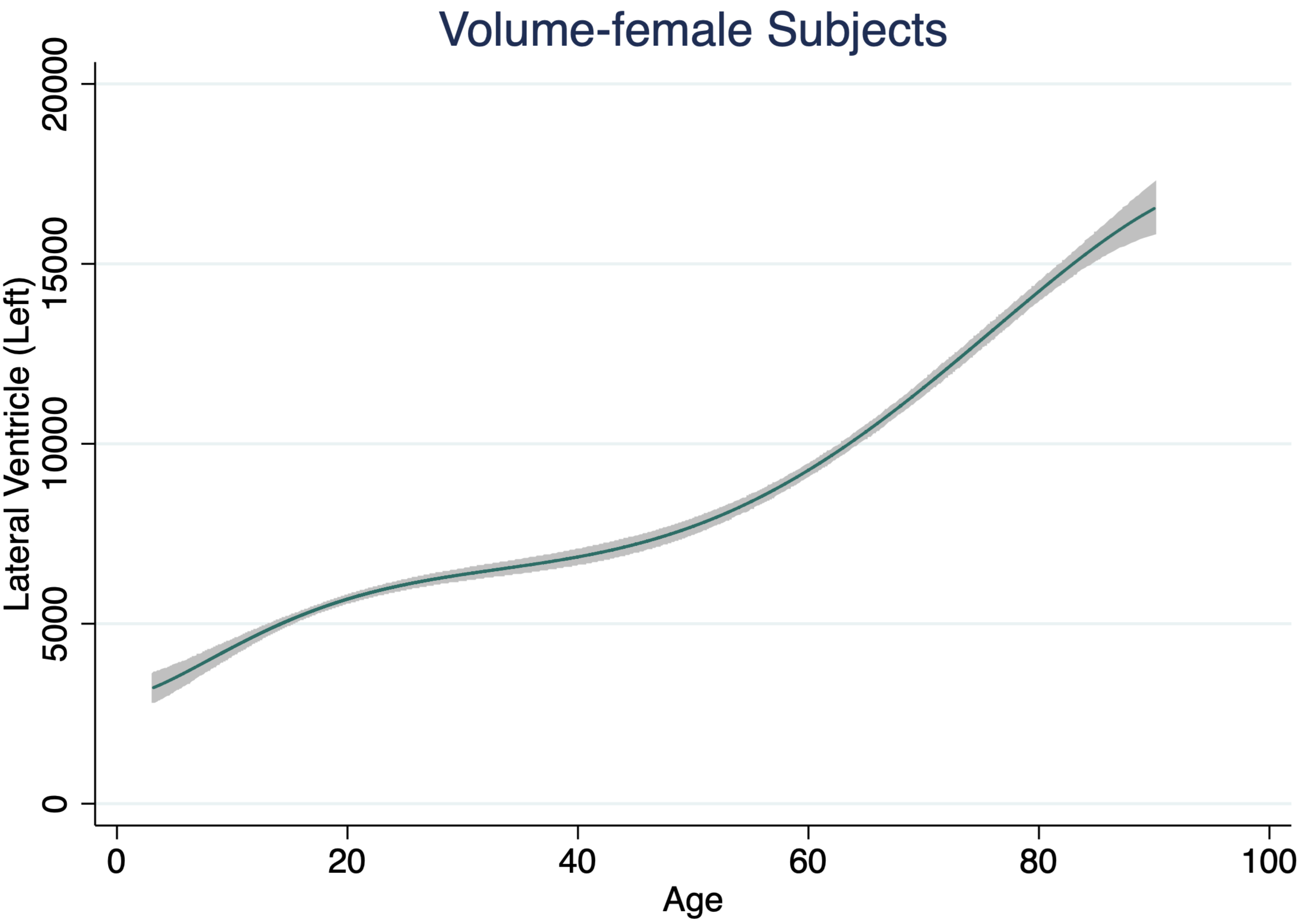

Volume-female Subjects
Lateral Ventricle (Left)
0
5000
10000
15000
20000
0
20
40
60
80
100
Age

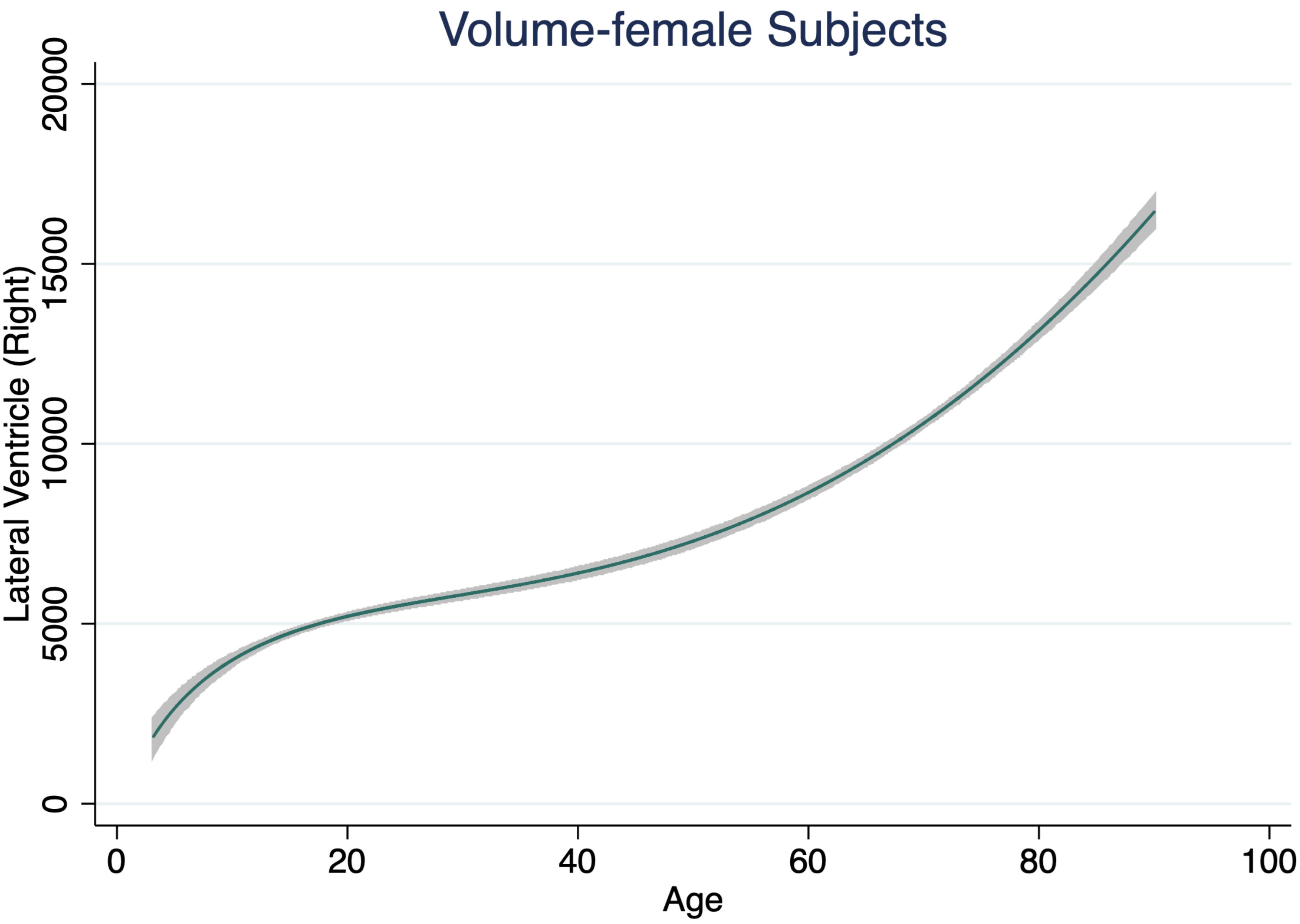
Volume-female Subjects
Lateral Ventricle (Right)
20000
15000
10000
5000
0
0
20
40
60
80
100
Age

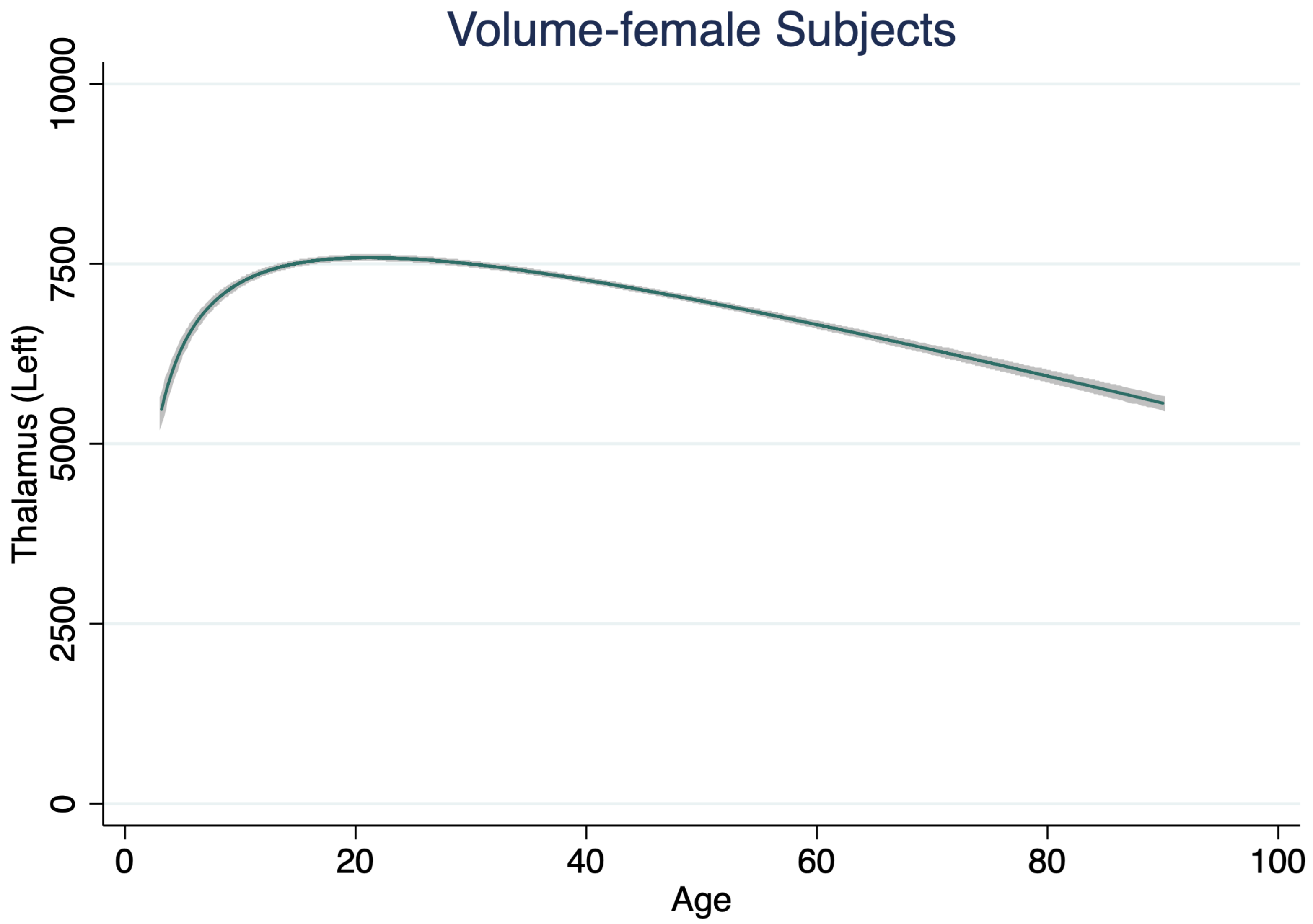

Volume-female Subjects
Thalamus (Left)
10000
7500
5000
2500
0
0
20
40
60
80
100
Age

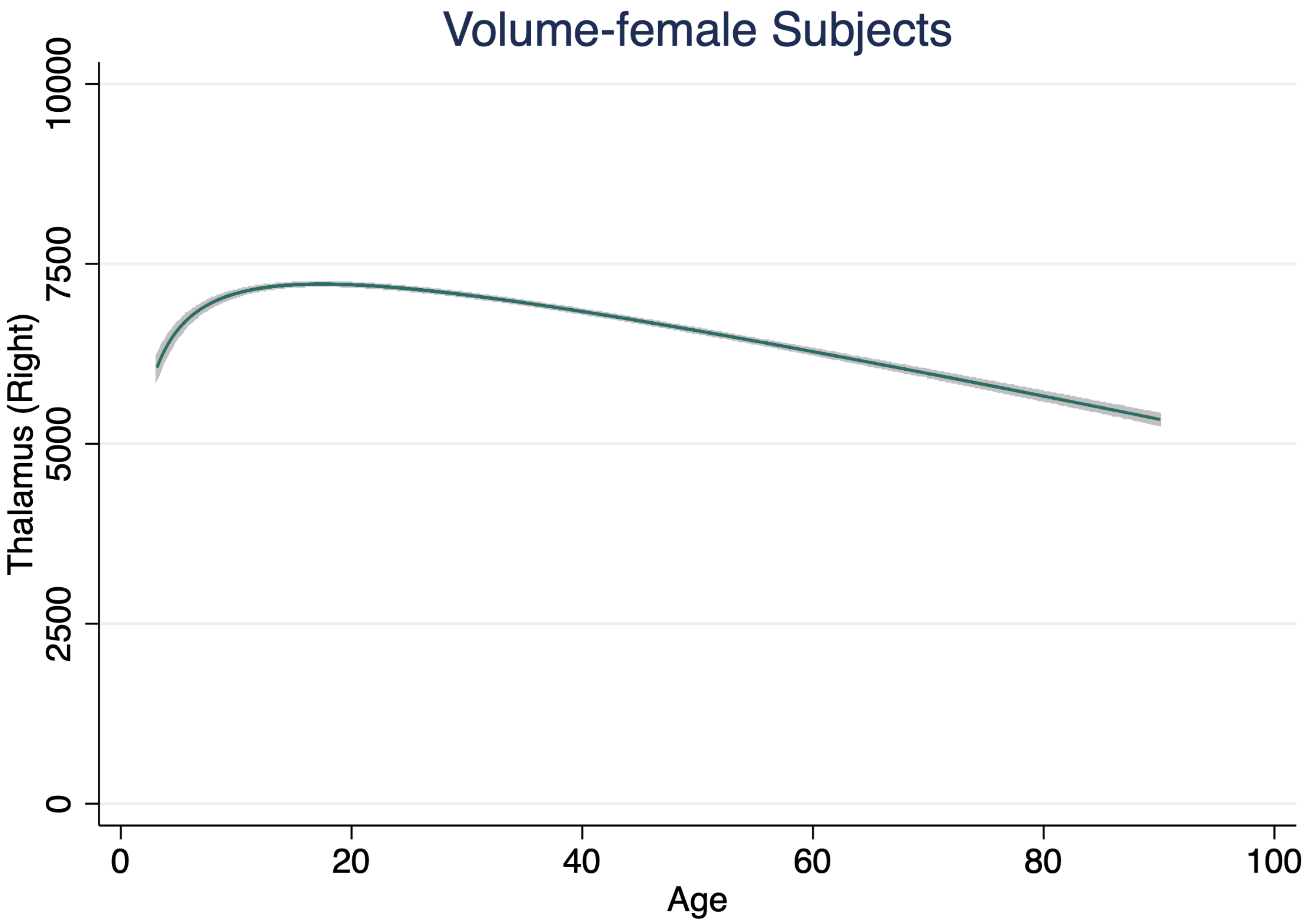
Volume-female Subjects
Thalamus (Right)
0
2500
5000
7500
10000
Age
0
20
40
60
80
100

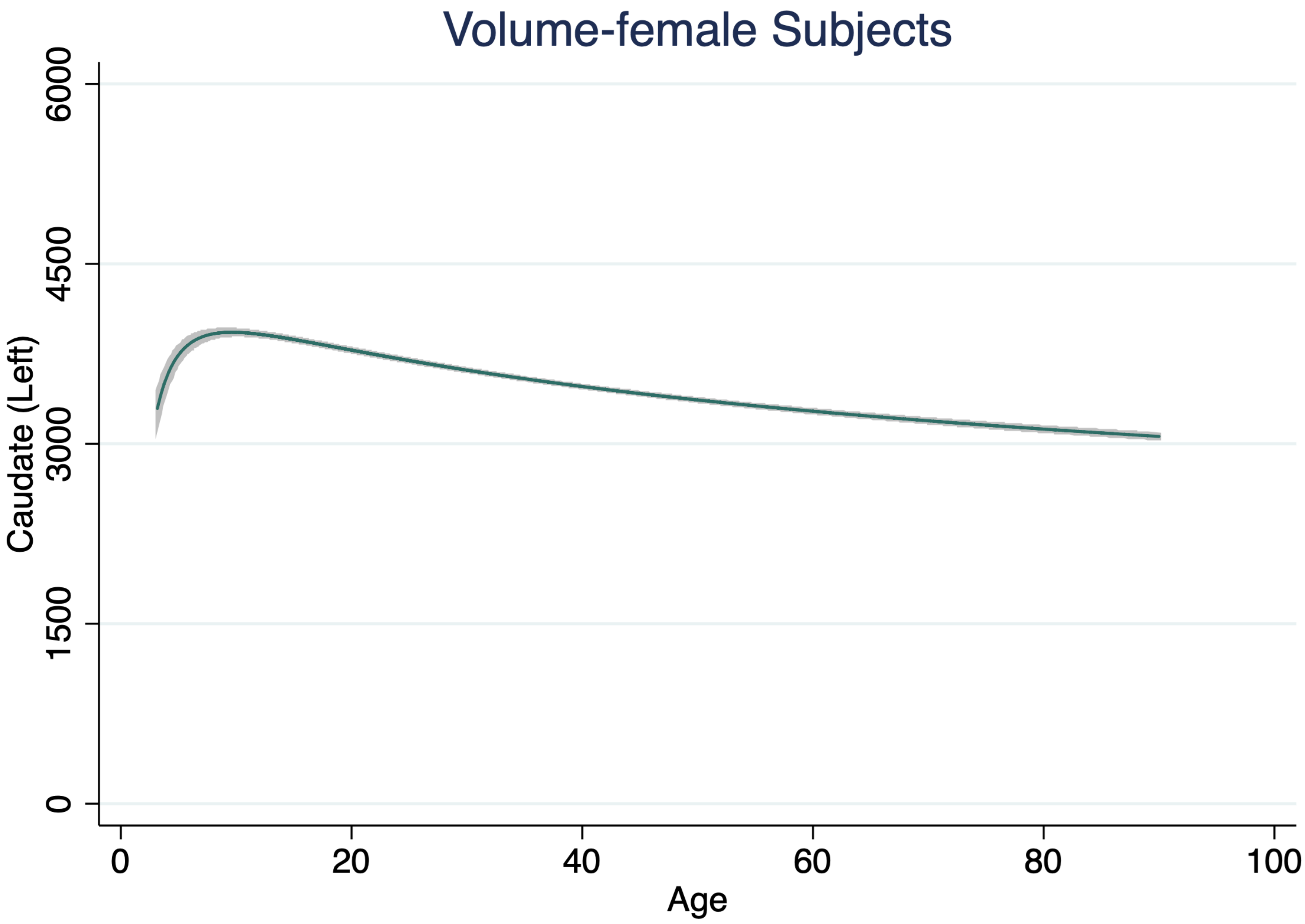

Volume-female Subjects
Caudate (Left)
6000
4500
3000
1500
0
0
20
40
60
80
100
Age

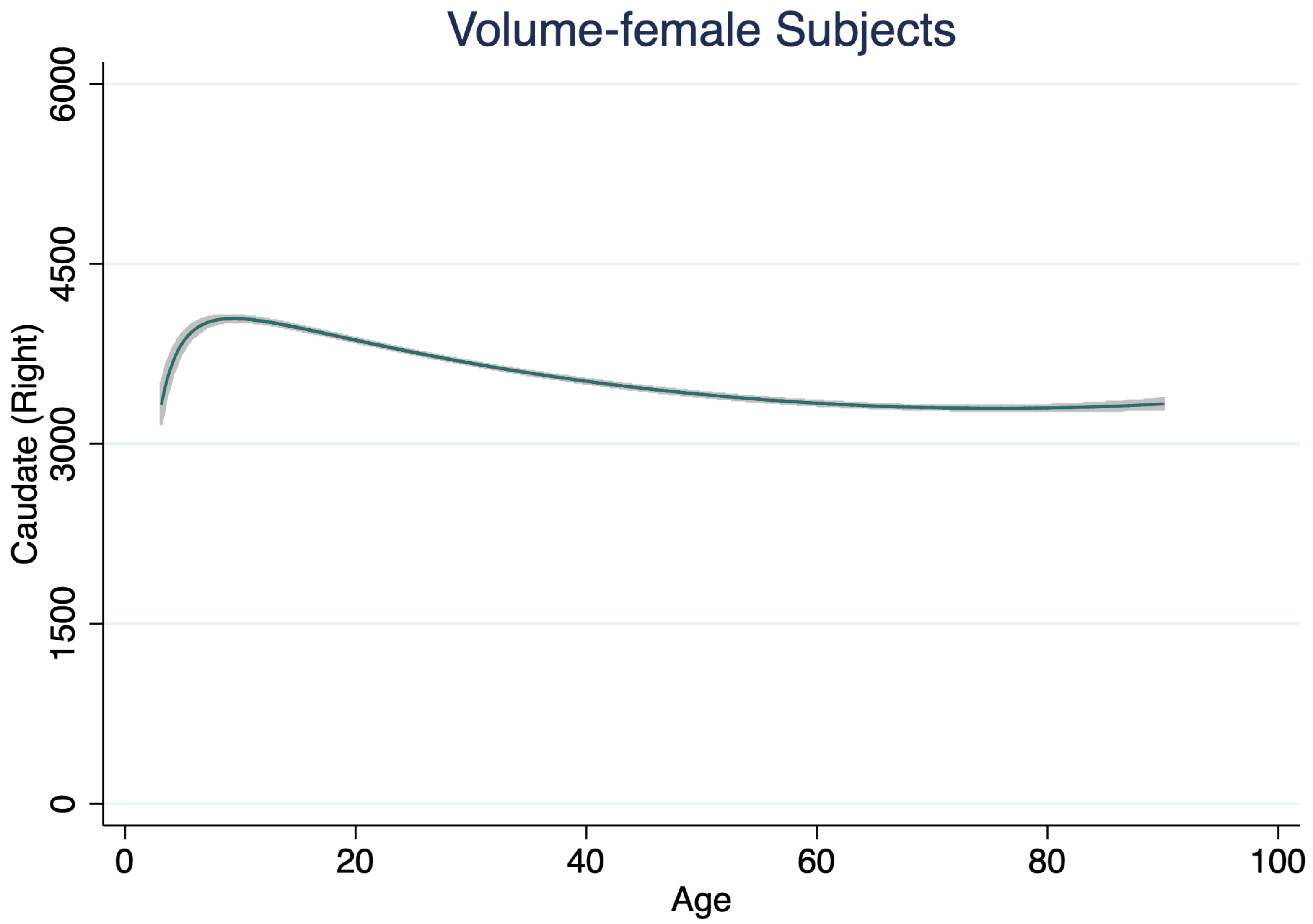
Volume-female Subjects
Caudate (Right)
6000
4500
3000
1500
0
0
20
40
60
80
100
Age

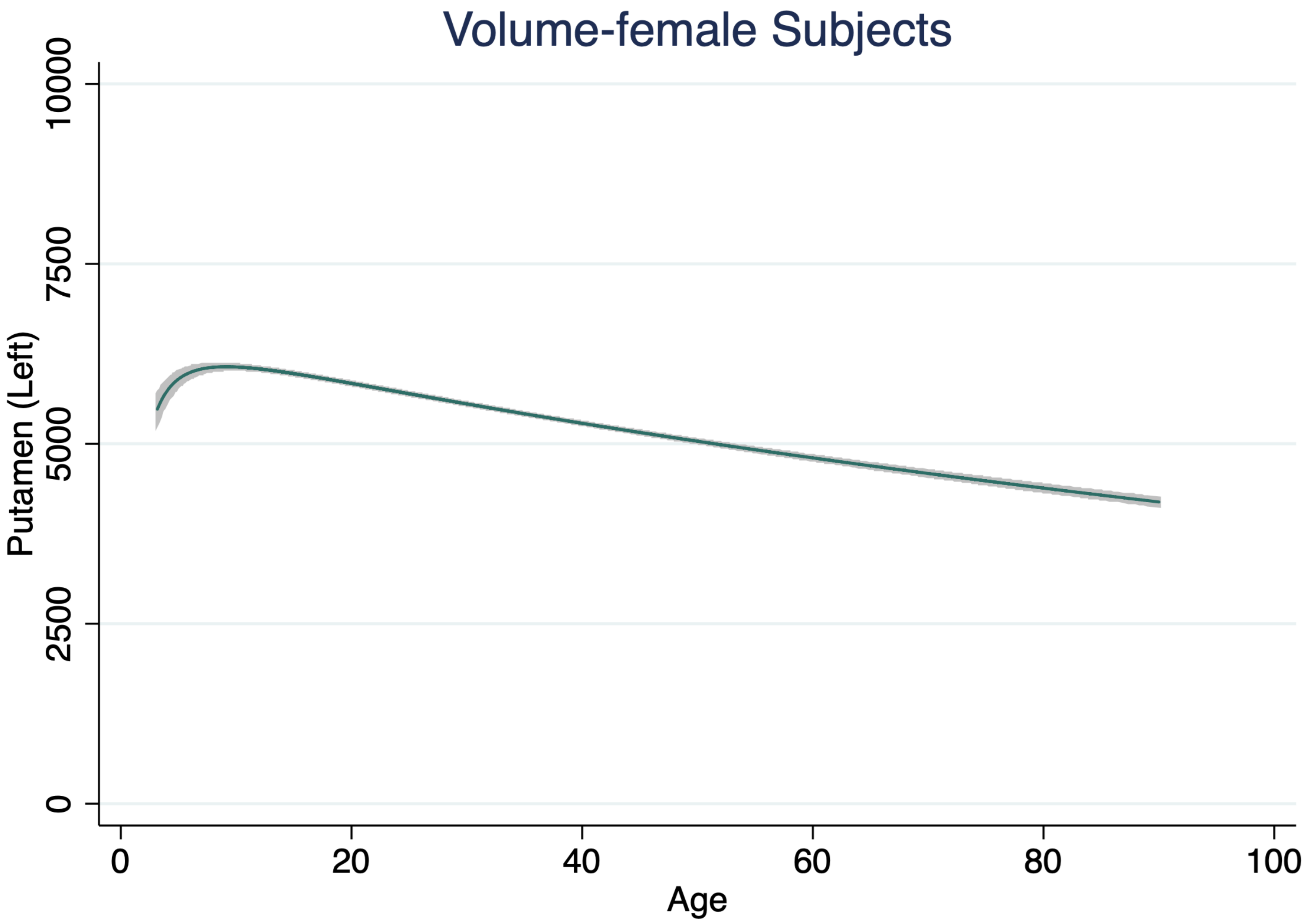
Volume-female Subjects
10000
7500
5000
2500
0
Putamen (Left)
0
20
40
60
80
100
Age

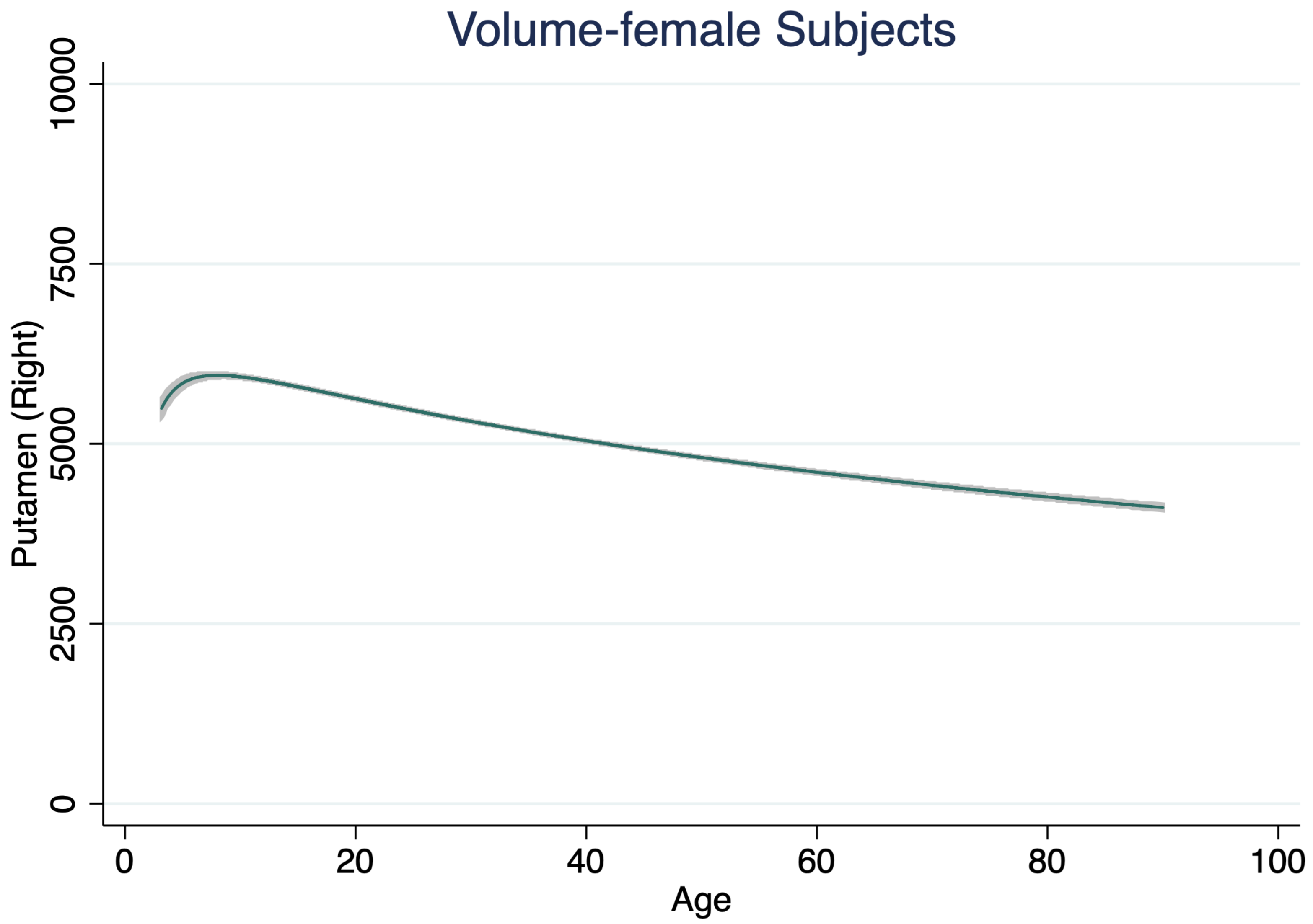

Volume-female Subjects
10000
7500
5000
2500
0
Putamen (Right)
0
20
40
60
80
100
Age

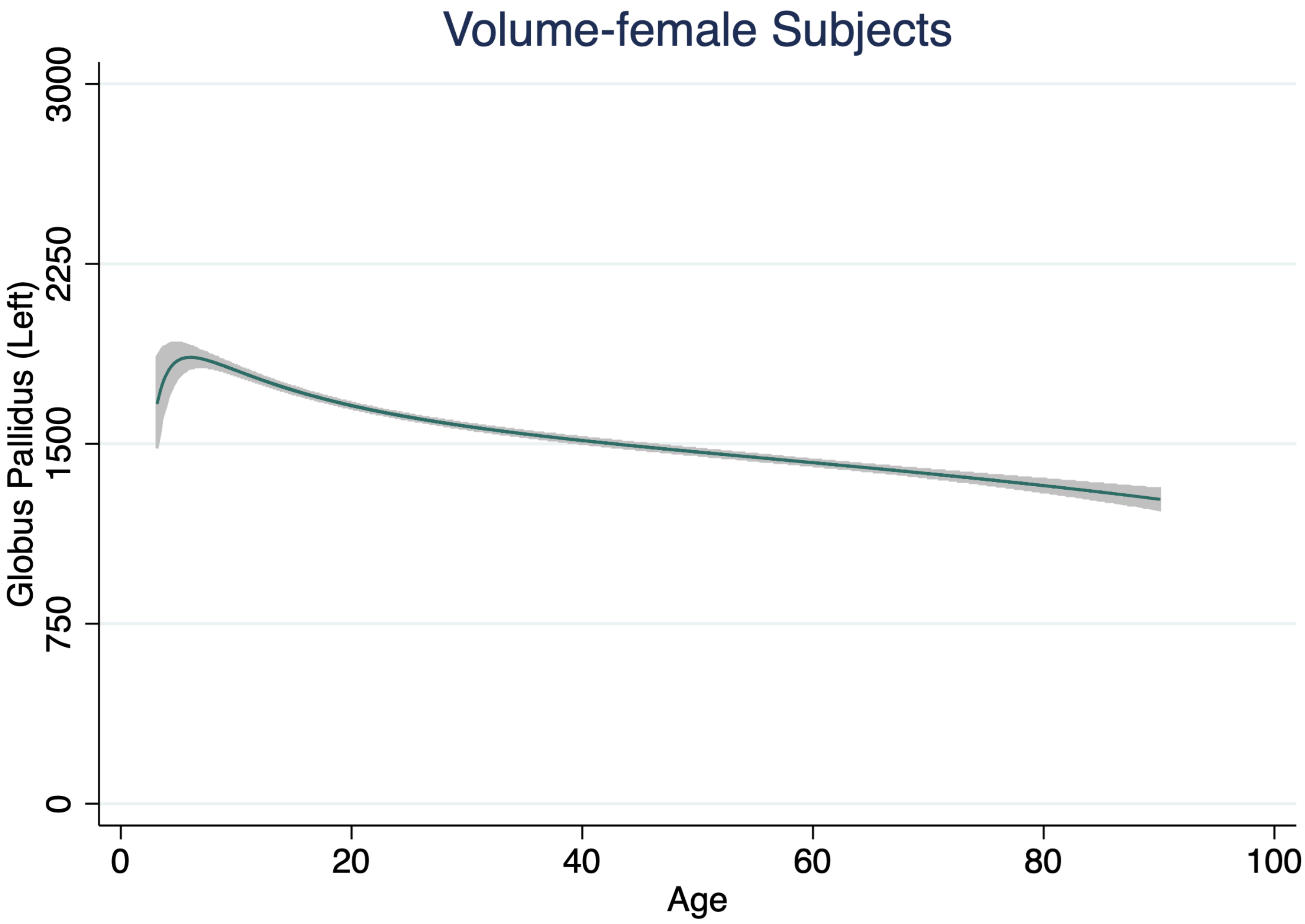

Volume-female Subjects
Globus Pallidus (Left)
3000
2250
1500
750
0
0
20
40
60
80
100
Age

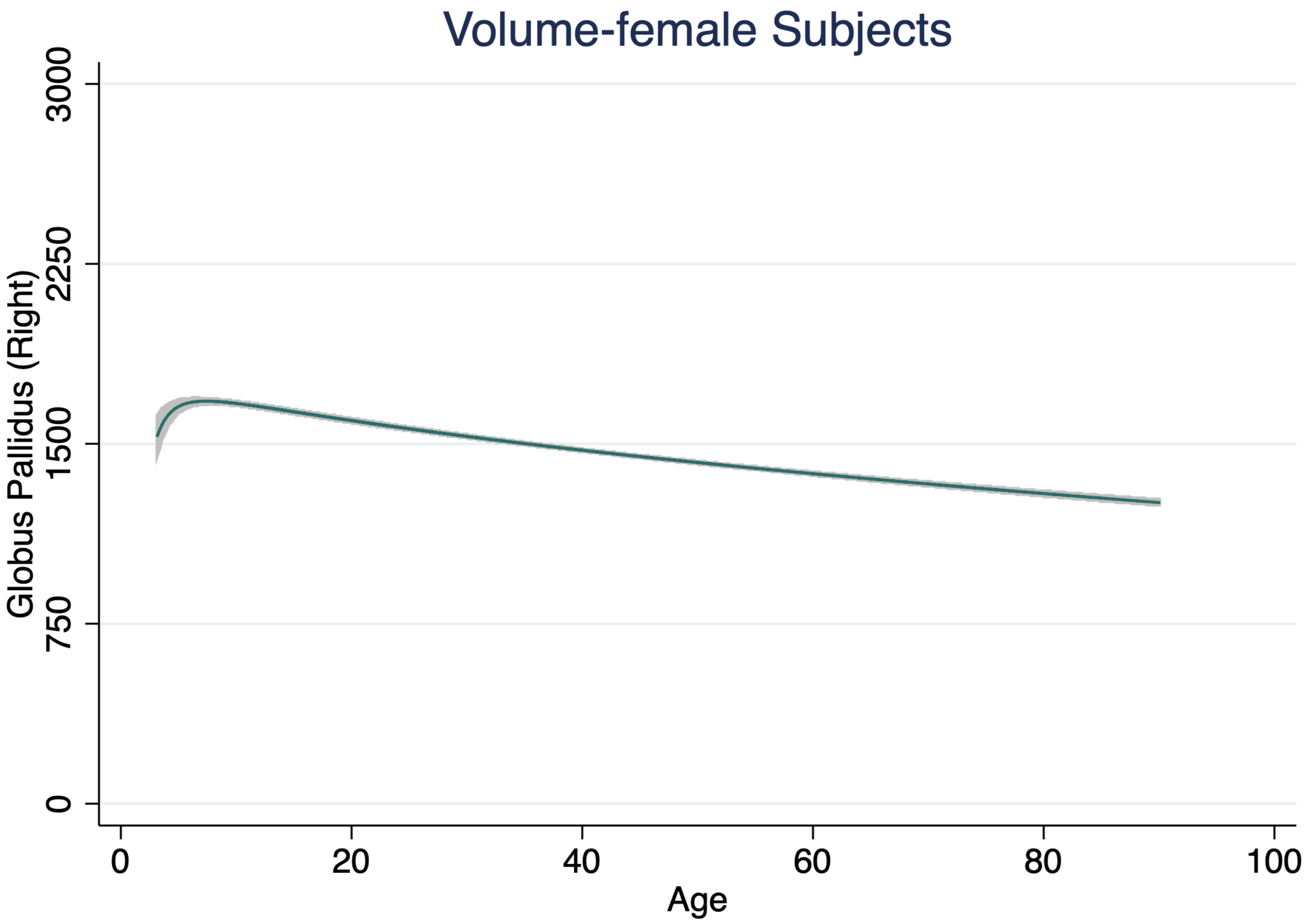
Volume-female Subjects
Globus Pallidus (Right)
0
750
1500
2250
3000
0
20
40
60
80
100
Age

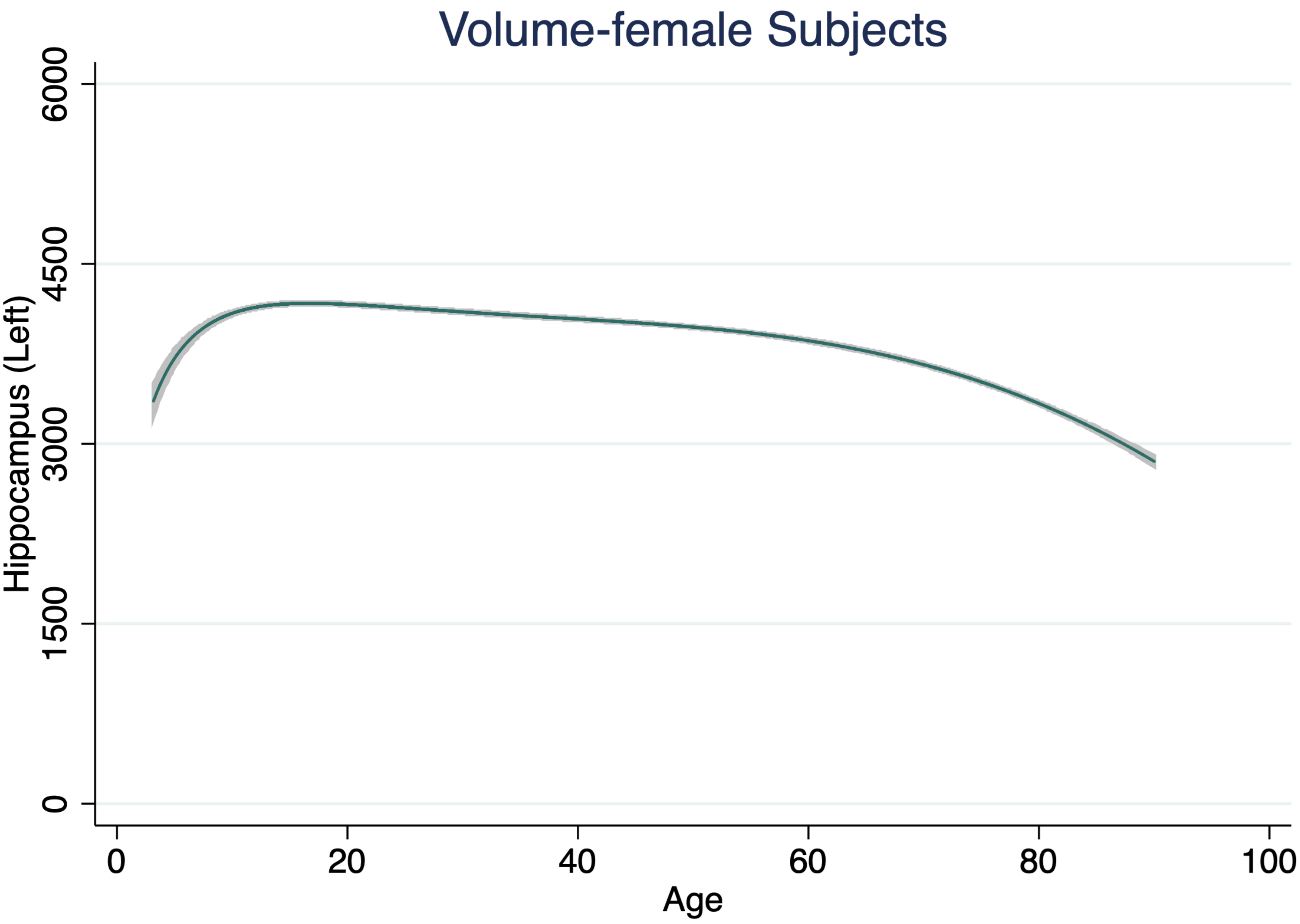
Volume-female Subjects
Hippocampus (Left)
6000
4500
3000
1500
0
0
20
40
60
80
100
Age

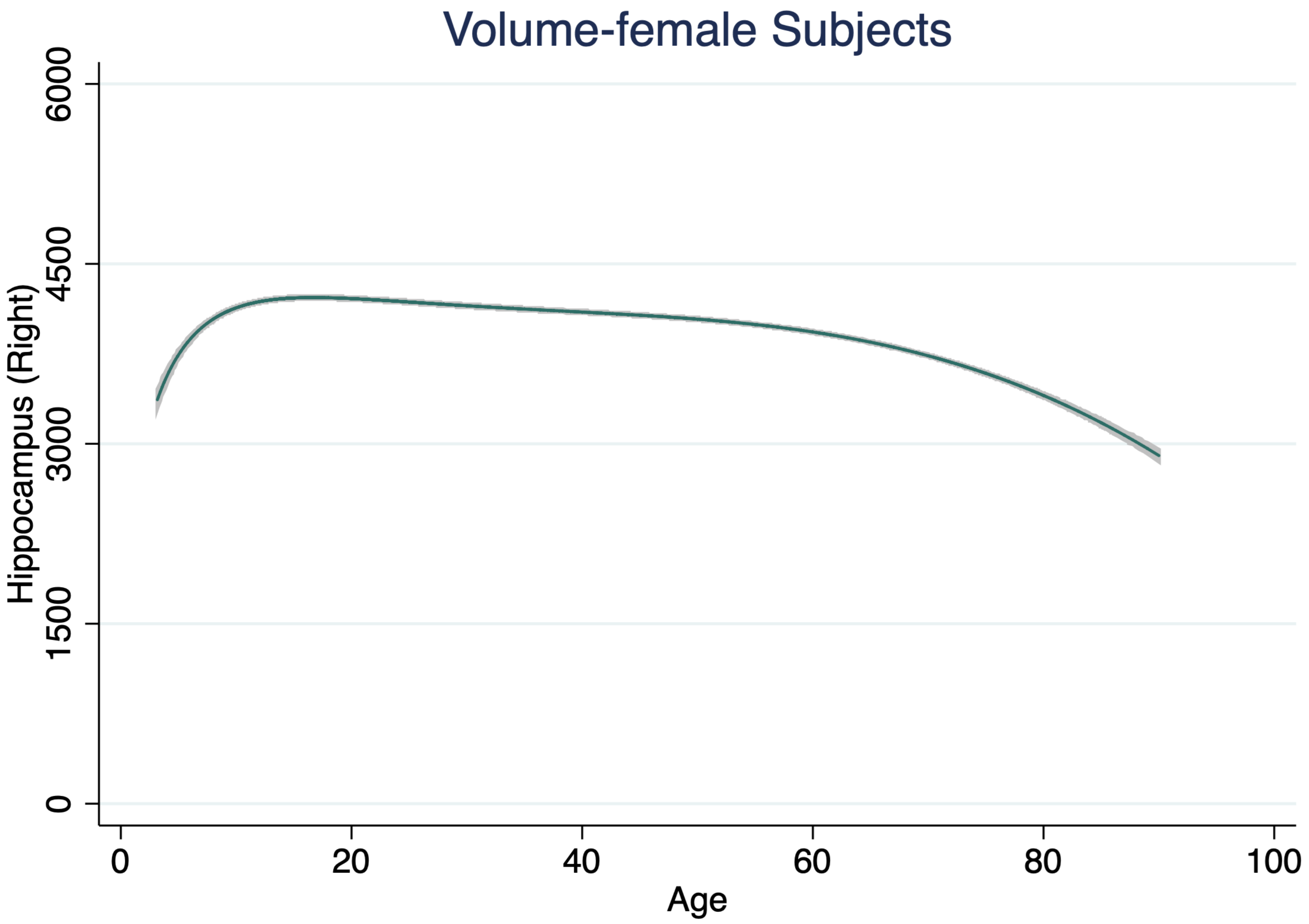
Volume-female Subjects
Hippocampus (Right)
6000
4500
3000
1500
0
0
20
40
60
80
100
Age

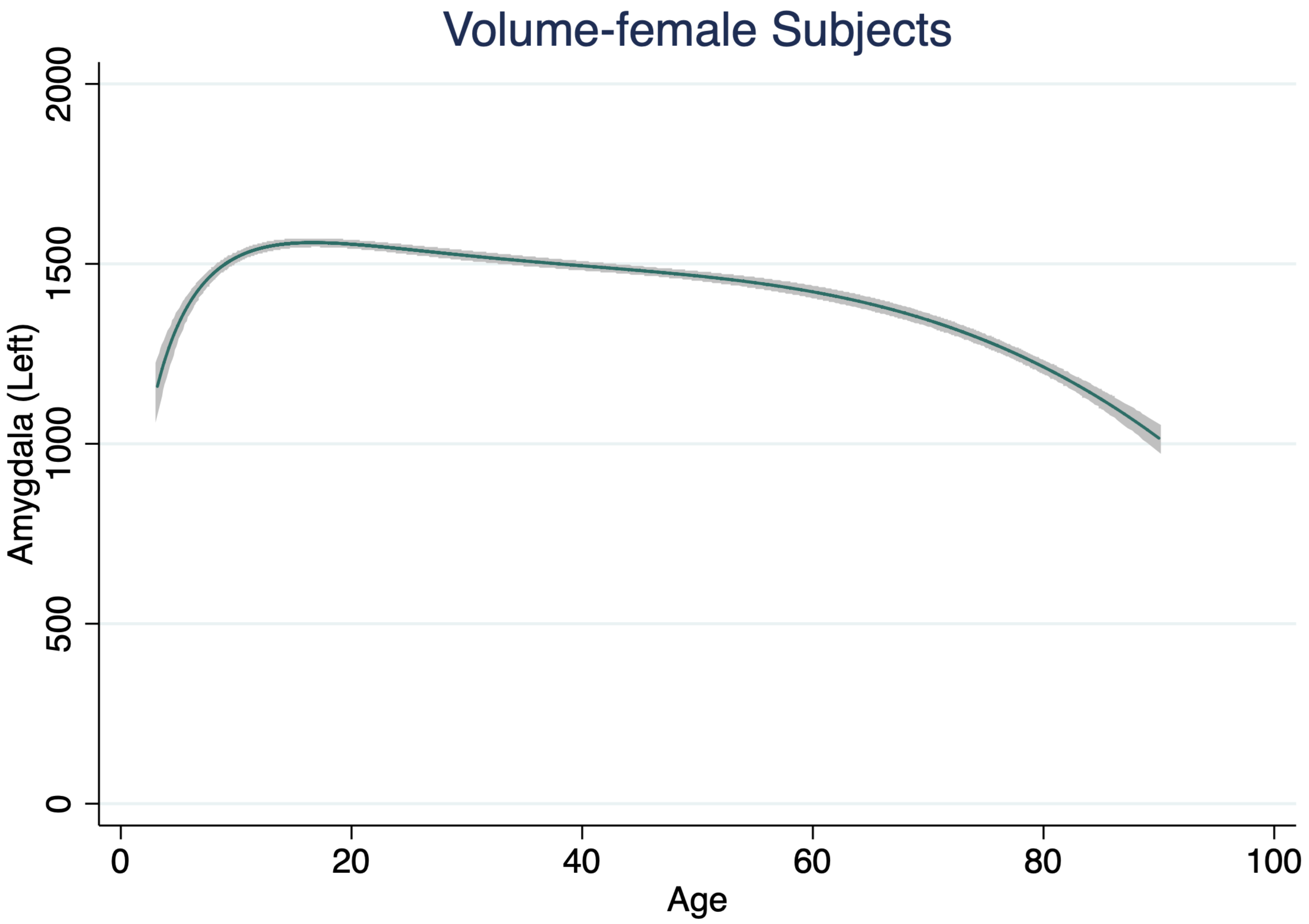

Volume-female Subjects
Amygdala (Left)
0
500
1000
1500
2000
Age
0
20
40
60
80
100

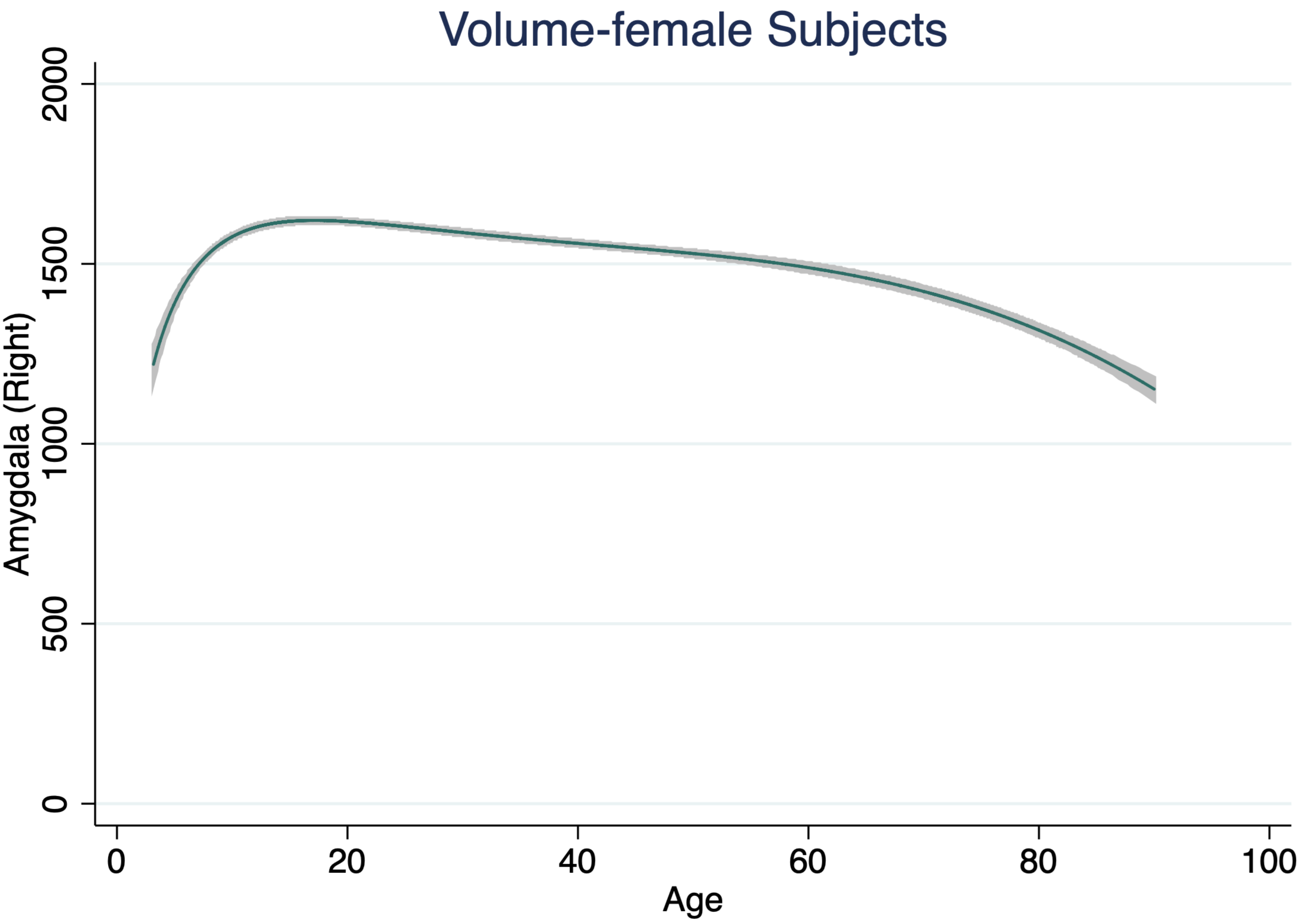

Volume-female Subjects
Amygdala (Right)
2000
1500
1000
500
0
0
20
40
60
80
100
Age

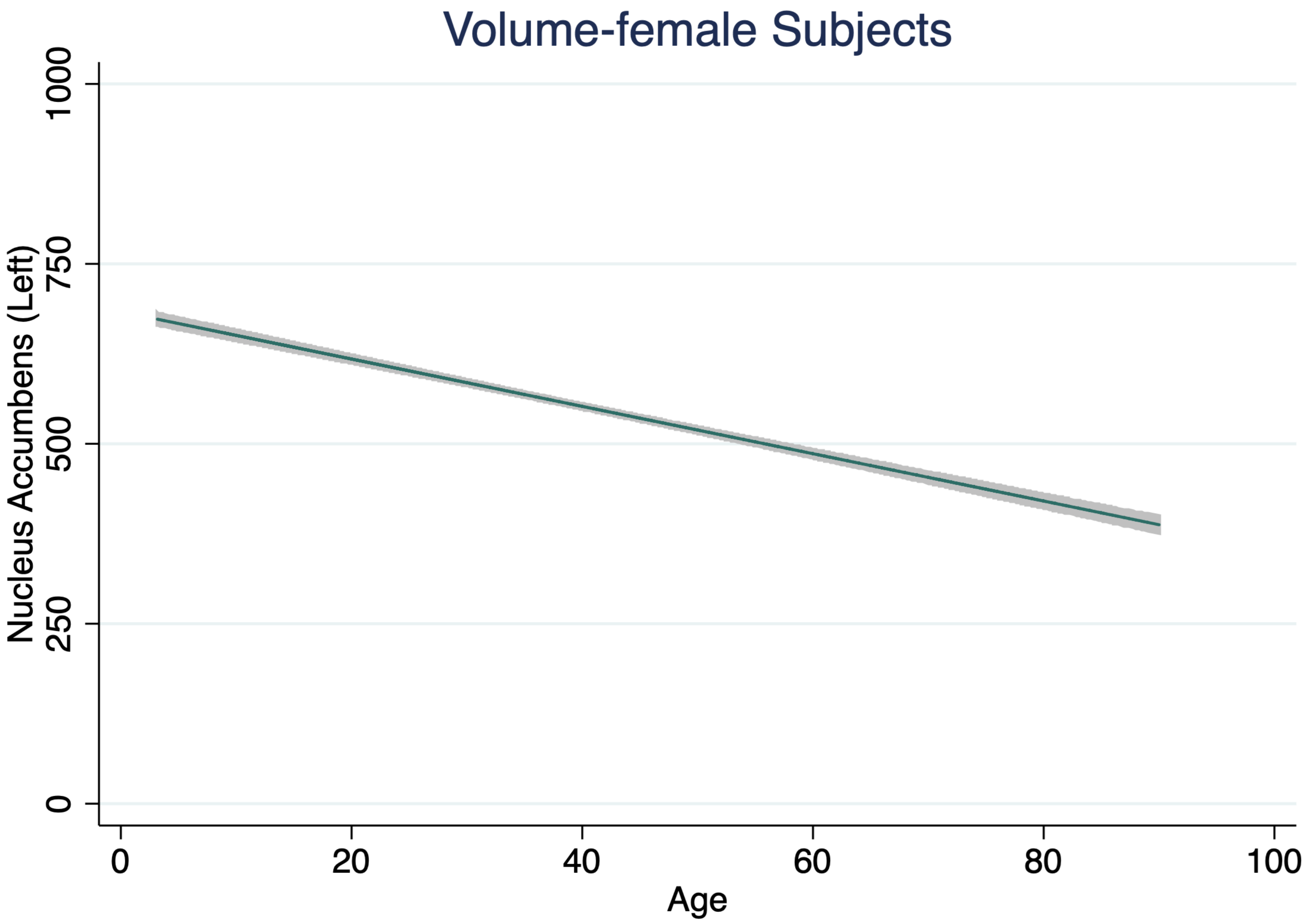

Volume-female Subjects
Nucleus Accumbens (Left)
1000
750
500
250
0
0
20
40
60
80
100
Age

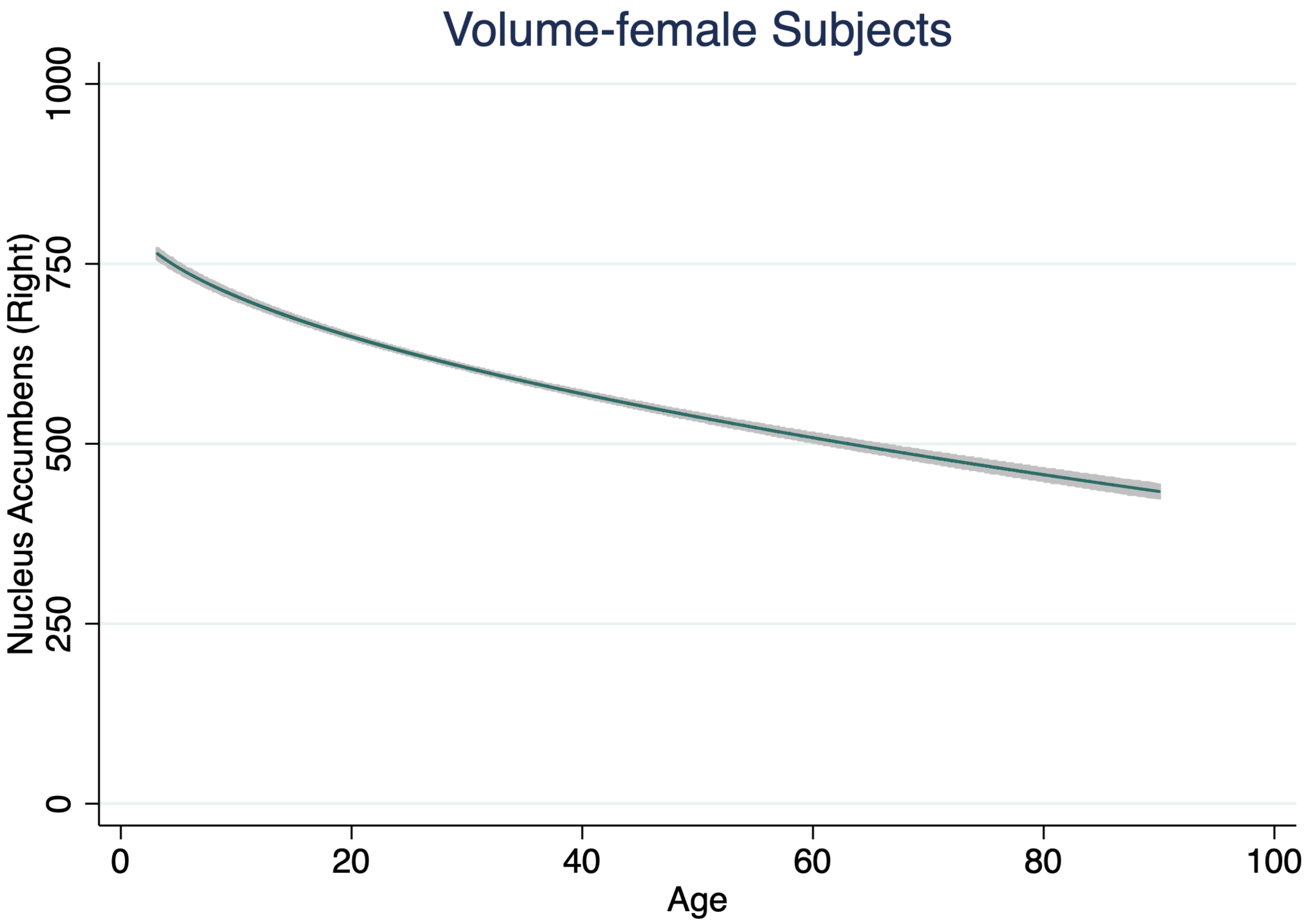

Volume-female Subjects
1000
750
500
250
0
Nucleus Accumbens (Right)
0
20
40
60
80
100
Age
